# Supplementary material for: Use of Ultrasound in Introducing Anatomical Pathology to Preclinical Medical Students, in Correlation with Physical Exam Curricula
Source: MedEdPORTAL. 2020 Sep 25;16:10950. doi: 10.15766/mep_2374-8265.10950 (PMC7521063; doi:10.15766/mep_2374-8265.10950)
Supplement: Supplementary file 1 — Session 1 FAST Exam & the Trauma Patient.pptxSession 2 Cardiac and Lung.pptxSession 3 Gallbladder, Kidneys, & AAA.pptxSession 4 Ocular US & Central Access.pptxSession 1 Instructor Script.docxSession 2 Instructor Script.docxSession 3 Instructor Script.docxSession 4 Instructor Script.docxSurvey Questions.docx [file mep_2374-8265.10950-s001.zip › A. Session 1 FAST Exam & the Trauma Patient.pptx]

## Slide 1
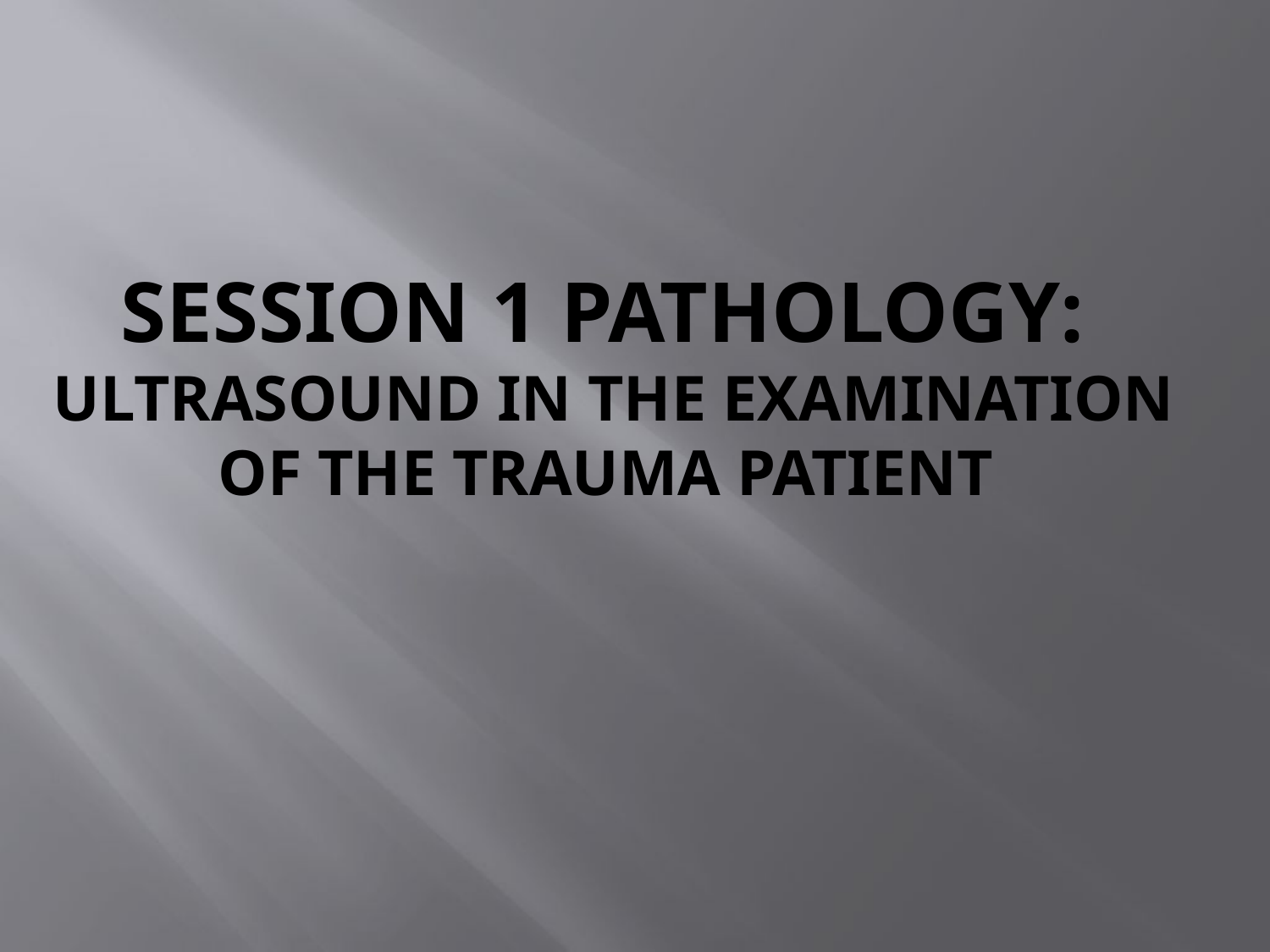

# Session 1 Pathology: Ultrasound in the examination of the Trauma patient

## Slide 2
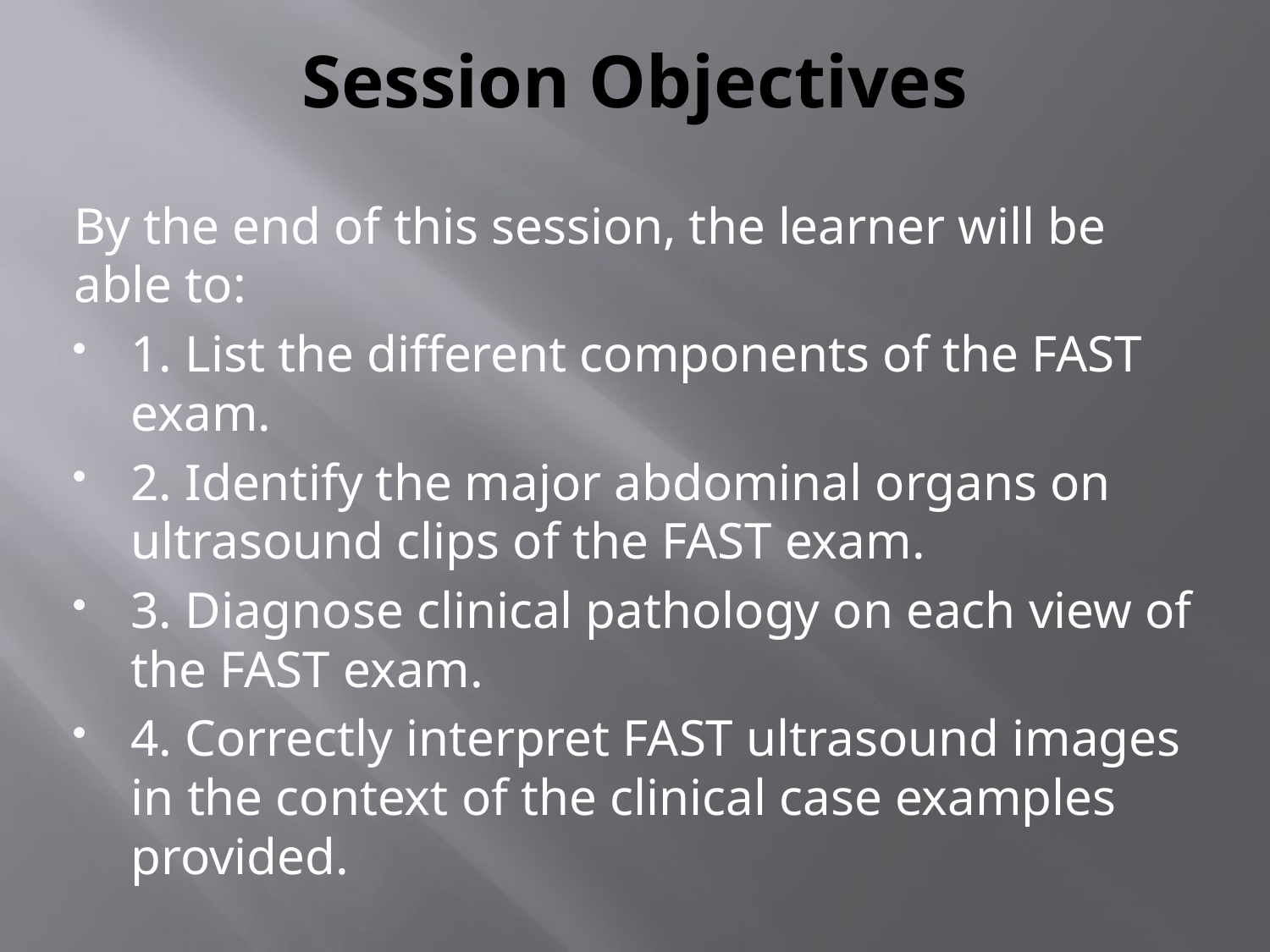

# Session Objectives
By the end of this session, the learner will be able to:
1. List the different components of the FAST exam.
2. Identify the major abdominal organs on ultrasound clips of the FAST exam.
3. Diagnose clinical pathology on each view of the FAST exam.
4. Correctly interpret FAST ultrasound images in the context of the clinical case examples provided.

## Slide 3
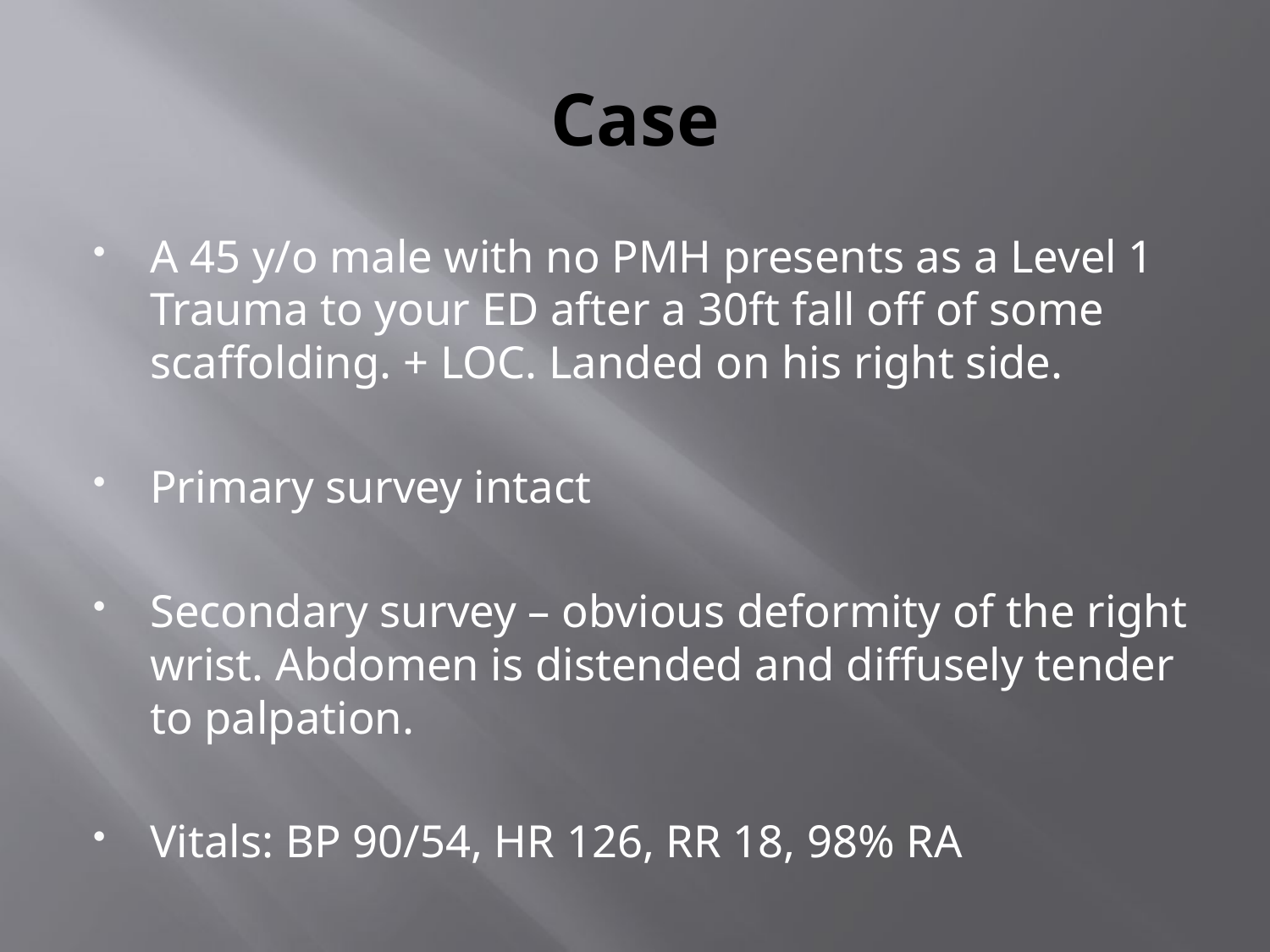

# Case
A 45 y/o male with no PMH presents as a Level 1 Trauma to your ED after a 30ft fall off of some scaffolding. + LOC. Landed on his right side.
Primary survey intact
Secondary survey – obvious deformity of the right wrist. Abdomen is distended and diffusely tender to palpation.
Vitals: BP 90/54, HR 126, RR 18, 98% RA

## Slide 4
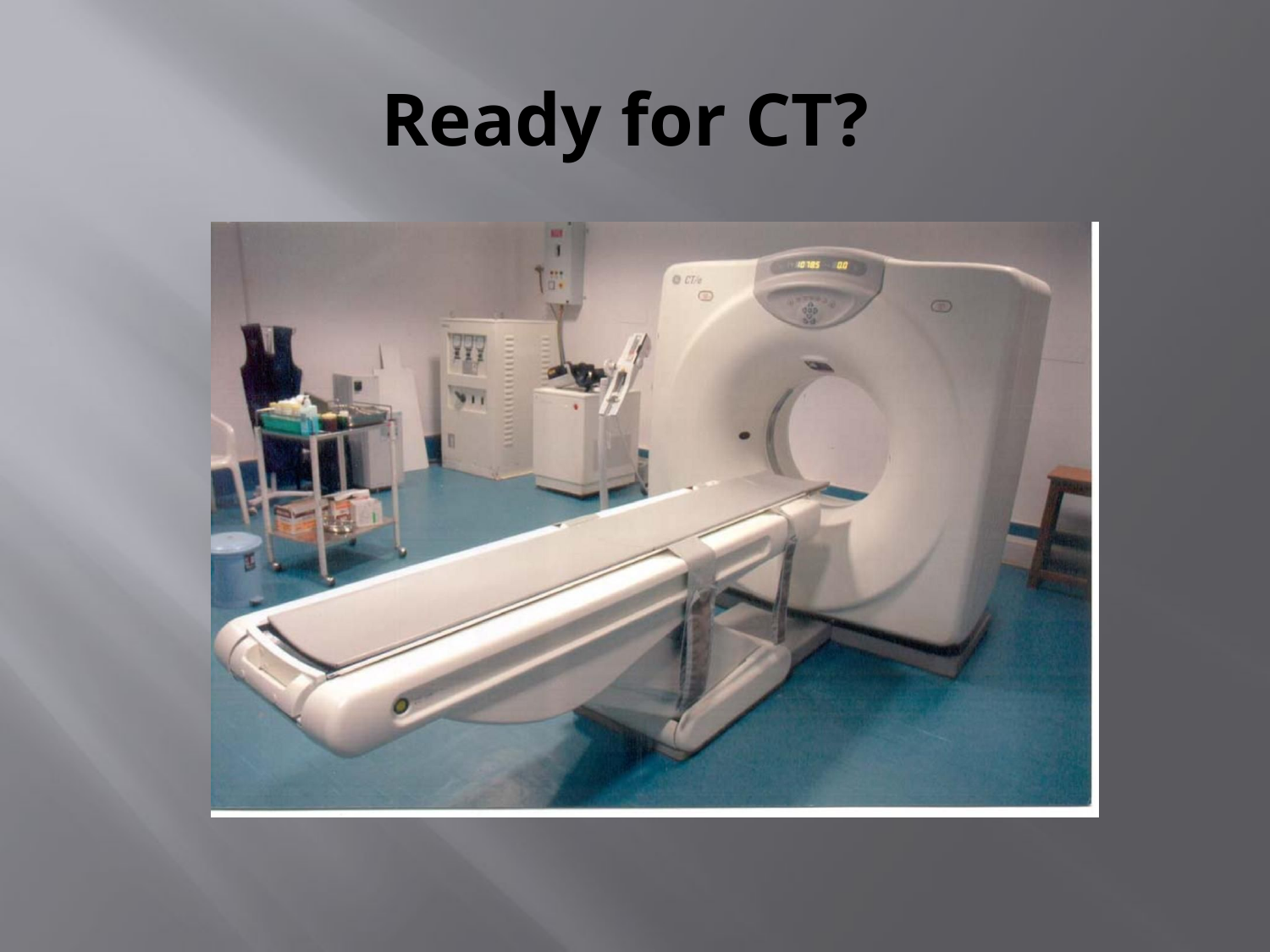

# Ready for CT?

## Slide 5
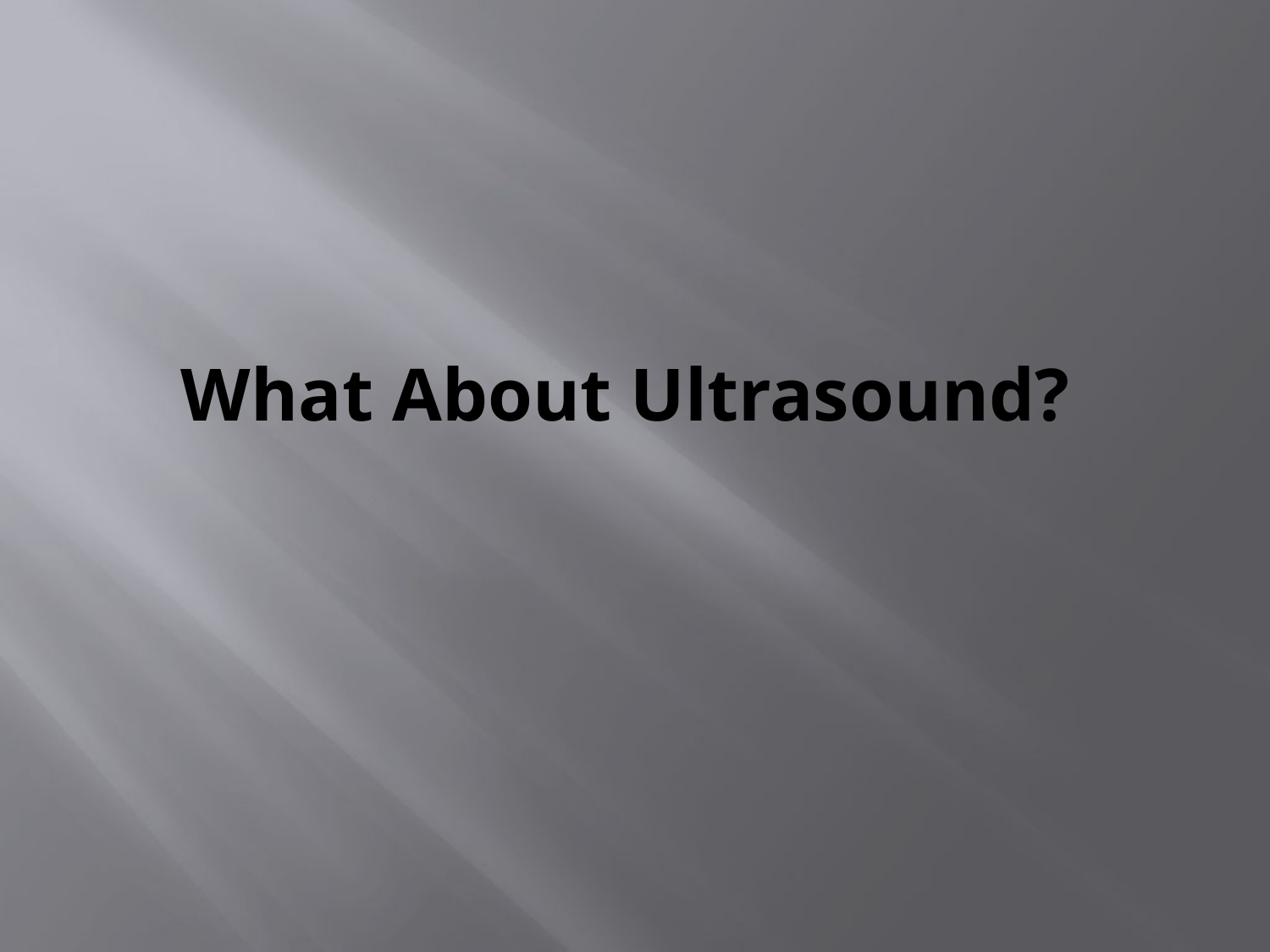

# What About Ultrasound?

## Slide 6
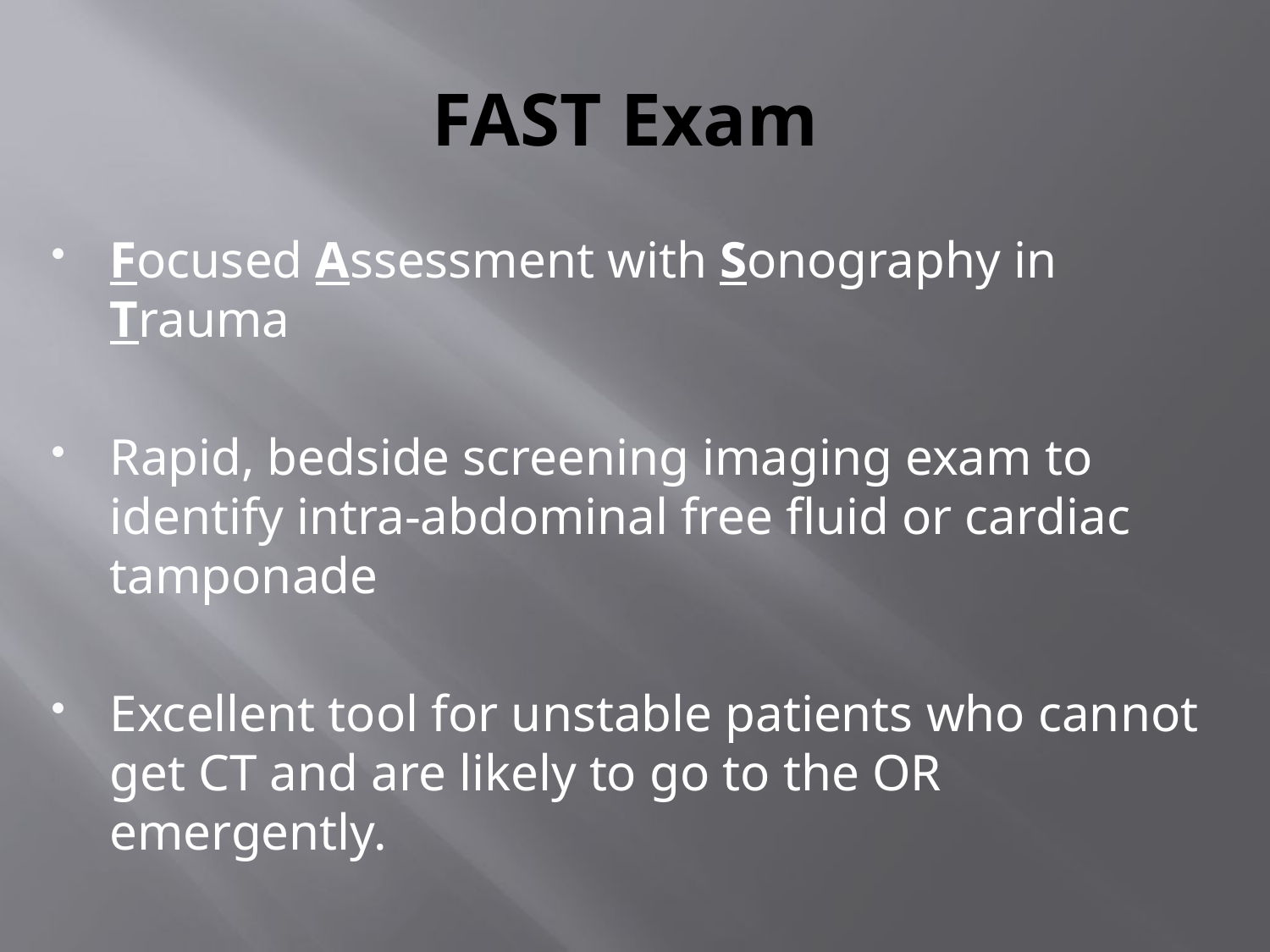

# FAST Exam
Focused Assessment with Sonography in Trauma
Rapid, bedside screening imaging exam to identify intra-abdominal free fluid or cardiac tamponade
Excellent tool for unstable patients who cannot get CT and are likely to go to the OR emergently.

## Slide 7
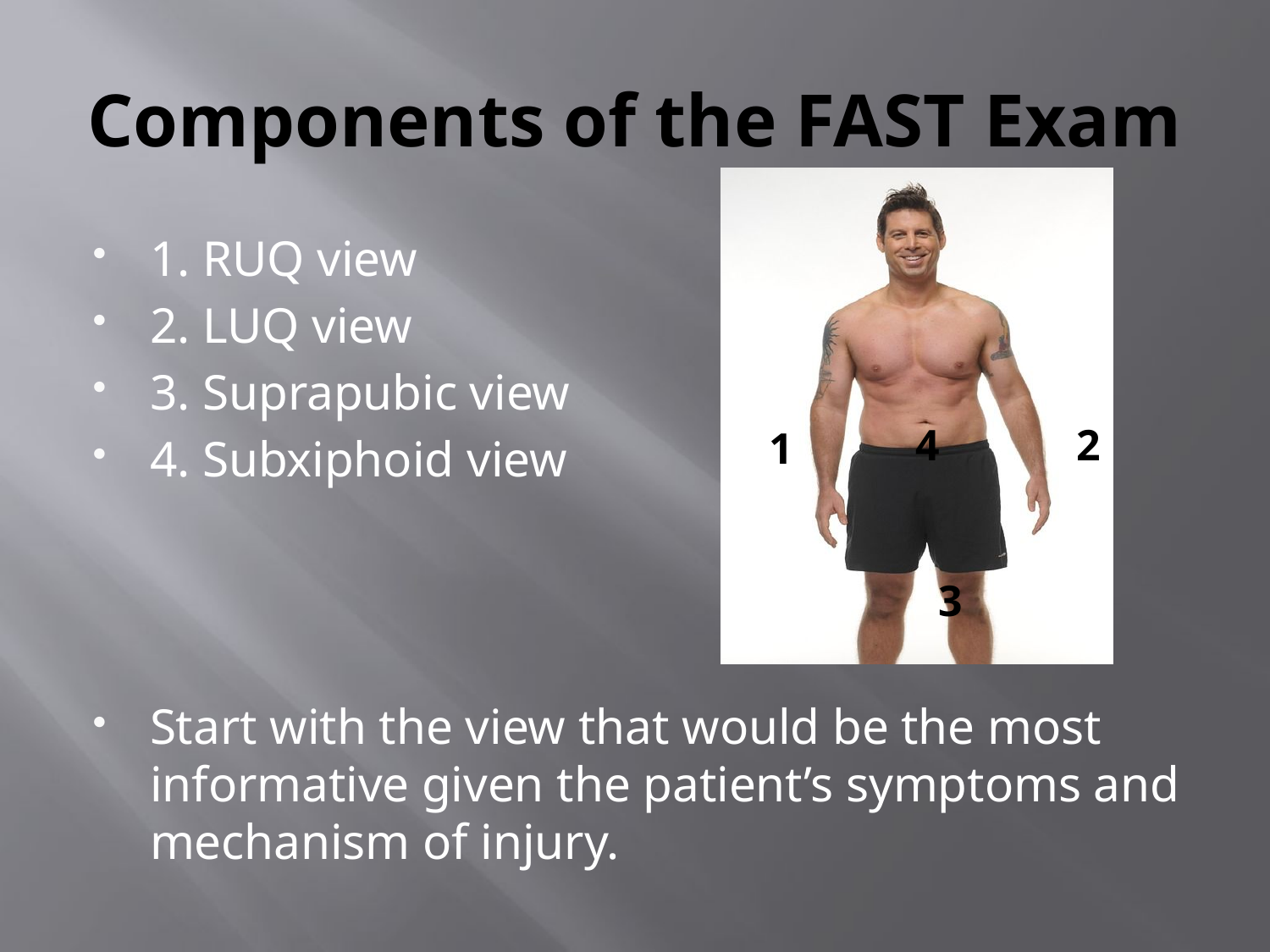

# Components of the FAST Exam
1. RUQ view
2. LUQ view
3. Suprapubic view
4. Subxiphoid view
Start with the view that would be the most informative given the patient’s symptoms and mechanism of injury.
4
2
1
3

## Slide 8
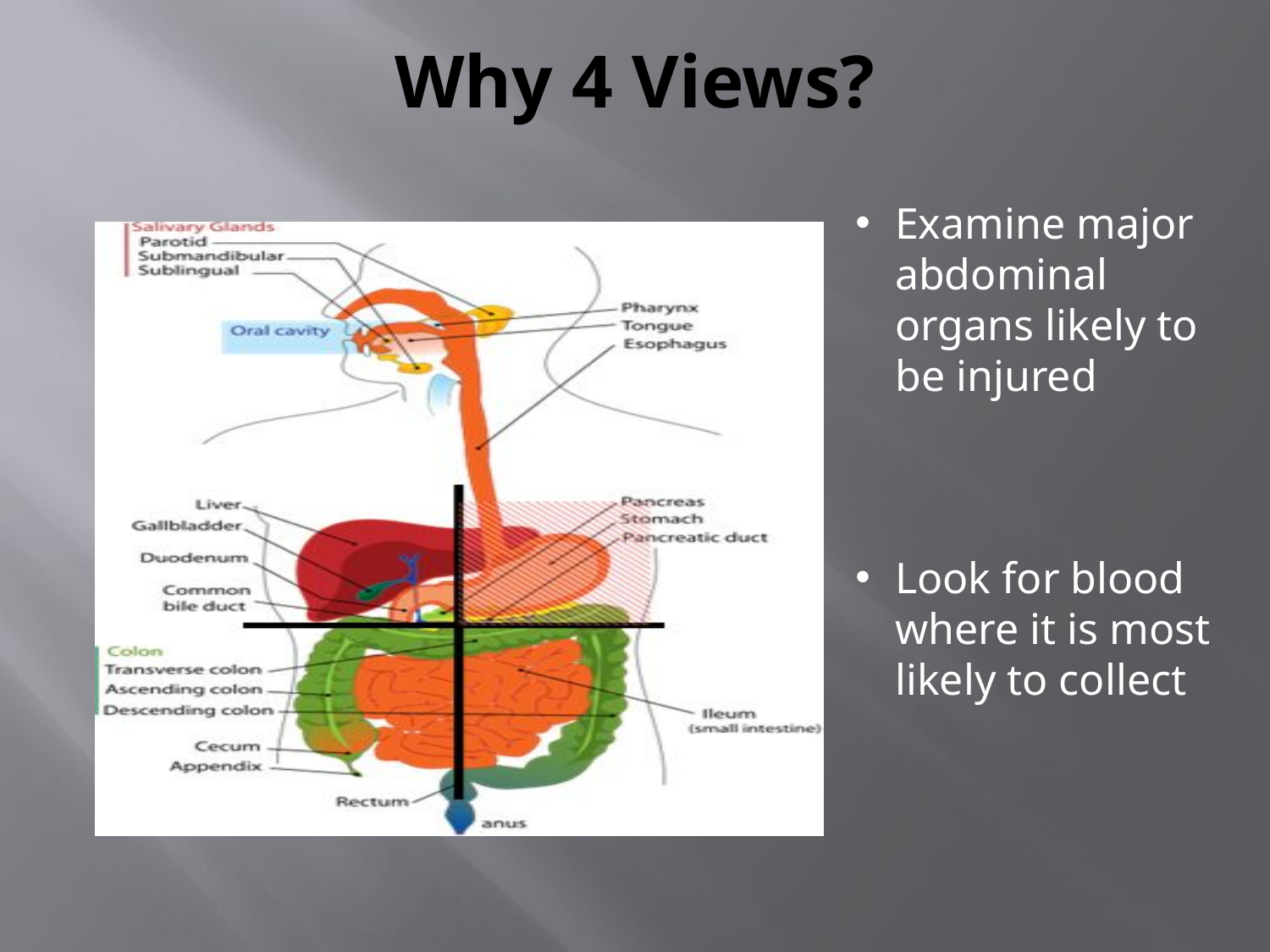

# Why 4 Views?
Examine major abdominal organs likely to be injured
Look for blood where it is most likely to collect

## Slide 9
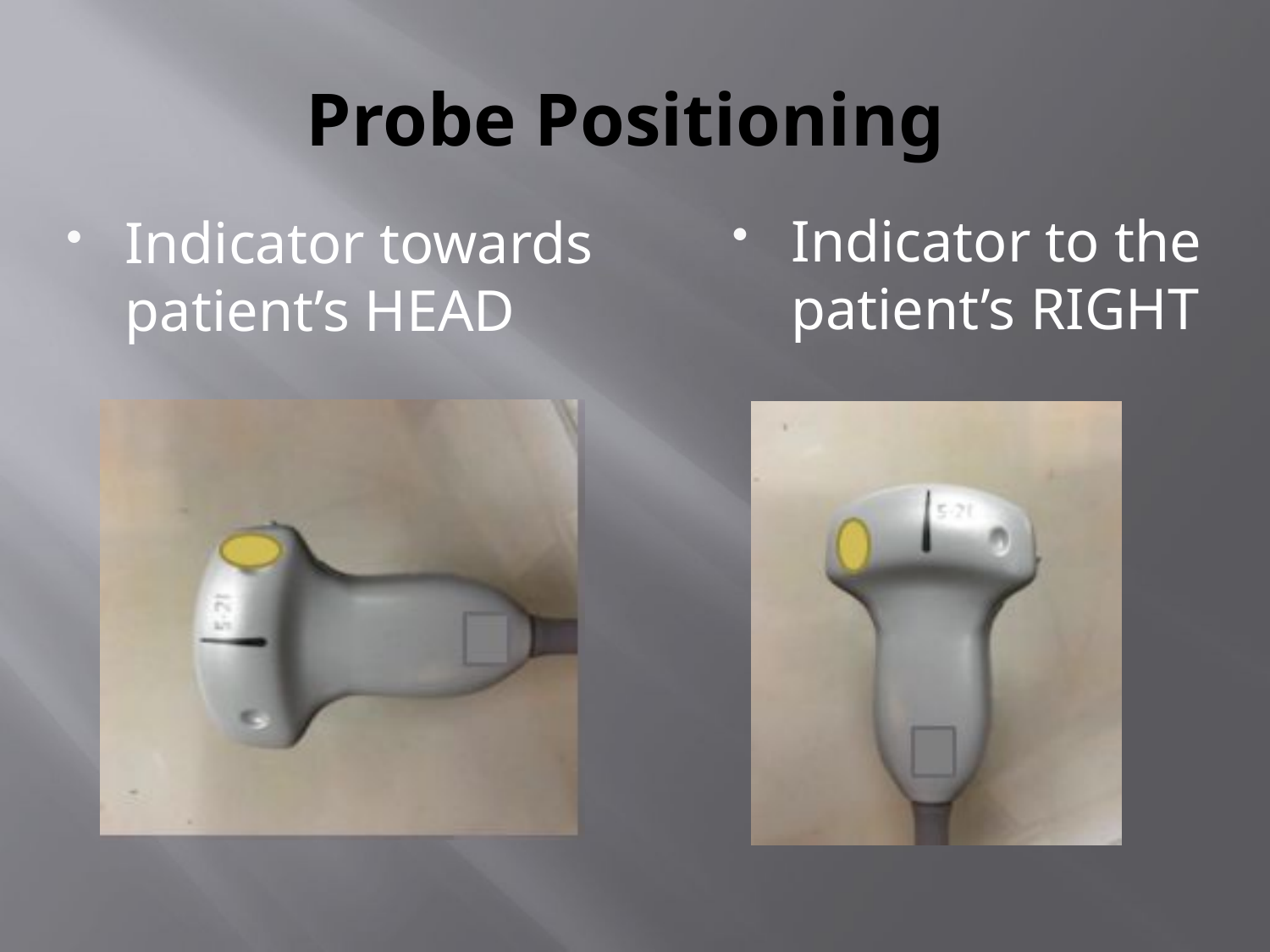

# Probe Positioning
Indicator to the patient’s RIGHT
Indicator towards patient’s HEAD

## Slide 10
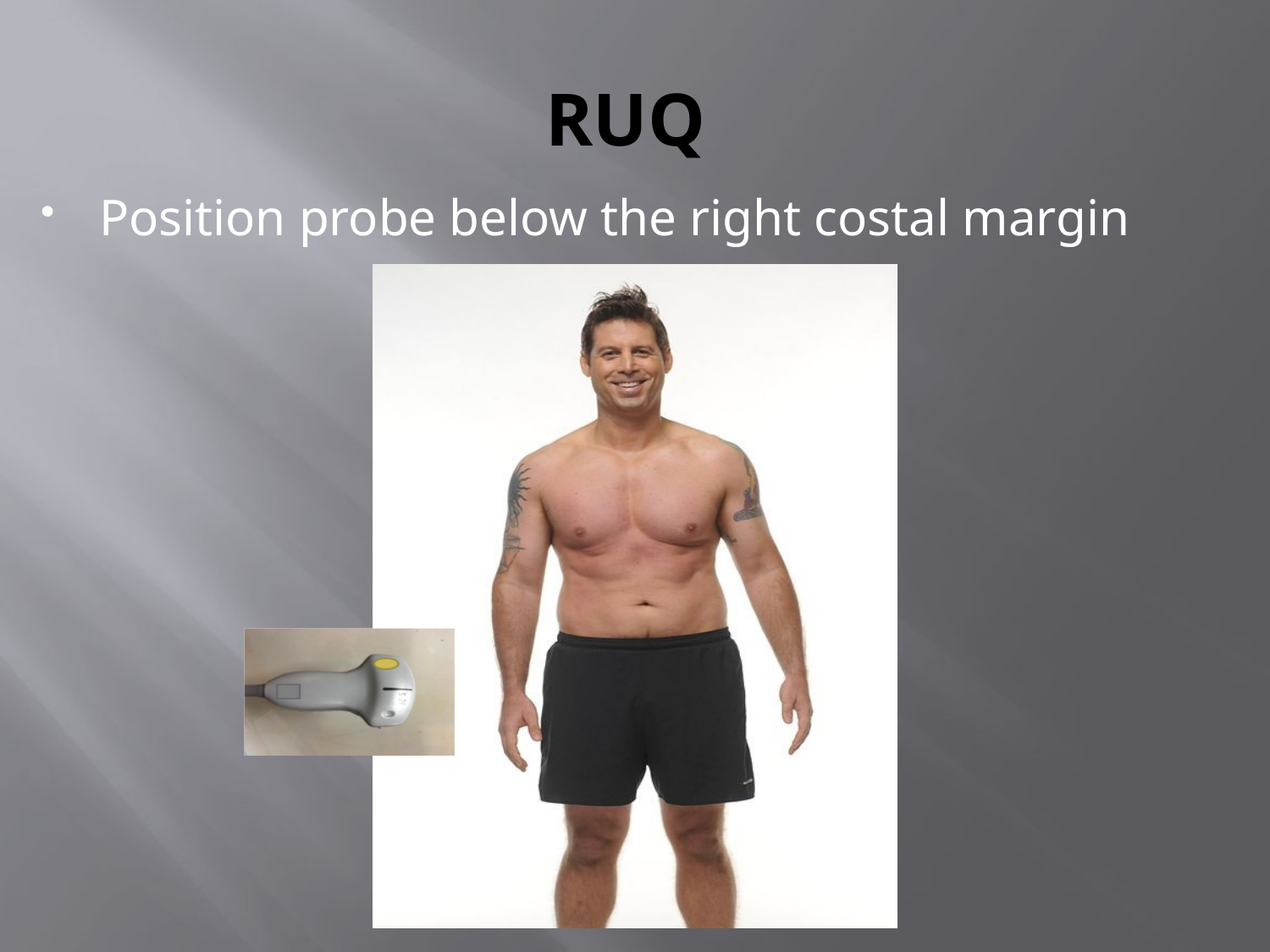

# RUQ
Position probe below the right costal margin

## Slide 11
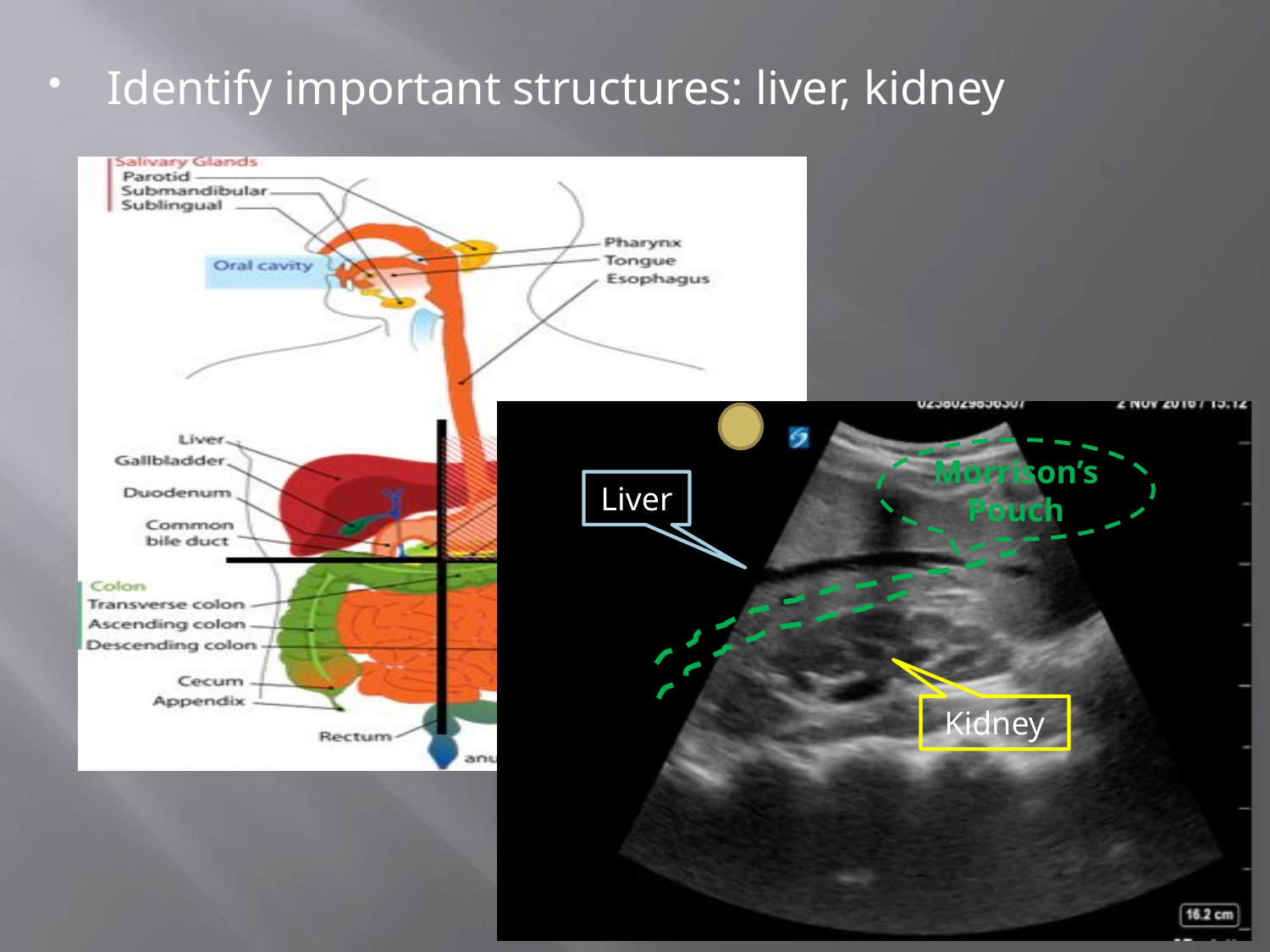

Identify important structures: liver, kidney
Morrison’s Pouch
Liver
Kidney

## Slide 12
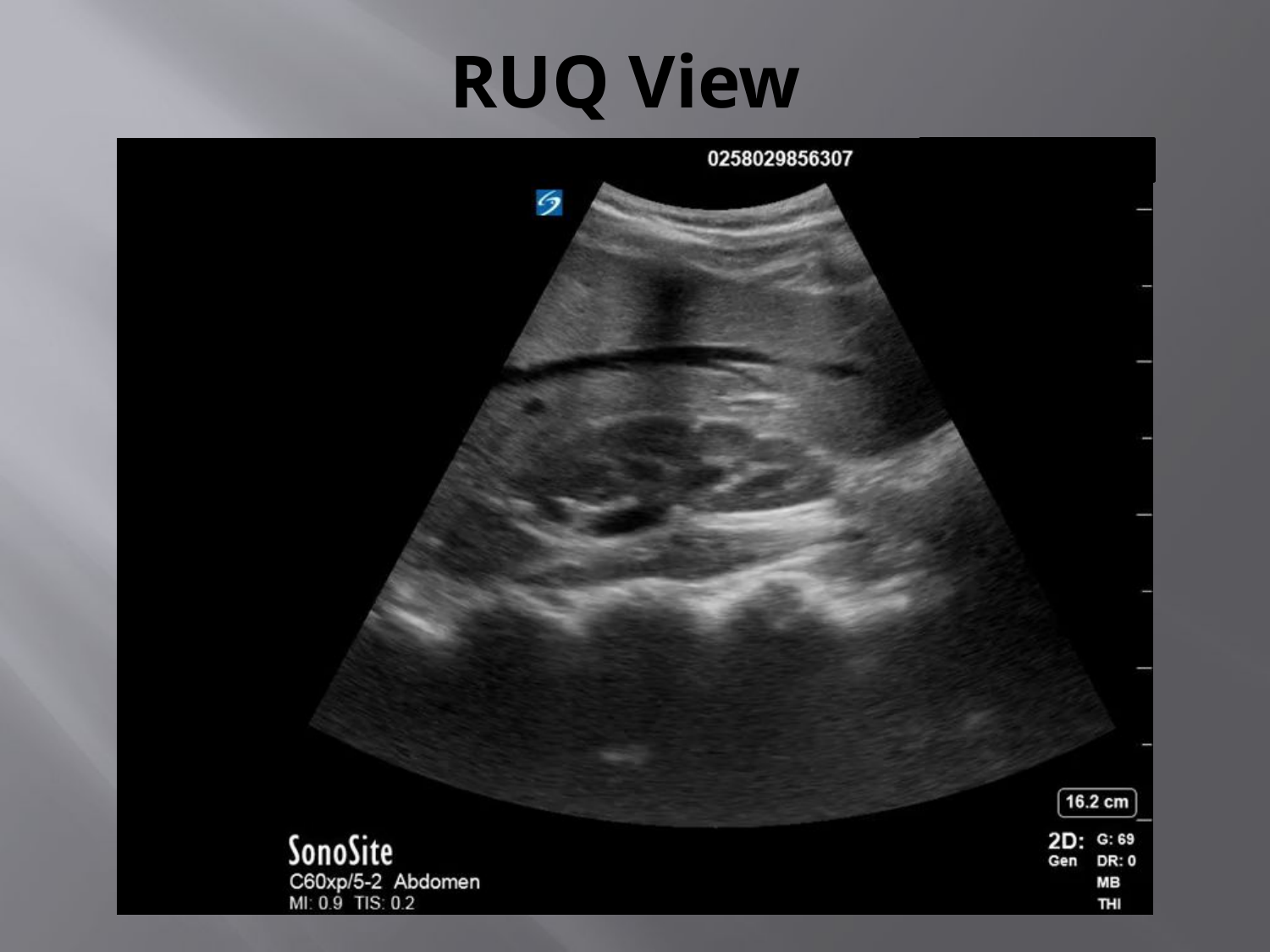

# RUQ View

## Slide 13
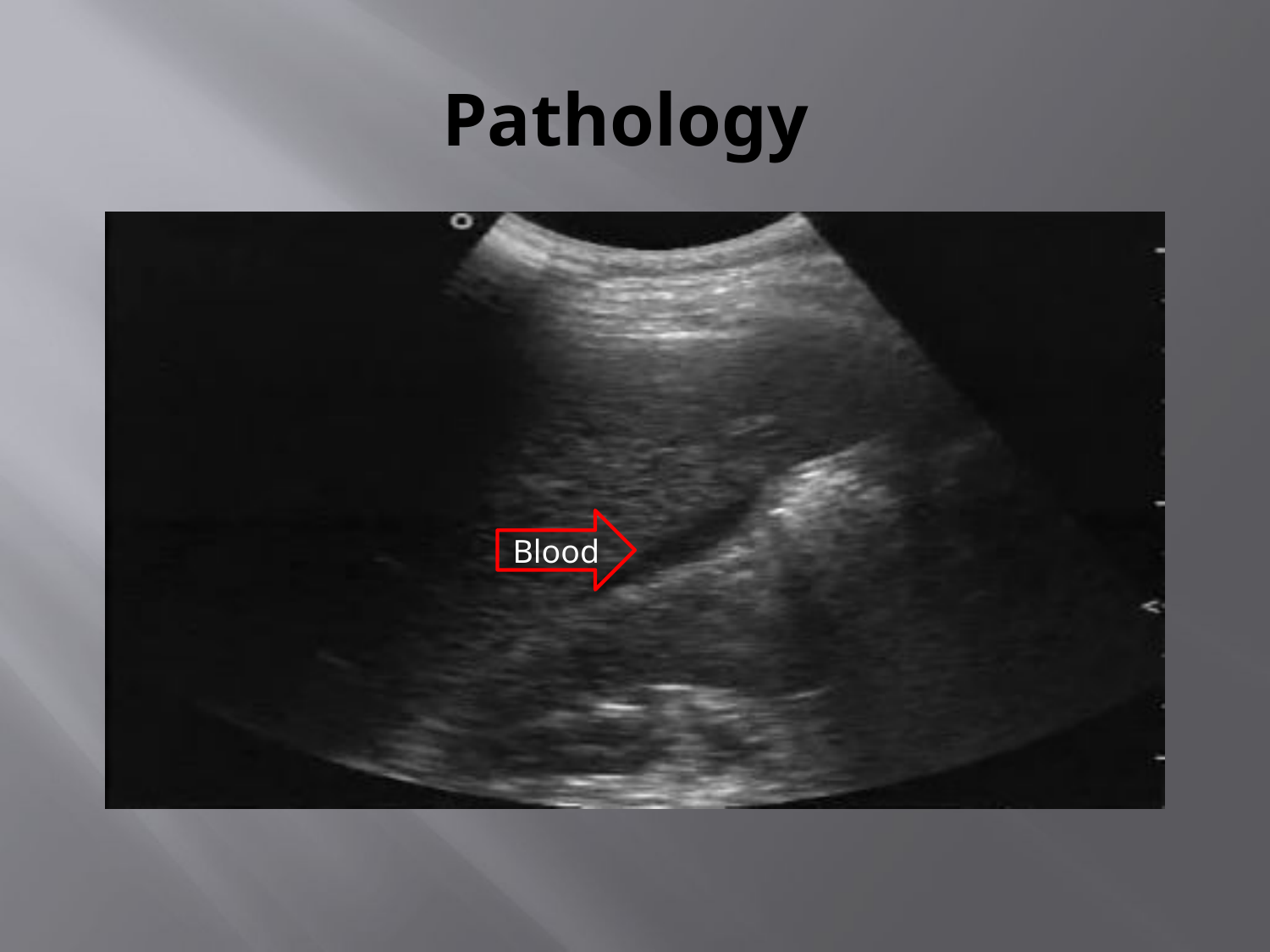

# Pathology
Blood

## Slide 14
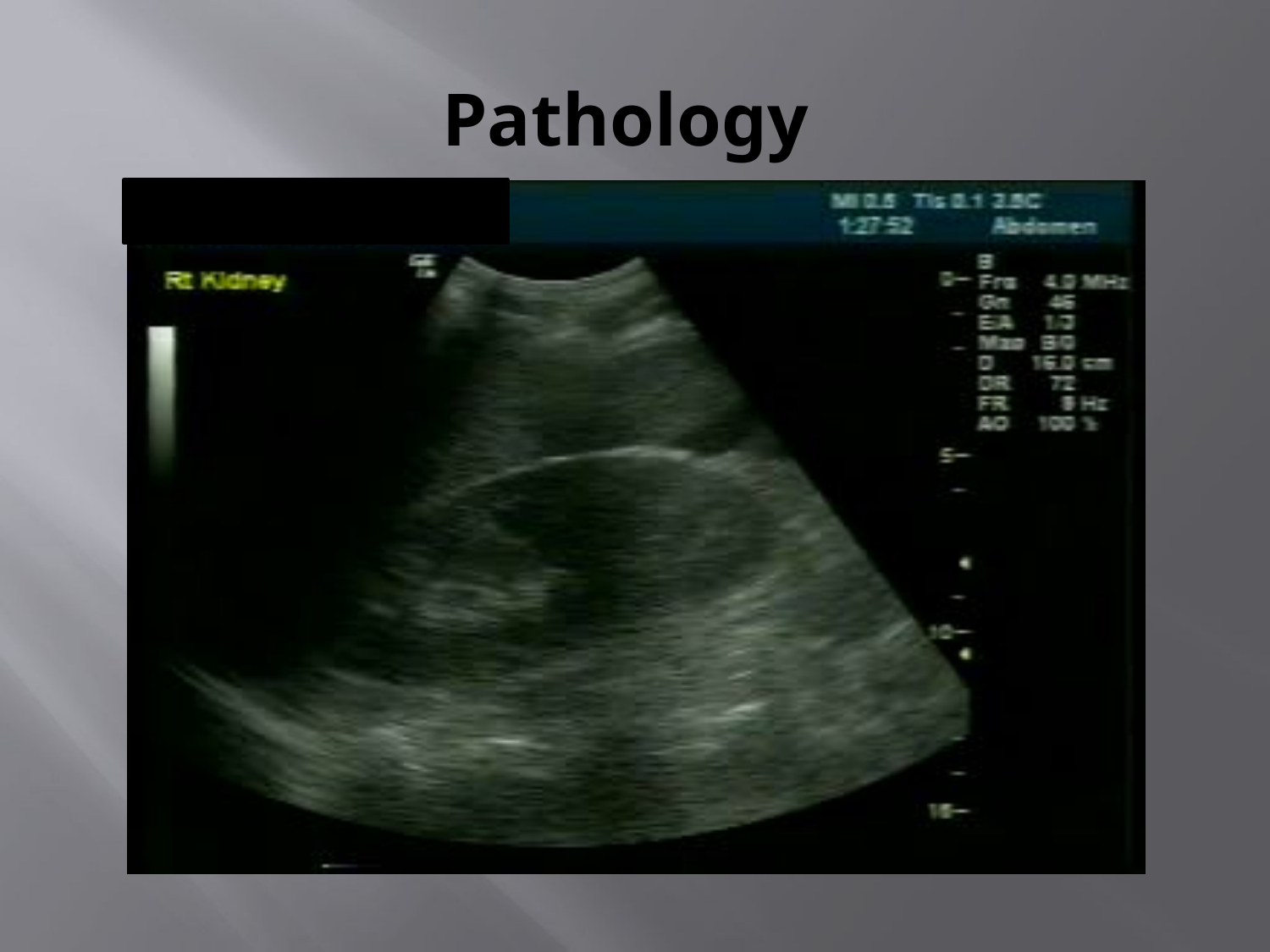

# Pathology

## Slide 15
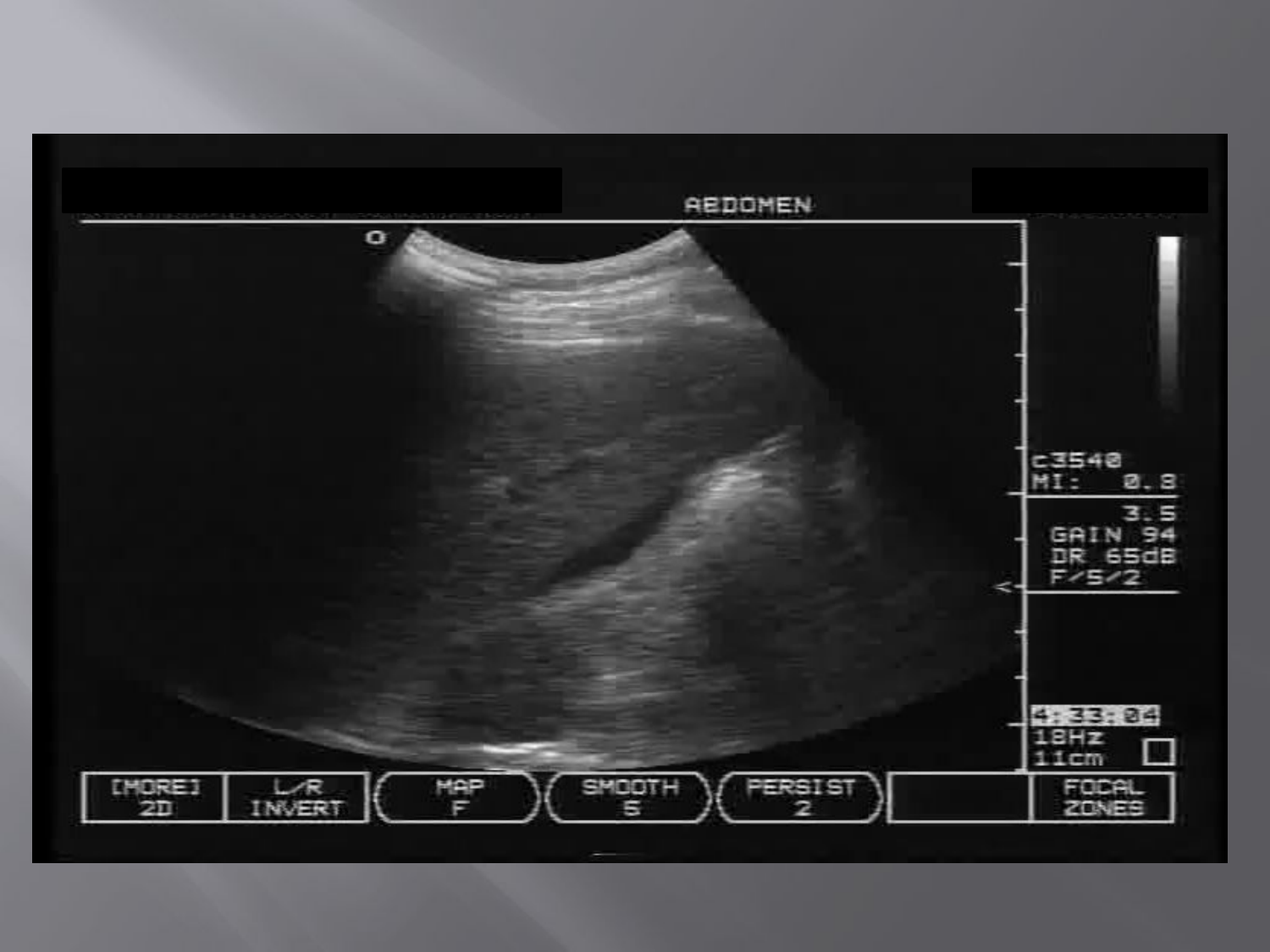

## Slide 16
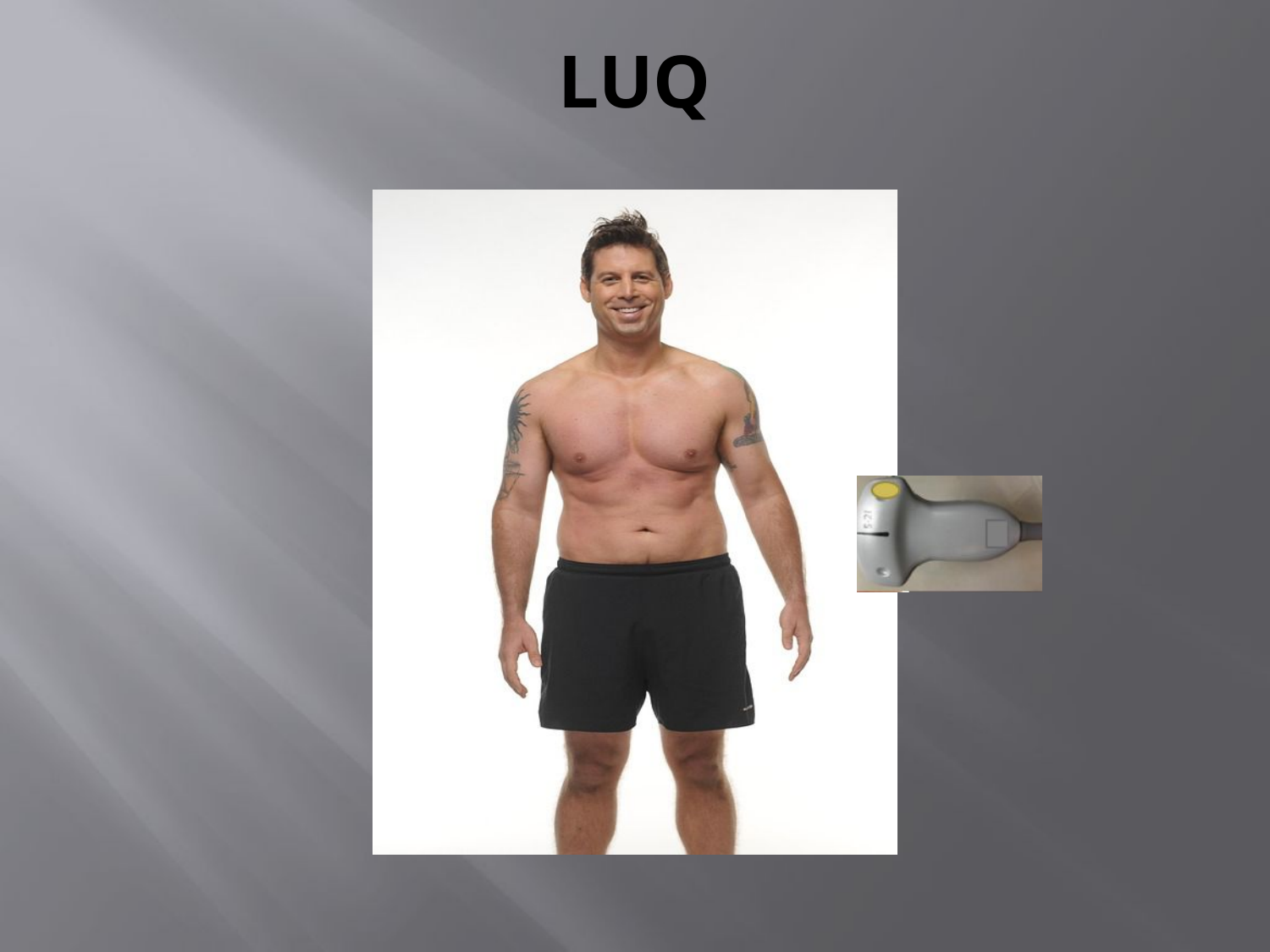

# LUQ

## Slide 17
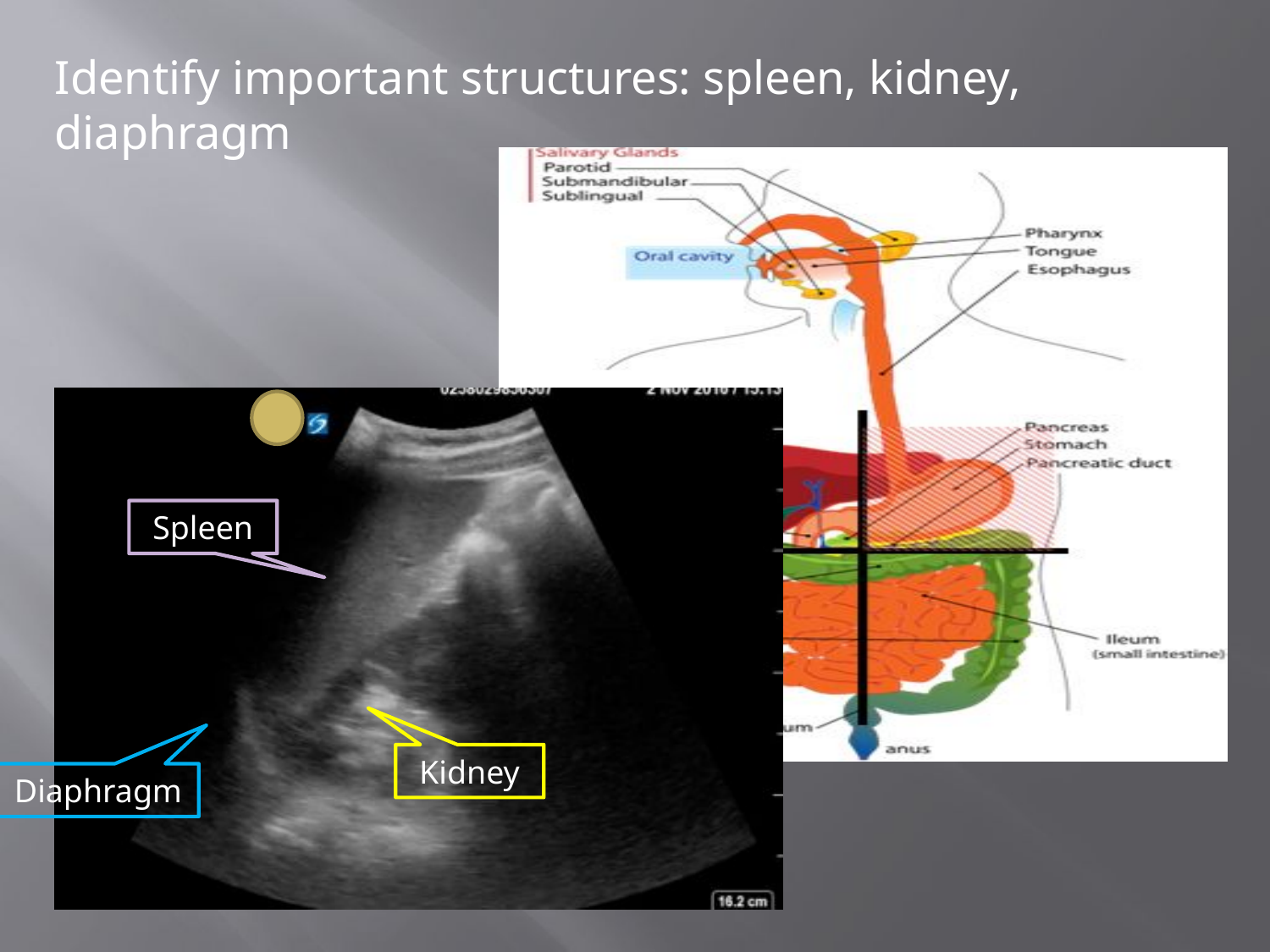

Identify important structures: spleen, kidney, diaphragm
Spleen
Kidney
Diaphragm

## Slide 18
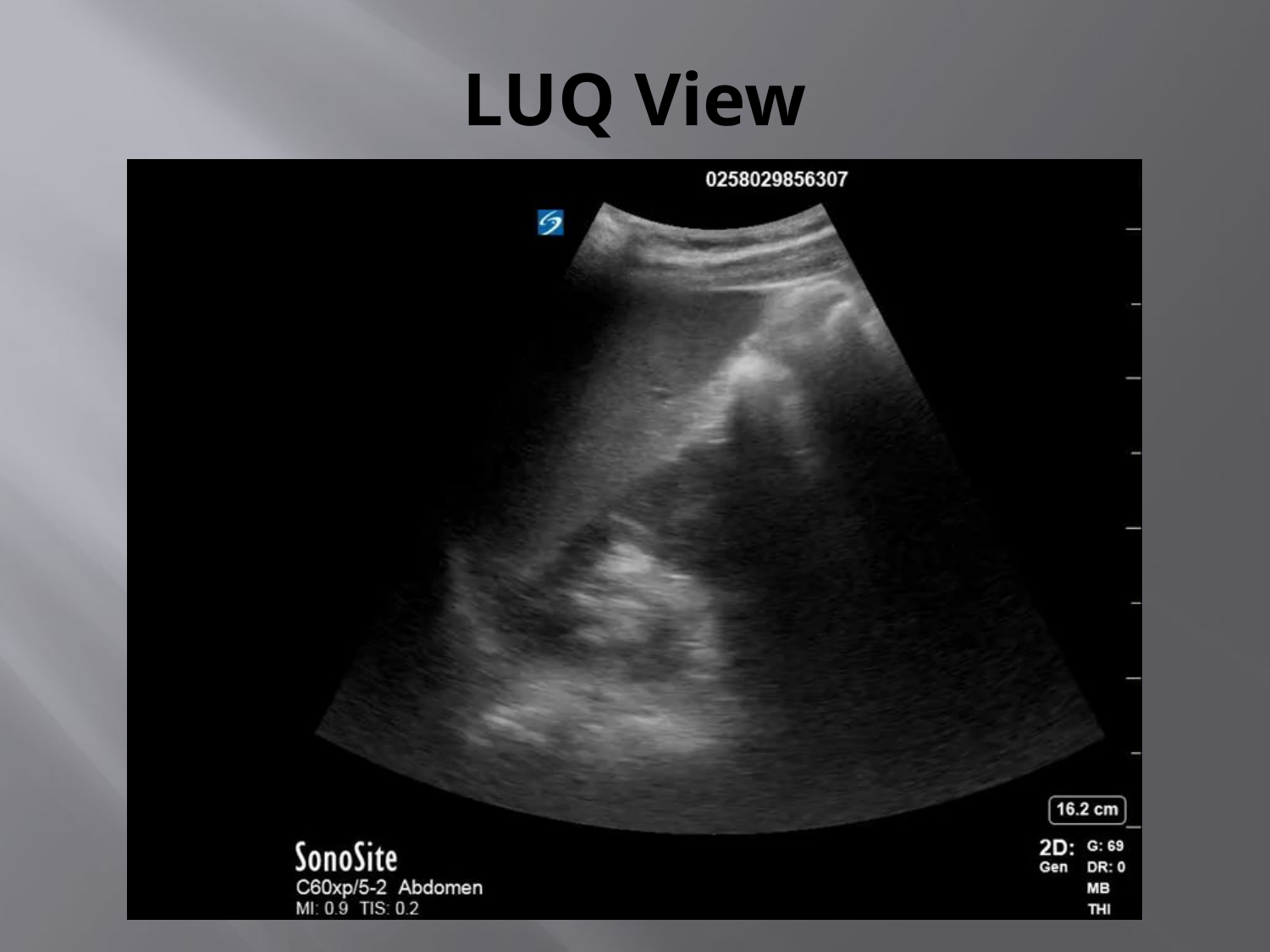

# LUQ View

## Slide 19
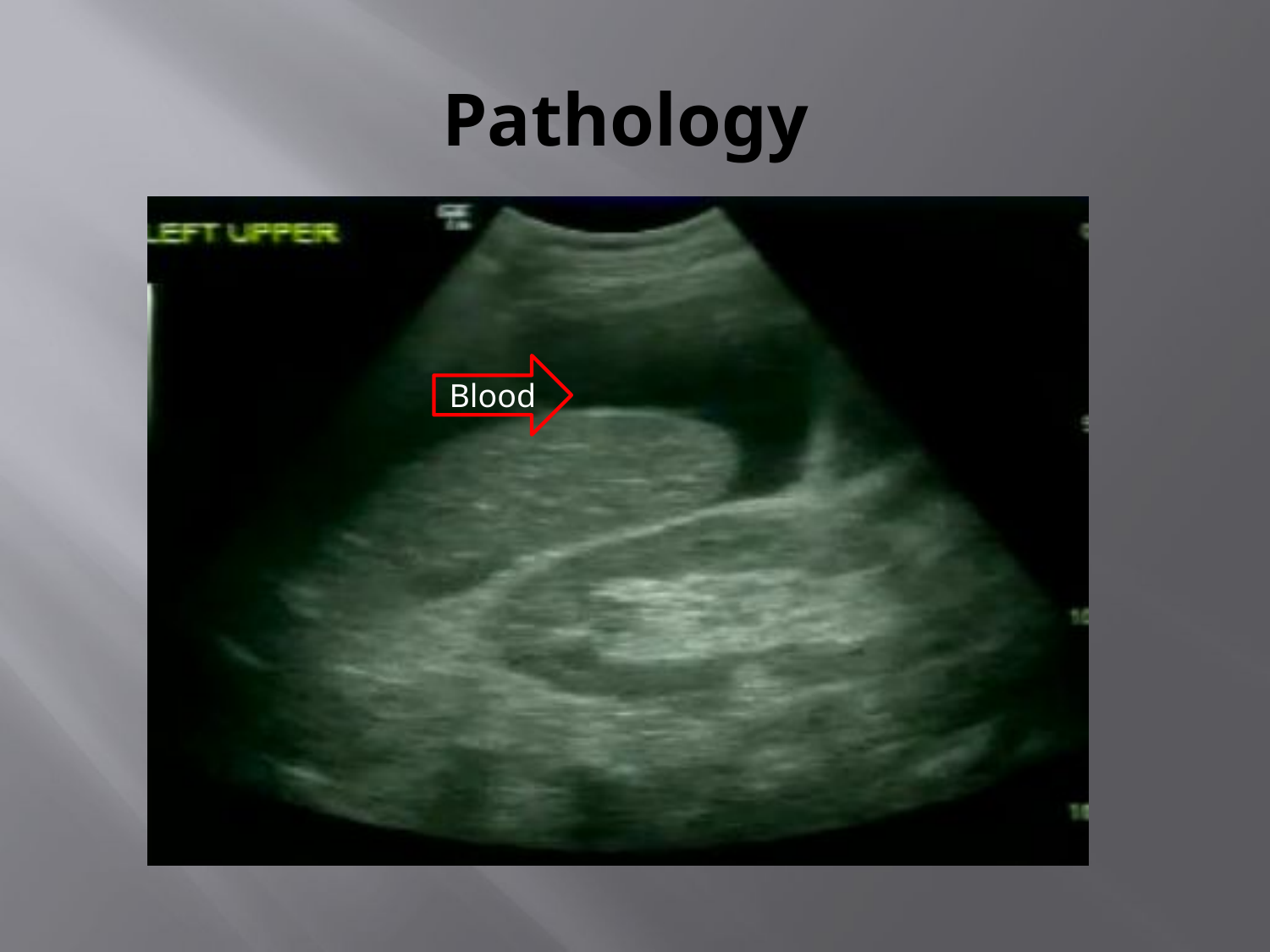

# Pathology
Blood

## Slide 20
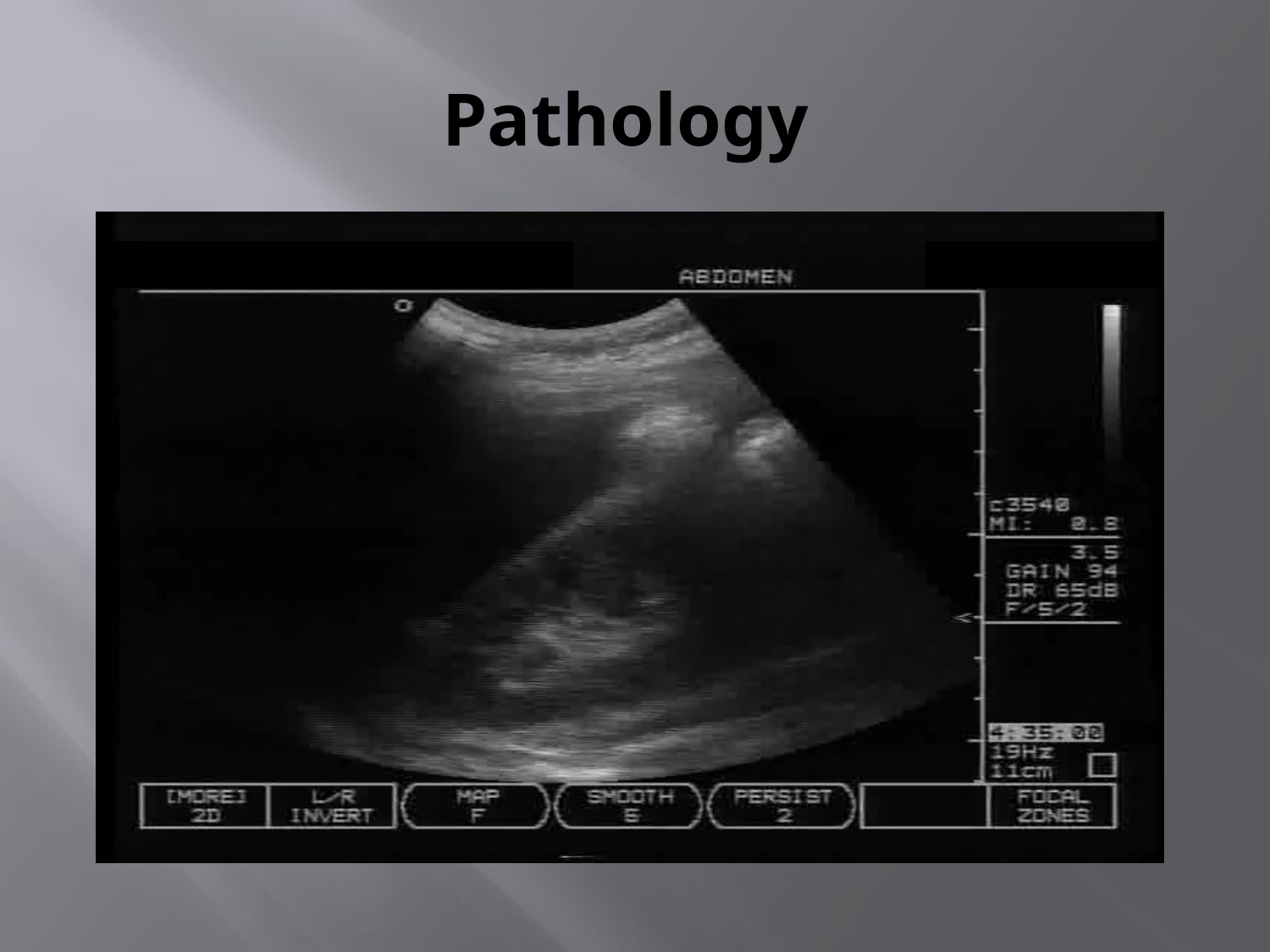

# Pathology

## Slide 21
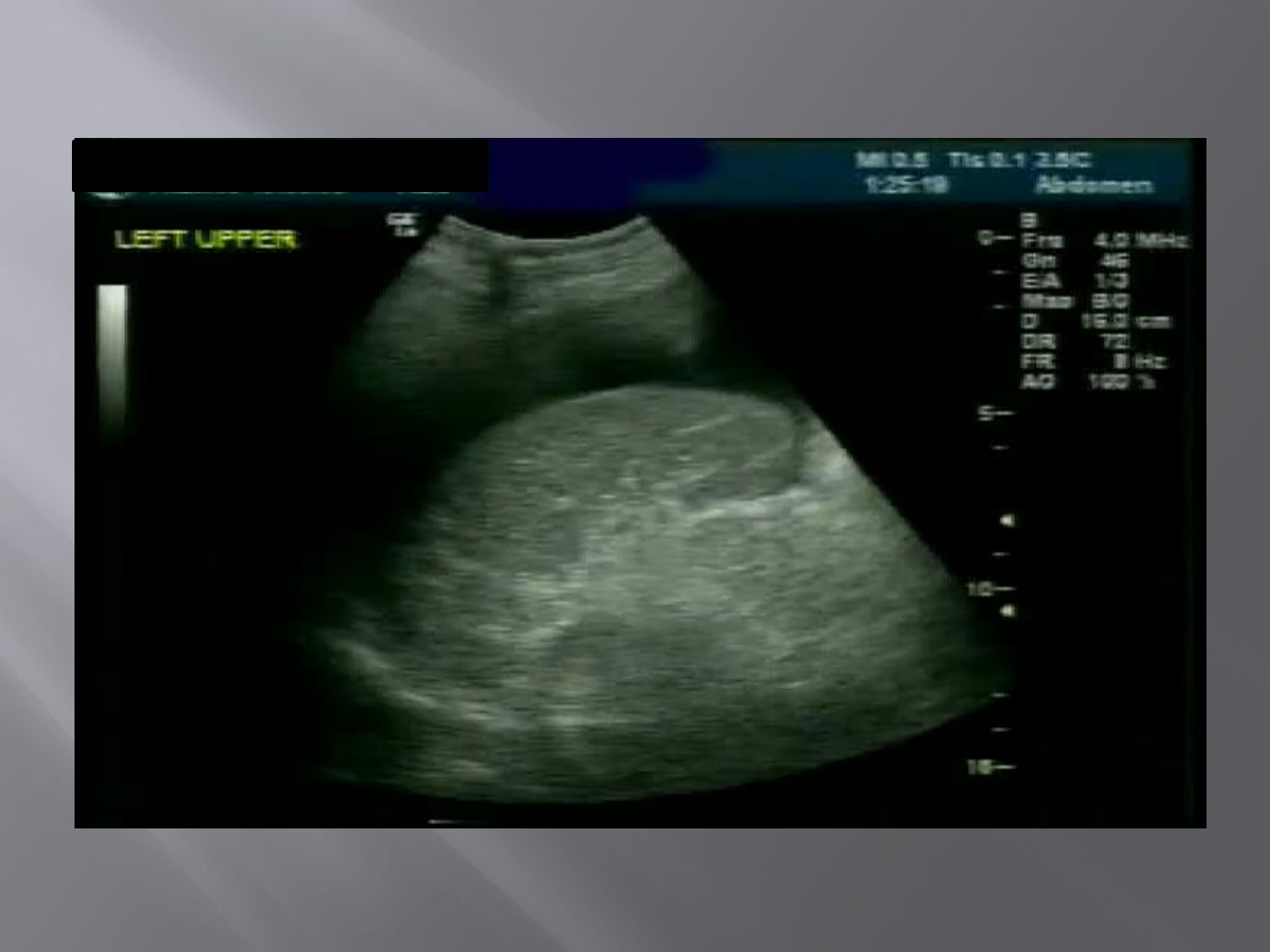

## Slide 22
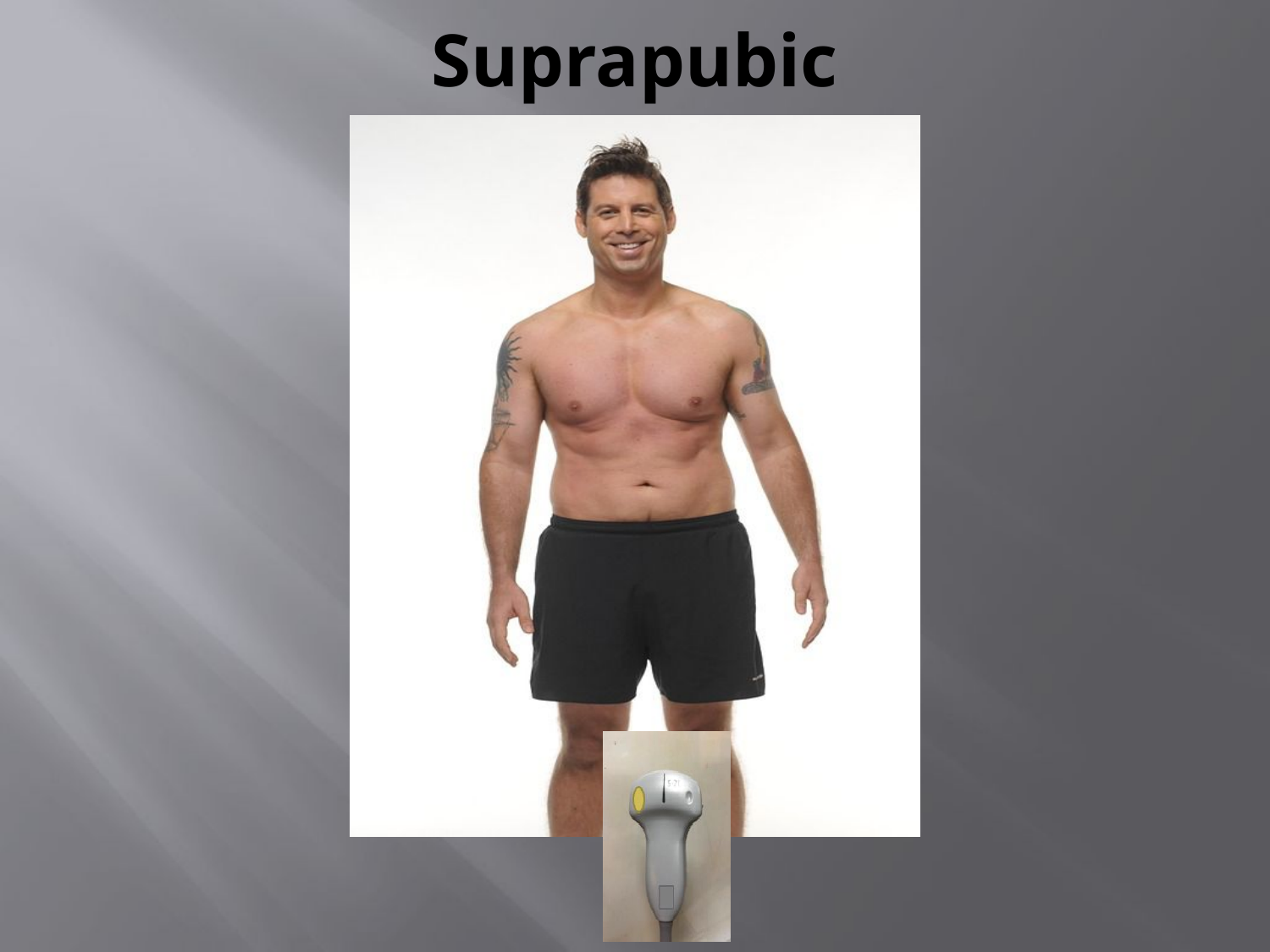

# Suprapubic

## Slide 23
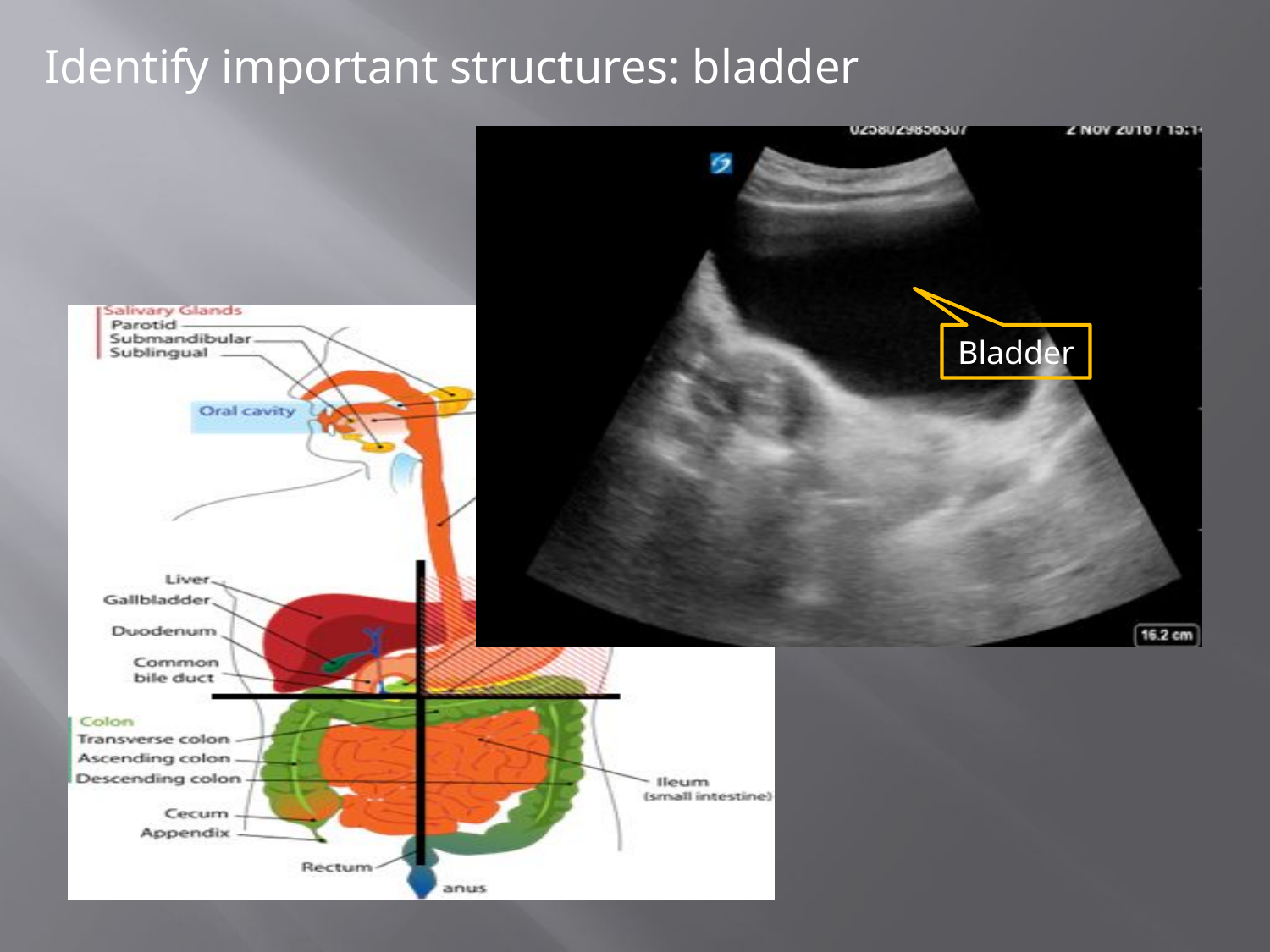

Identify important structures: bladder
Bladder

## Slide 24
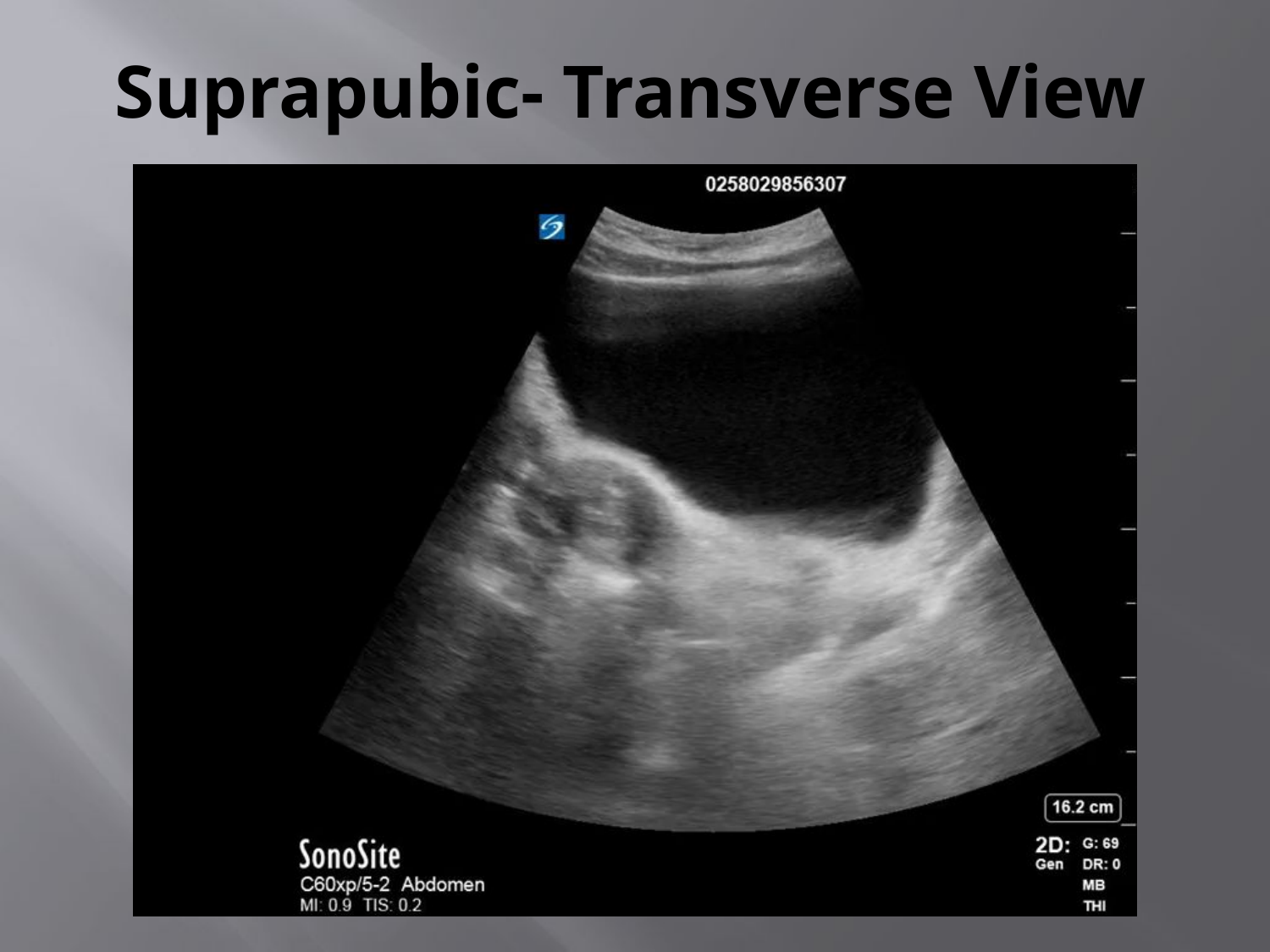

# Suprapubic- Transverse View

## Slide 25
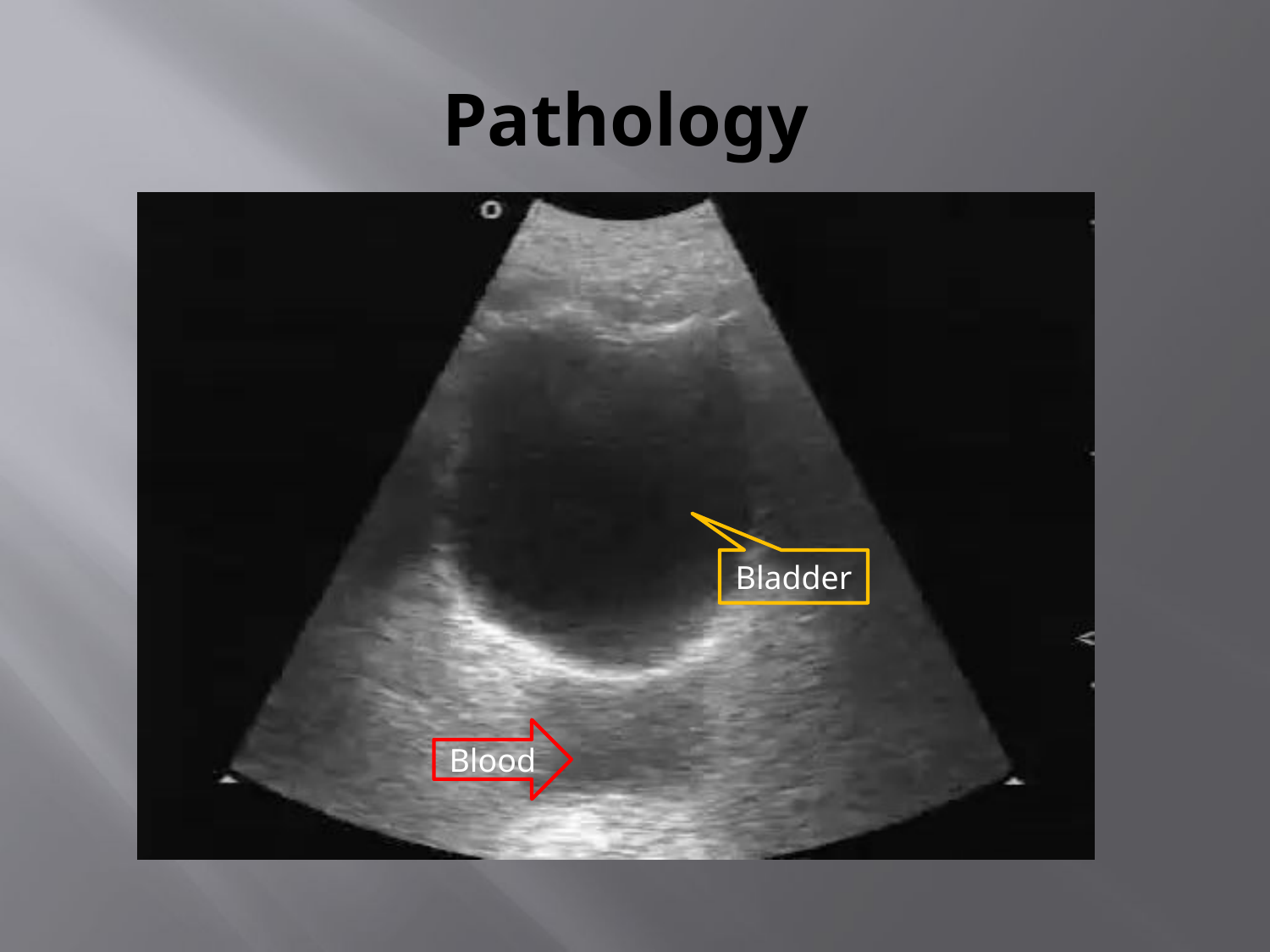

# Pathology
Bladder
Blood

## Slide 26
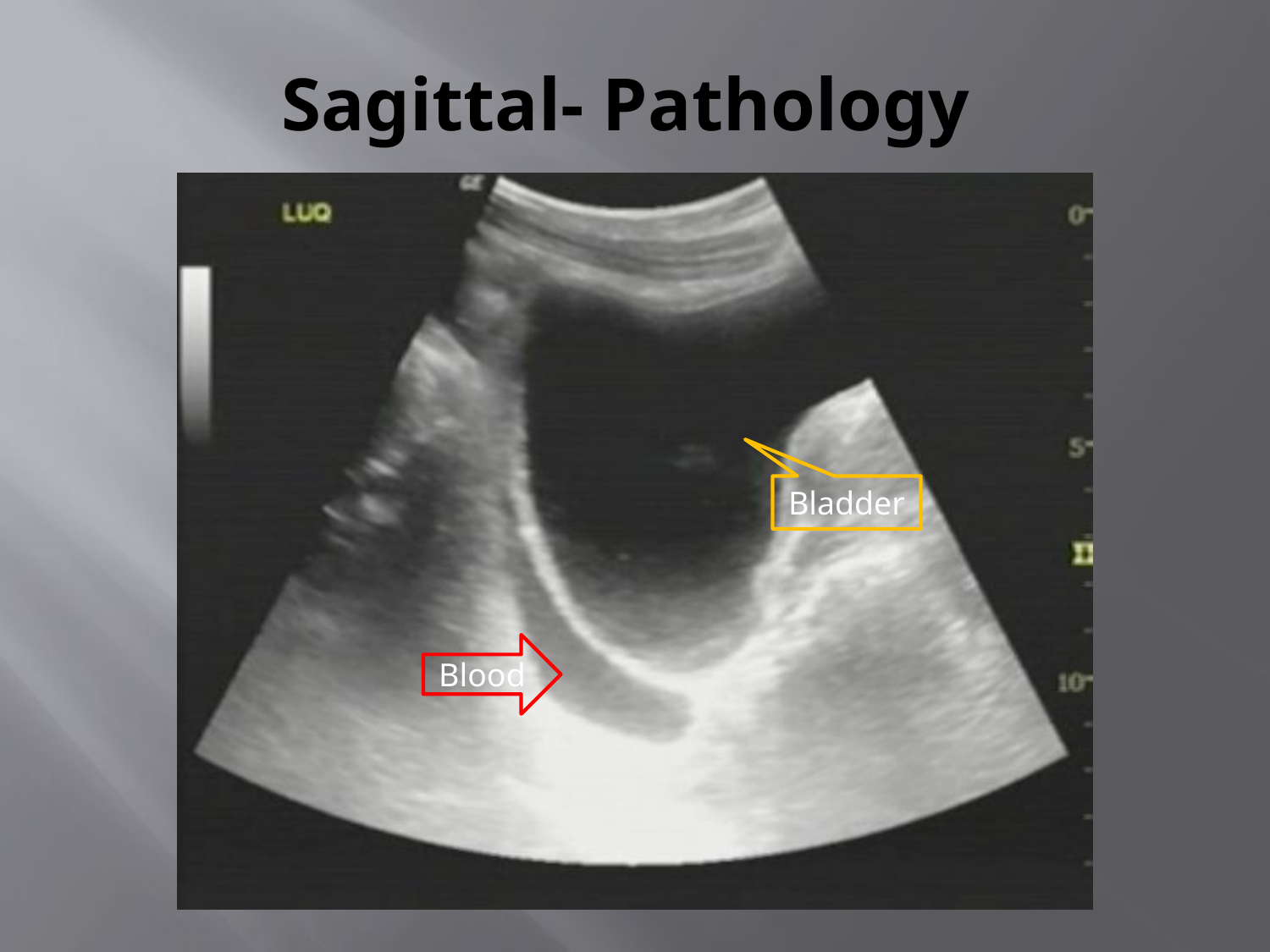

# Sagittal- Pathology
Bladder
Blood

## Slide 27
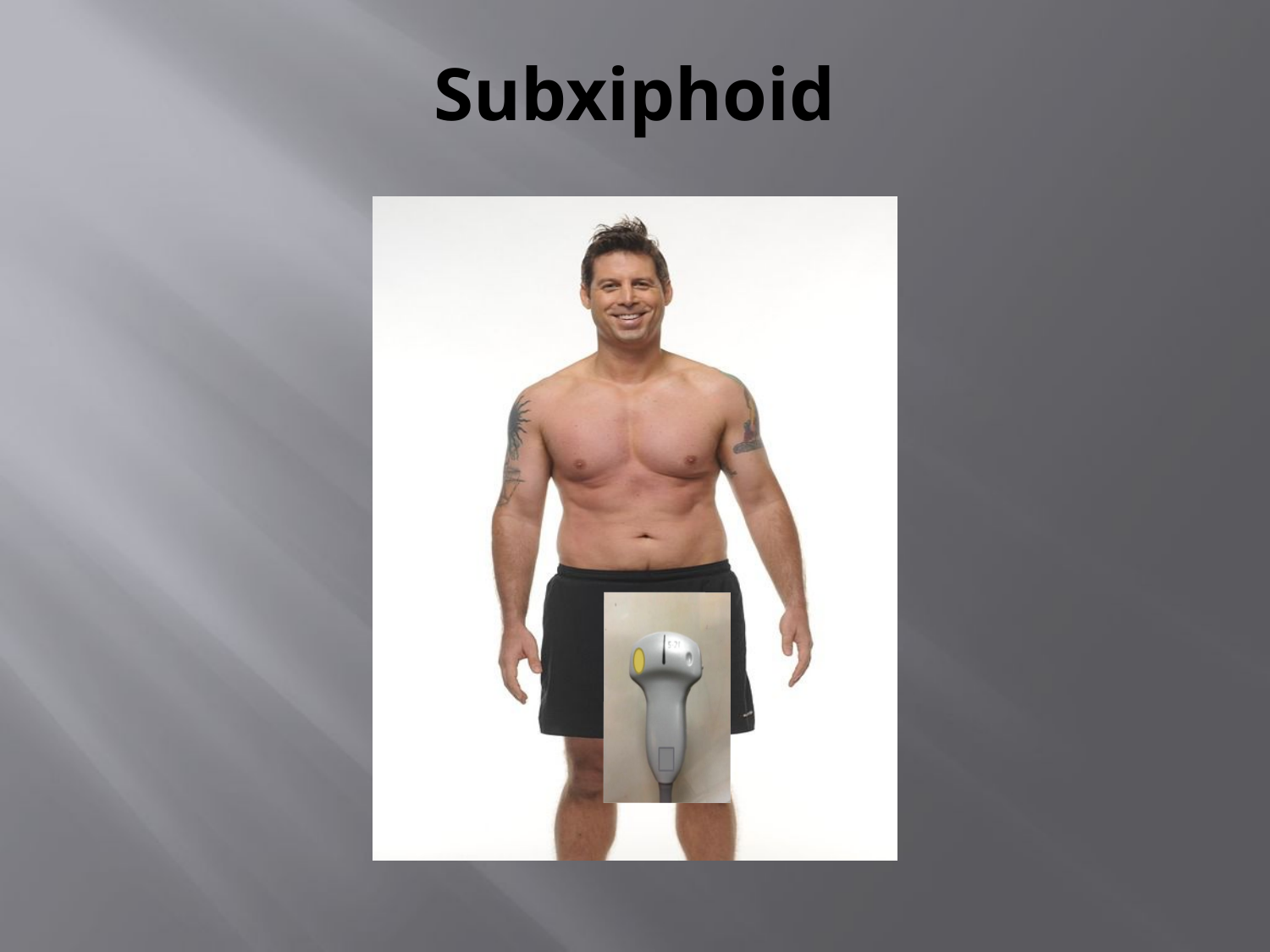

# Subxiphoid

## Slide 28
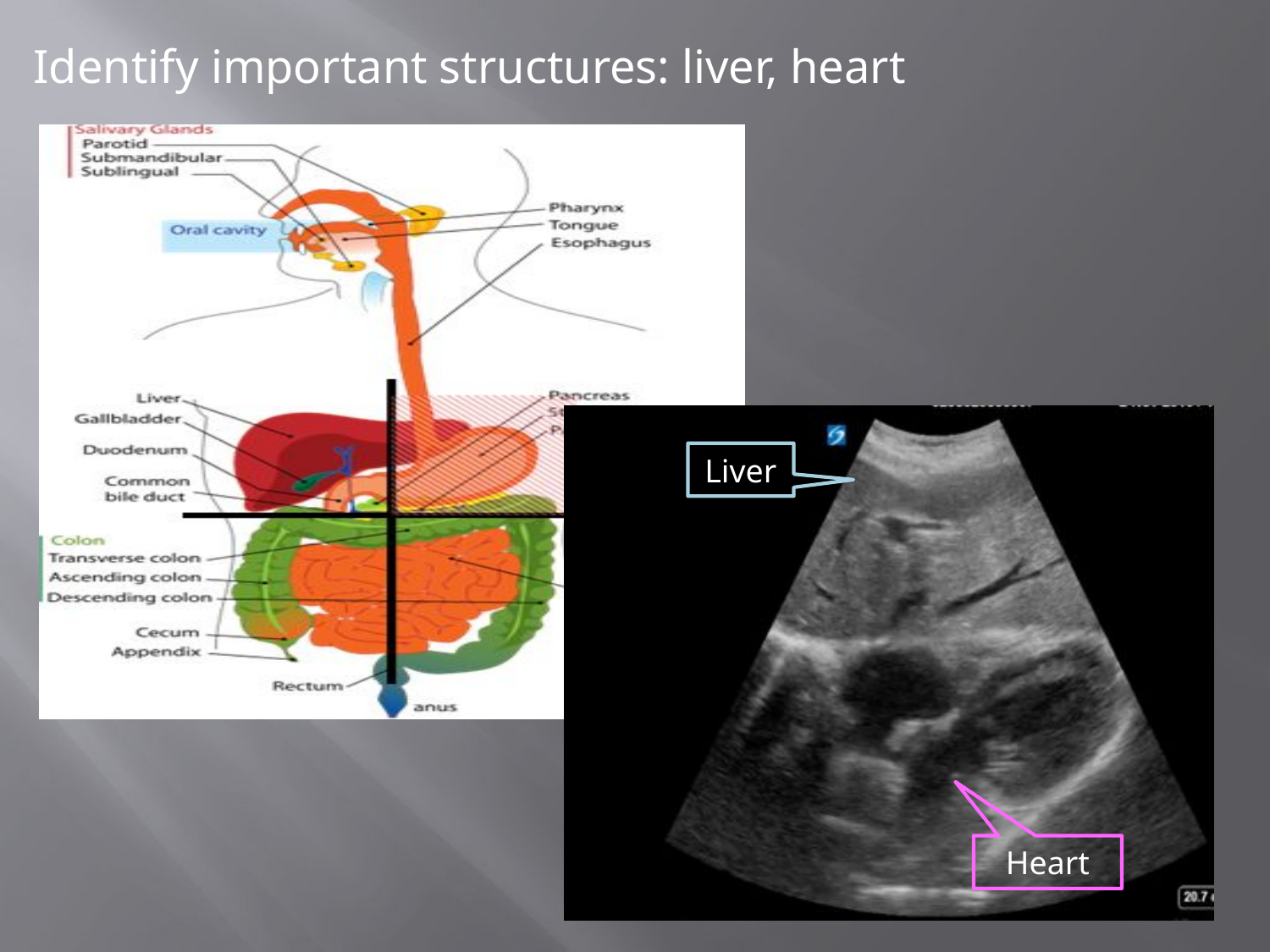

Identify important structures: liver, heart
Liver
Heart

## Slide 29
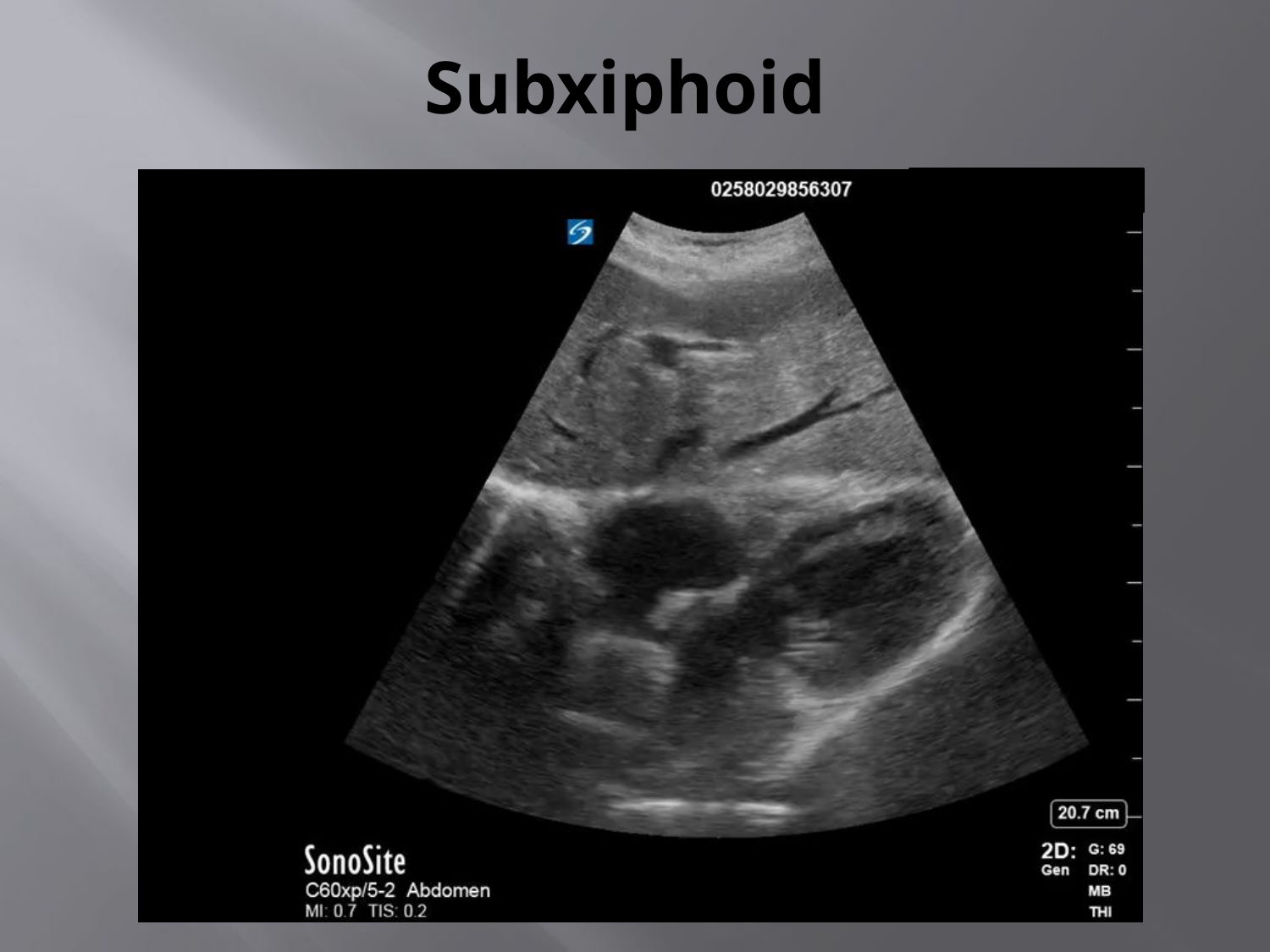

# Subxiphoid

## Slide 30
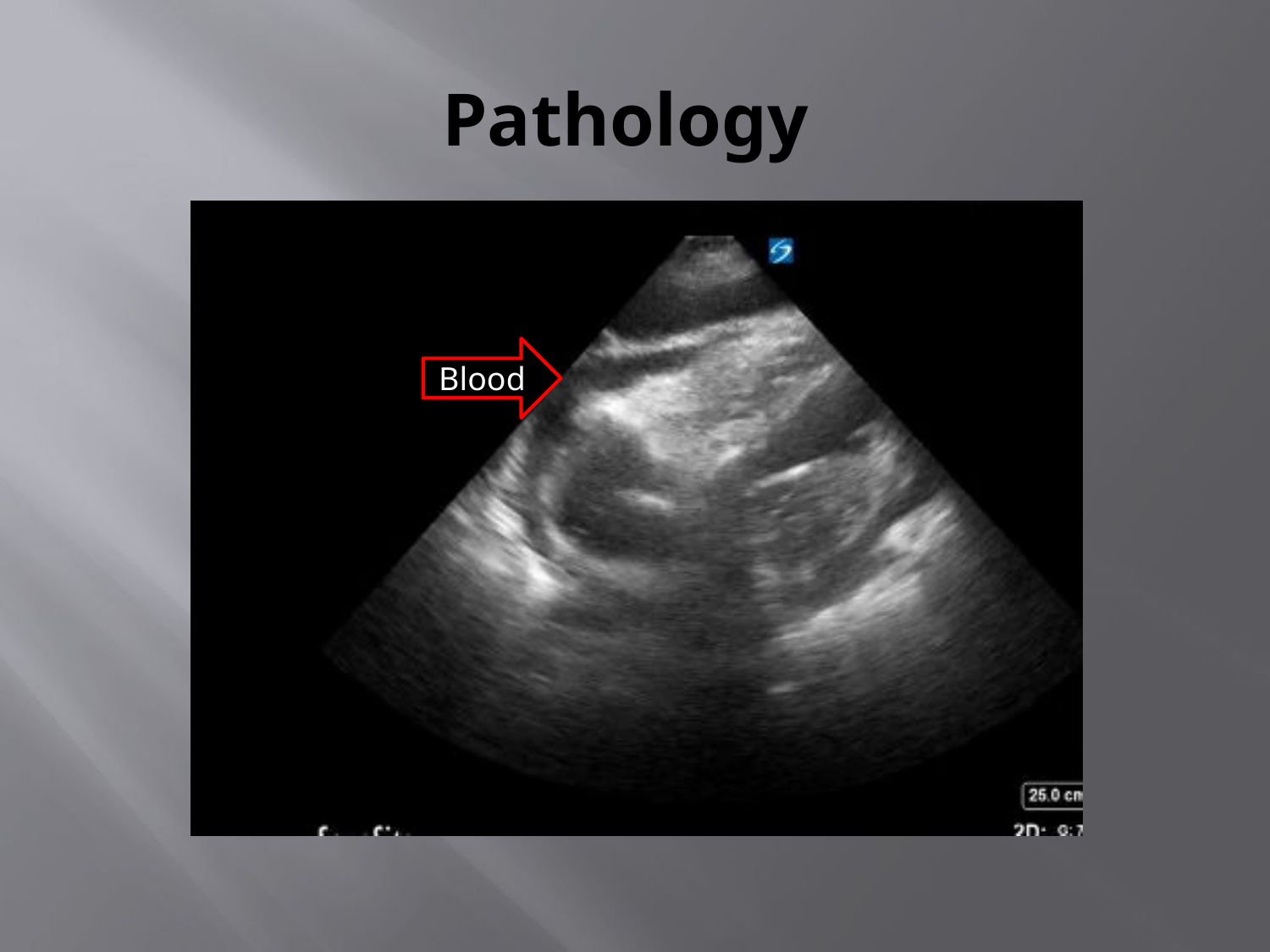

# Pathology
Blood

## Slide 31
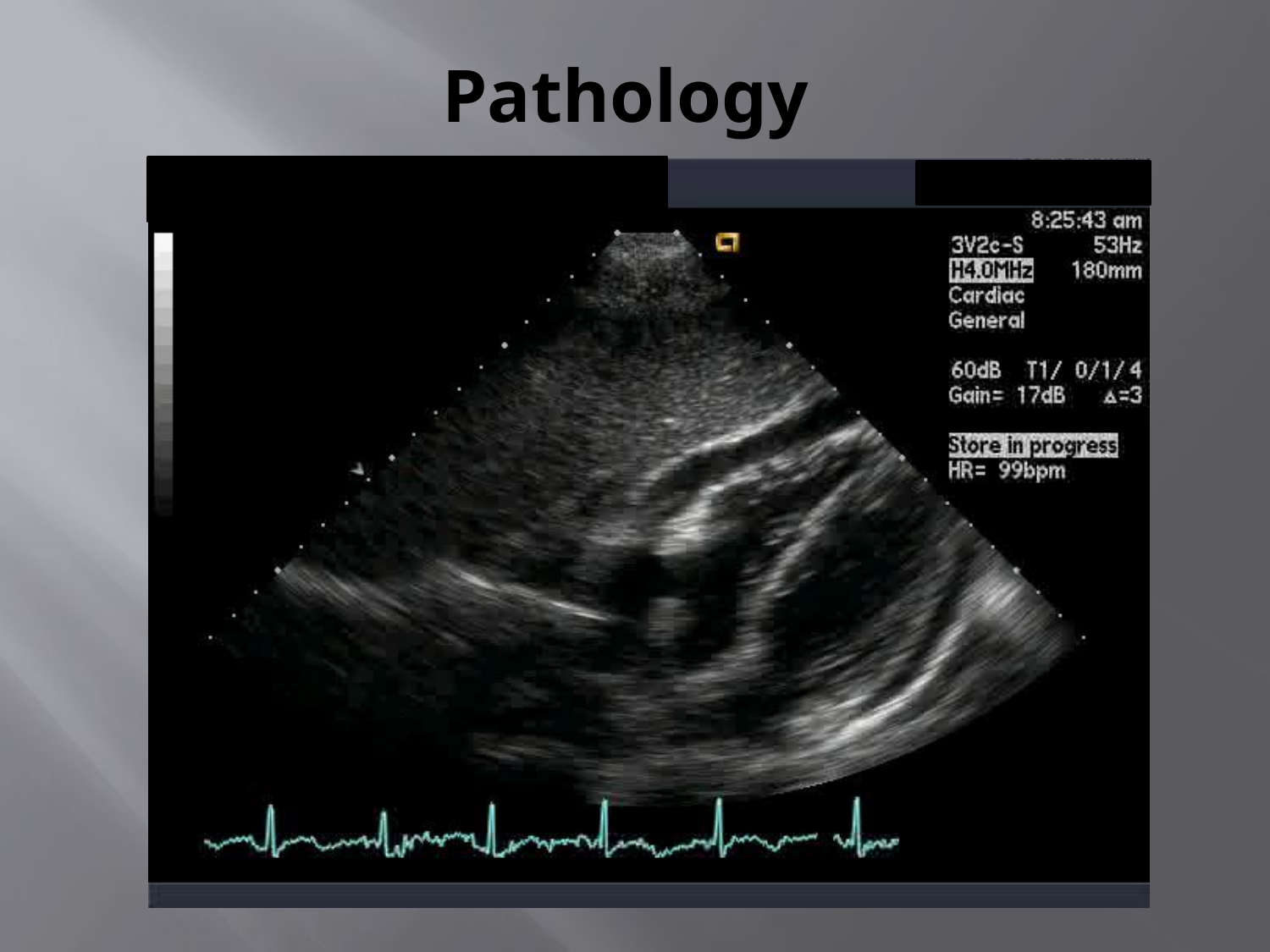

# Pathology

## Slide 32
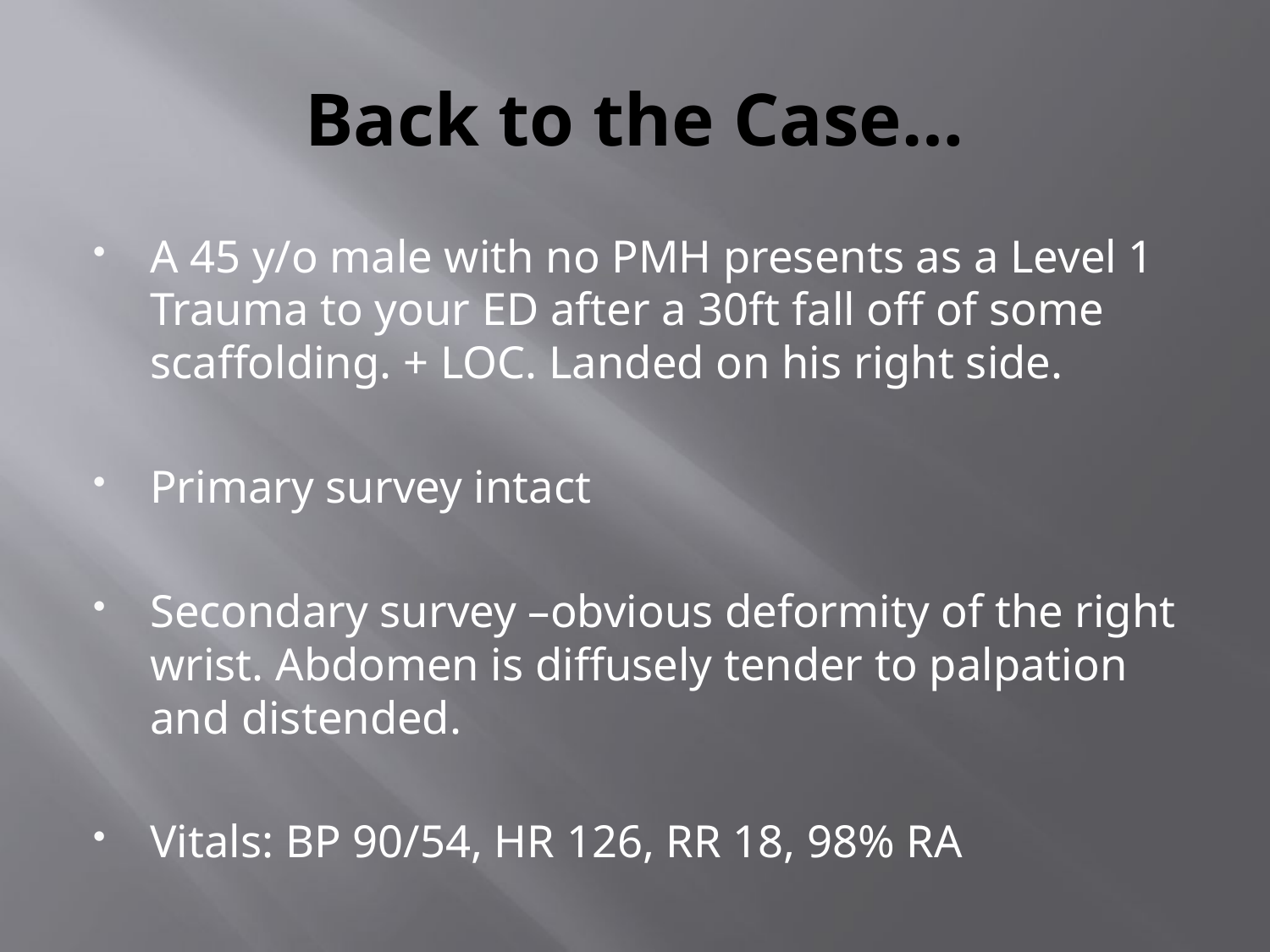

# Back to the Case…
A 45 y/o male with no PMH presents as a Level 1 Trauma to your ED after a 30ft fall off of some scaffolding. + LOC. Landed on his right side.
Primary survey intact
Secondary survey –obvious deformity of the right wrist. Abdomen is diffusely tender to palpation and distended.
Vitals: BP 90/54, HR 126, RR 18, 98% RA

## Slide 33
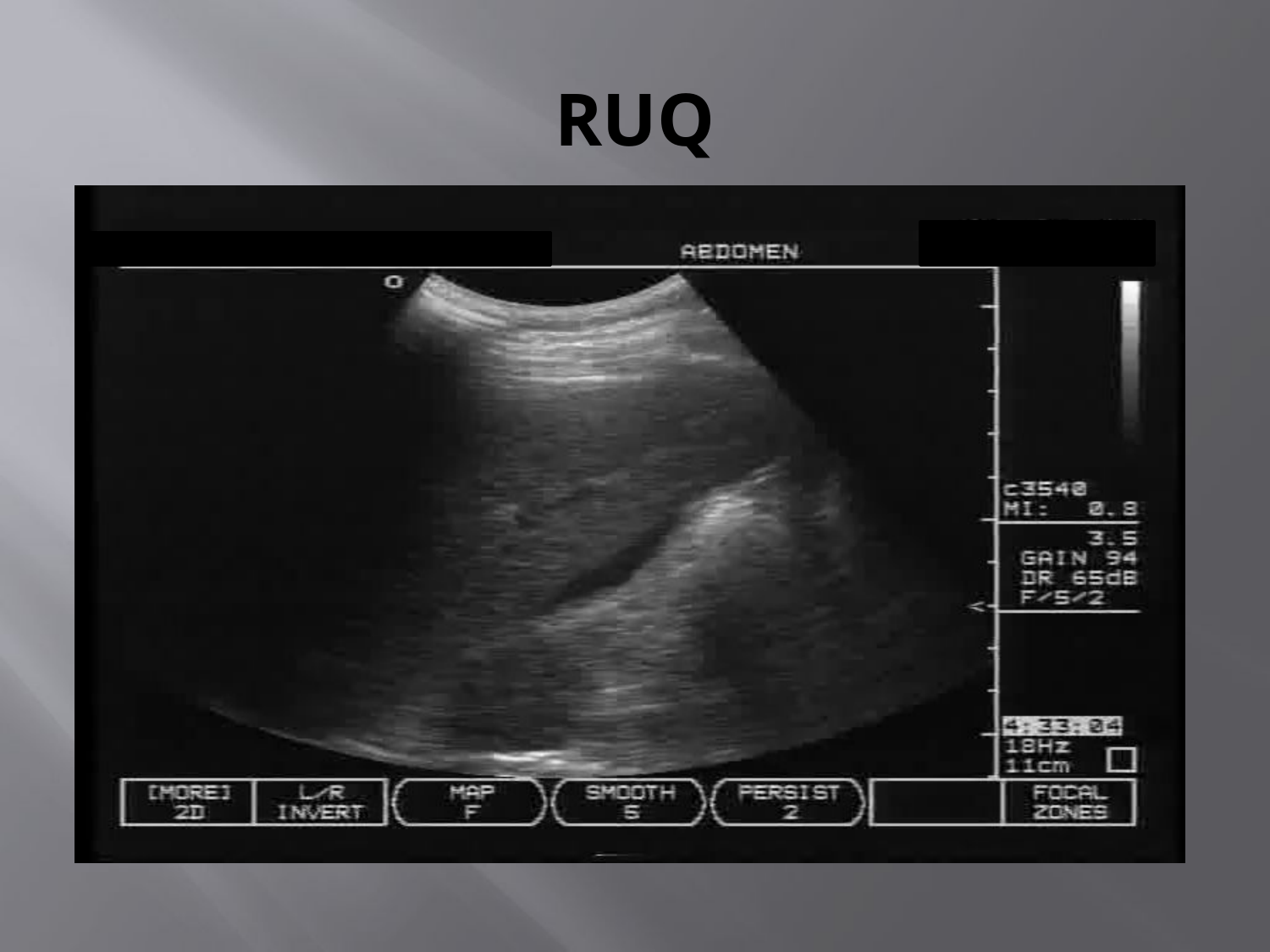

# RUQ

## Slide 34
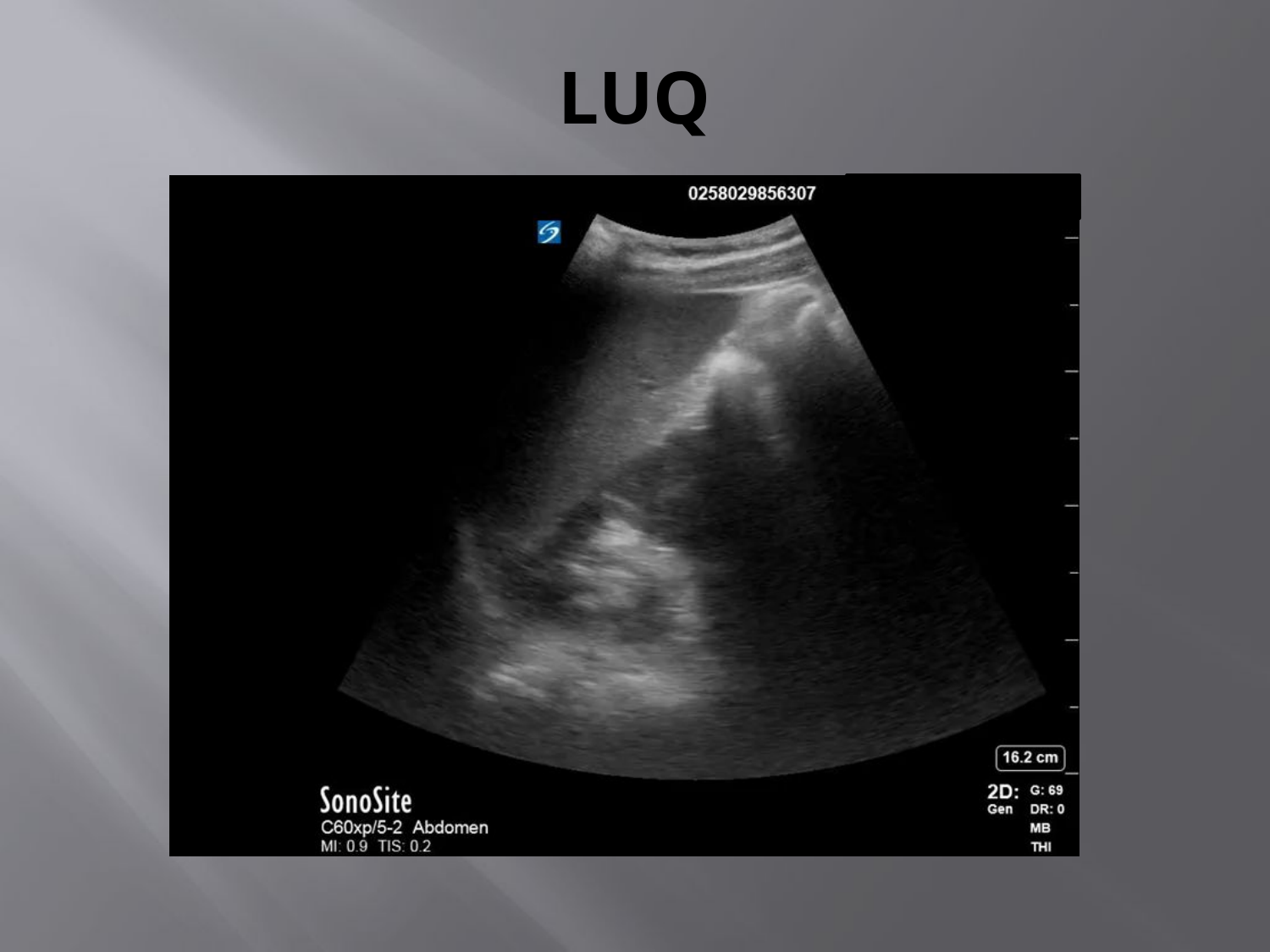

# LUQ

## Slide 35
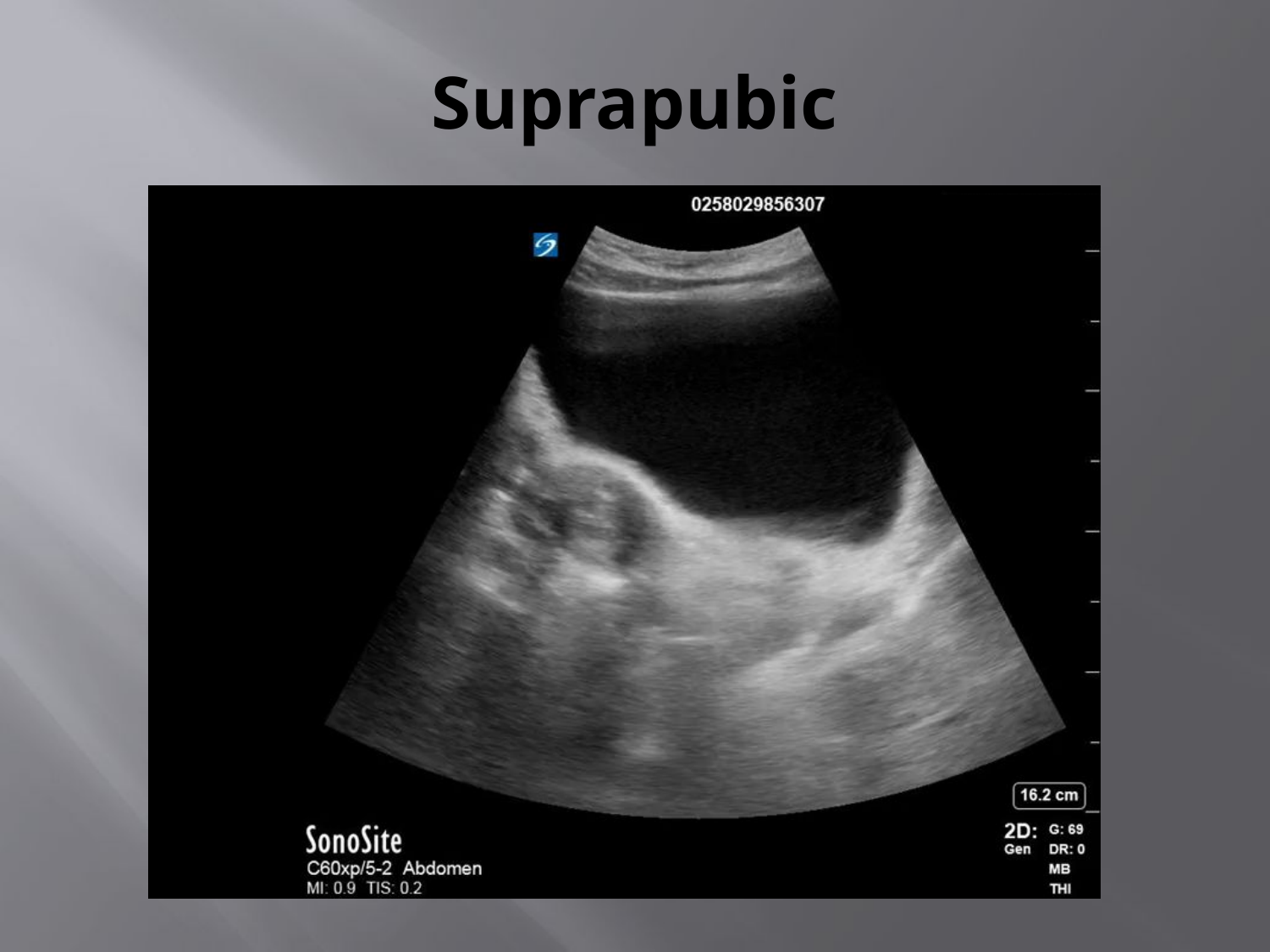

# Suprapubic

## Slide 36
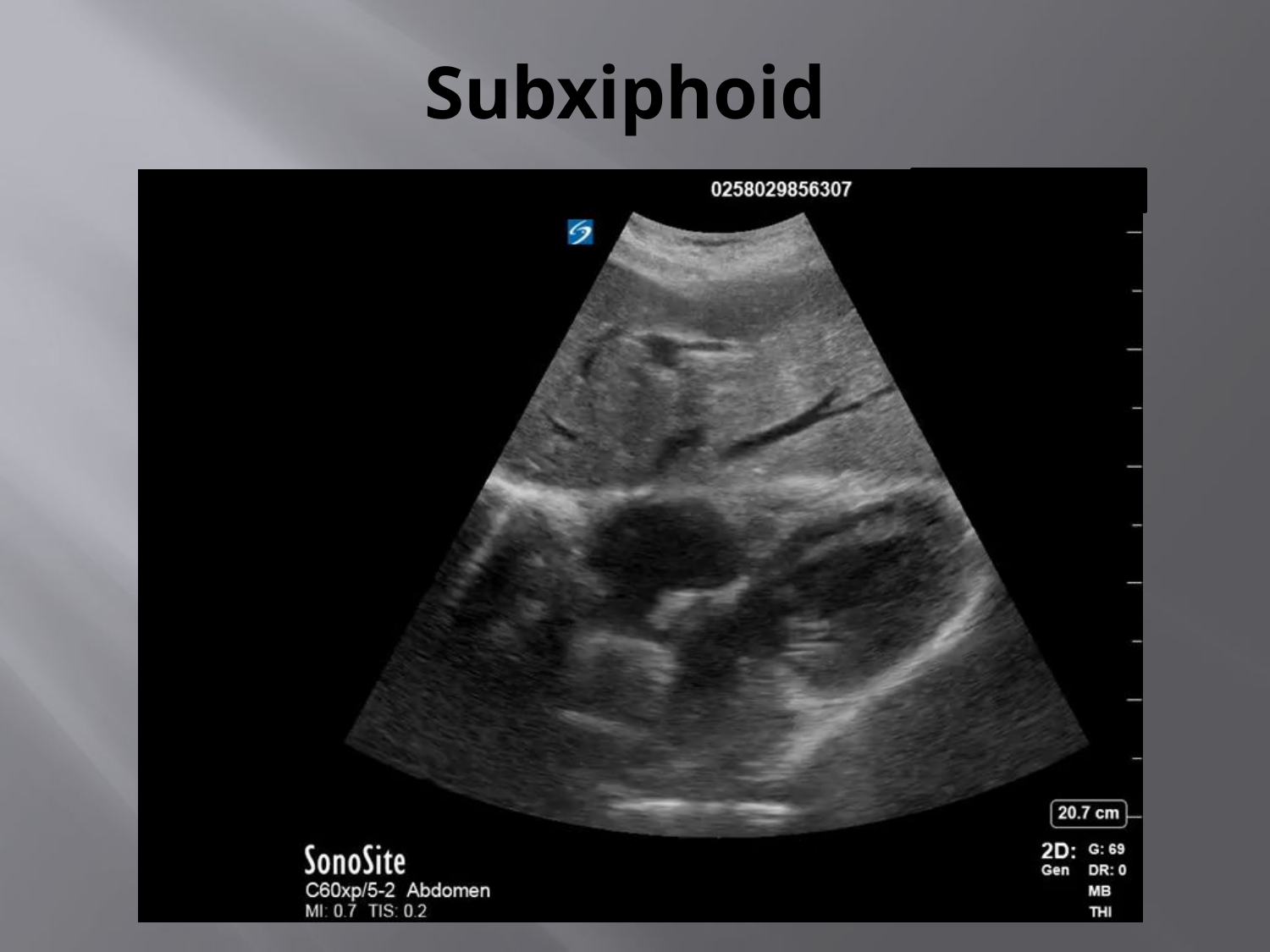

# Subxiphoid

## Slide 37
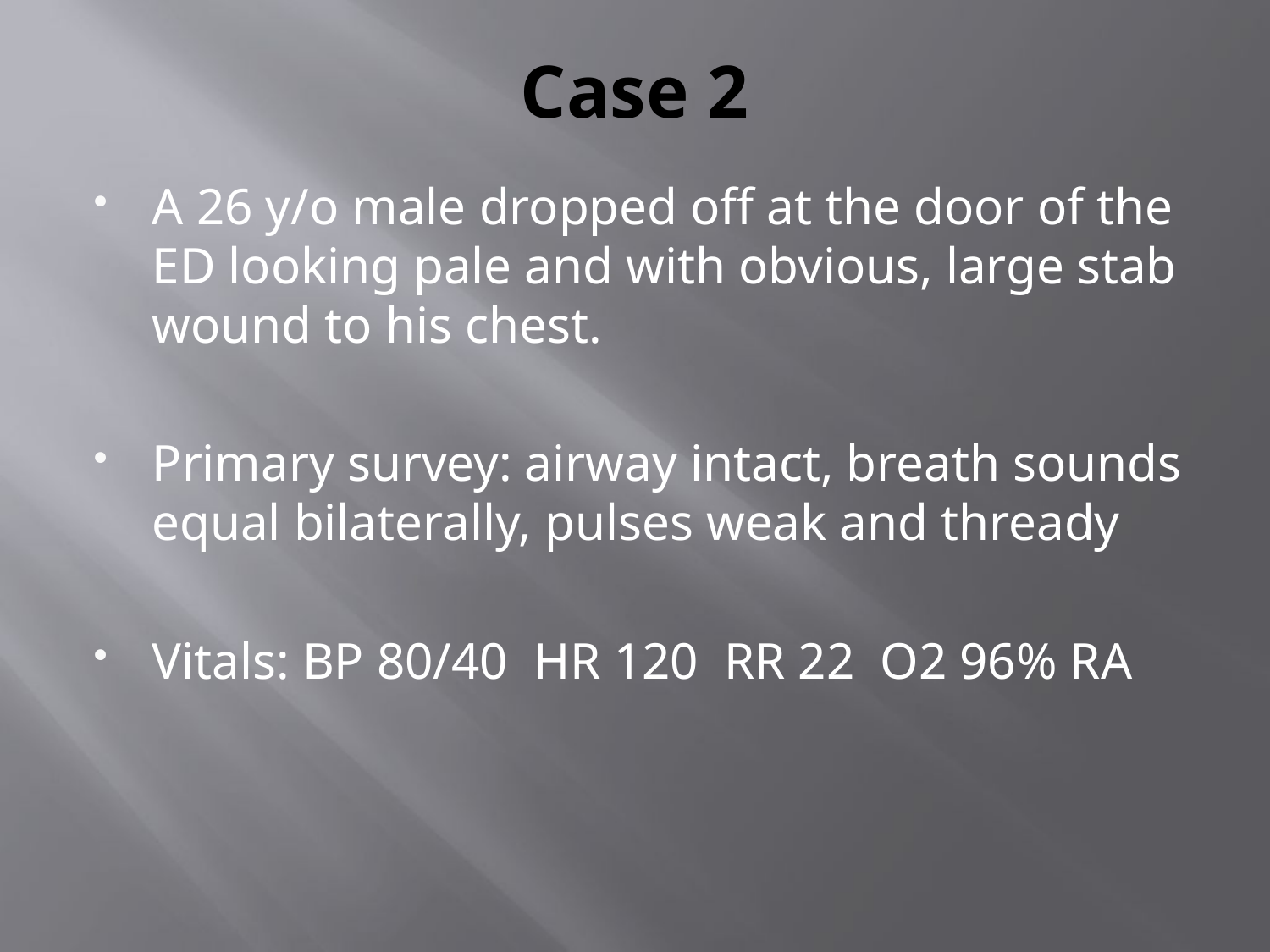

# Case 2
A 26 y/o male dropped off at the door of the ED looking pale and with obvious, large stab wound to his chest.
Primary survey: airway intact, breath sounds equal bilaterally, pulses weak and thready
Vitals: BP 80/40 HR 120 RR 22 O2 96% RA

## Slide 38
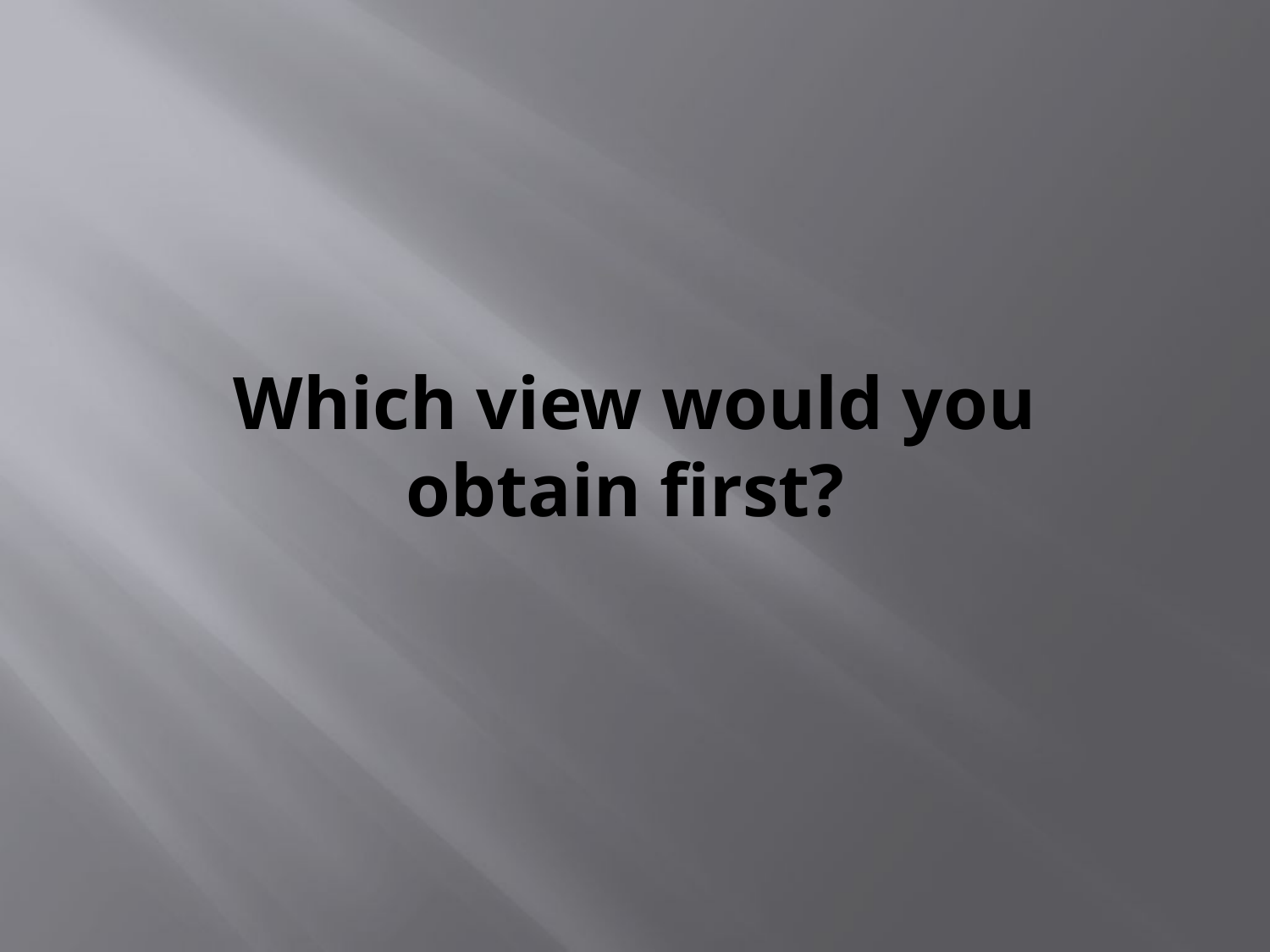

# Which view would you obtain first?

## Slide 39
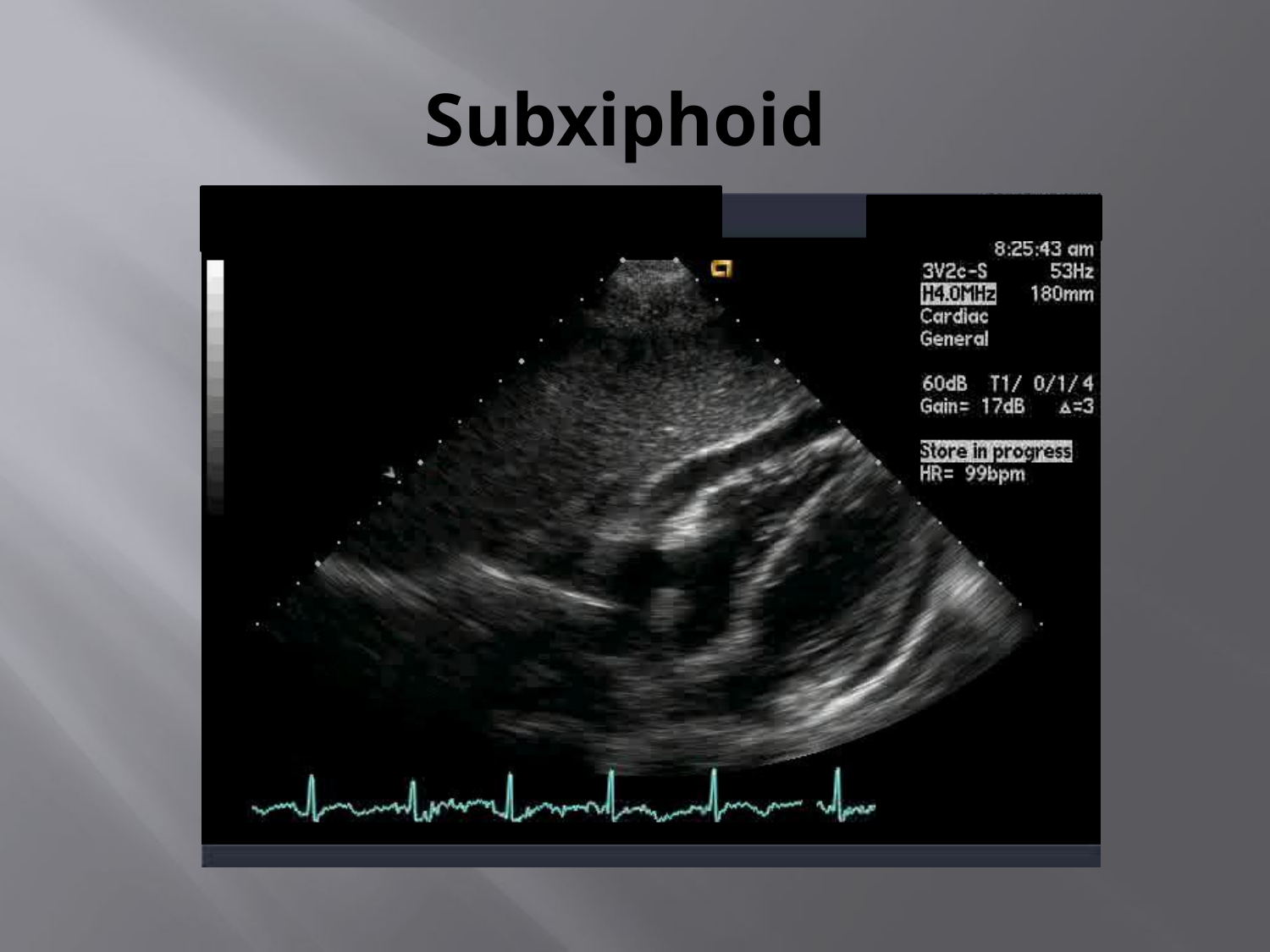

# Subxiphoid

## Slide 40
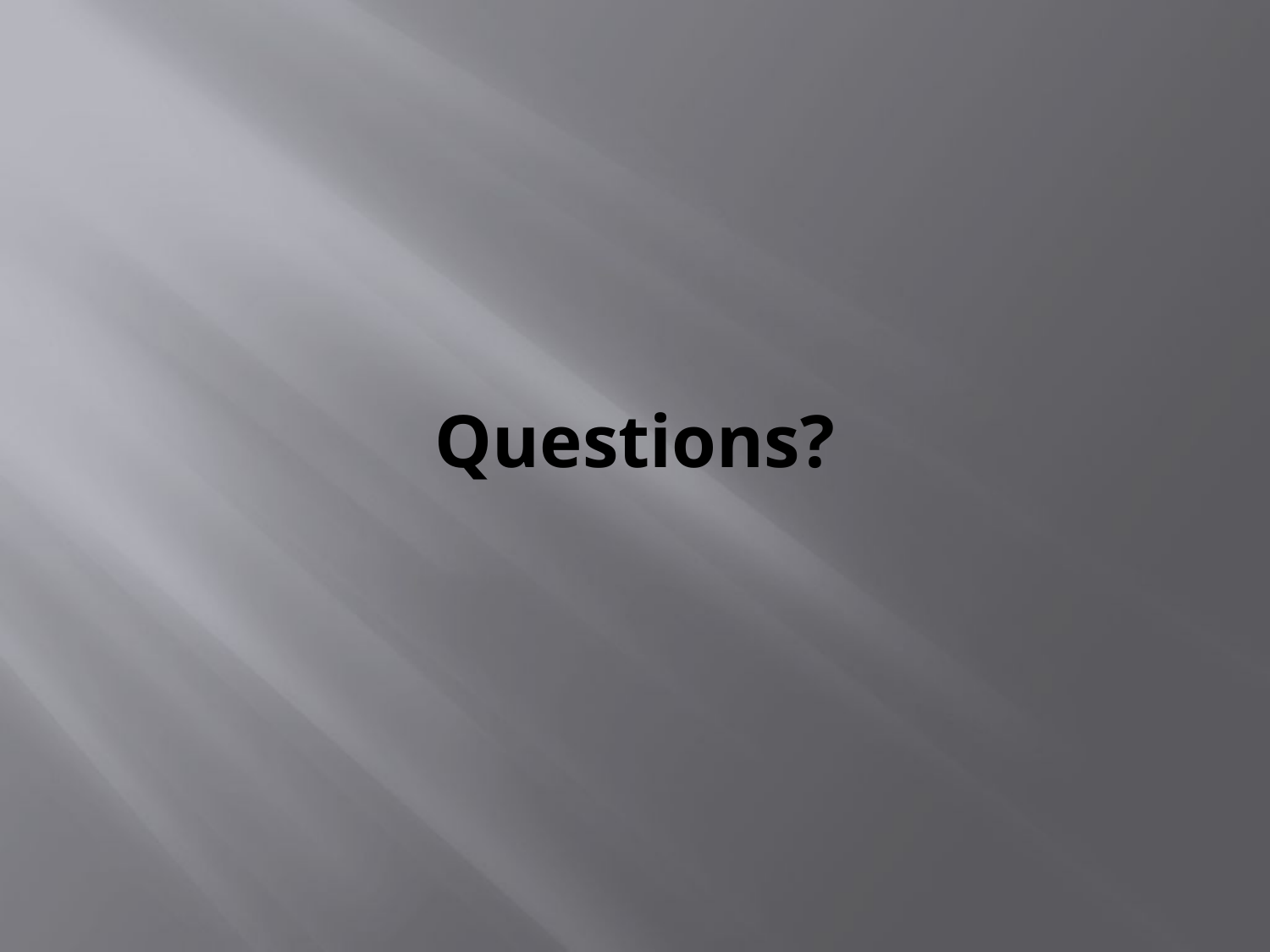

# Questions?

## Slide 41
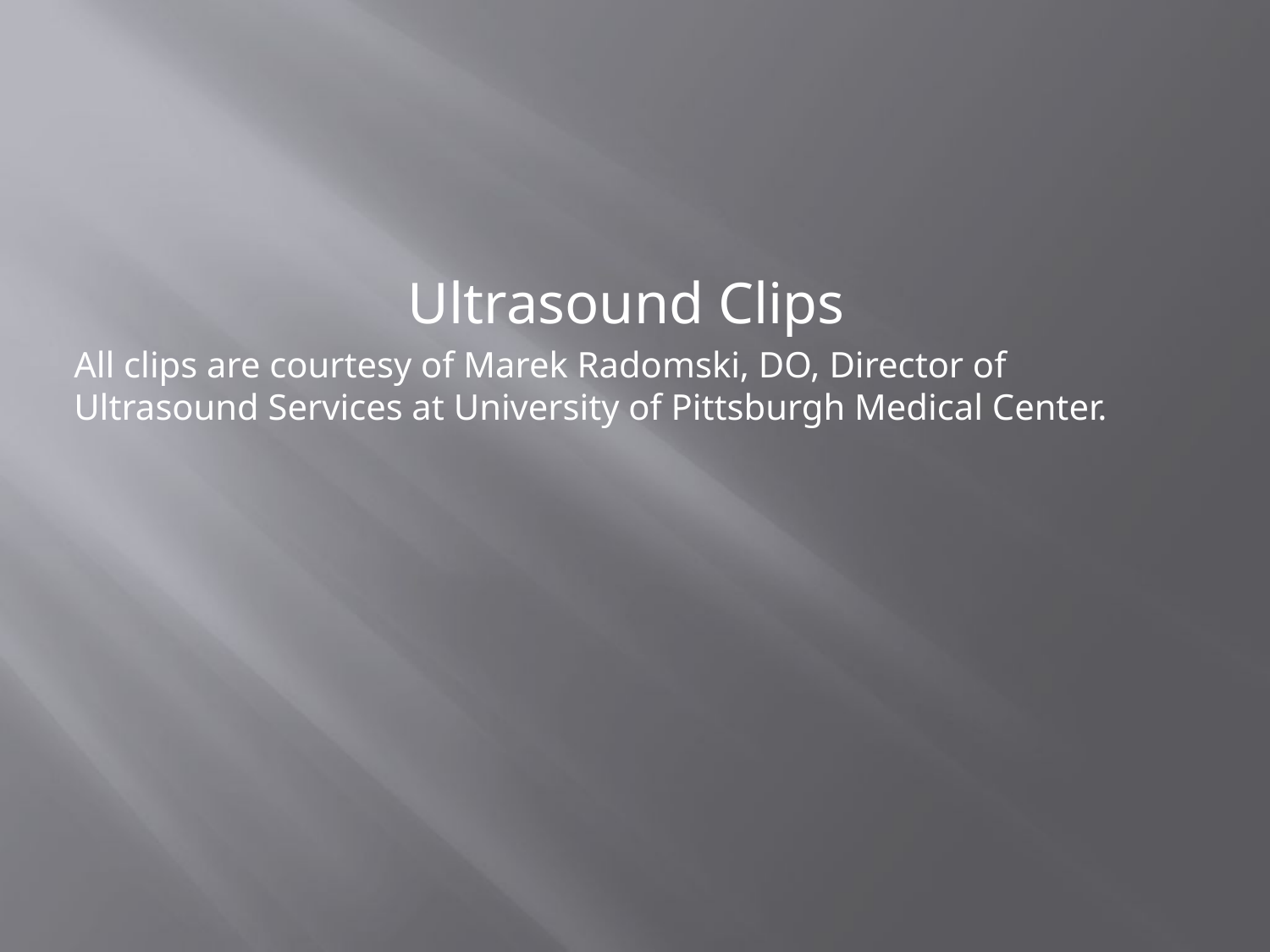

#
Ultrasound Clips
All clips are courtesy of Marek Radomski, DO, Director of Ultrasound Services at University of Pittsburgh Medical Center.
