# Supplementary material for: Use of Ultrasound in Introducing Anatomical Pathology to Preclinical Medical Students, in Correlation with Physical Exam Curricula
Source: MedEdPORTAL. 2020 Sep 25;16:10950. doi: 10.15766/mep_2374-8265.10950 (PMC7521063; doi:10.15766/mep_2374-8265.10950)
Supplement: Supplementary file 1 — Session 1 FAST Exam & the Trauma Patient.pptxSession 2 Cardiac and Lung.pptxSession 3 Gallbladder, Kidneys, & AAA.pptxSession 4 Ocular US & Central Access.pptxSession 1 Instructor Script.docxSession 2 Instructor Script.docxSession 3 Instructor Script.docxSession 4 Instructor Script.docxSurvey Questions.docx [file mep_2374-8265.10950-s001.zip › B. Session 2 Cardiac and Lung.pptx]

## Slide 1
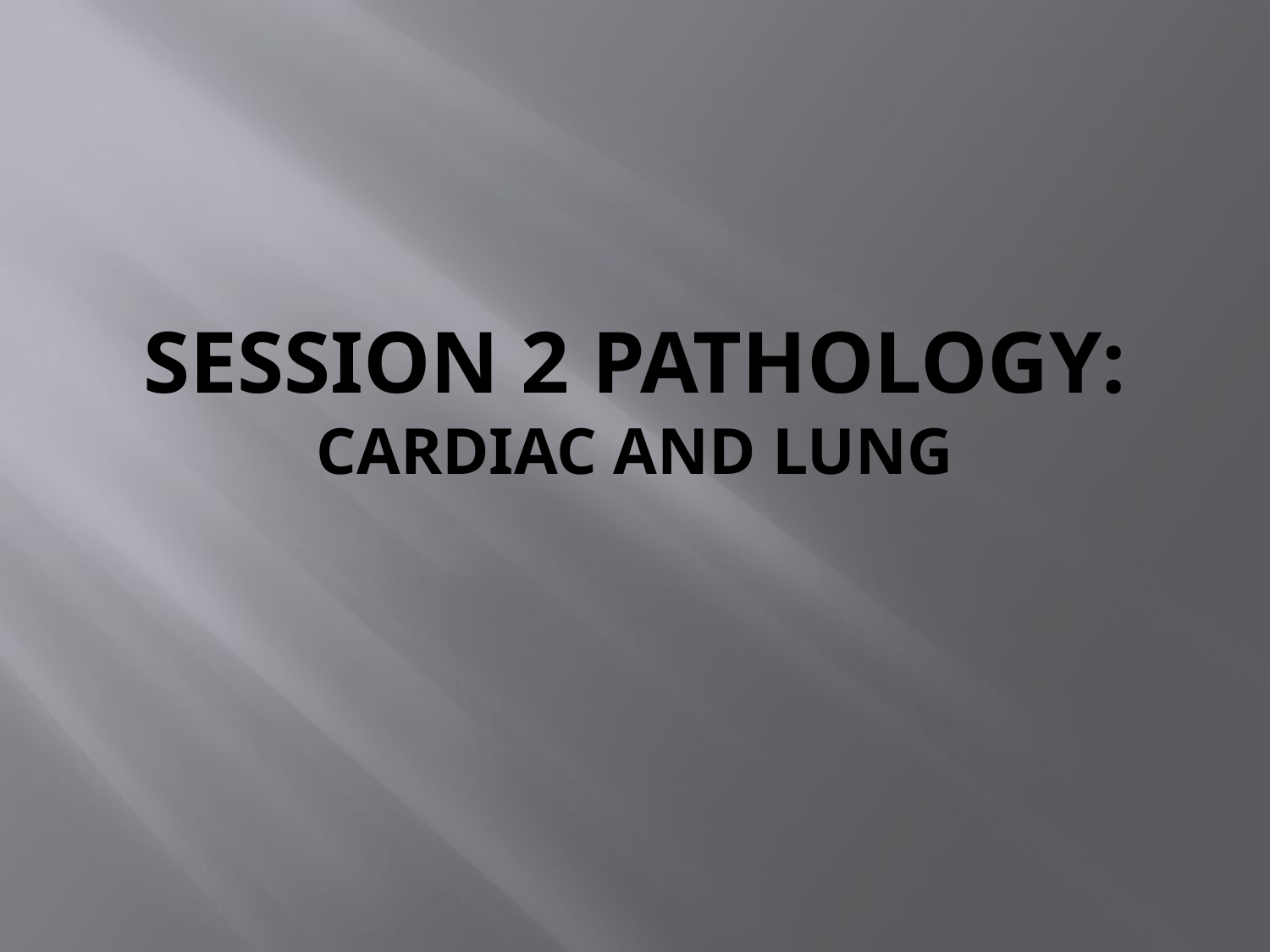

# Session 2 Pathology: Cardiac and Lung

## Slide 2
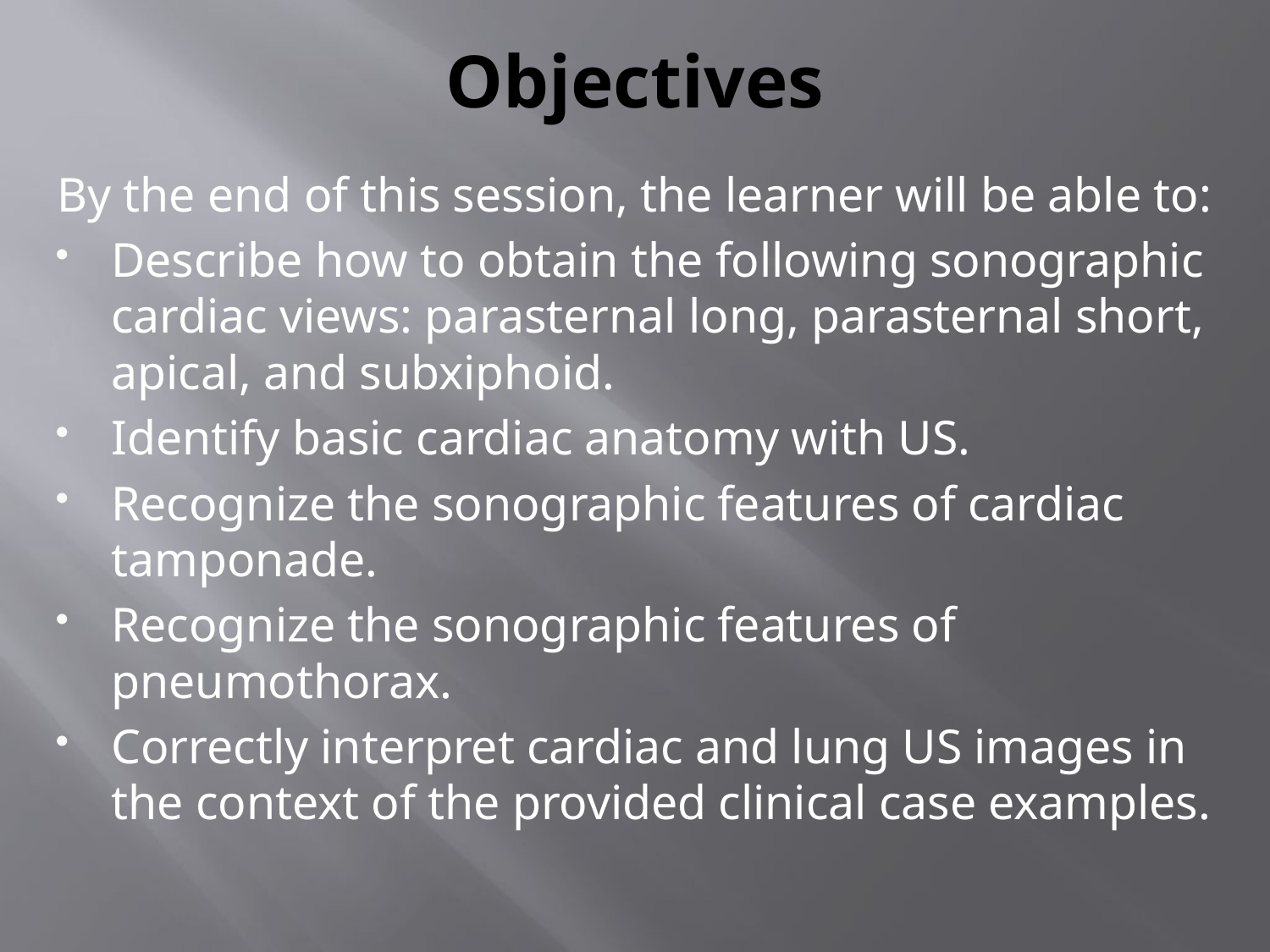

# Objectives
By the end of this session, the learner will be able to:
Describe how to obtain the following sonographic cardiac views: parasternal long, parasternal short, apical, and subxiphoid.
Identify basic cardiac anatomy with US.
Recognize the sonographic features of cardiac tamponade.
Recognize the sonographic features of pneumothorax.
Correctly interpret cardiac and lung US images in the context of the provided clinical case examples.

## Slide 3
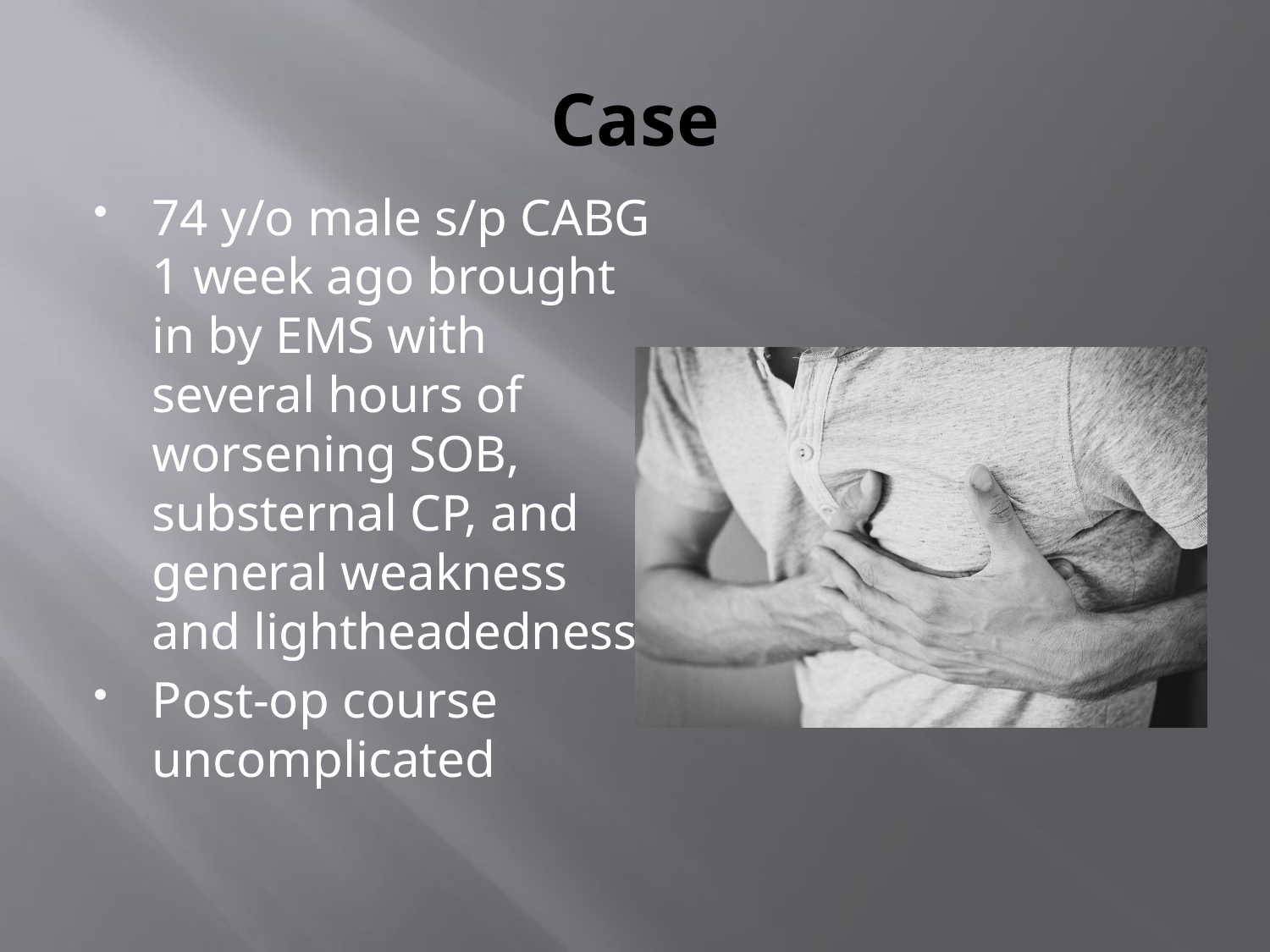

# Case
74 y/o male s/p CABG 1 week ago brought in by EMS with several hours of worsening SOB, substernal CP, and general weakness and lightheadedness.
Post-op course uncomplicated

## Slide 4
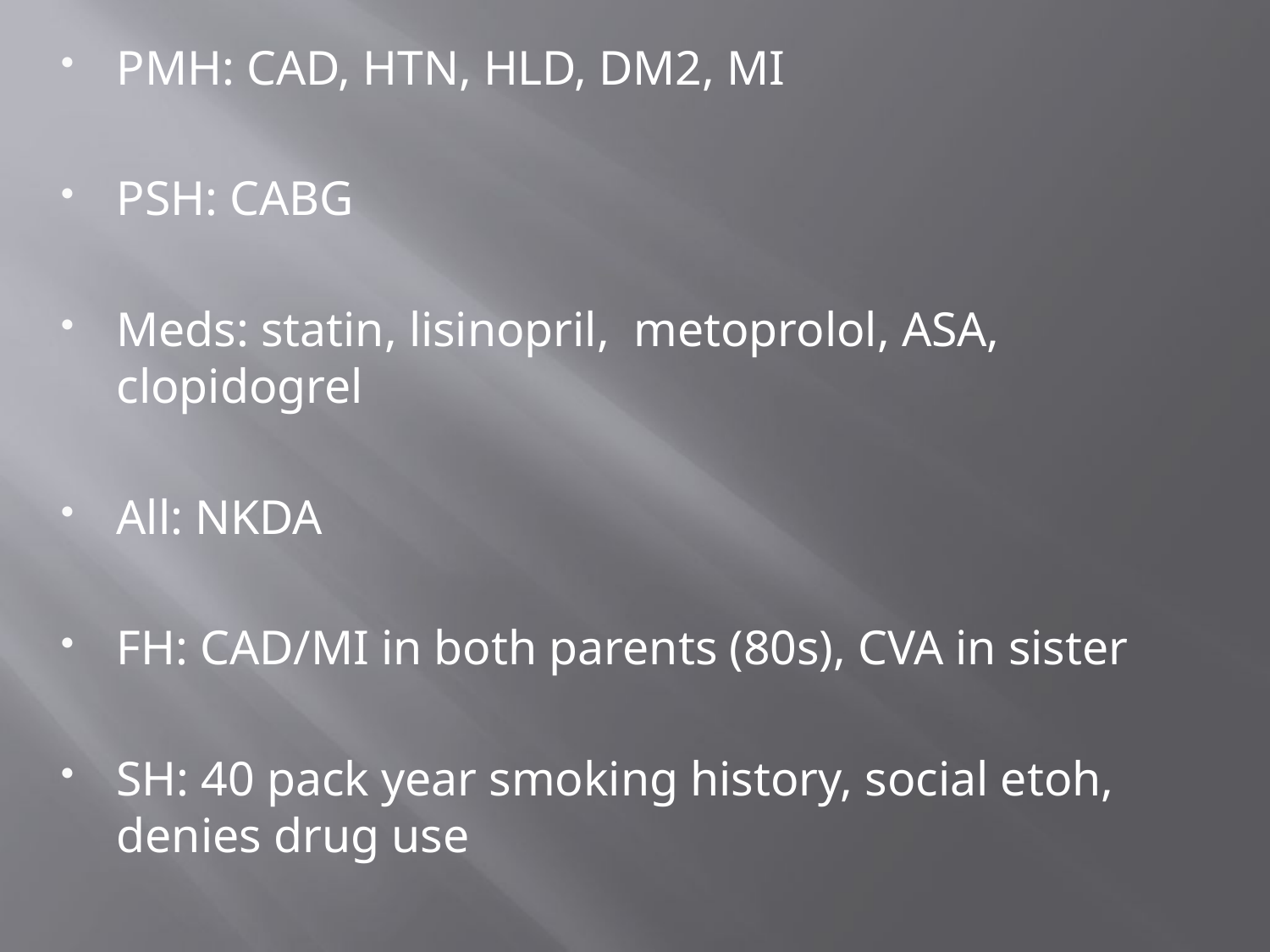

PMH: CAD, HTN, HLD, DM2, MI
PSH: CABG
Meds: statin, lisinopril, metoprolol, ASA, clopidogrel
All: NKDA
FH: CAD/MI in both parents (80s), CVA in sister
SH: 40 pack year smoking history, social etoh, denies drug use

## Slide 5
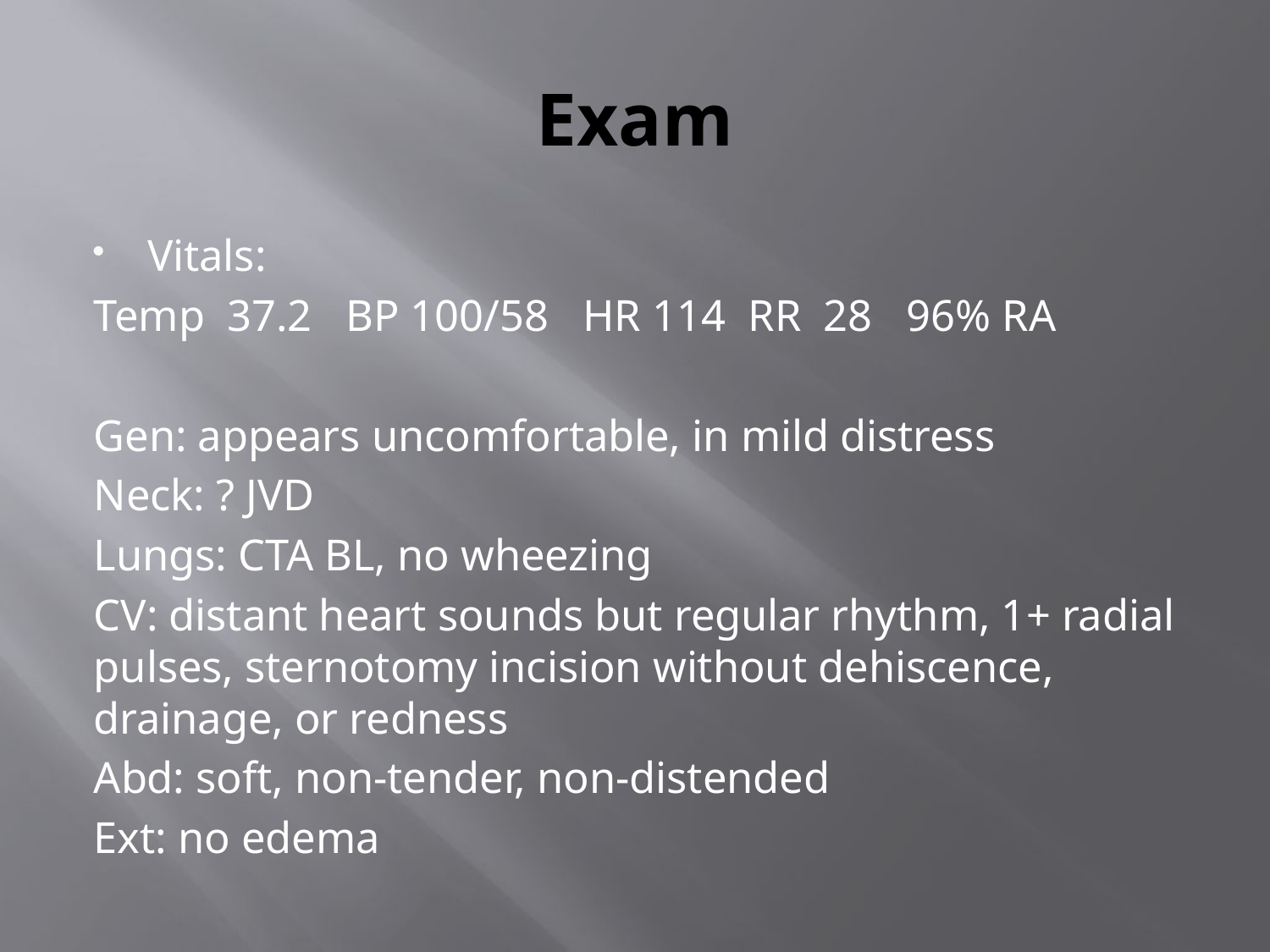

# Exam
Vitals:
Temp 37.2 BP 100/58 HR 114 RR 28 96% RA
Gen: appears uncomfortable, in mild distress
Neck: ? JVD
Lungs: CTA BL, no wheezing
CV: distant heart sounds but regular rhythm, 1+ radial pulses, sternotomy incision without dehiscence, drainage, or redness
Abd: soft, non-tender, non-distended
Ext: no edema

## Slide 6
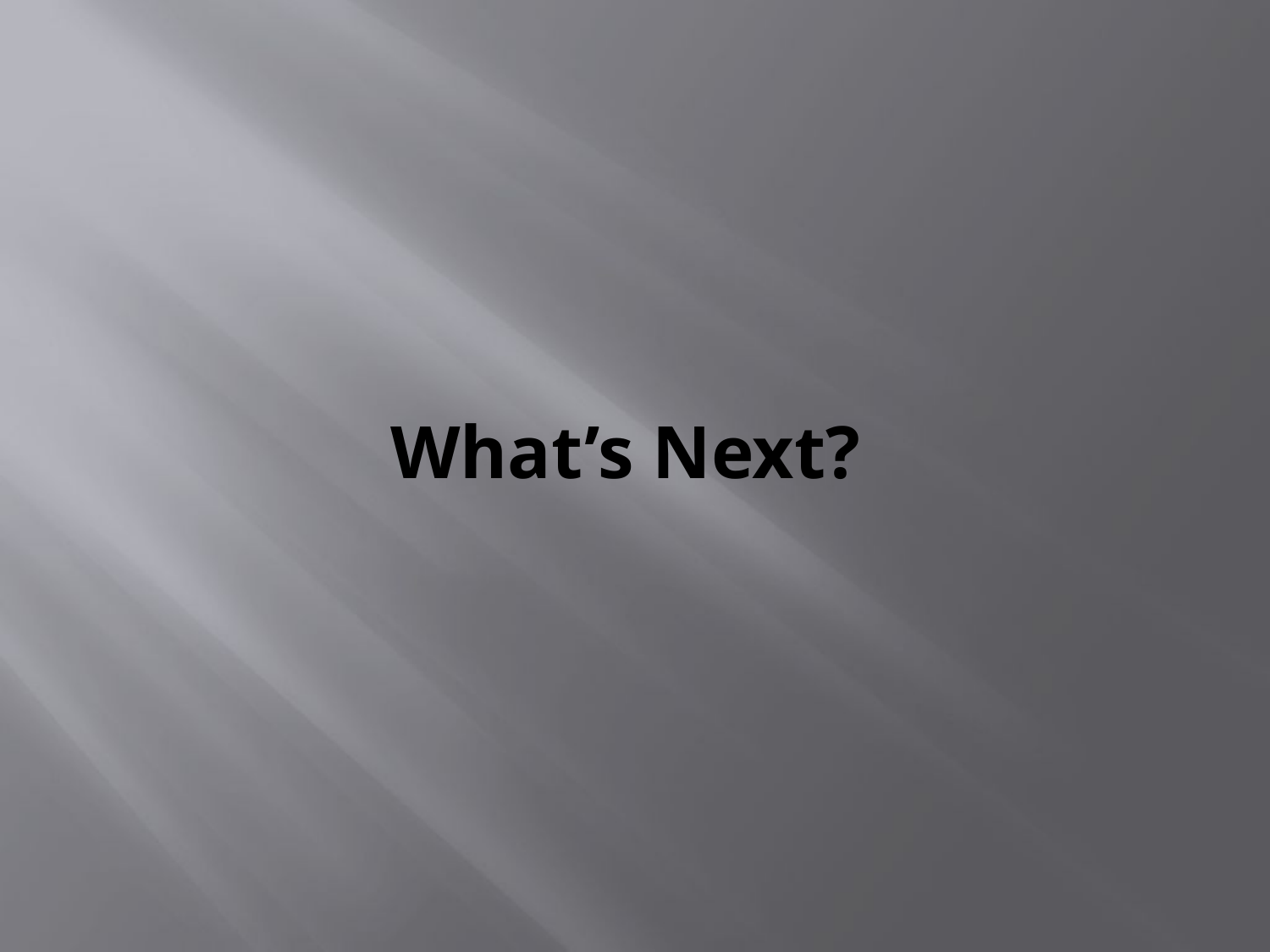

# What’s Next?

## Slide 7
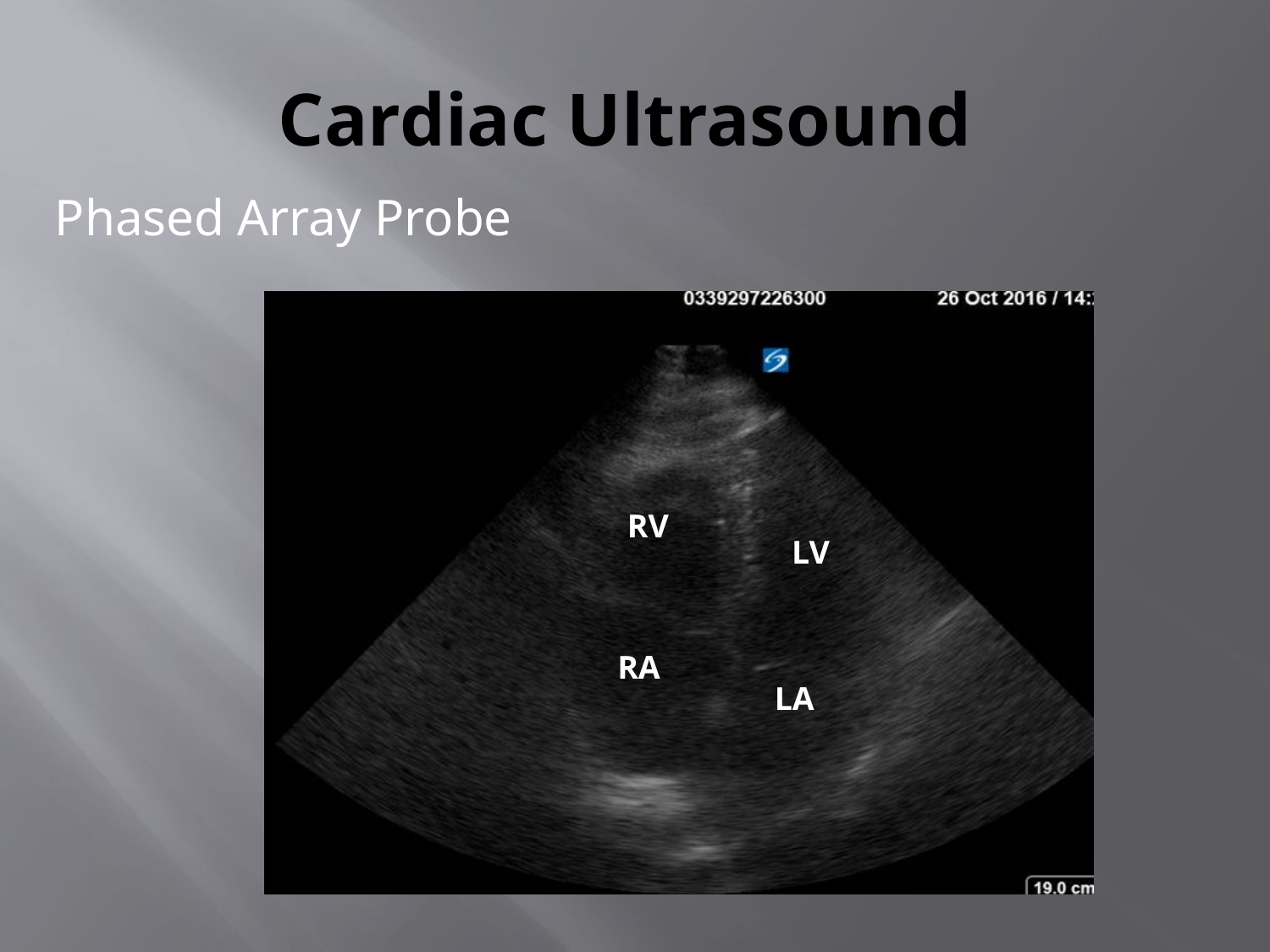

# Cardiac Ultrasound
Phased Array Probe
RV
LV
RA
LA

## Slide 8
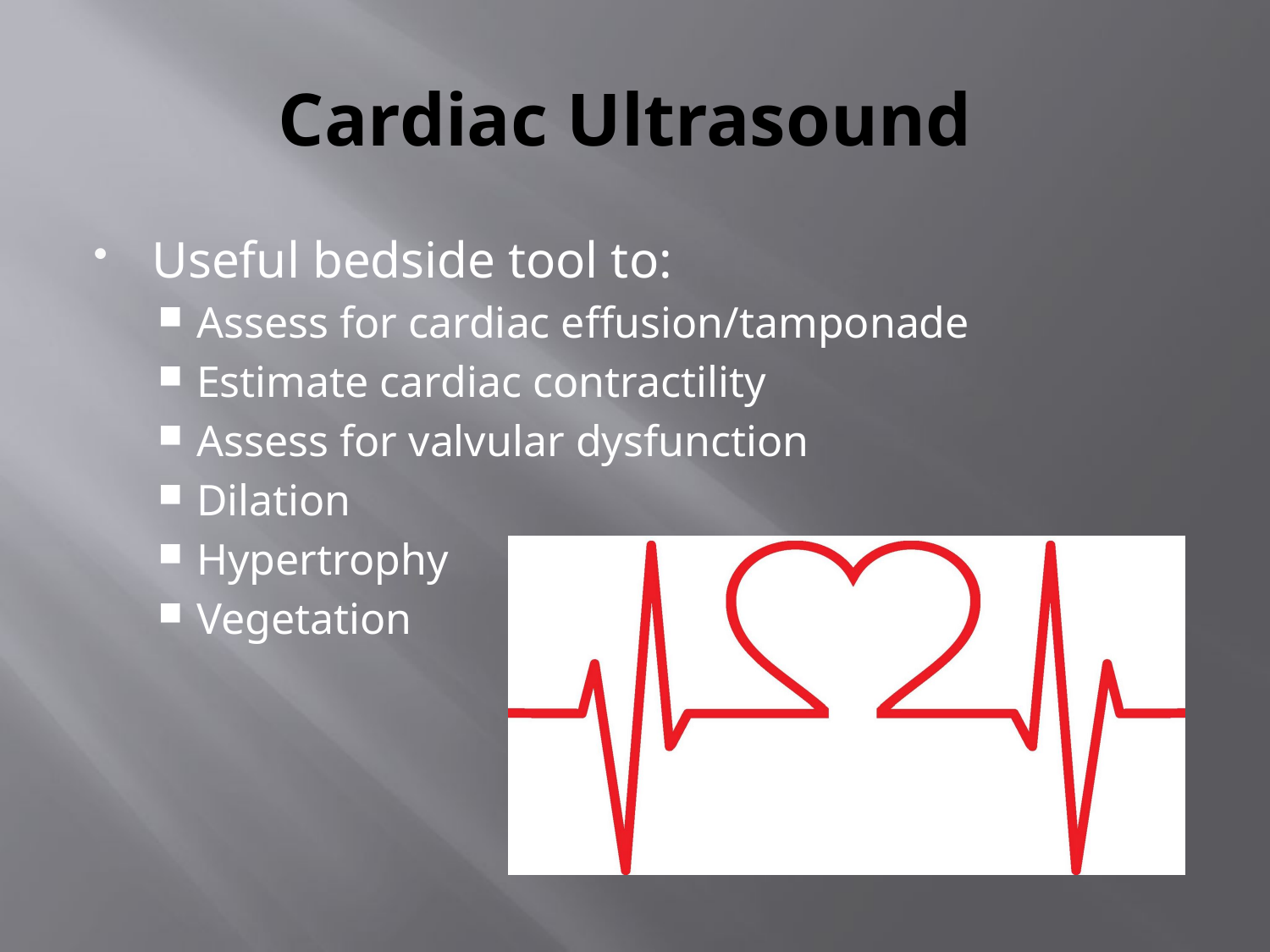

# Cardiac Ultrasound
Useful bedside tool to:
Assess for cardiac effusion/tamponade
Estimate cardiac contractility
Assess for valvular dysfunction
Dilation
Hypertrophy
Vegetation

## Slide 9
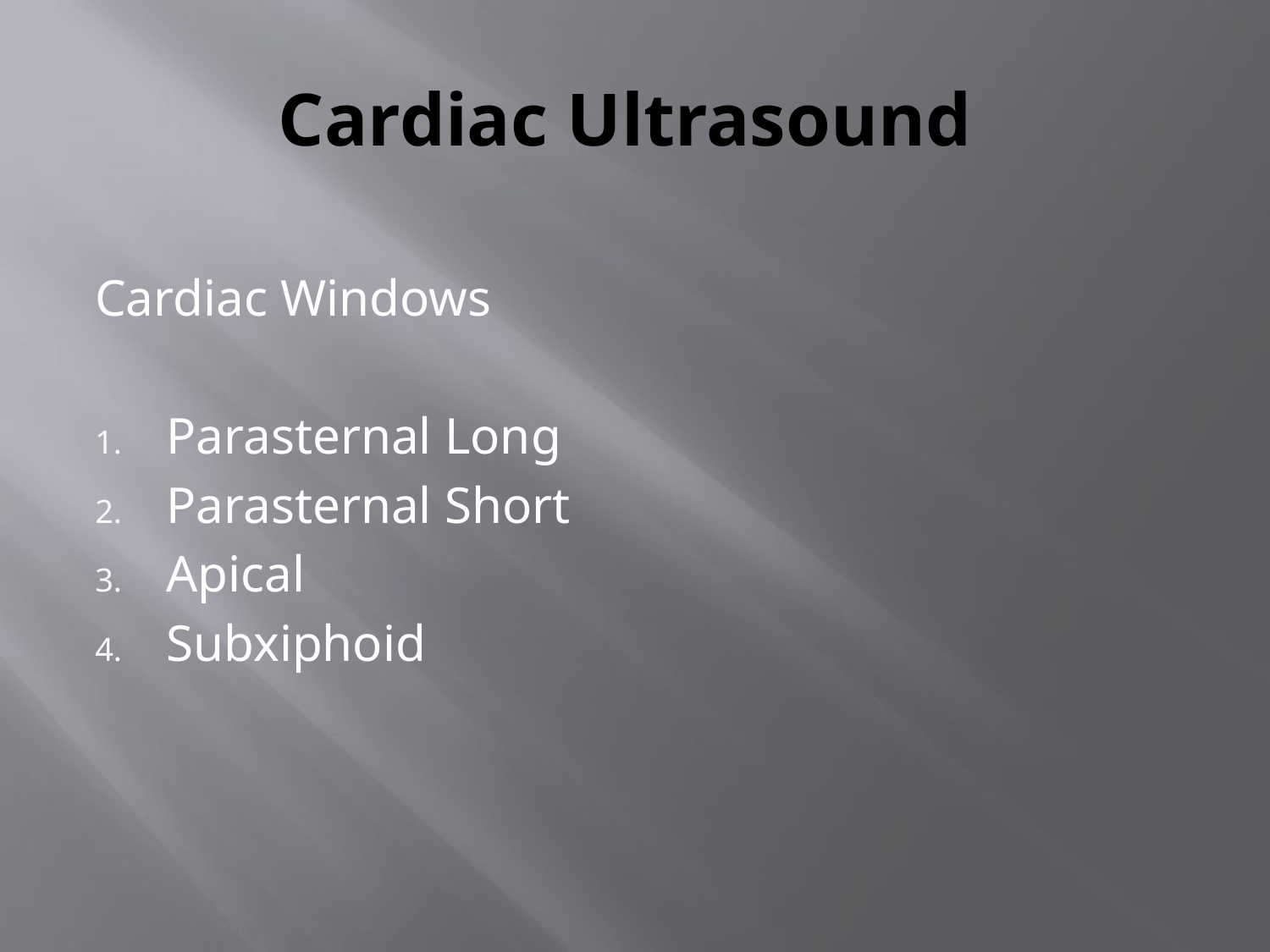

# Cardiac Ultrasound
Cardiac Windows
Parasternal Long
Parasternal Short
Apical
Subxiphoid

## Slide 10
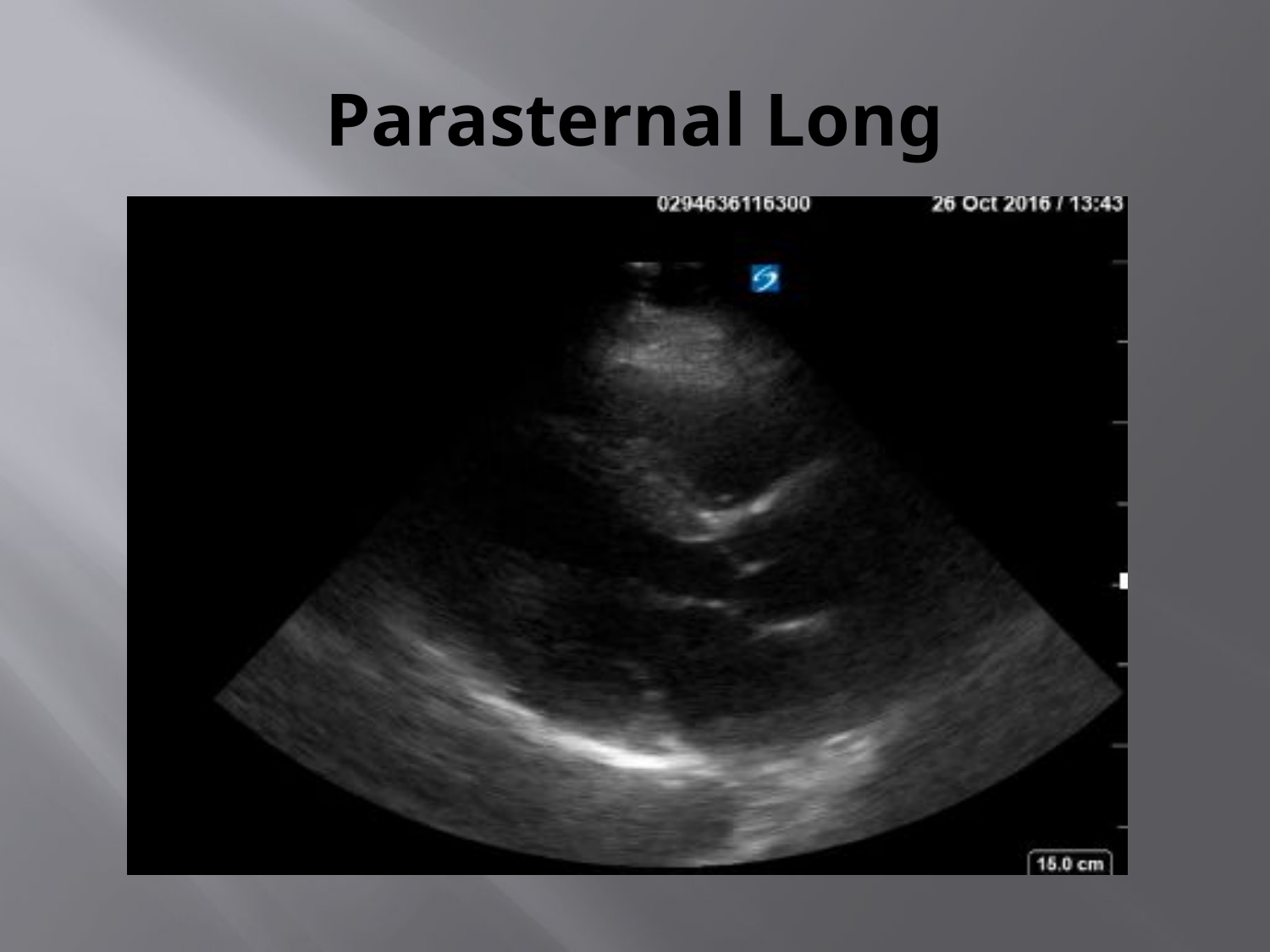

# Parasternal Long

## Slide 11
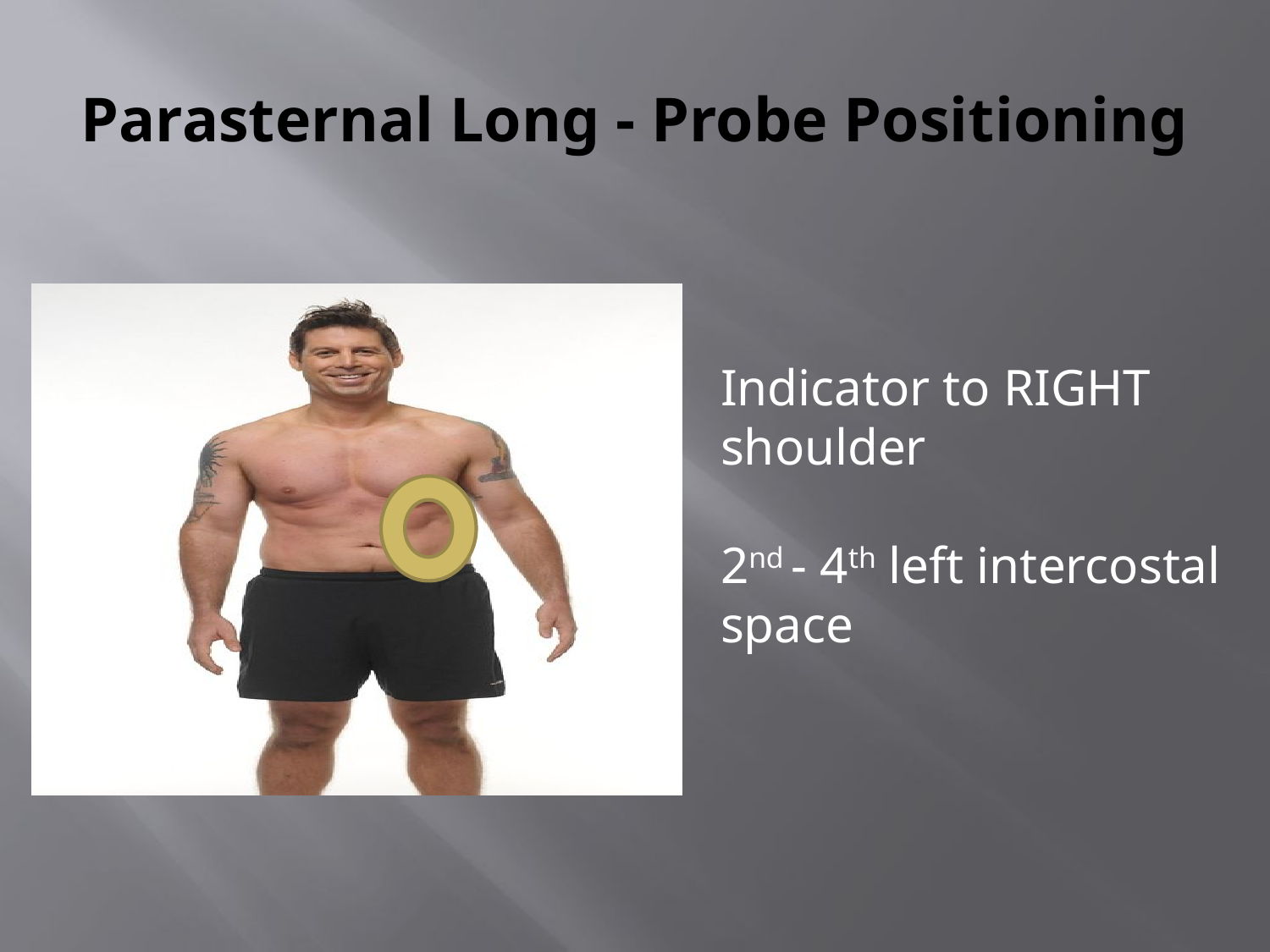

# Parasternal Long - Probe Positioning
Indicator to RIGHT shoulder
2nd - 4th left intercostal space

## Slide 12
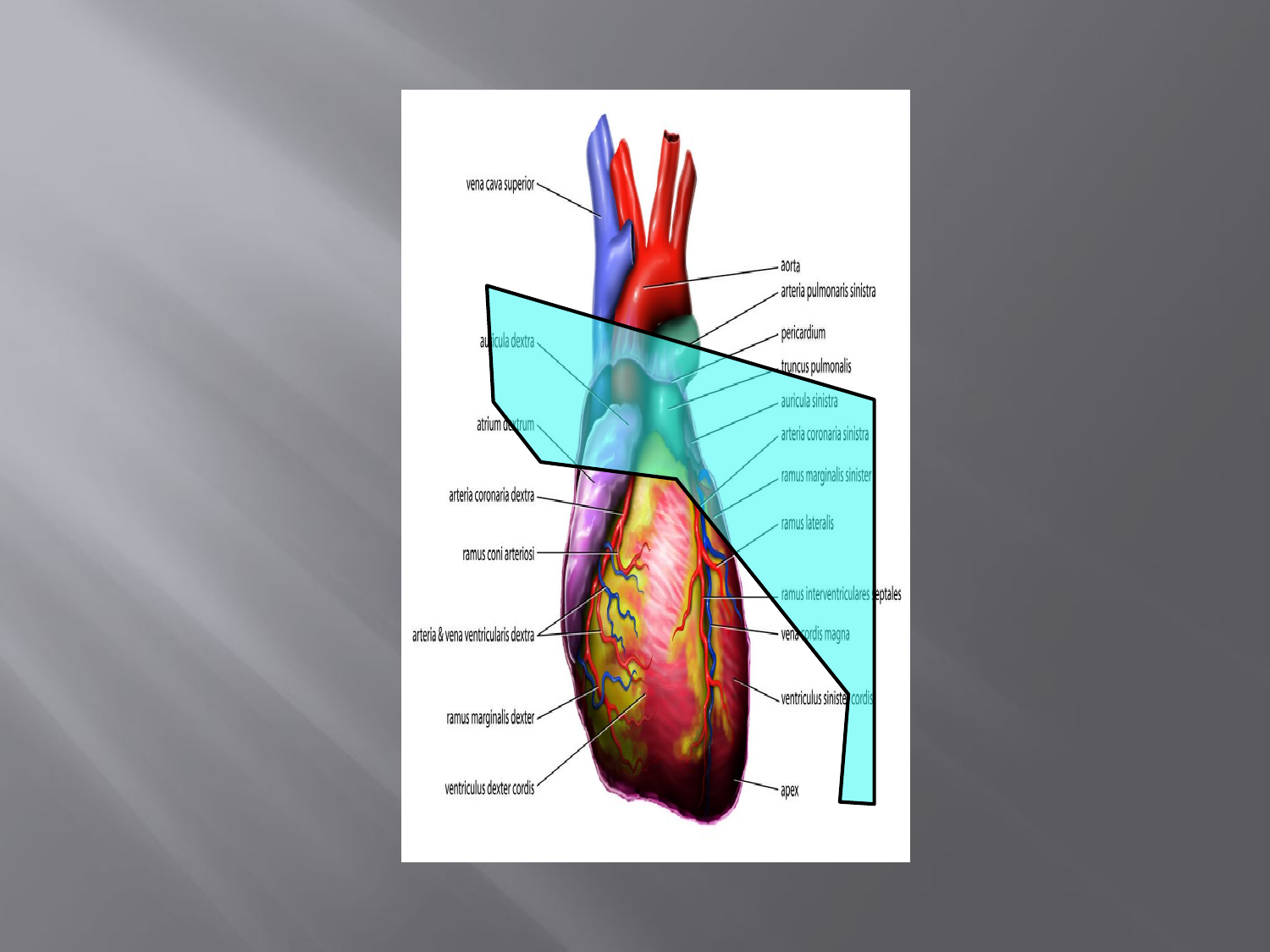

## Slide 13
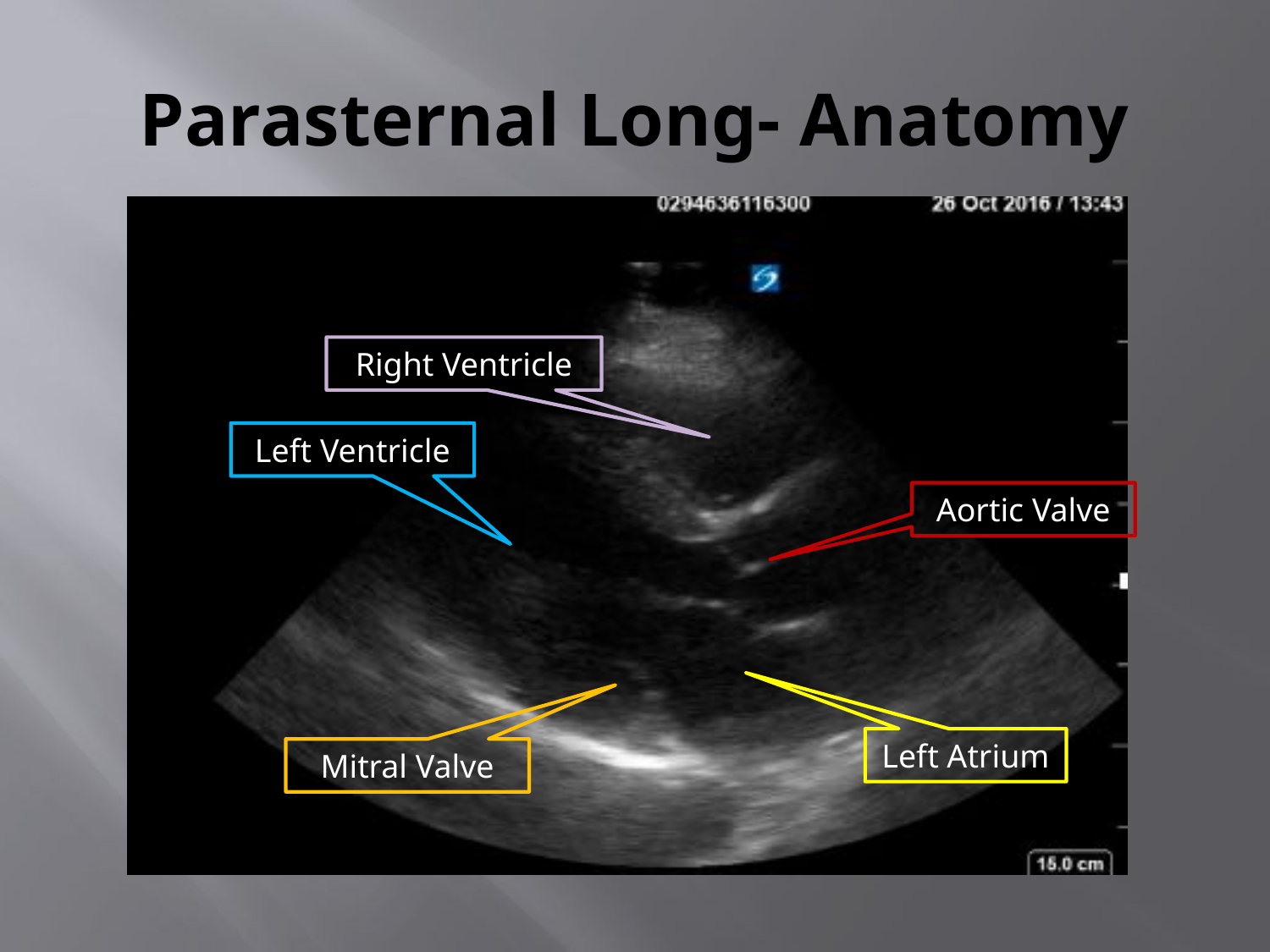

# Parasternal Long- Anatomy
Right Ventricle
Left Ventricle
Aortic Valve
Left Atrium
Mitral Valve

## Slide 14
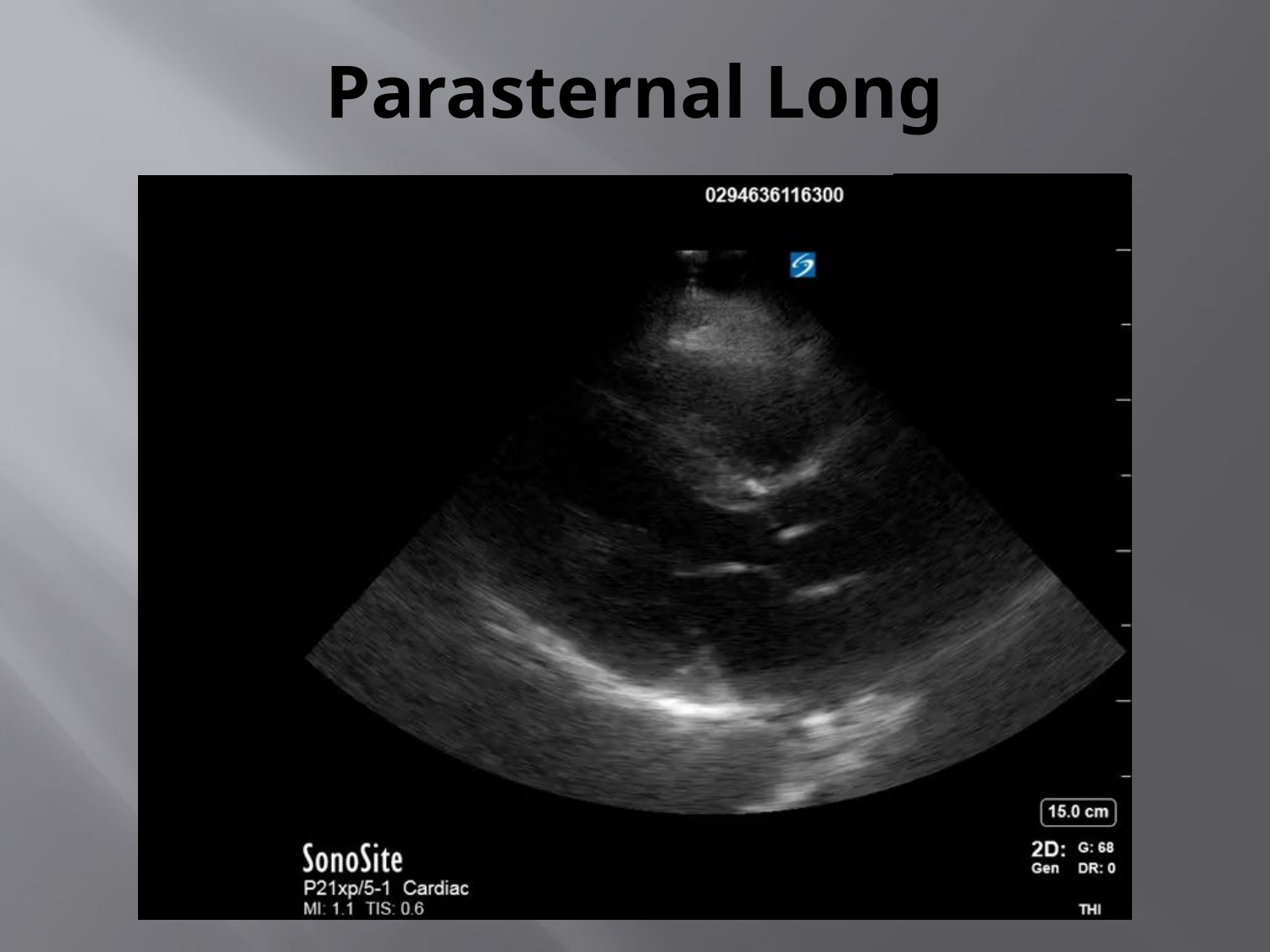

# Parasternal Long

## Slide 15
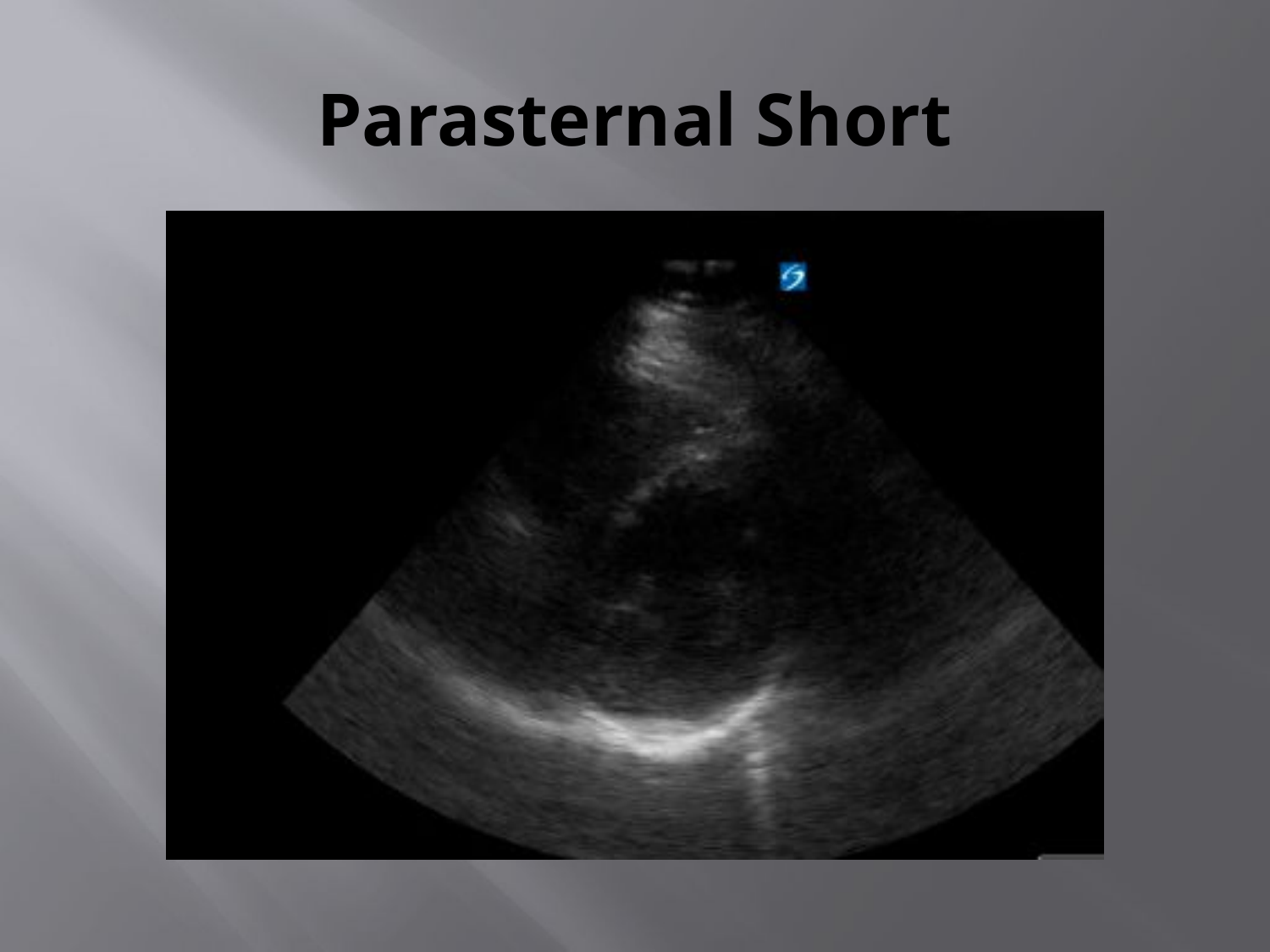

# Parasternal Short

## Slide 16
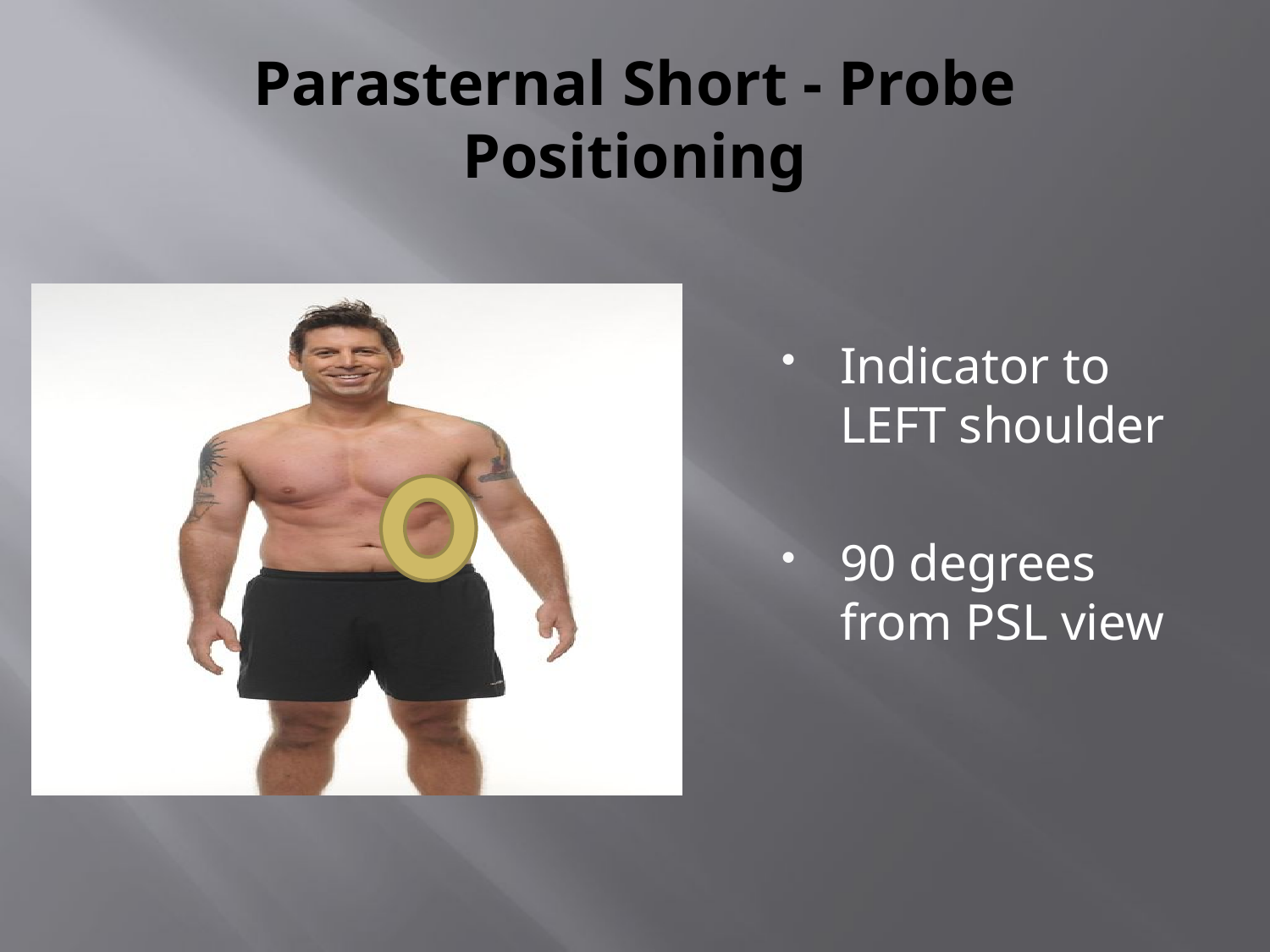

# Parasternal Short - Probe Positioning
Indicator to LEFT shoulder
90 degrees from PSL view

## Slide 17
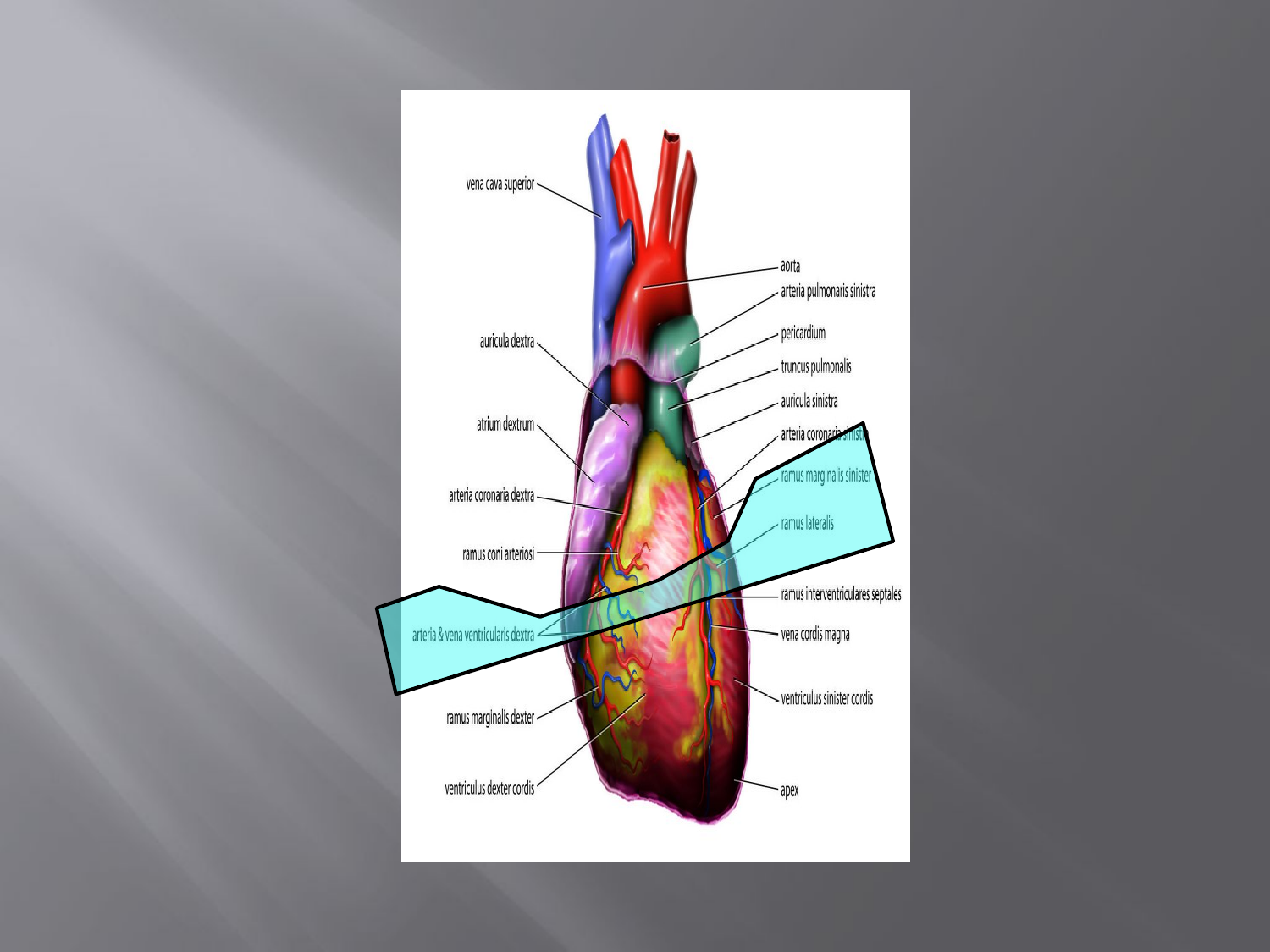

## Slide 18
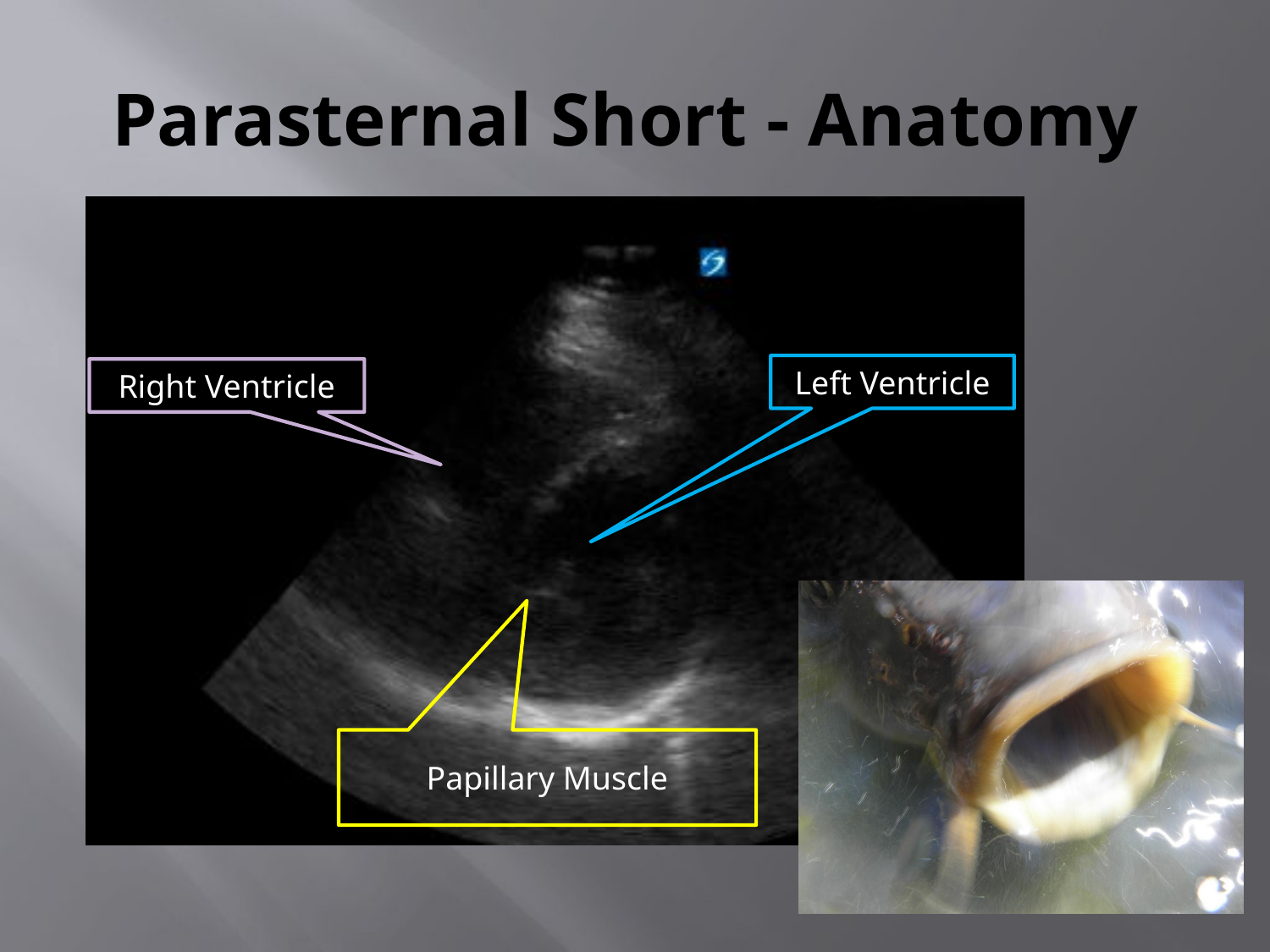

# Parasternal Short - Anatomy
Left Ventricle
Right Ventricle
Papillary Muscle

## Slide 19
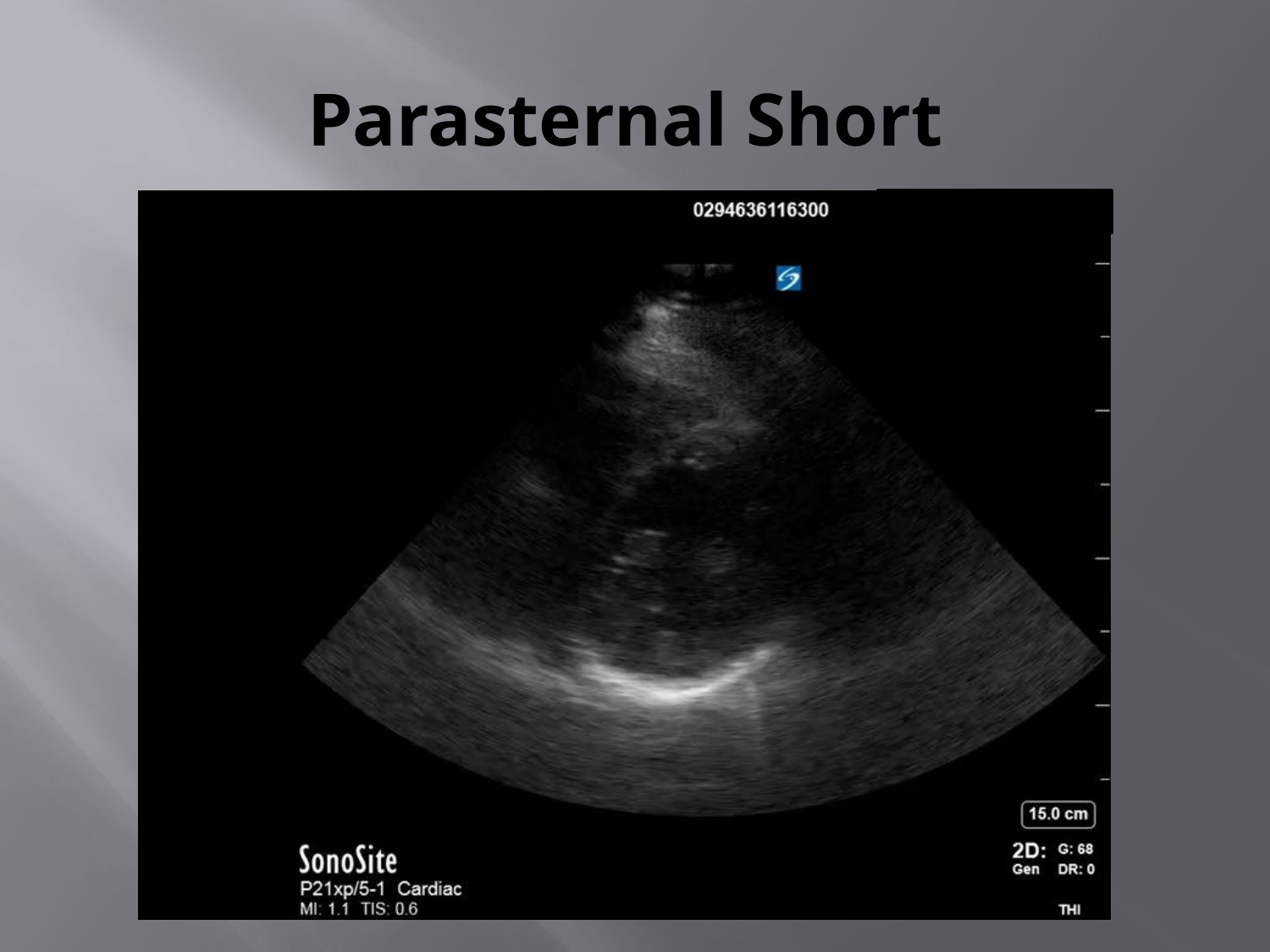

# Parasternal Short

## Slide 20
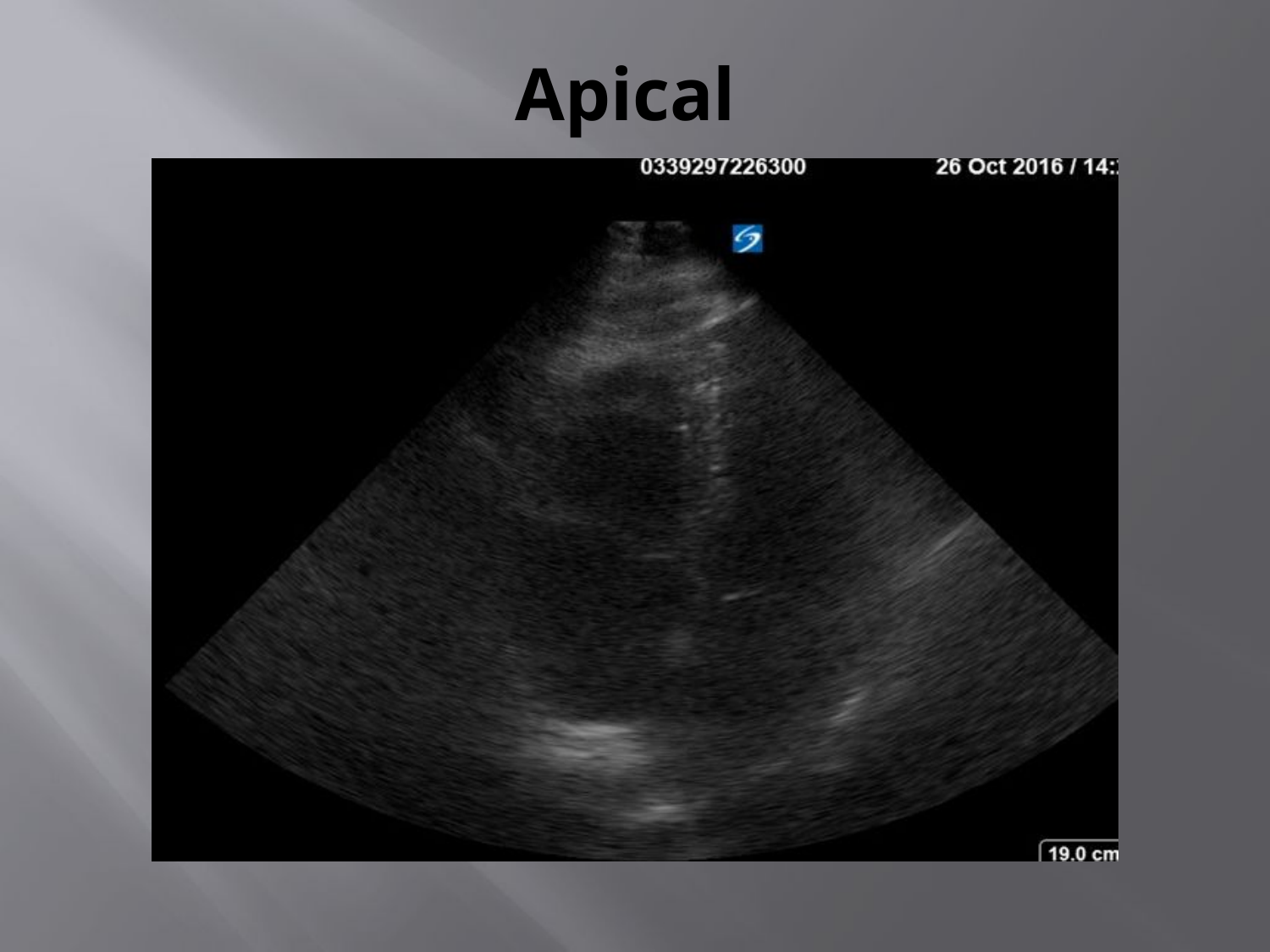

# Apical

## Slide 21
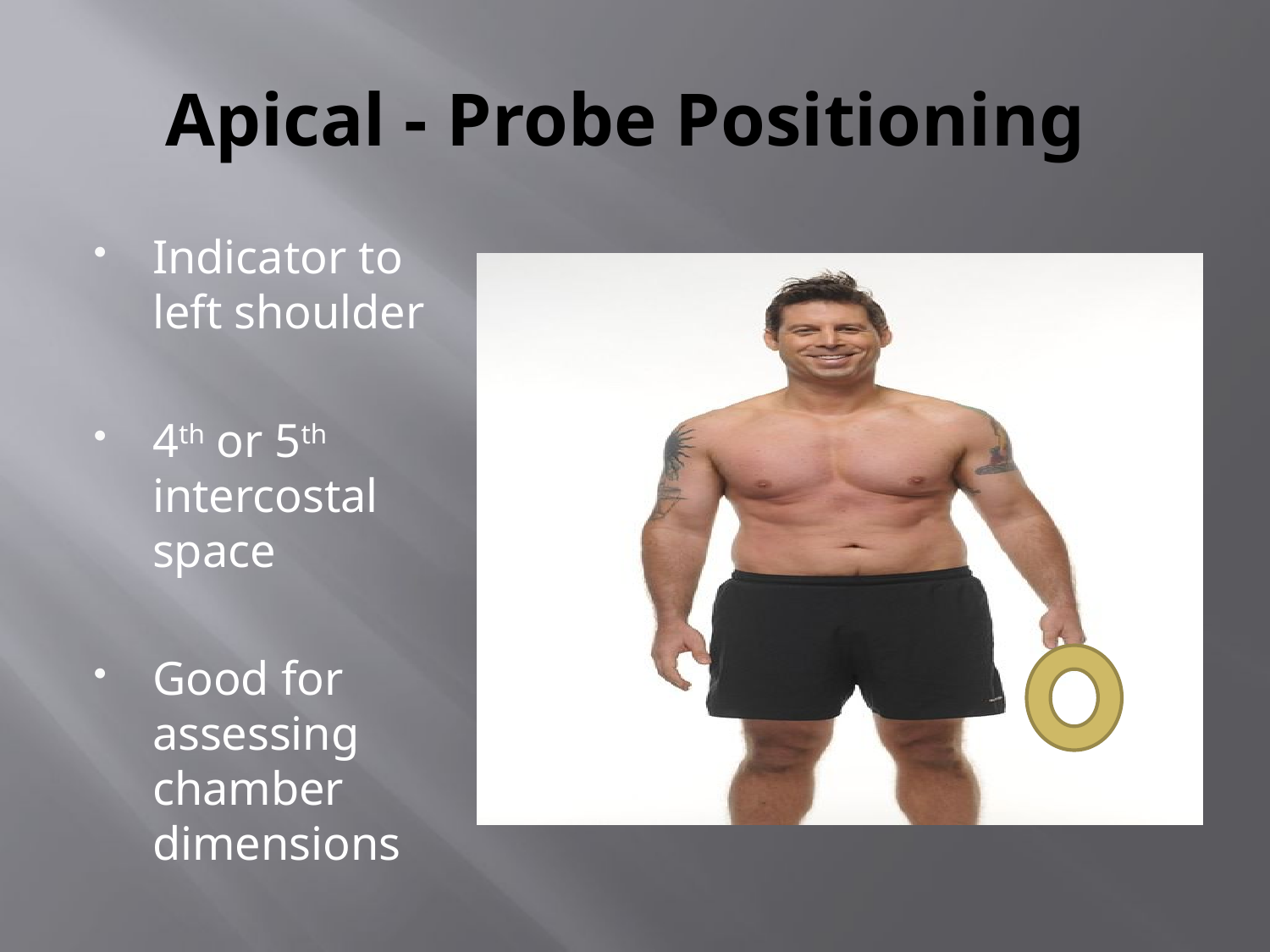

# Apical - Probe Positioning
Indicator to left shoulder
4th or 5th intercostal space
Good for assessing chamber dimensions

## Slide 22
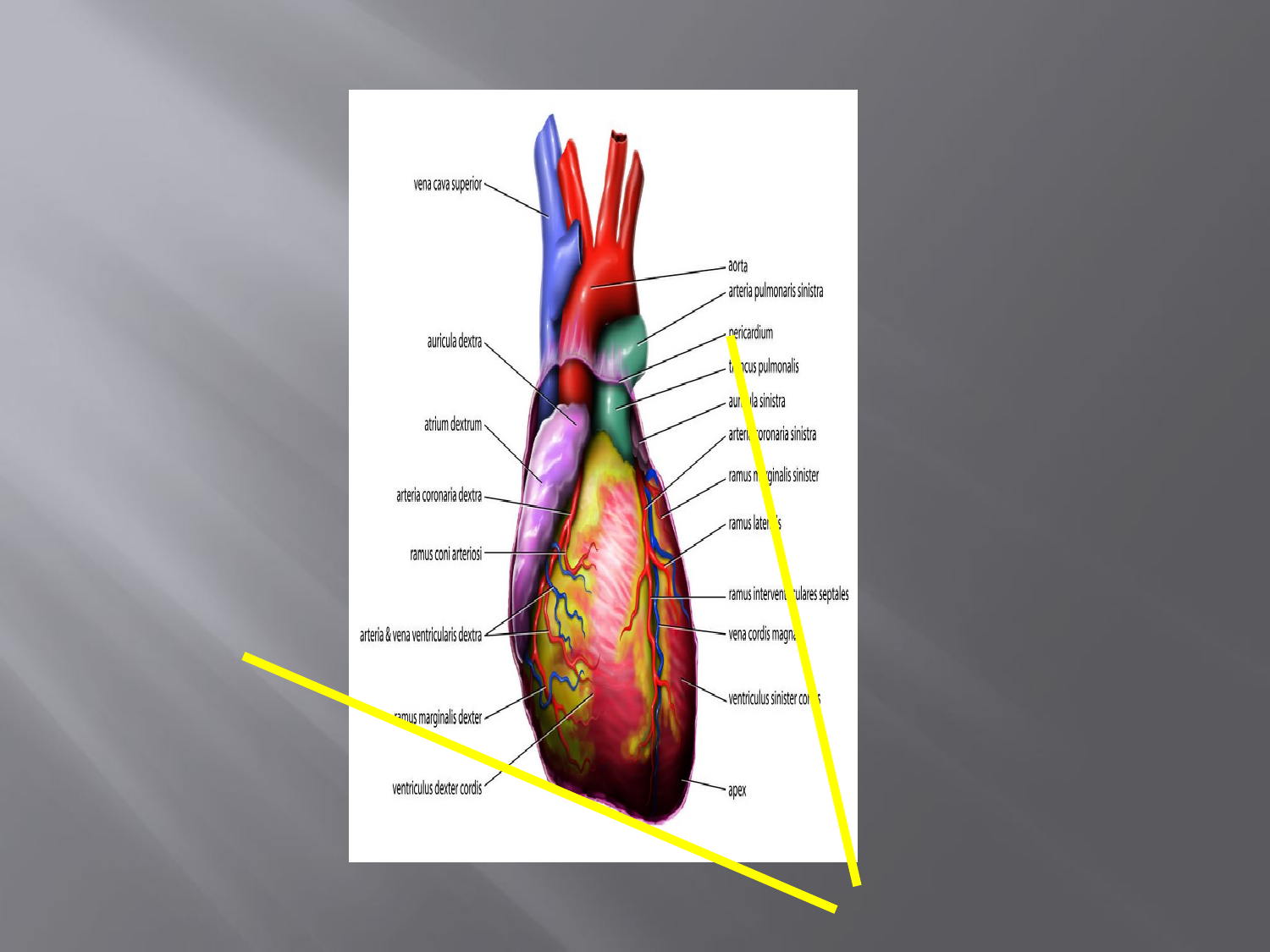

## Slide 23
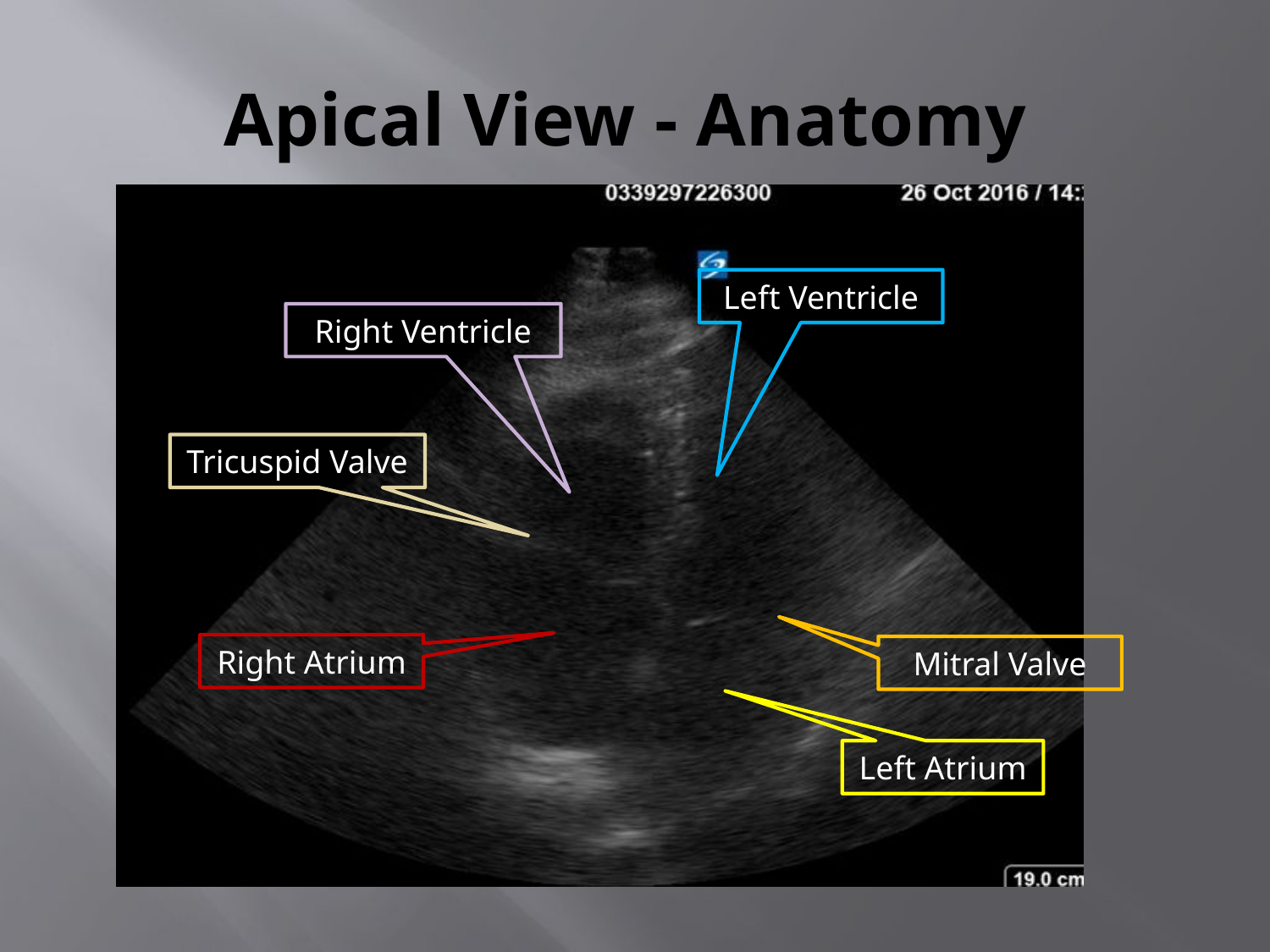

# Apical View - Anatomy
Left Ventricle
Right Ventricle
Tricuspid Valve
Right Atrium
Mitral Valve
Left Atrium

## Slide 24
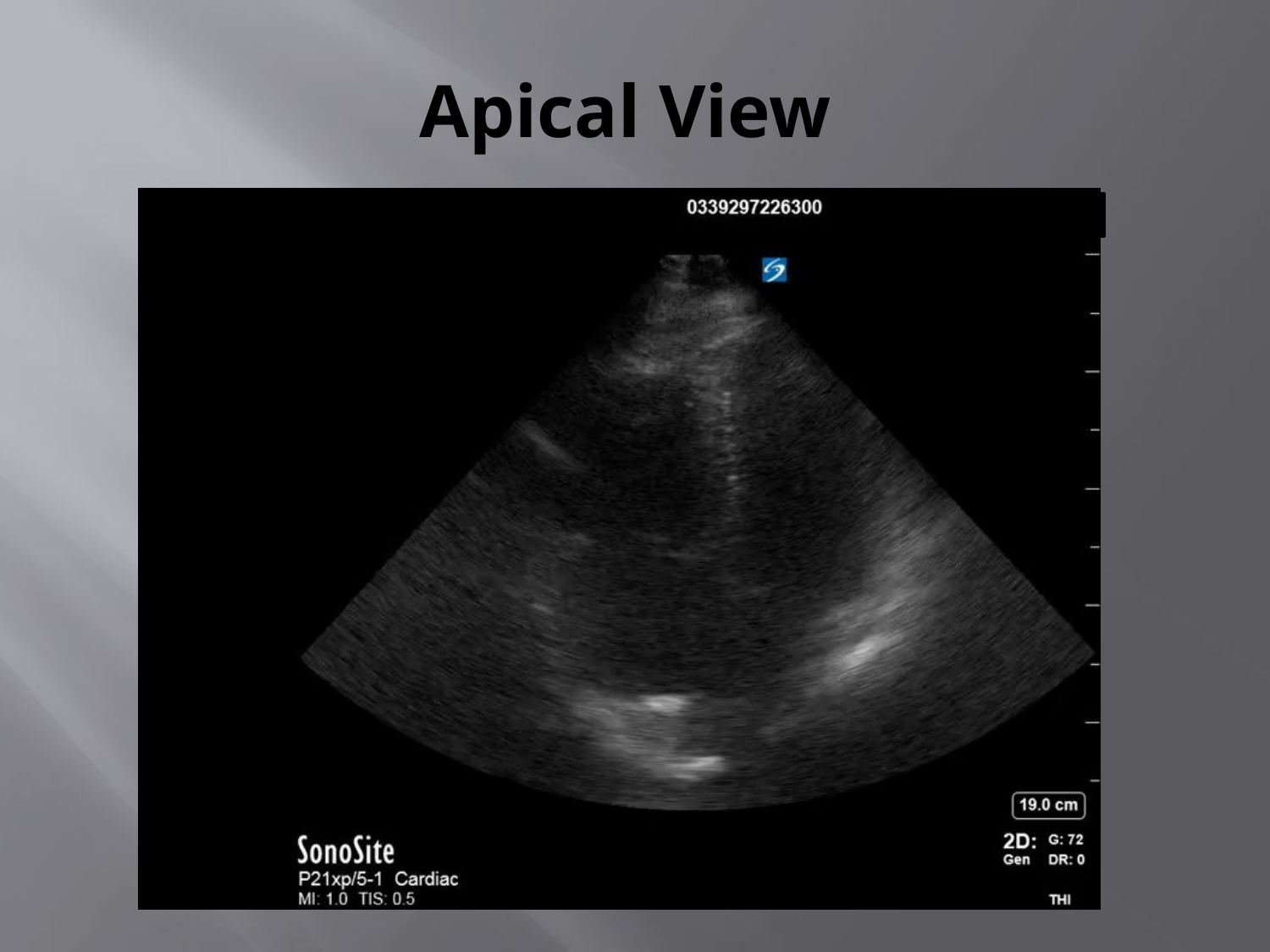

# Apical View

## Slide 25
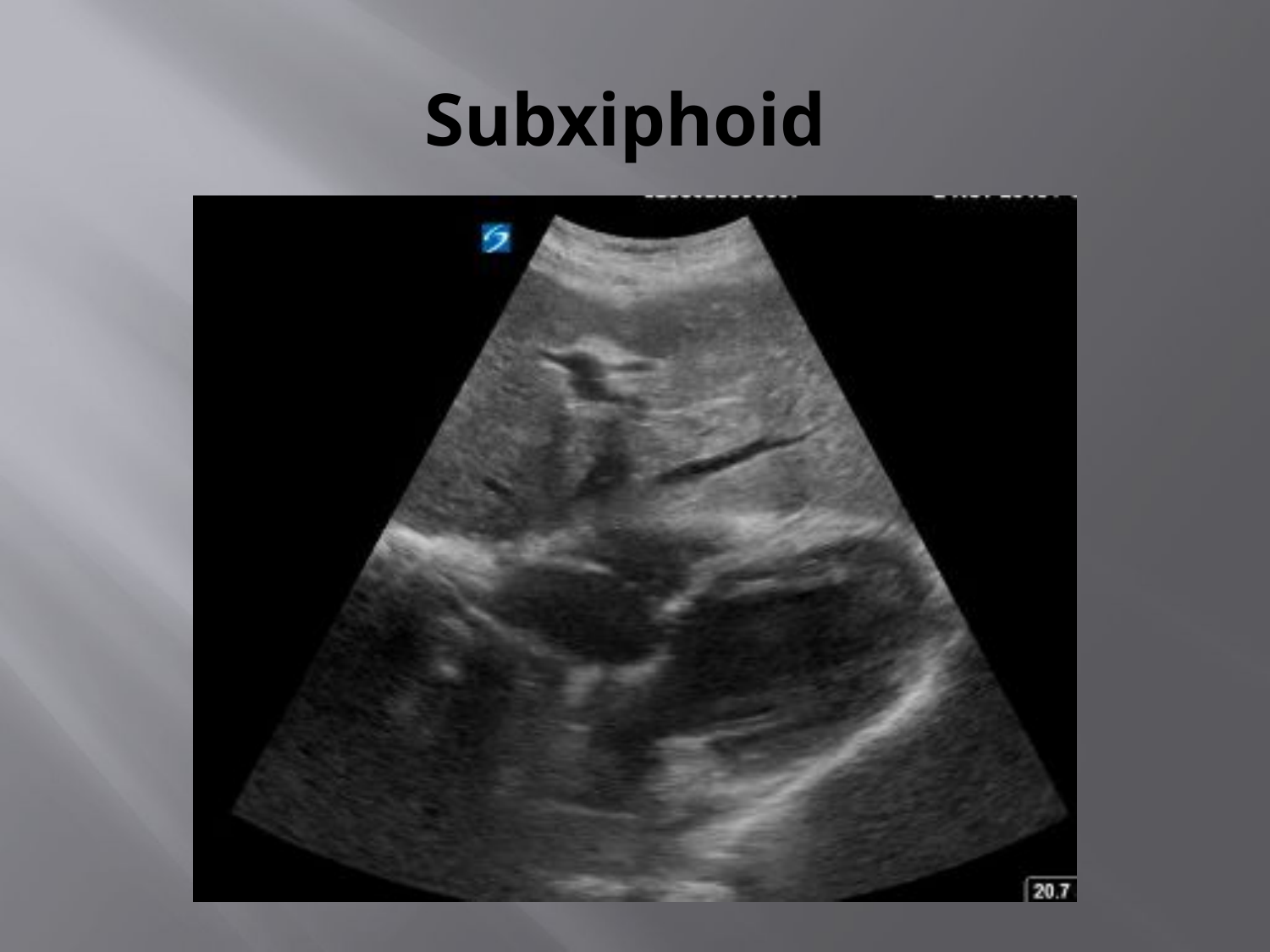

# Subxiphoid

## Slide 26
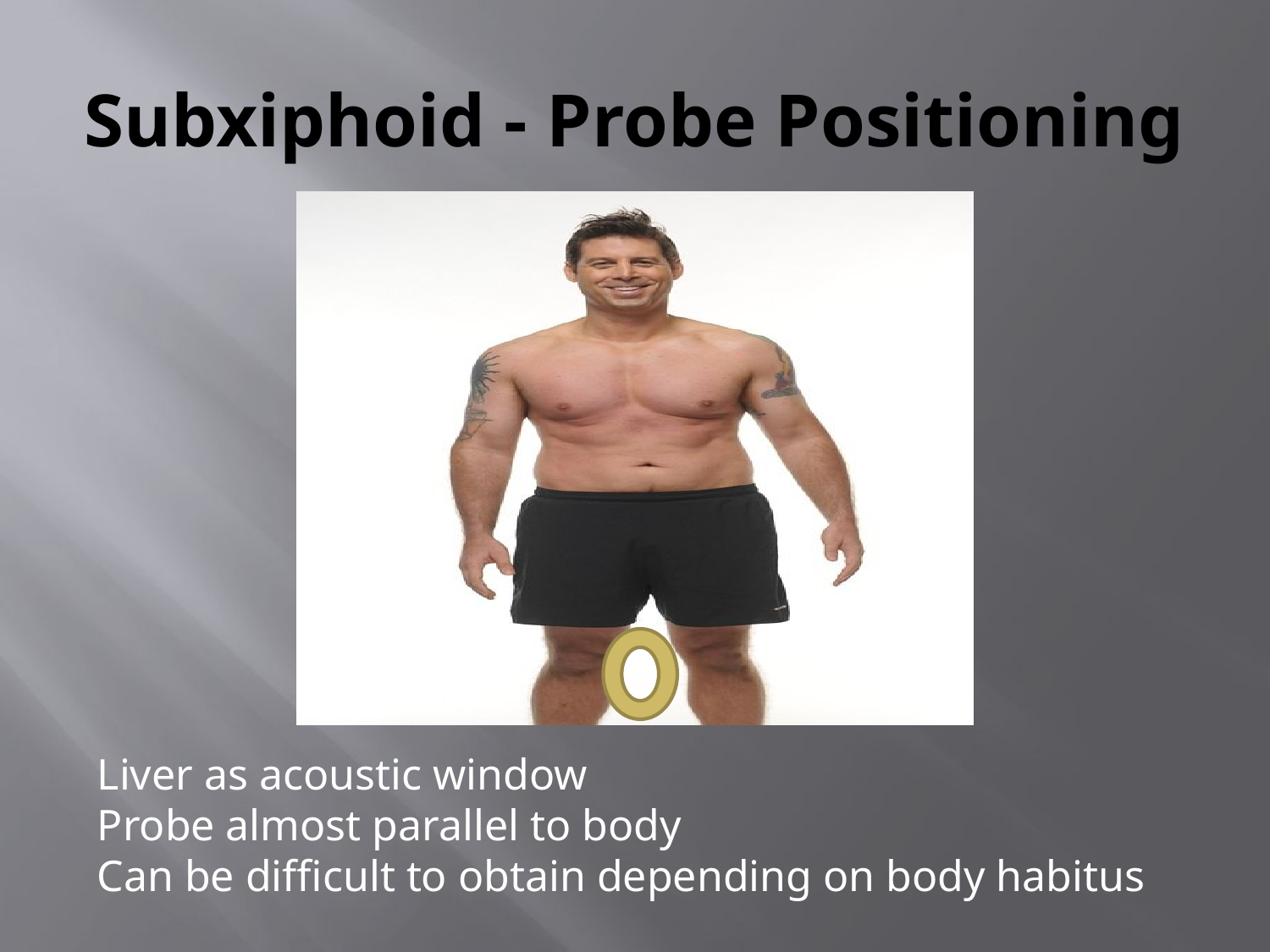

# Subxiphoid - Probe Positioning
Liver as acoustic window
Probe almost parallel to body
Can be difficult to obtain depending on body habitus

## Slide 27
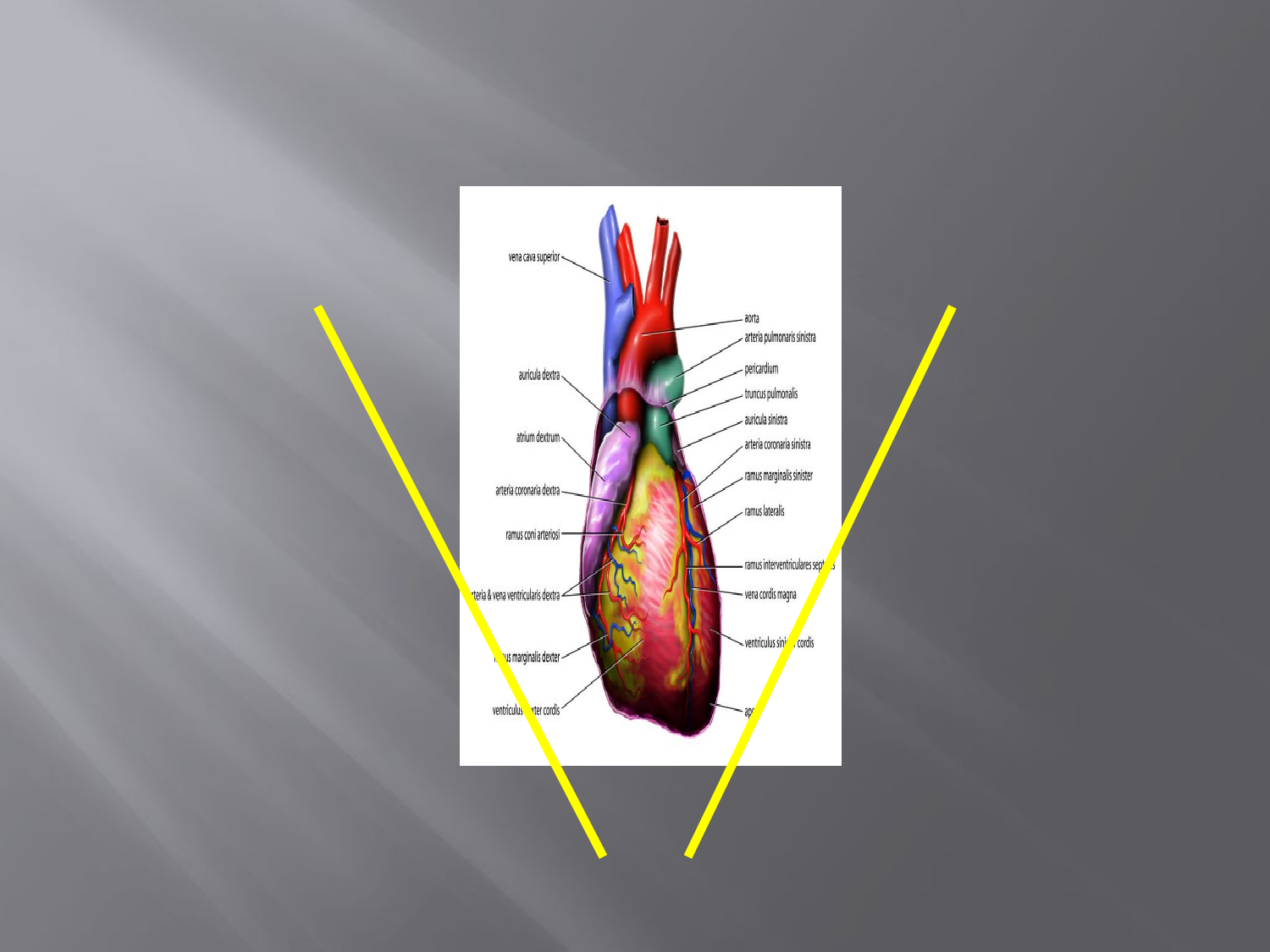

## Slide 28
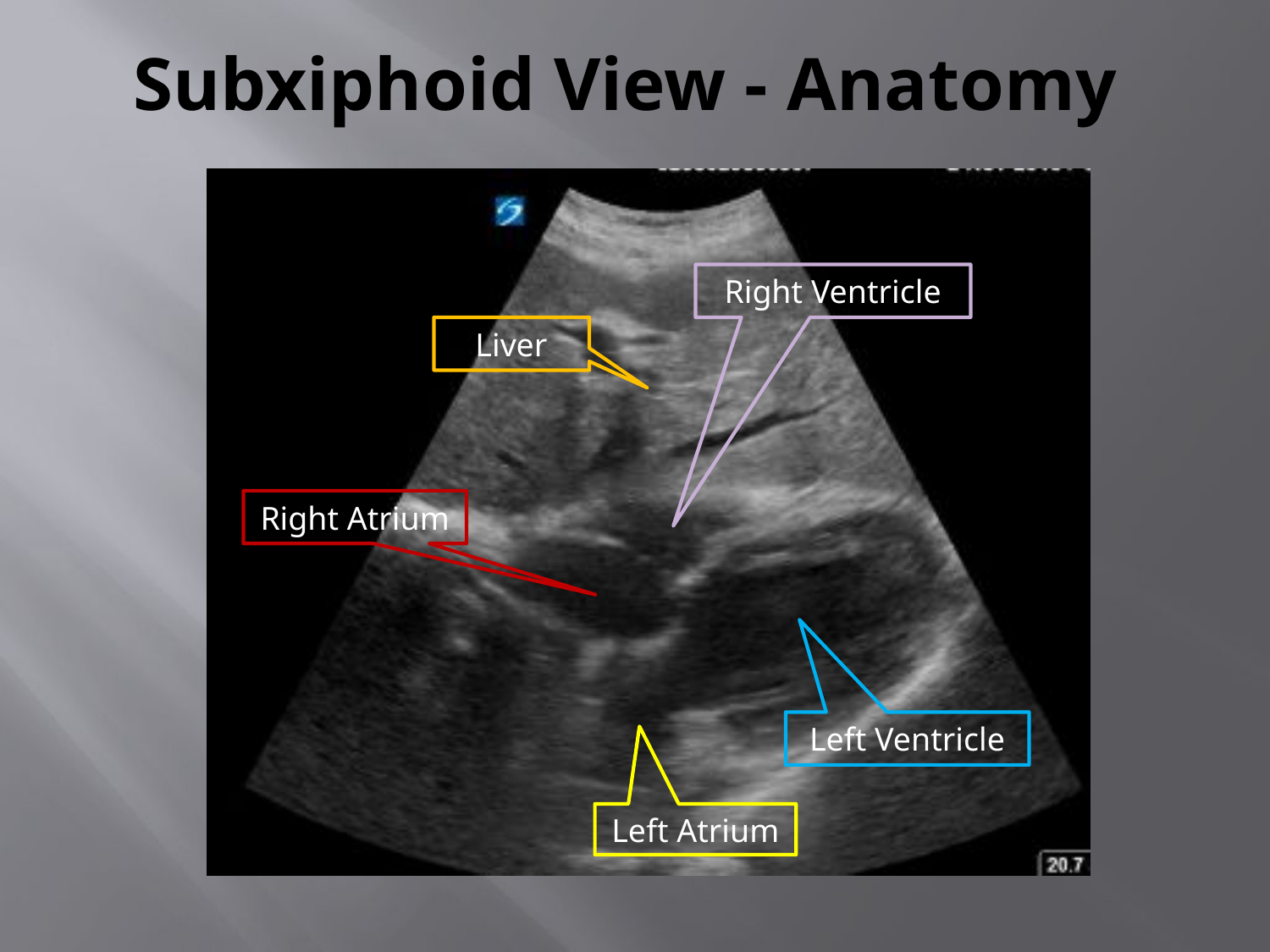

# Subxiphoid View - Anatomy
Right Ventricle
Liver
Right Atrium
Left Ventricle
Left Atrium

## Slide 29
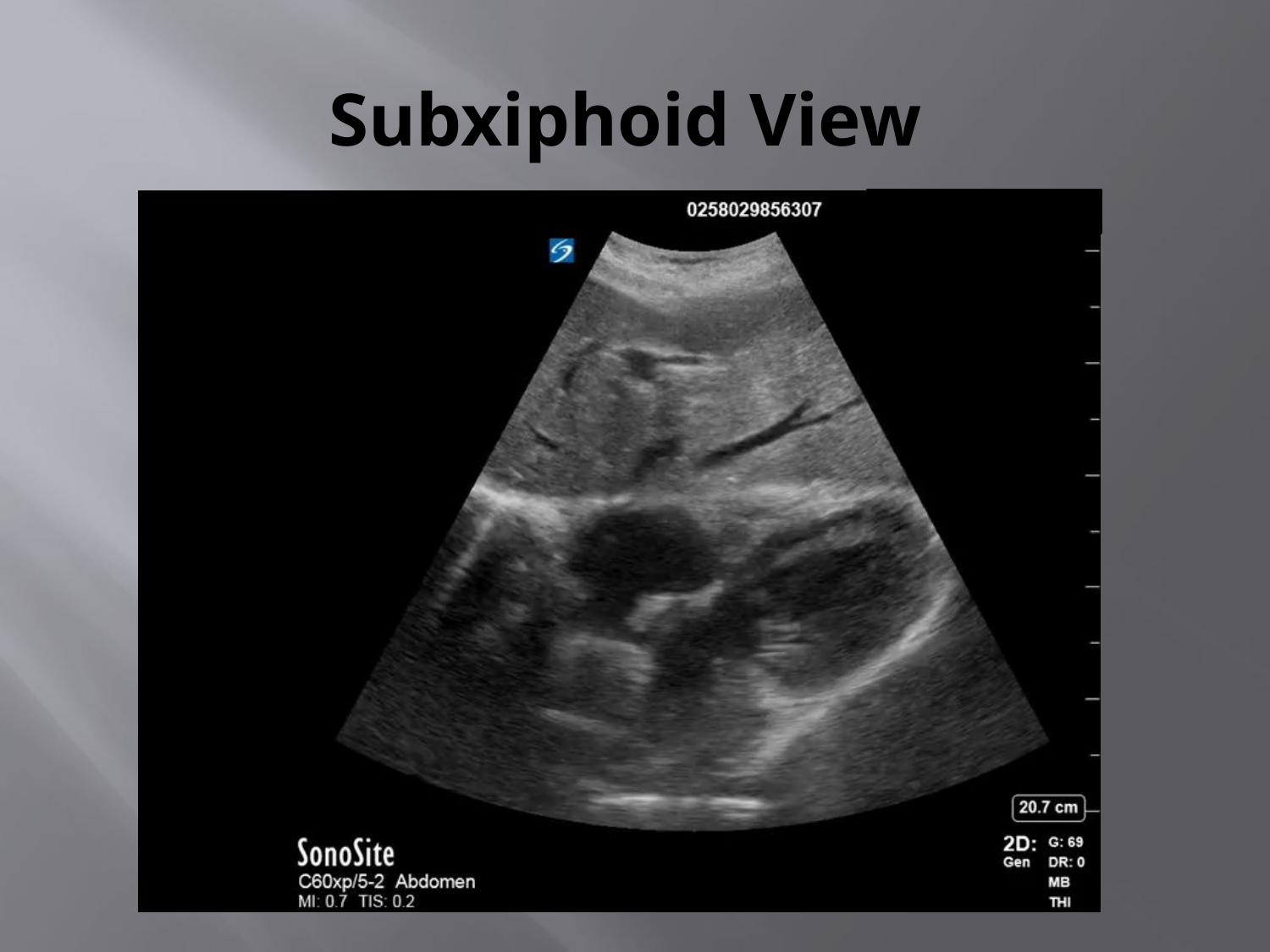

# Subxiphoid View

## Slide 30
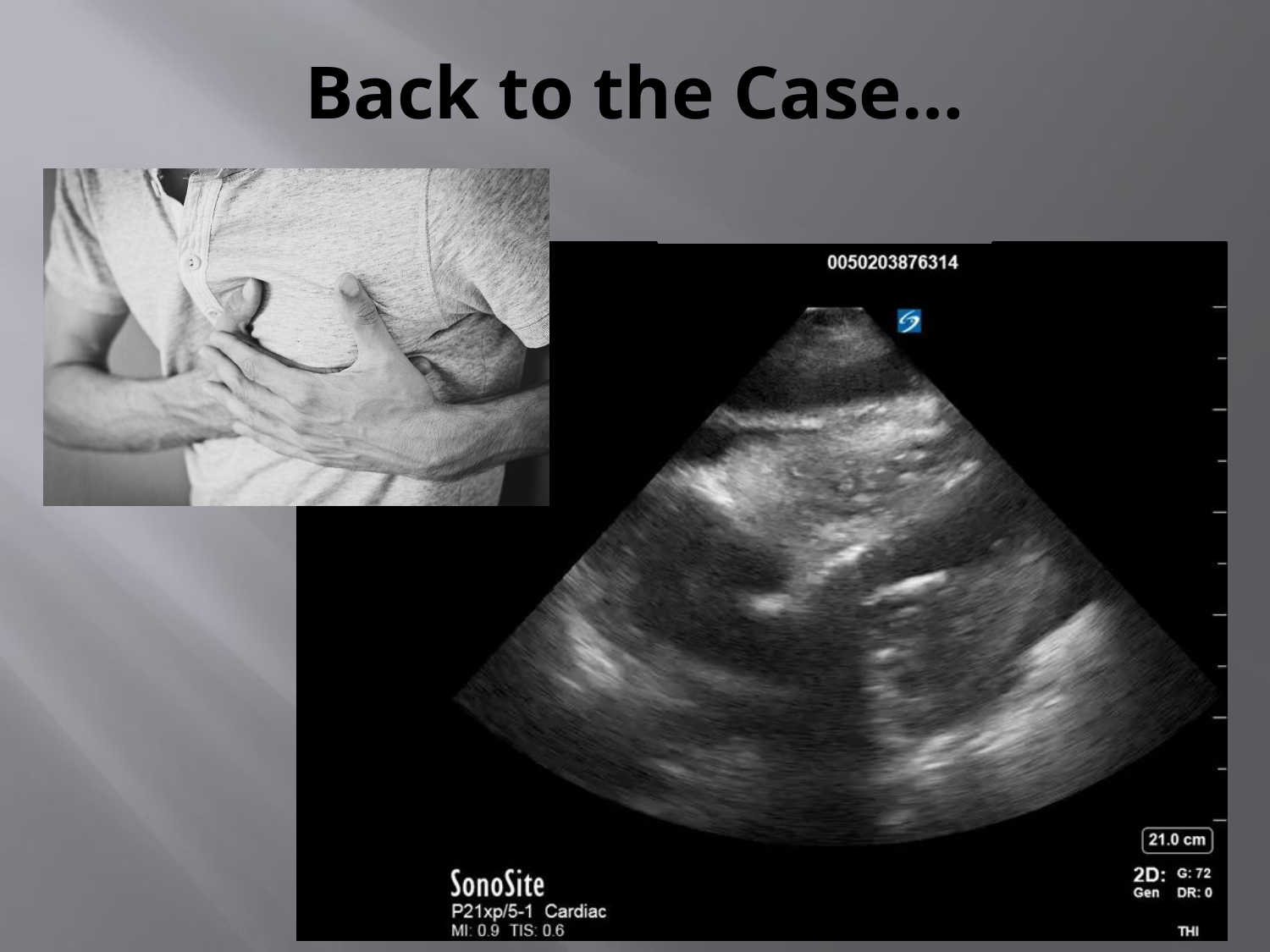

# Back to the Case…

## Slide 31
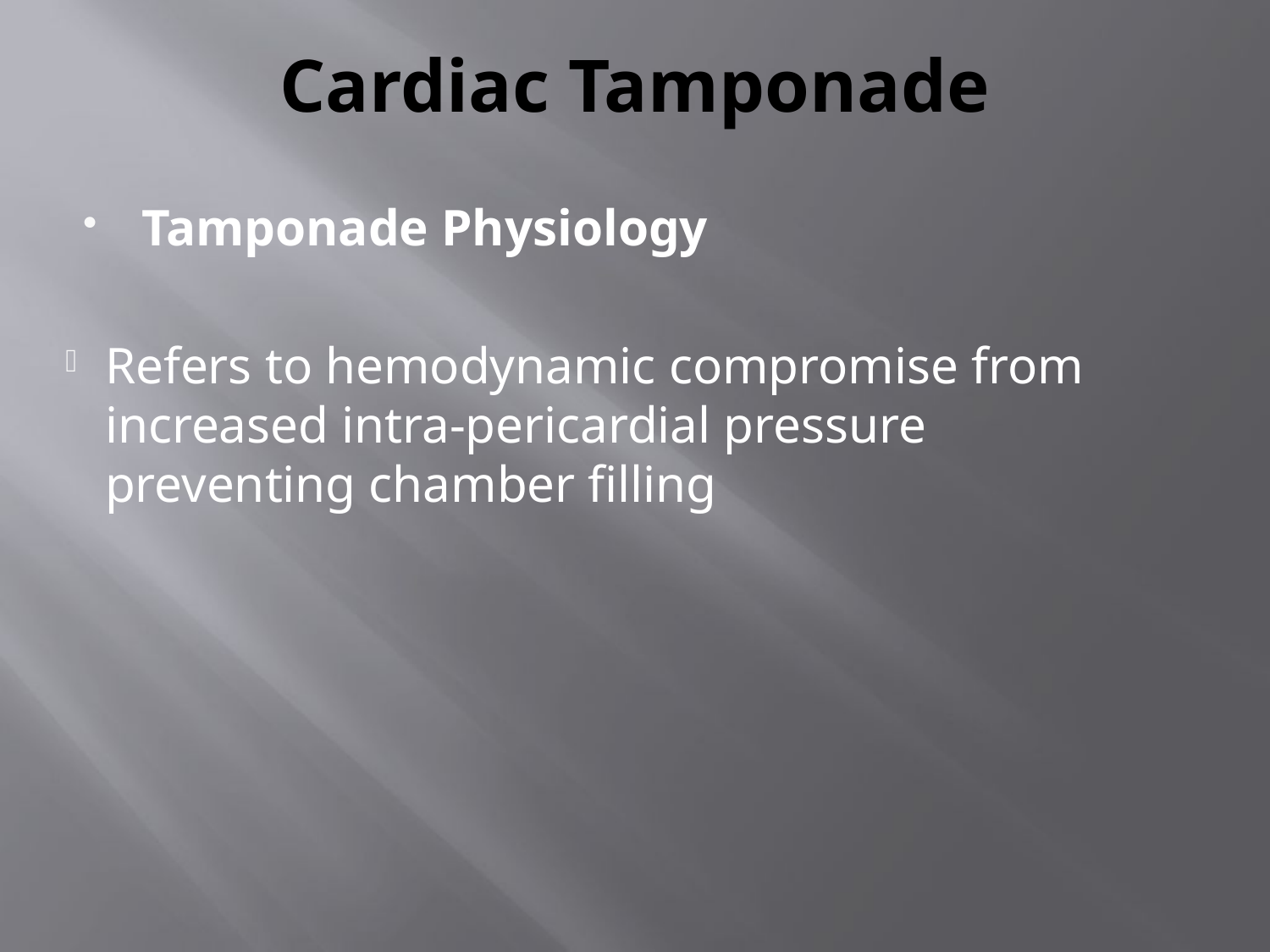

# Cardiac Tamponade
Tamponade Physiology
Refers to hemodynamic compromise from increased intra-pericardial pressure preventing chamber filling

## Slide 32
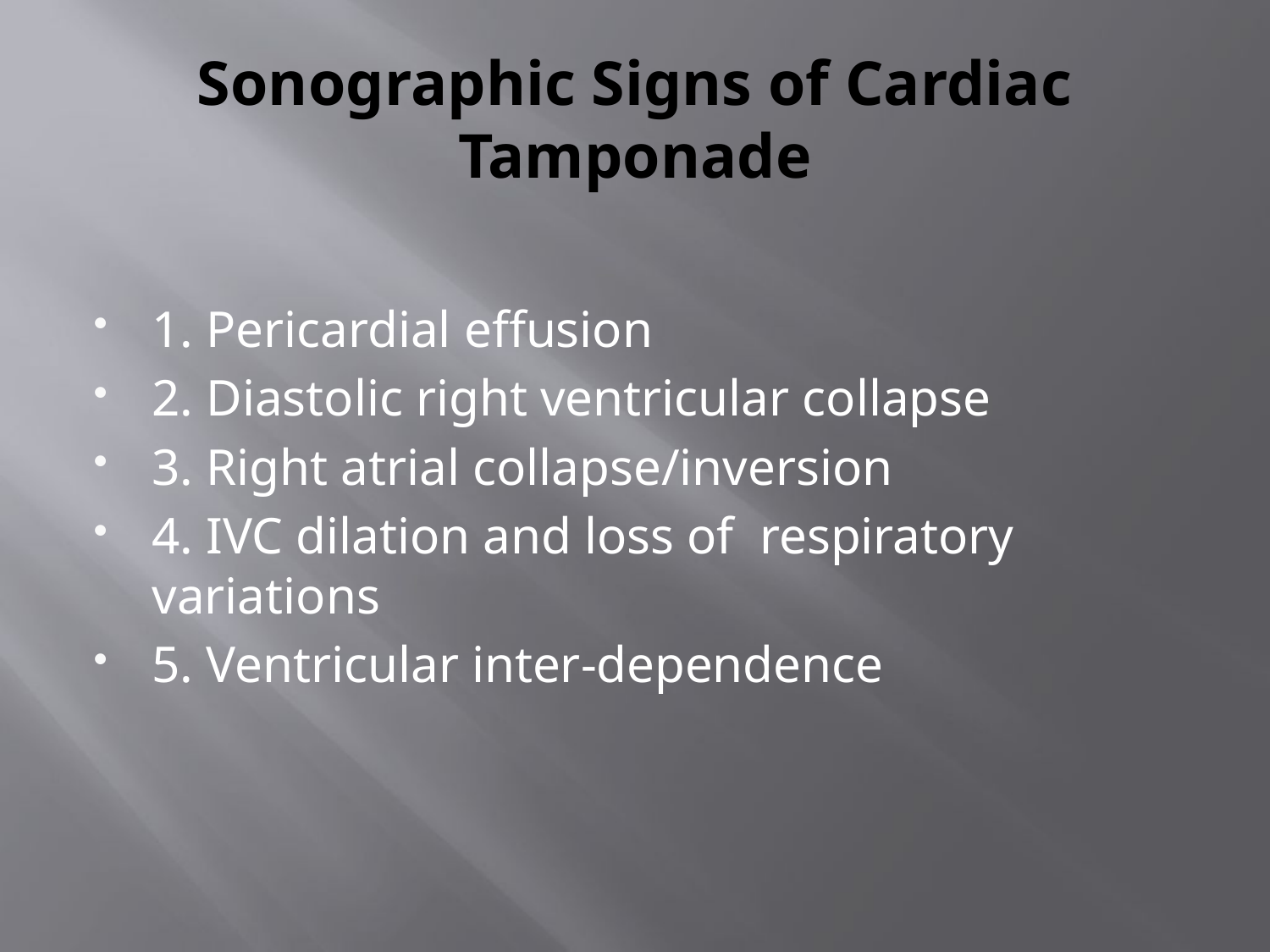

# Sonographic Signs of Cardiac Tamponade
1. Pericardial effusion
2. Diastolic right ventricular collapse
3. Right atrial collapse/inversion
4. IVC dilation and loss of respiratory variations
5. Ventricular inter-dependence

## Slide 33
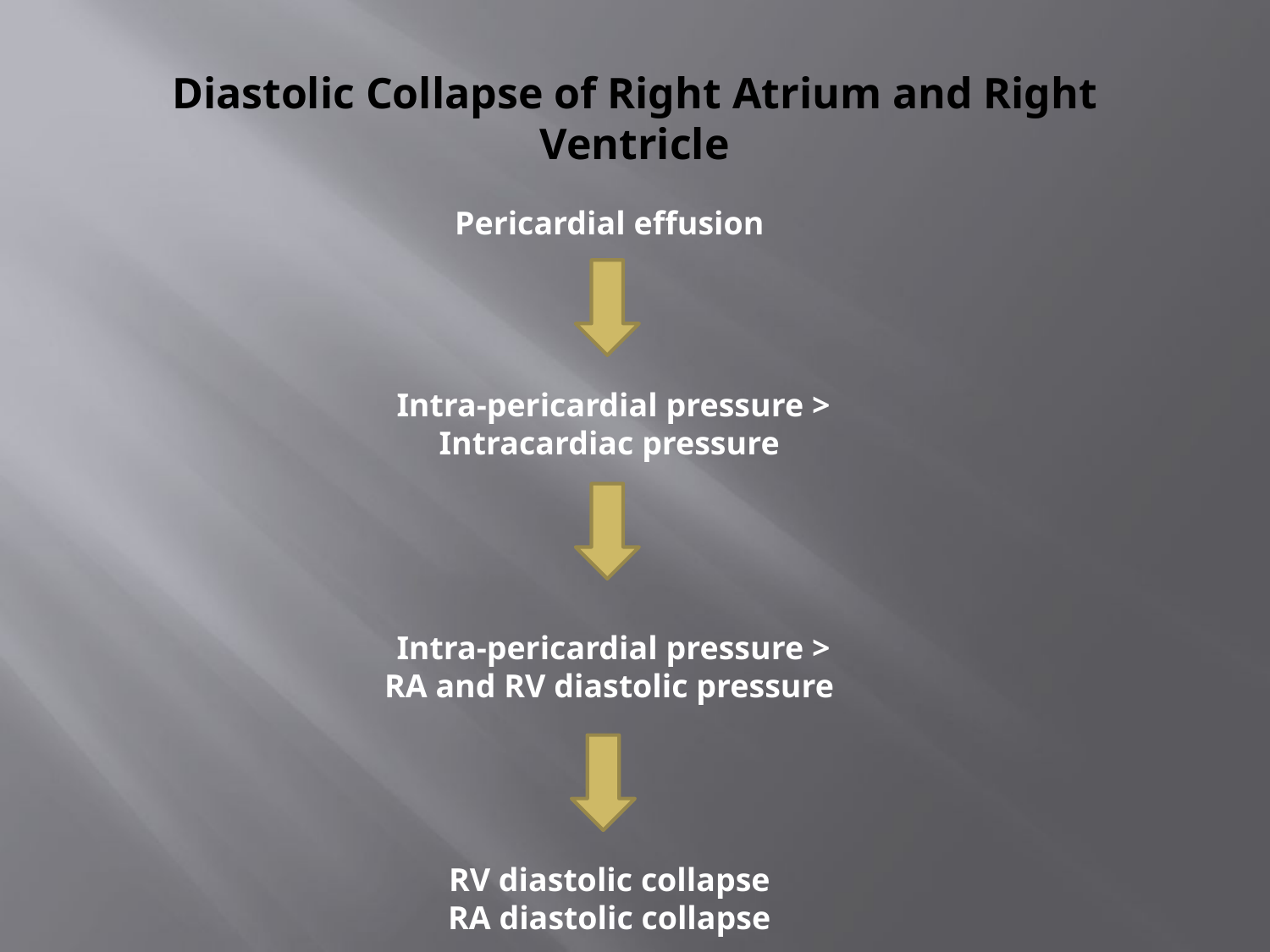

# Diastolic Collapse of Right Atrium and Right Ventricle
Pericardial effusion
Intra-pericardial pressure > Intracardiac pressure
Intra-pericardial pressure > RA and RV diastolic pressure
RV diastolic collapse
RA diastolic collapse

## Slide 34
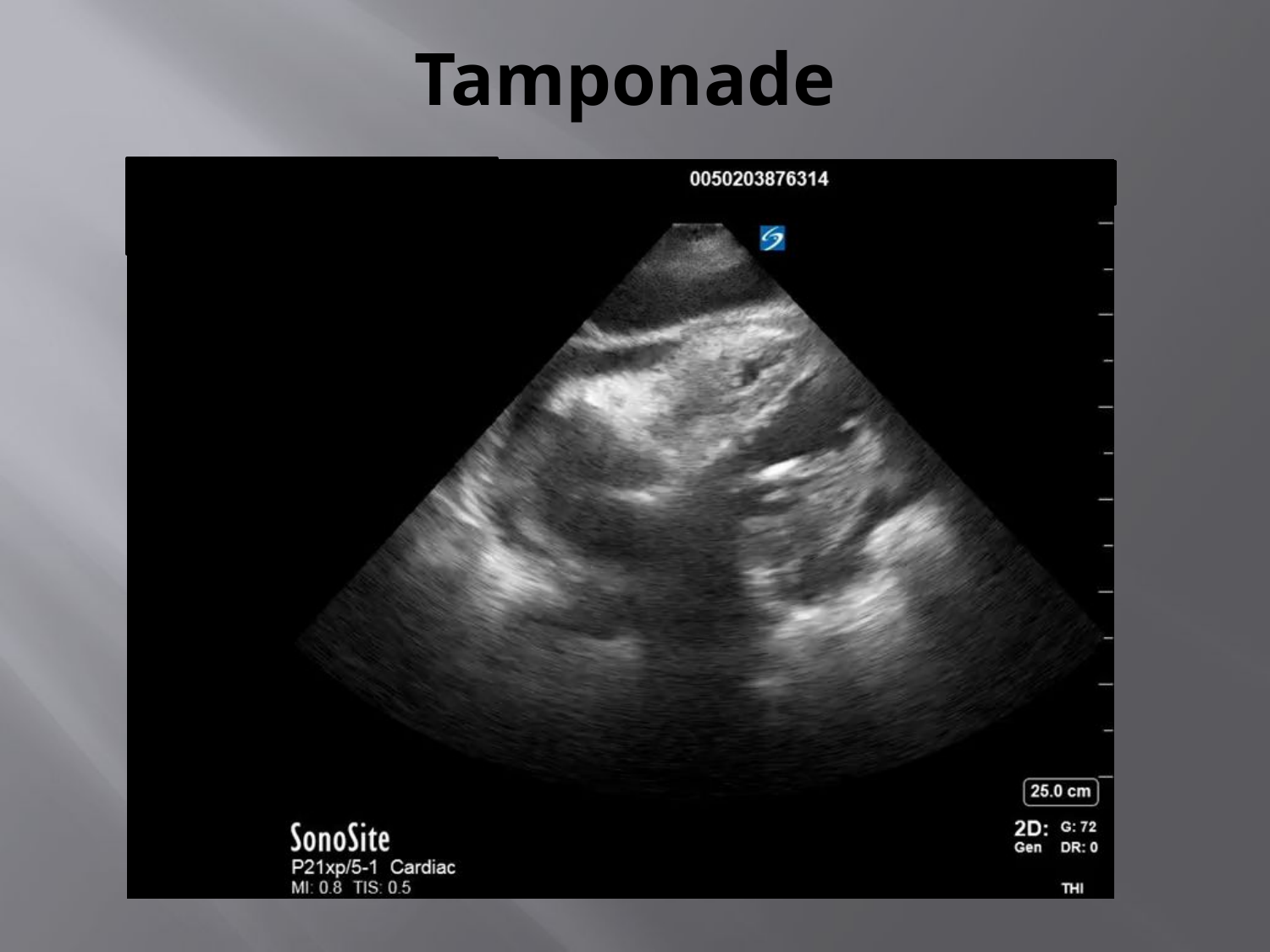

# Tamponade

## Slide 35
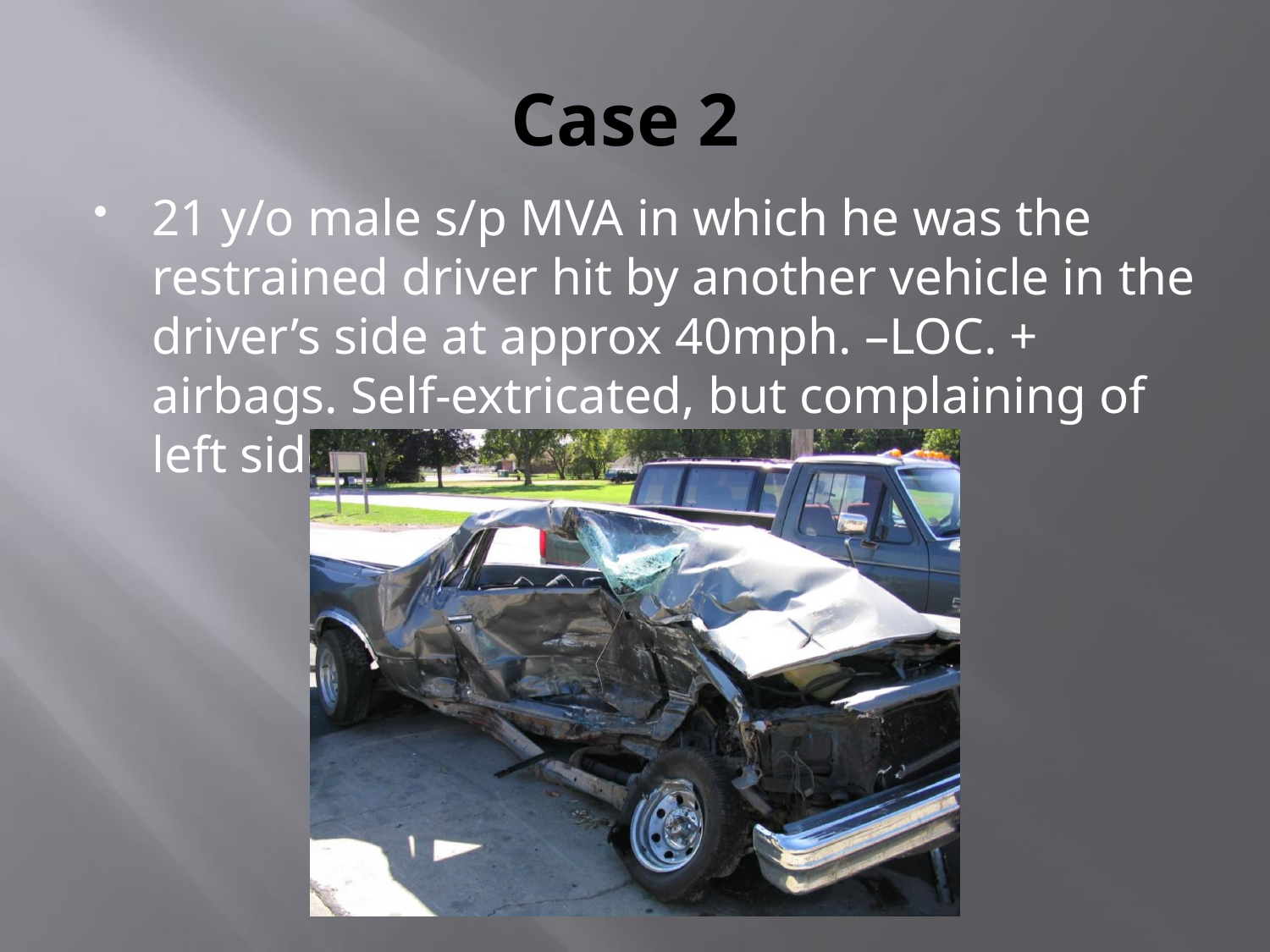

# Case 2
21 y/o male s/p MVA in which he was the restrained driver hit by another vehicle in the driver’s side at approx 40mph. –LOC. + airbags. Self-extricated, but complaining of left side pain.

## Slide 36
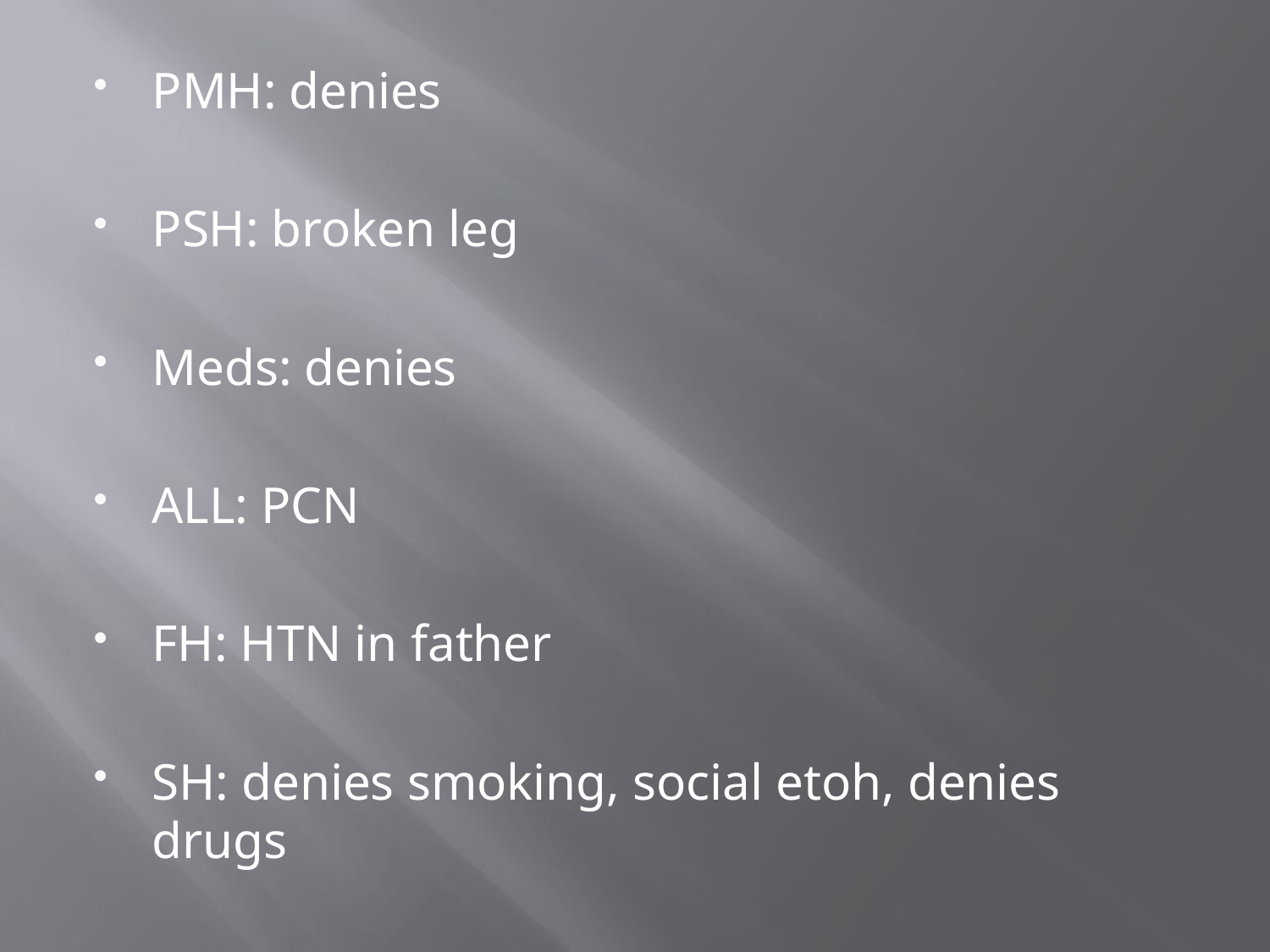

PMH: denies
PSH: broken leg
Meds: denies
ALL: PCN
FH: HTN in father
SH: denies smoking, social etoh, denies drugs

## Slide 37
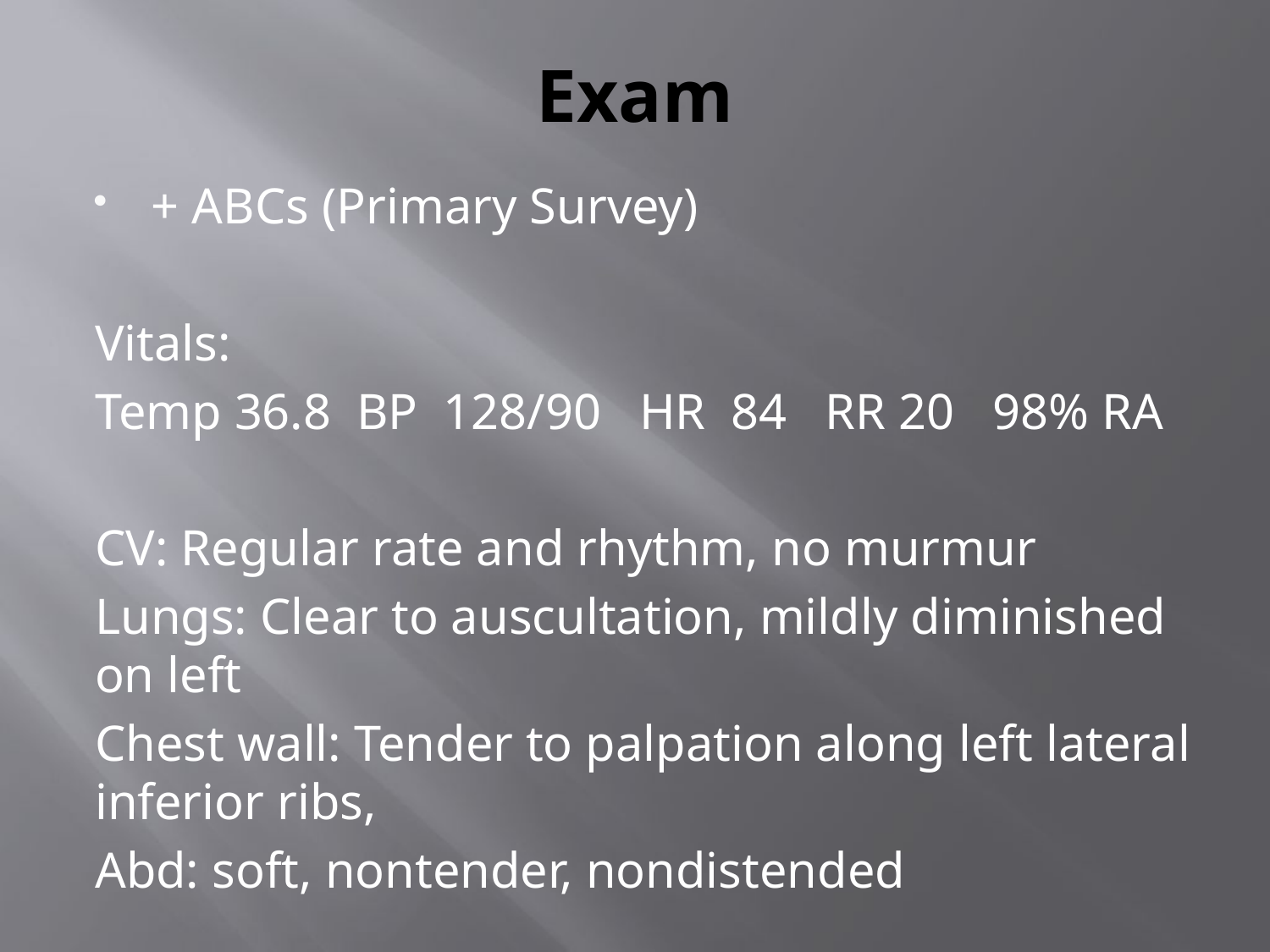

# Exam
+ ABCs (Primary Survey)
Vitals:
Temp 36.8 BP 128/90 HR 84 RR 20 98% RA
CV: Regular rate and rhythm, no murmur
Lungs: Clear to auscultation, mildly diminished on left
Chest wall: Tender to palpation along left lateral inferior ribs,
Abd: soft, nontender, nondistended

## Slide 38
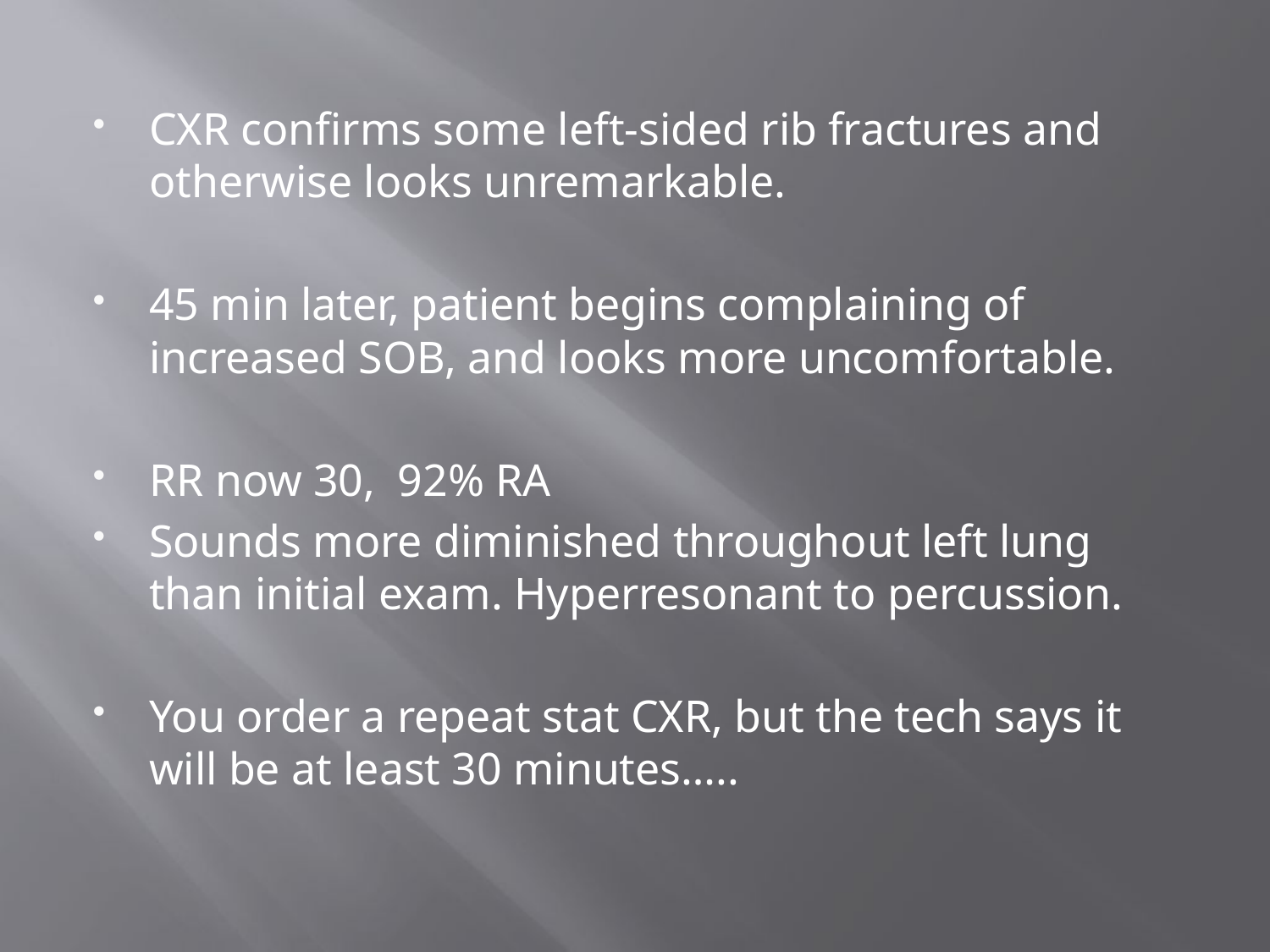

CXR confirms some left-sided rib fractures and otherwise looks unremarkable.
45 min later, patient begins complaining of increased SOB, and looks more uncomfortable.
RR now 30, 92% RA
Sounds more diminished throughout left lung than initial exam. Hyperresonant to percussion.
You order a repeat stat CXR, but the tech says it will be at least 30 minutes…..

## Slide 39
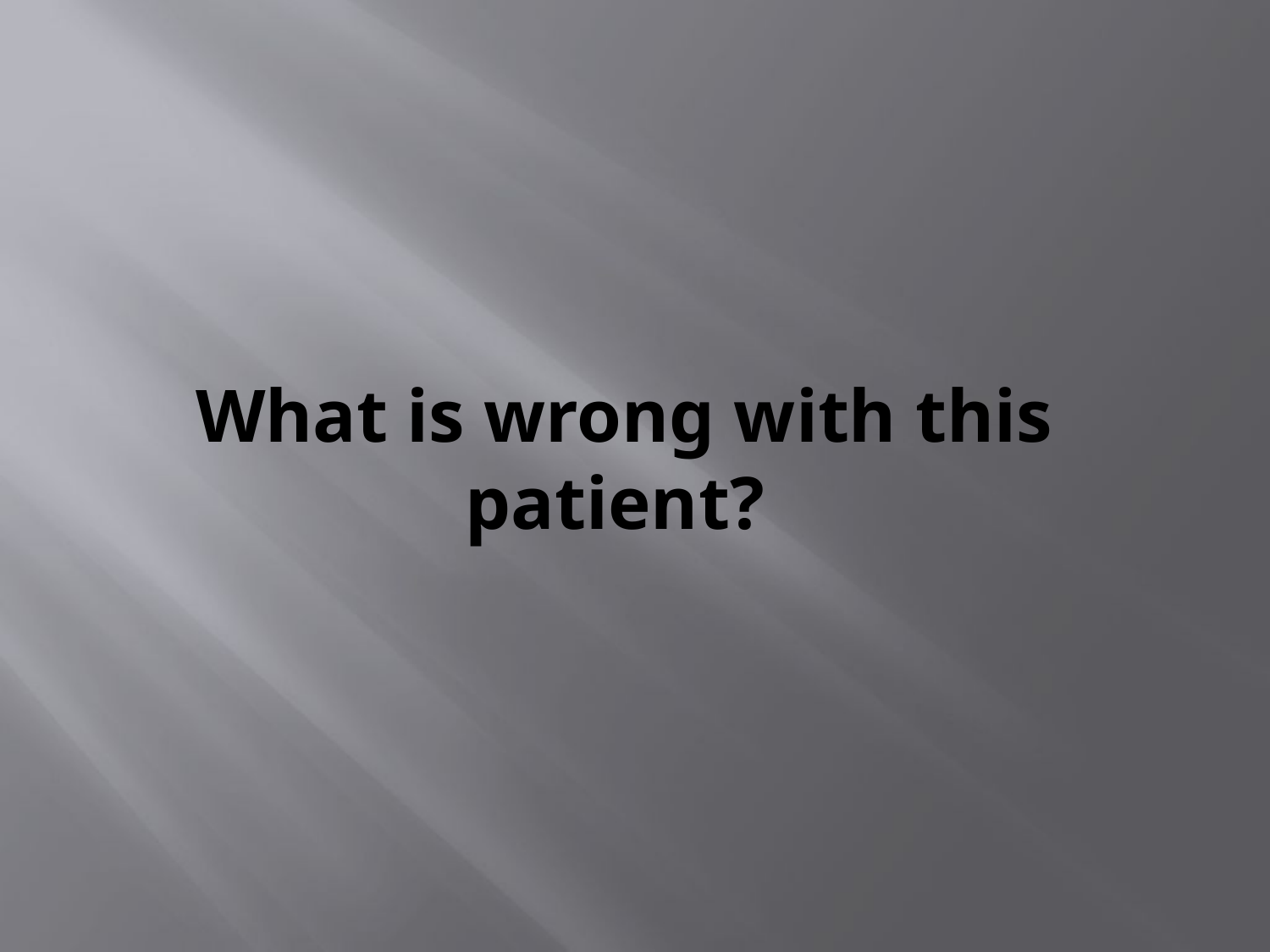

# What is wrong with this patient?

## Slide 40
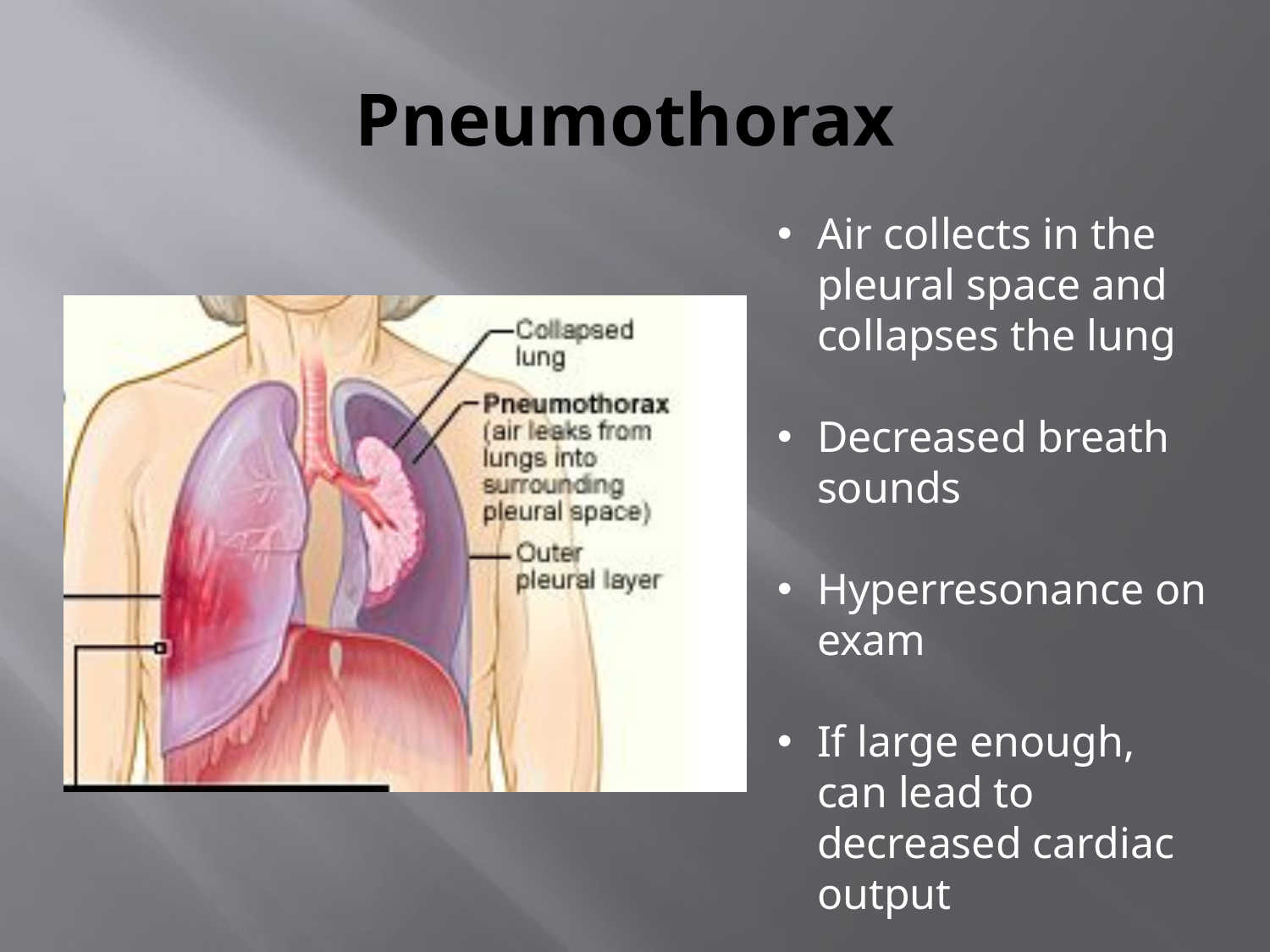

# Pneumothorax
Air collects in the pleural space and collapses the lung
Decreased breath sounds
Hyperresonance on exam
If large enough, can lead to decreased cardiac output

## Slide 41
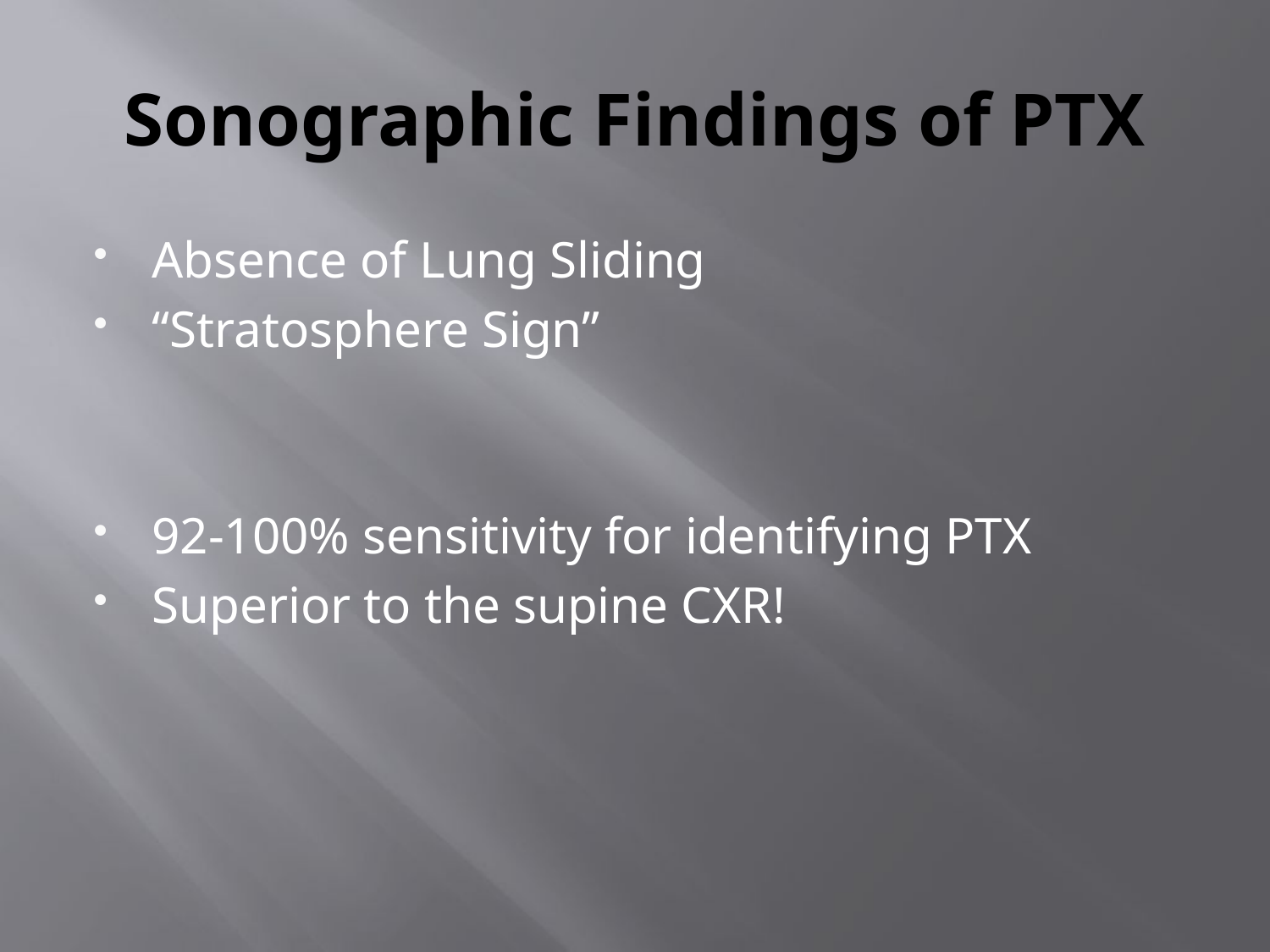

# Sonographic Findings of PTX
Absence of Lung Sliding
“Stratosphere Sign”
92-100% sensitivity for identifying PTX
Superior to the supine CXR!

## Slide 42
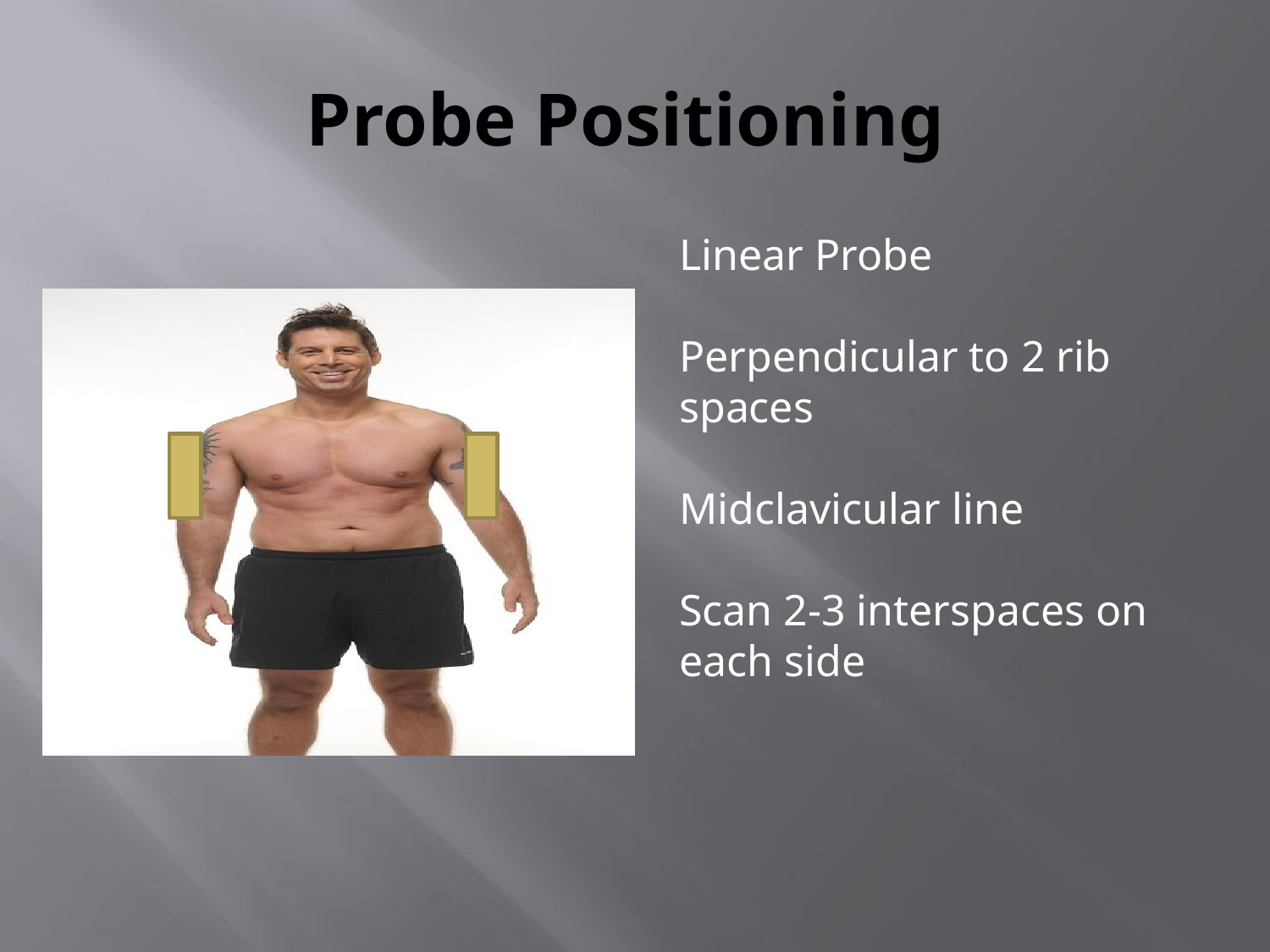

# Probe Positioning
Linear Probe
Perpendicular to 2 rib spaces
Midclavicular line
Scan 2-3 interspaces on each side

## Slide 43
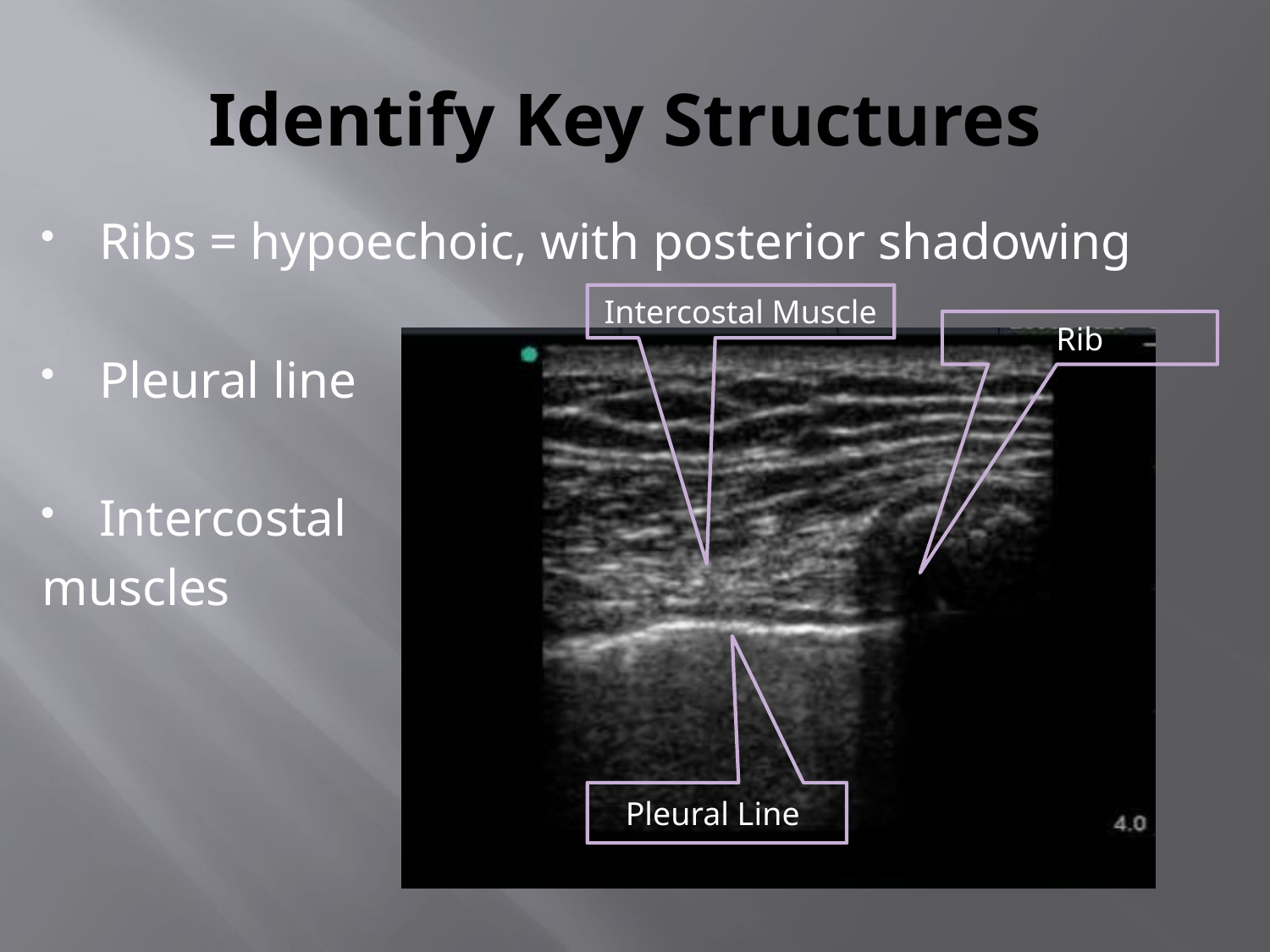

# Identify Key Structures
Ribs = hypoechoic, with posterior shadowing
Pleural line
Intercostal
muscles
Intercostal Muscle
Rib
Pleural Line

## Slide 44
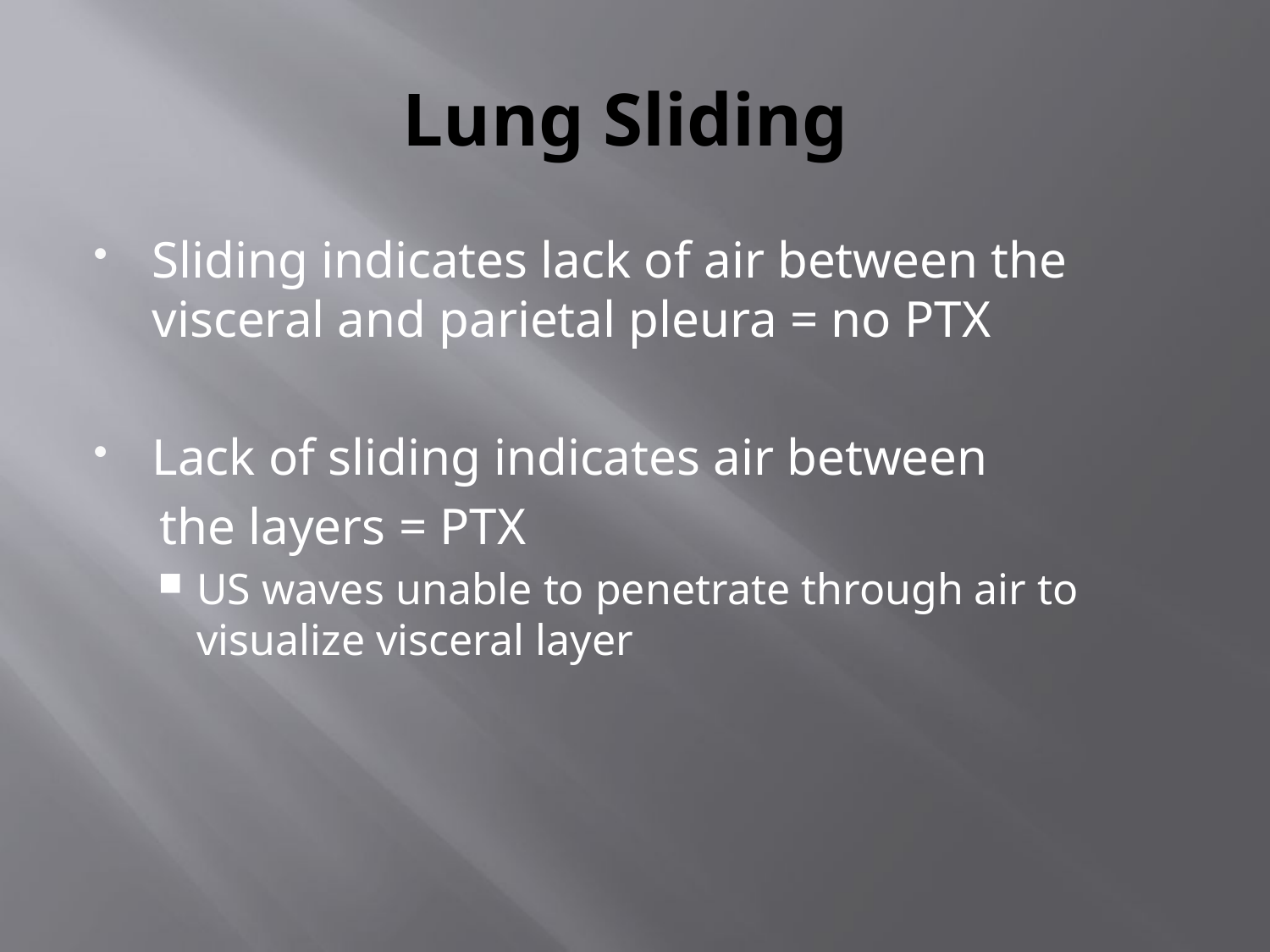

# Lung Sliding
Sliding indicates lack of air between the visceral and parietal pleura = no PTX
Lack of sliding indicates air between
 the layers = PTX
US waves unable to penetrate through air to visualize visceral layer

## Slide 45
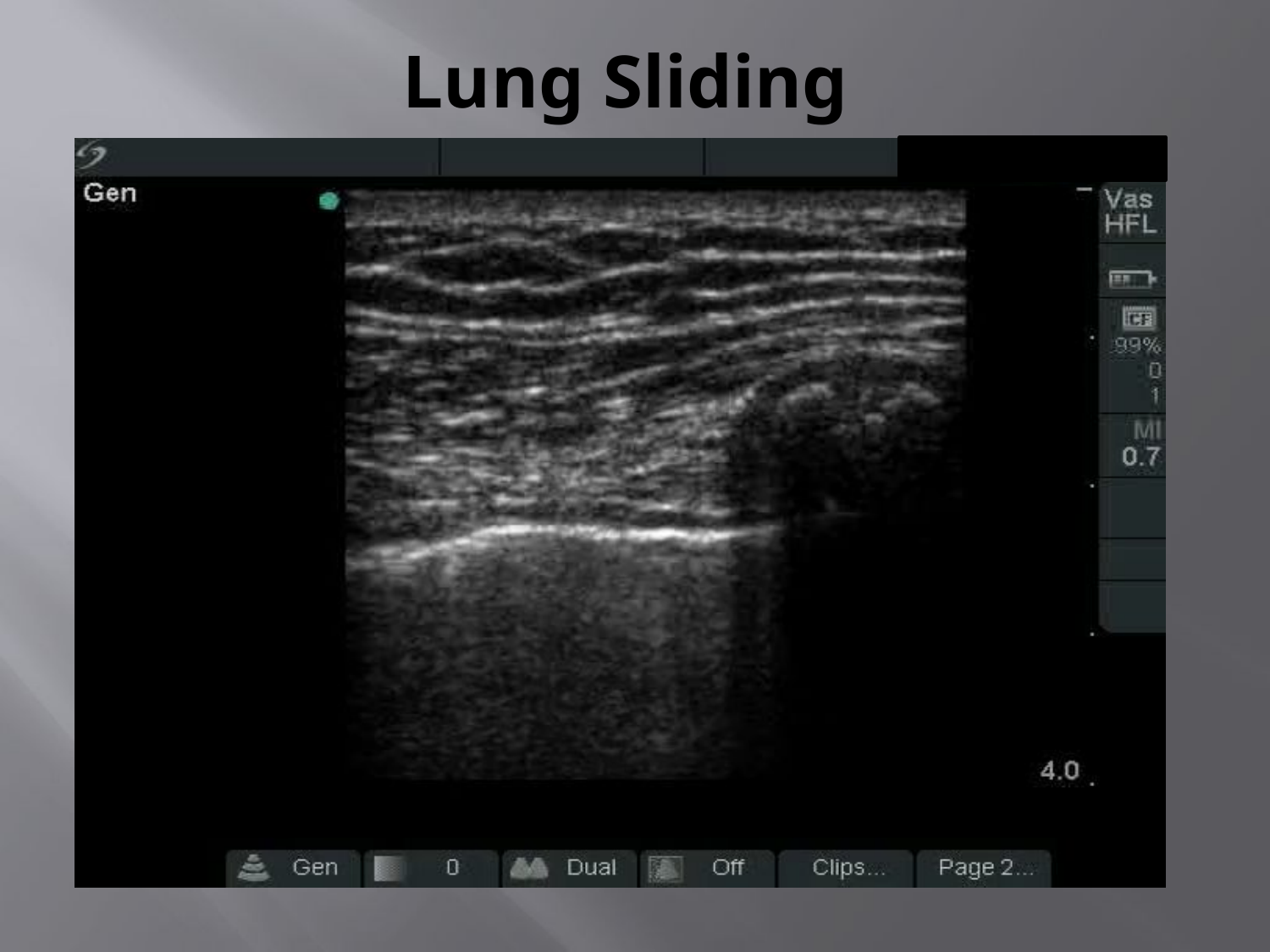

# Lung Sliding

## Slide 46
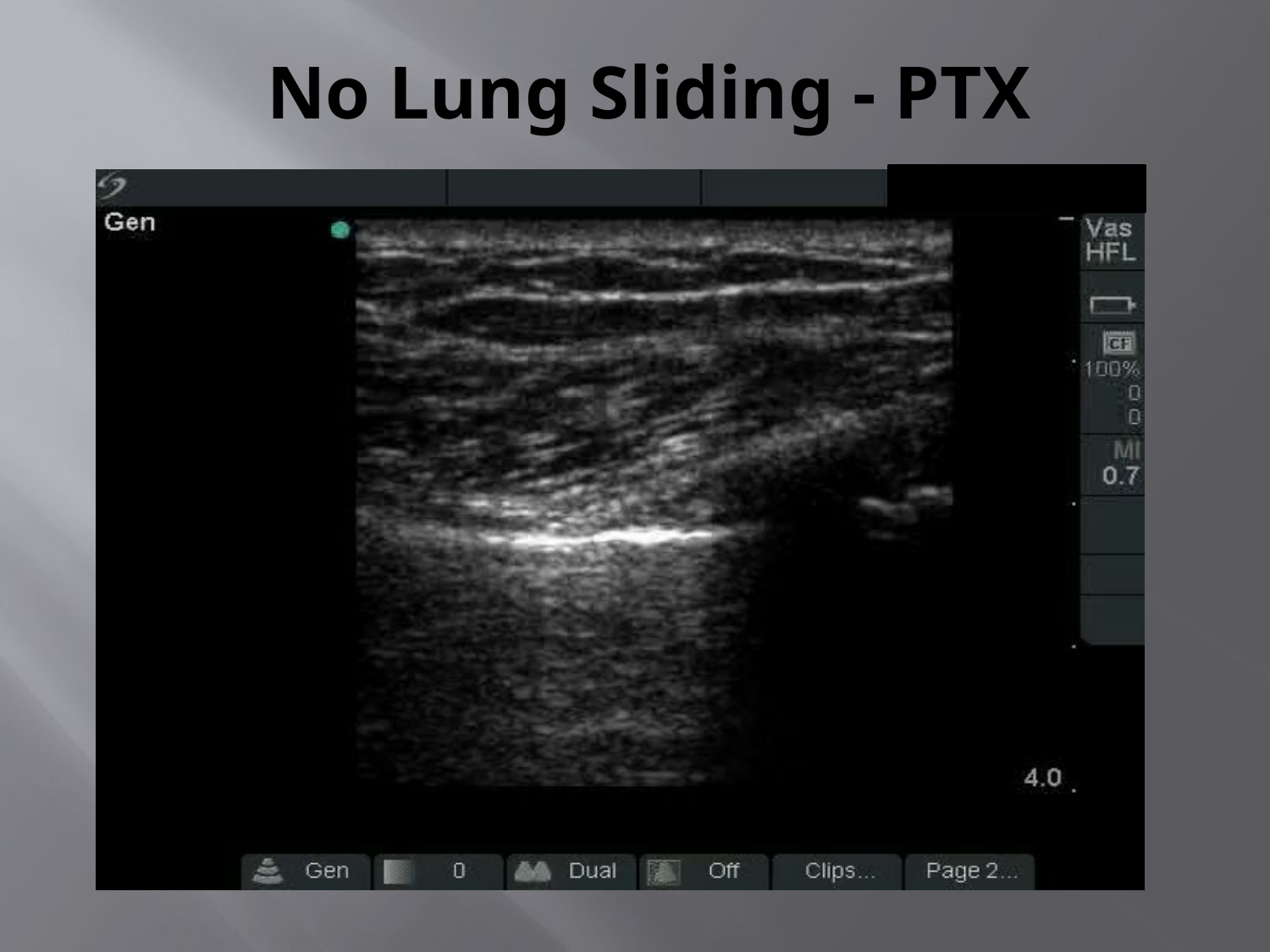

# No Lung Sliding - PTX

## Slide 47
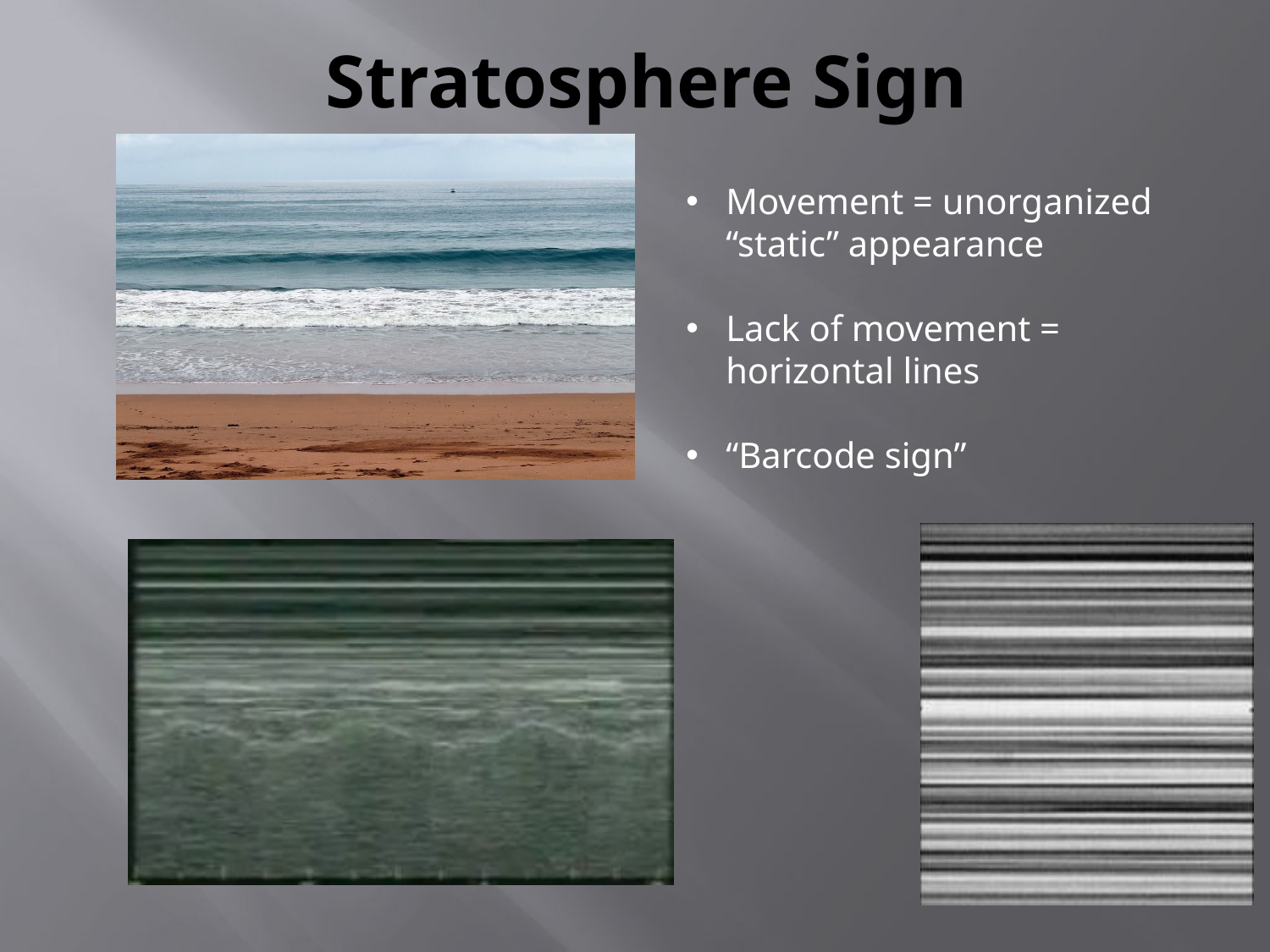

# Stratosphere Sign
Movement = unorganized “static” appearance
Lack of movement = horizontal lines
“Barcode sign”

## Slide 48
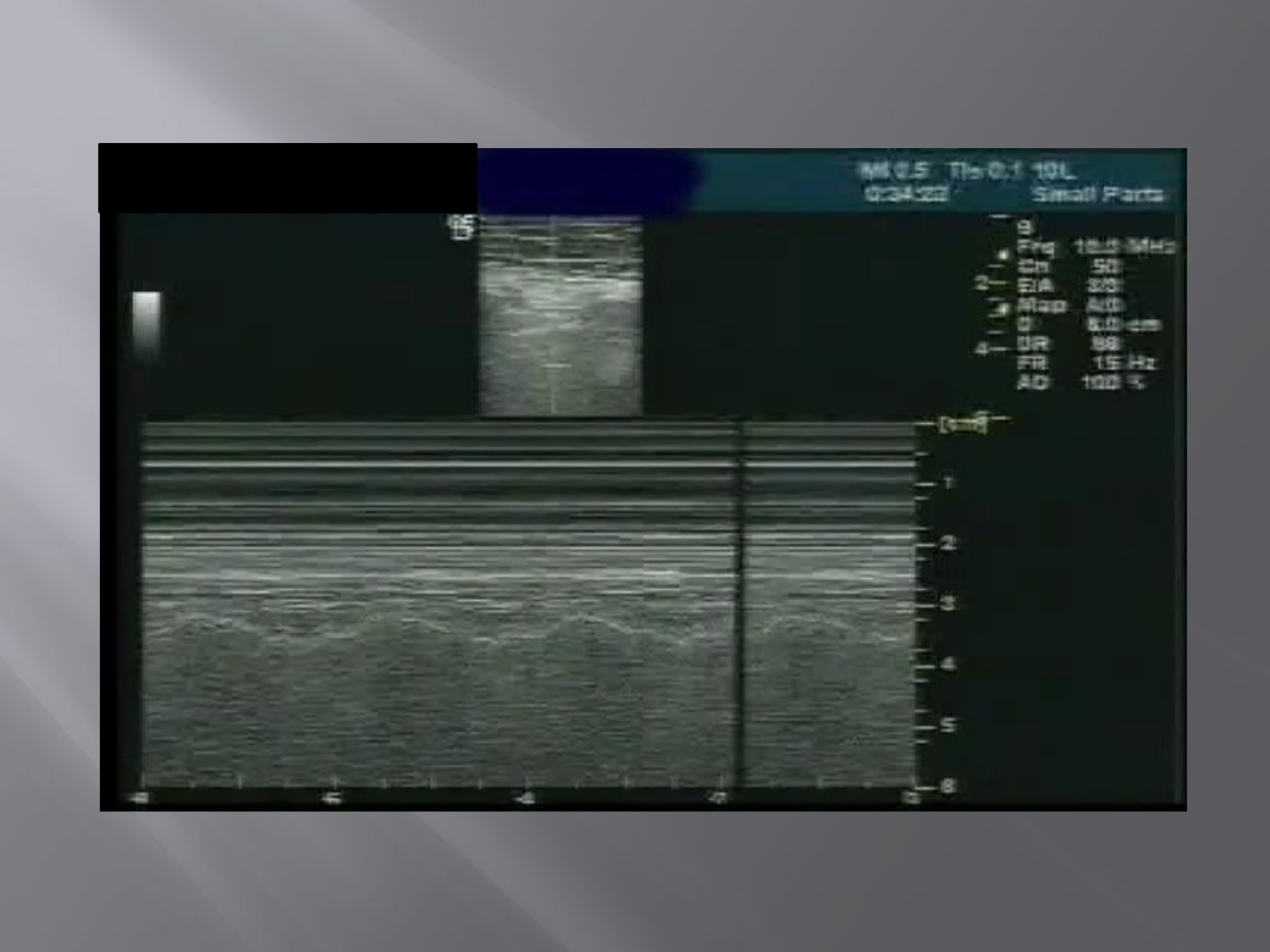

## Slide 49
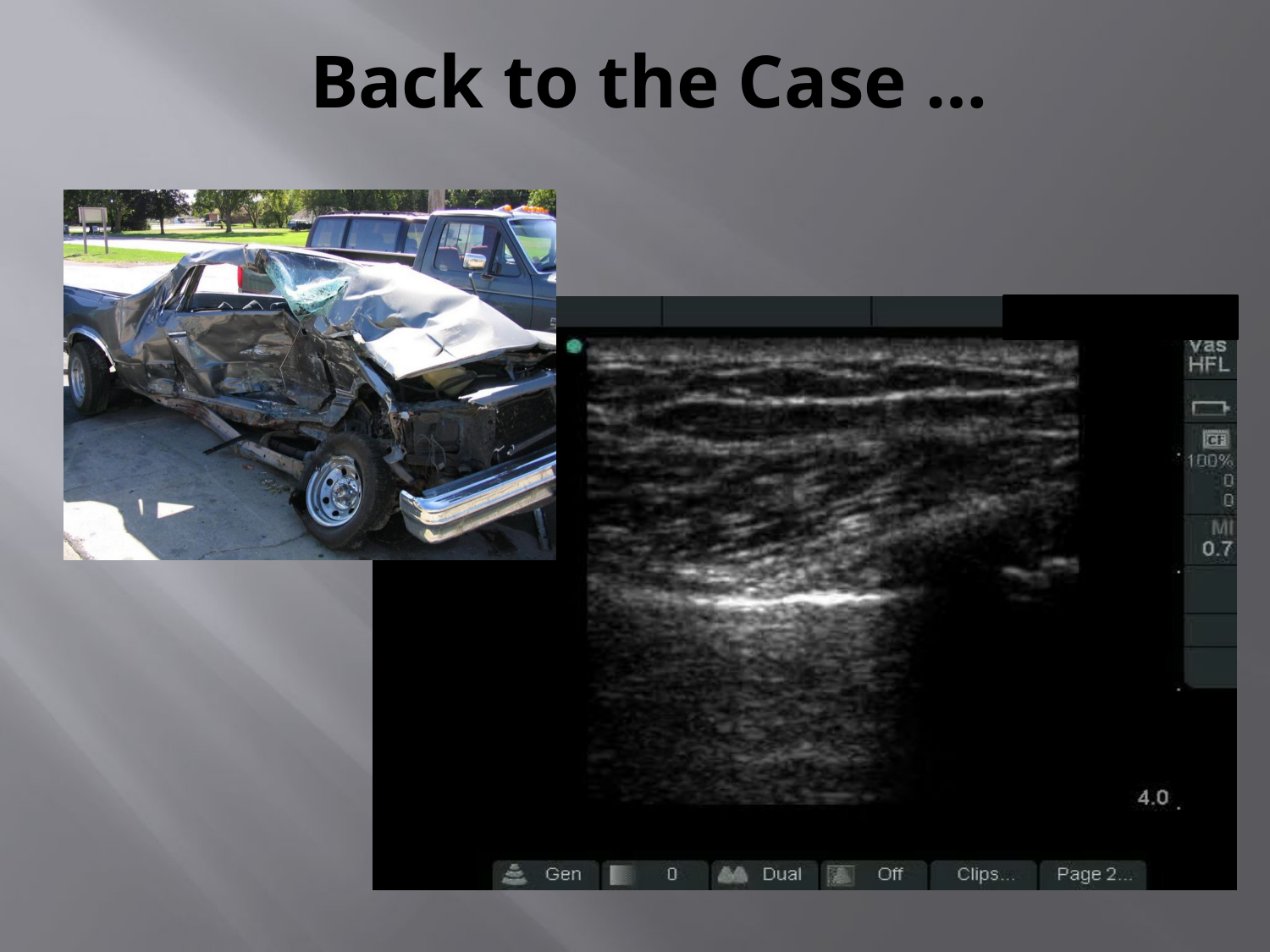

# Back to the Case …

## Slide 50
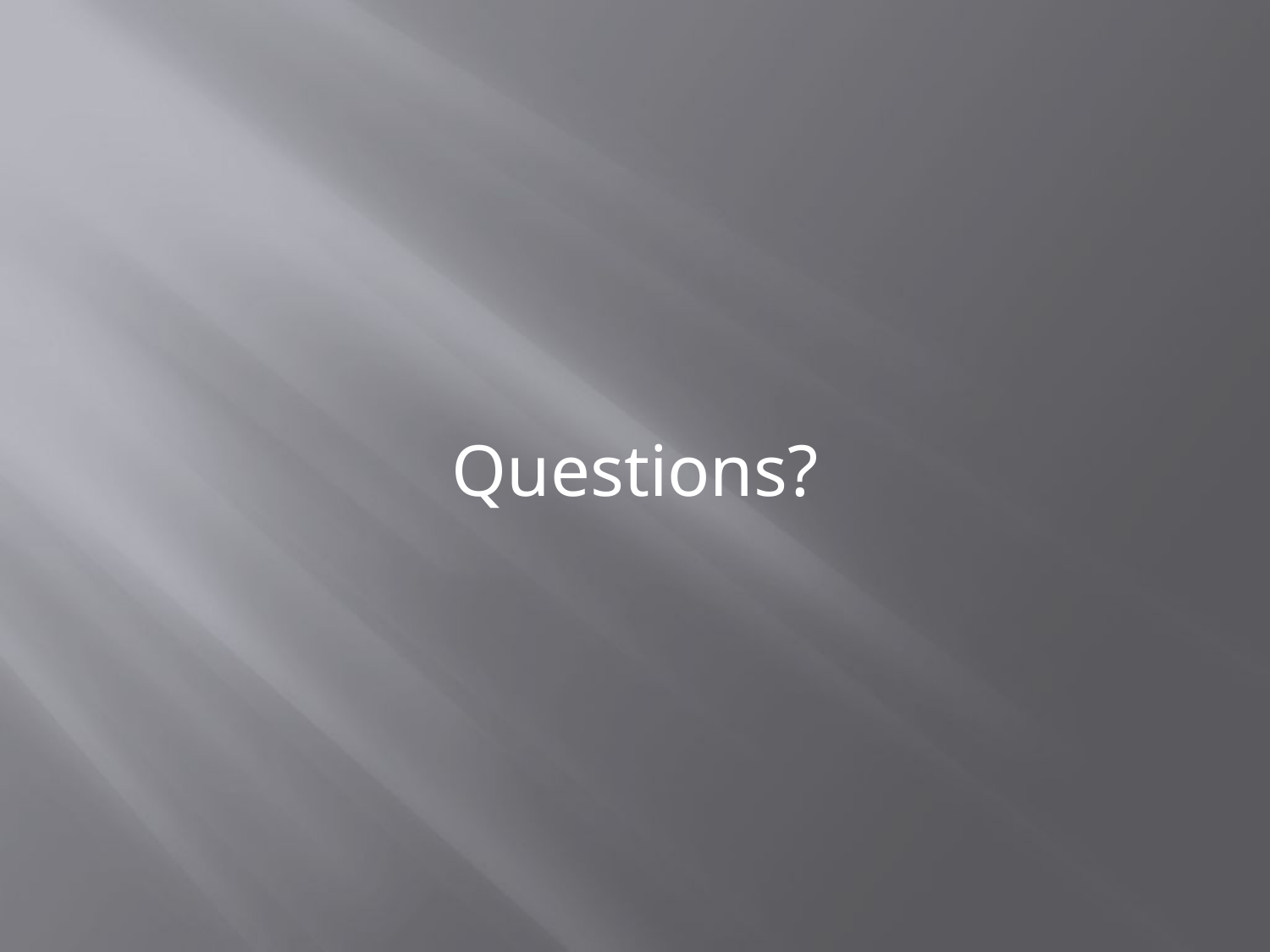

Questions?

## Slide 51
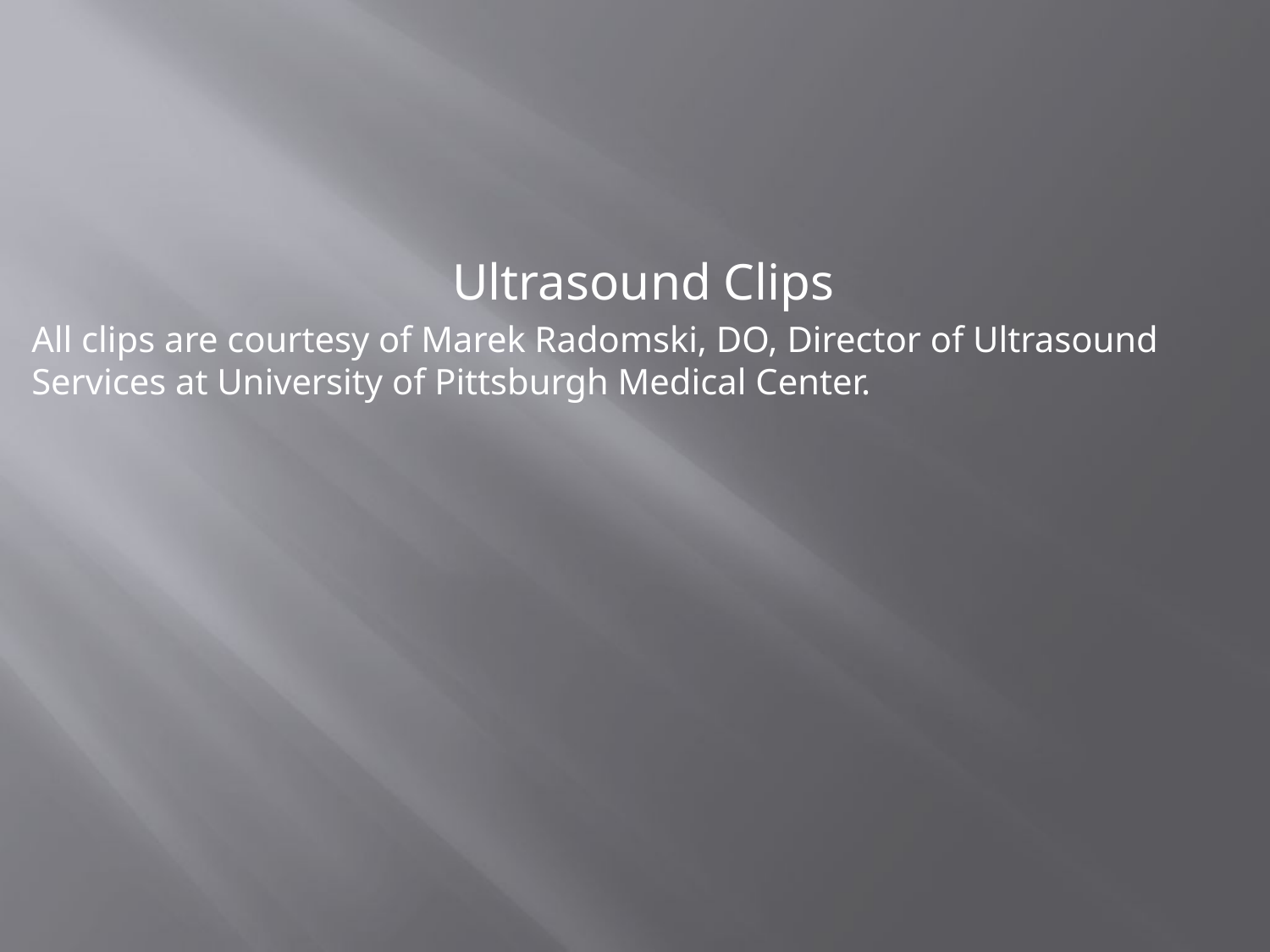

Ultrasound Clips
All clips are courtesy of Marek Radomski, DO, Director of Ultrasound Services at University of Pittsburgh Medical Center.
#
