# Supplementary material for: Use of Ultrasound in Introducing Anatomical Pathology to Preclinical Medical Students, in Correlation with Physical Exam Curricula
Source: MedEdPORTAL. 2020 Sep 25;16:10950. doi: 10.15766/mep_2374-8265.10950 (PMC7521063; doi:10.15766/mep_2374-8265.10950)
Supplement: Supplementary file 1 — Session 1 FAST Exam & the Trauma Patient.pptxSession 2 Cardiac and Lung.pptxSession 3 Gallbladder, Kidneys, & AAA.pptxSession 4 Ocular US & Central Access.pptxSession 1 Instructor Script.docxSession 2 Instructor Script.docxSession 3 Instructor Script.docxSession 4 Instructor Script.docxSurvey Questions.docx [file mep_2374-8265.10950-s001.zip › C. Session 3 Gallbladder, Kidneys, & AAA.pptx]

## Slide 1
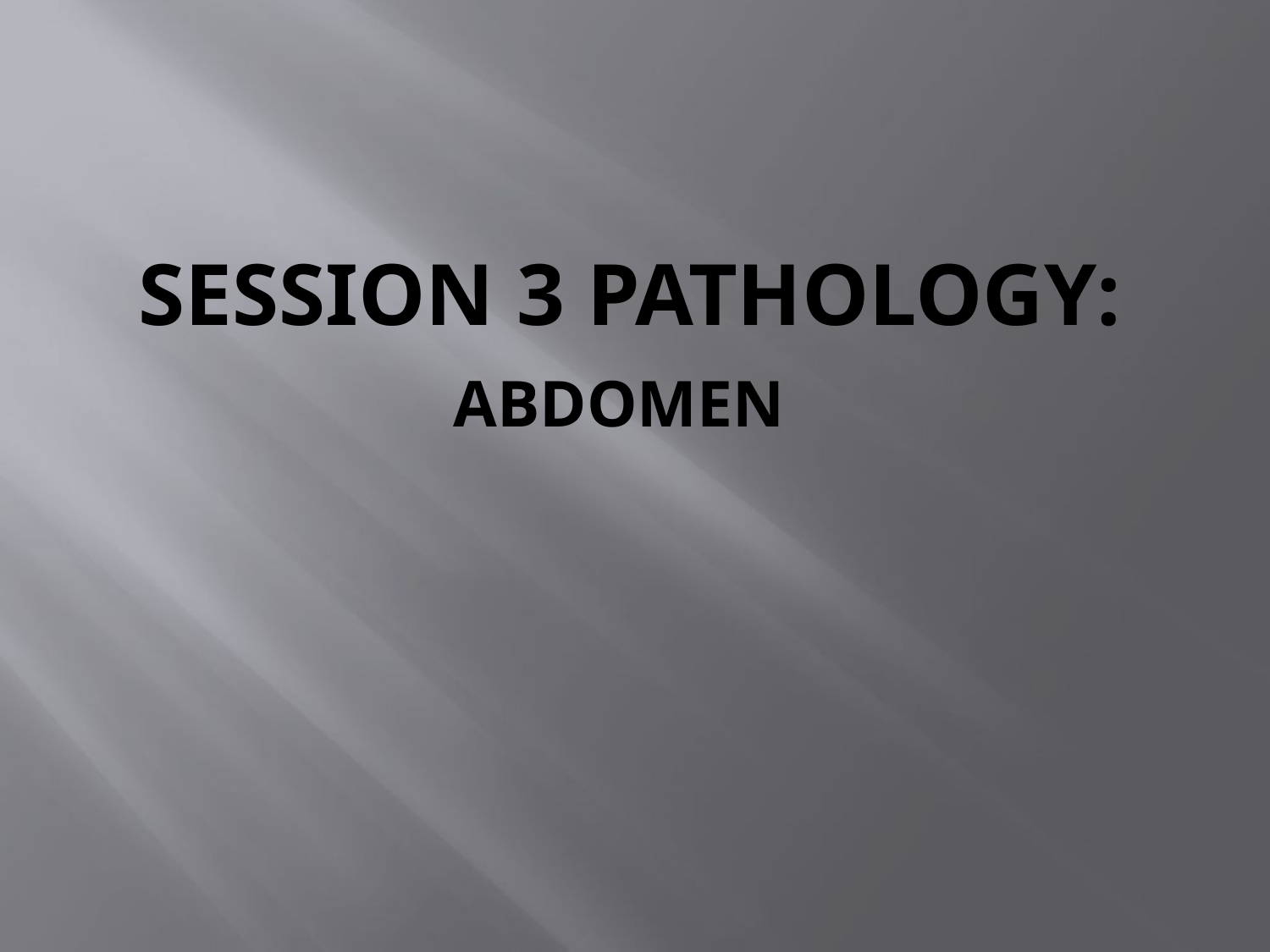

# Session 3 Pathology: Abdomen

## Slide 2
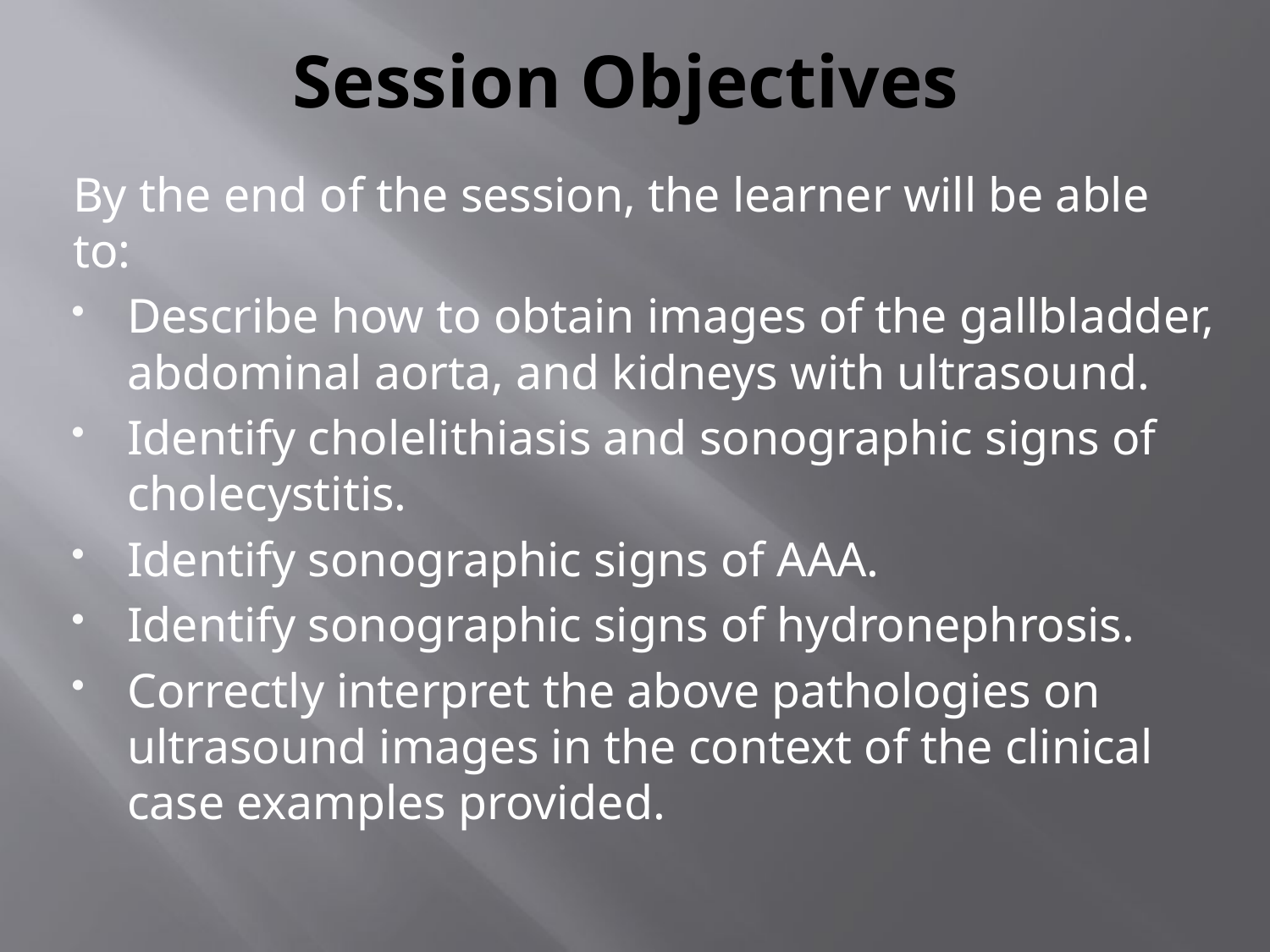

# Session Objectives
By the end of the session, the learner will be able to:
Describe how to obtain images of the gallbladder, abdominal aorta, and kidneys with ultrasound.
Identify cholelithiasis and sonographic signs of cholecystitis.
Identify sonographic signs of AAA.
Identify sonographic signs of hydronephrosis.
Correctly interpret the above pathologies on ultrasound images in the context of the clinical case examples provided.

## Slide 3
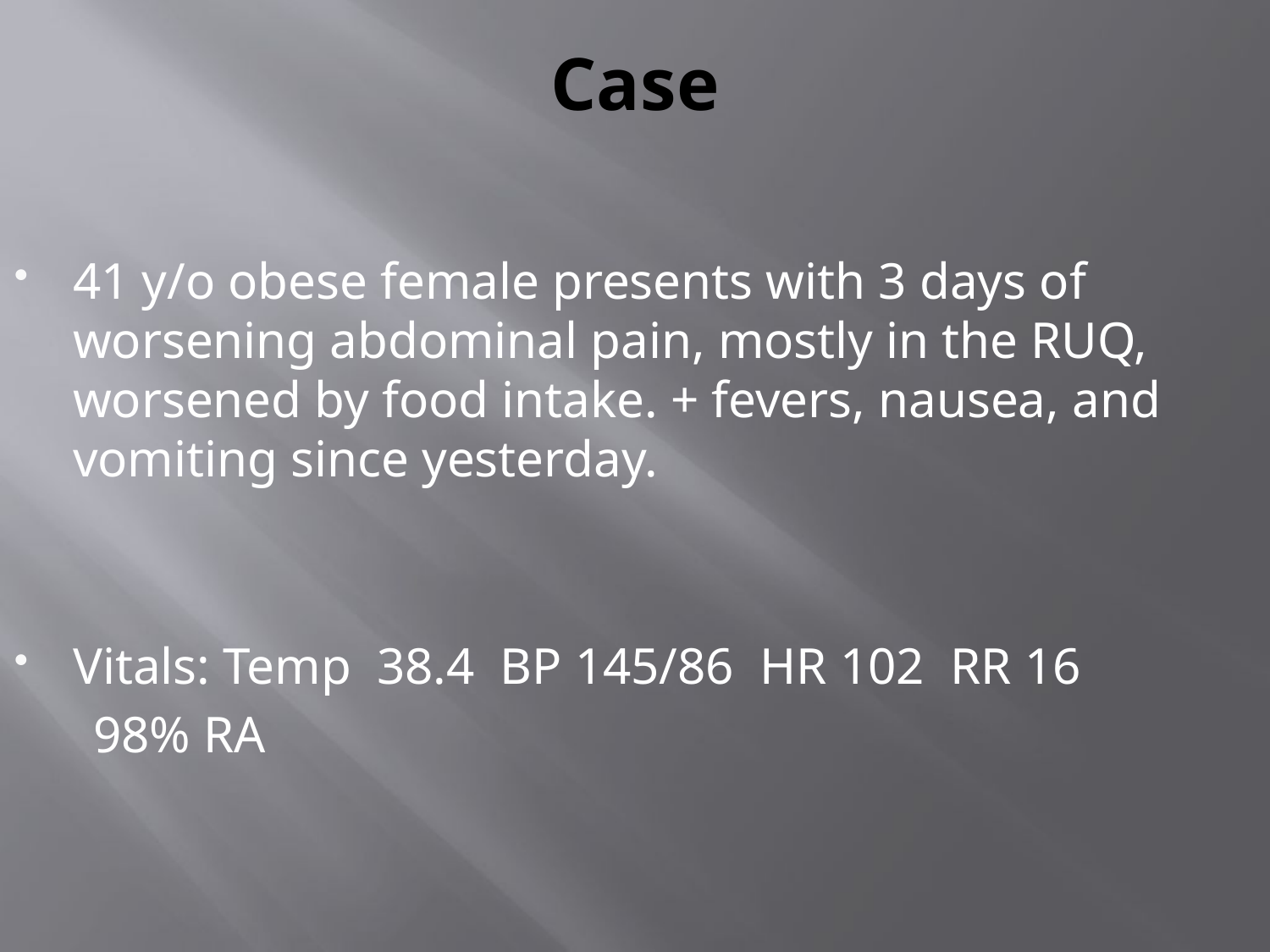

# Case
41 y/o obese female presents with 3 days of worsening abdominal pain, mostly in the RUQ, worsened by food intake. + fevers, nausea, and vomiting since yesterday.
Vitals: Temp 38.4 BP 145/86 HR 102 RR 16
 98% RA

## Slide 4
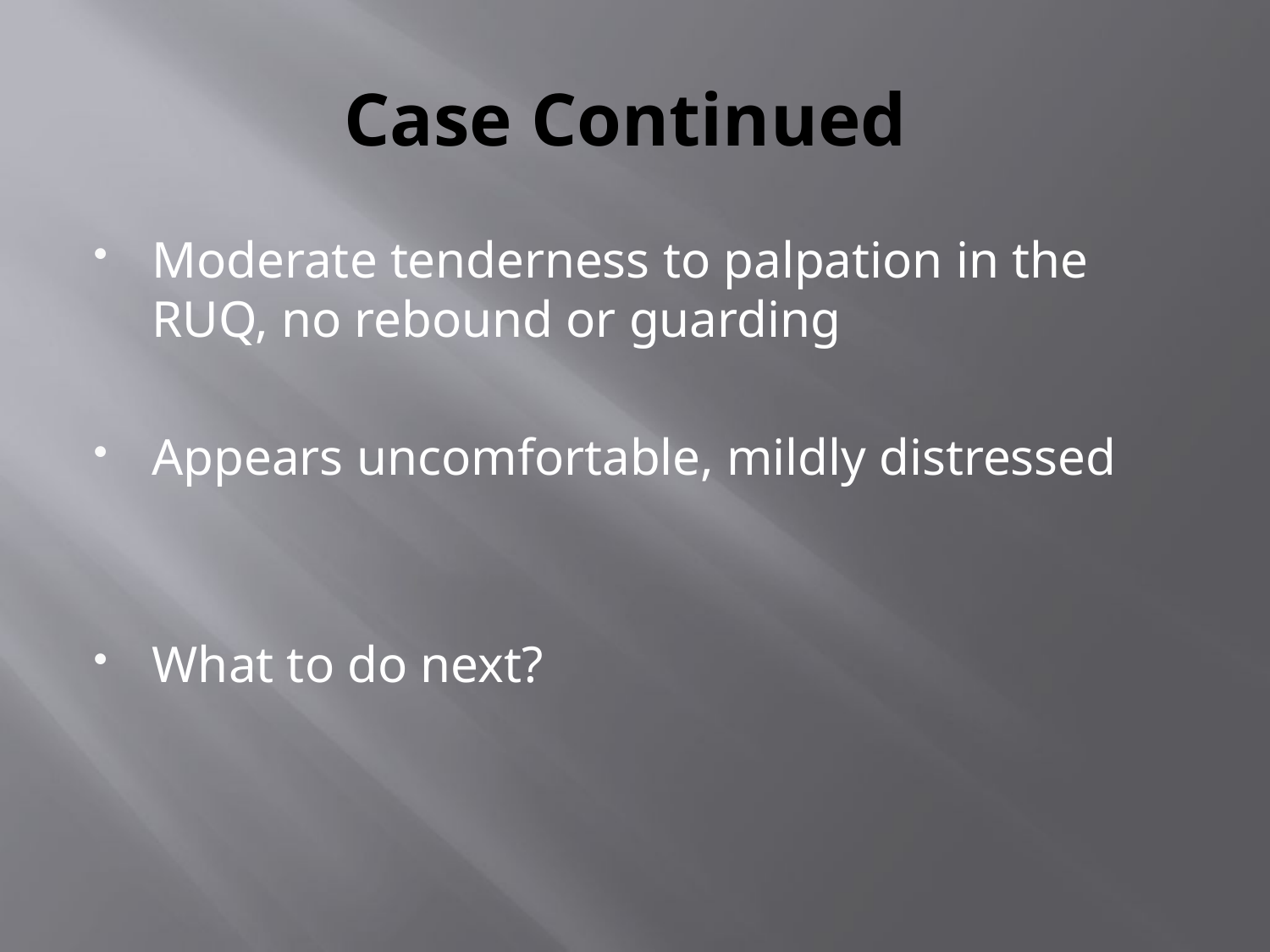

# Case Continued
Moderate tenderness to palpation in the RUQ, no rebound or guarding
Appears uncomfortable, mildly distressed
What to do next?

## Slide 5
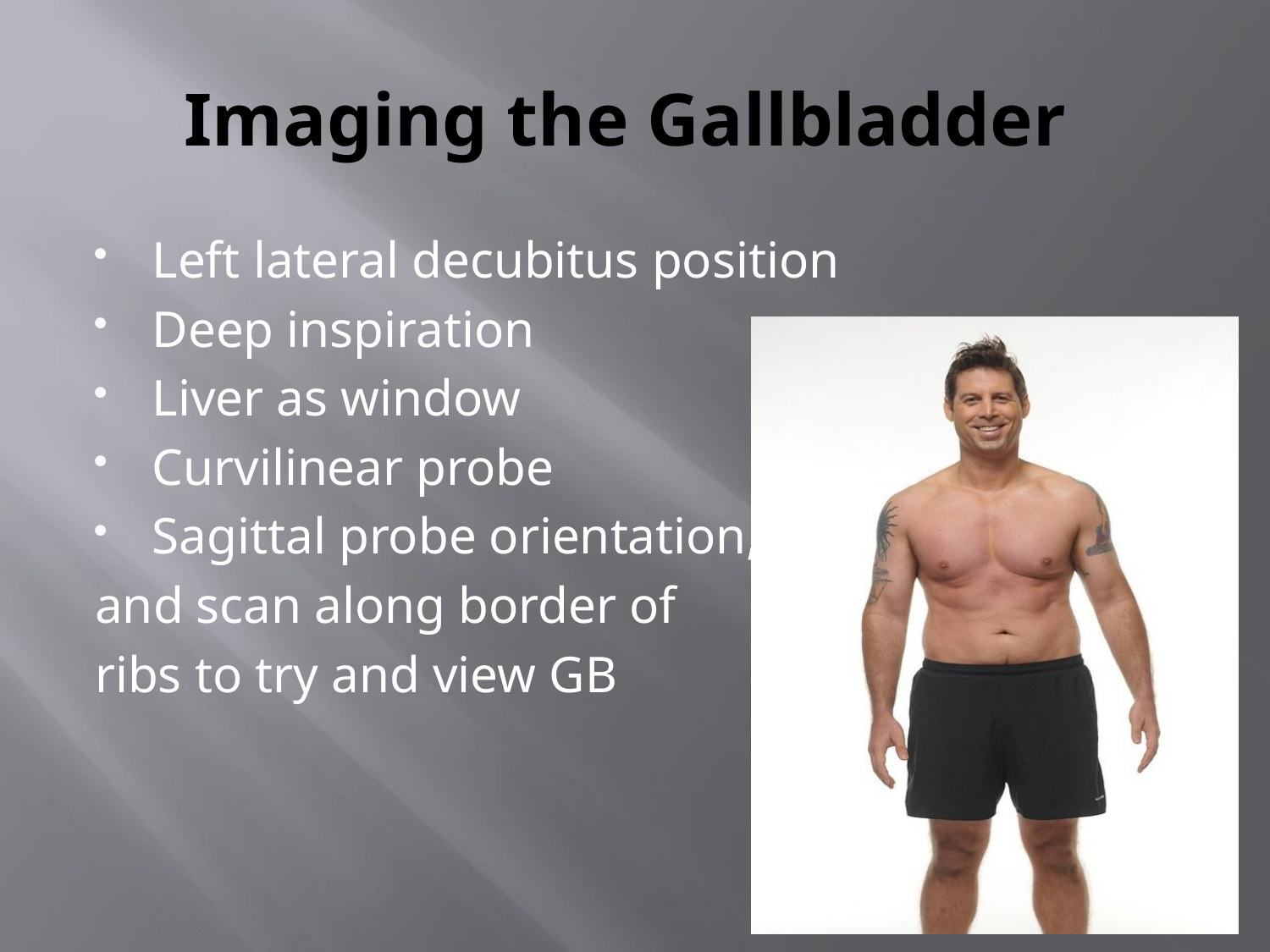

# Imaging the Gallbladder
Left lateral decubitus position
Deep inspiration
Liver as window
Curvilinear probe
Sagittal probe orientation,
and scan along border of
ribs to try and view GB

## Slide 6
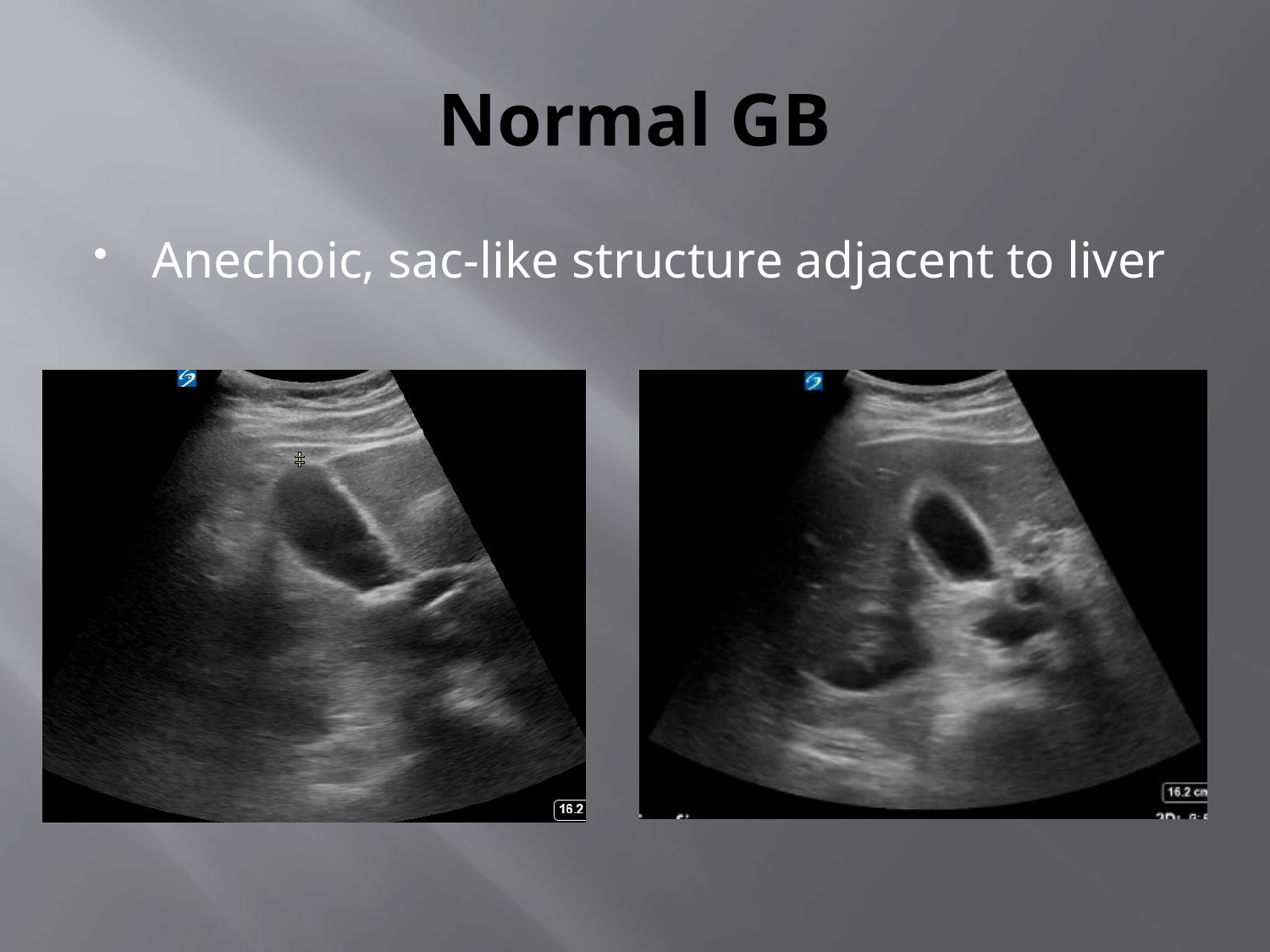

# Normal GB
Anechoic, sac-like structure adjacent to liver

## Slide 7
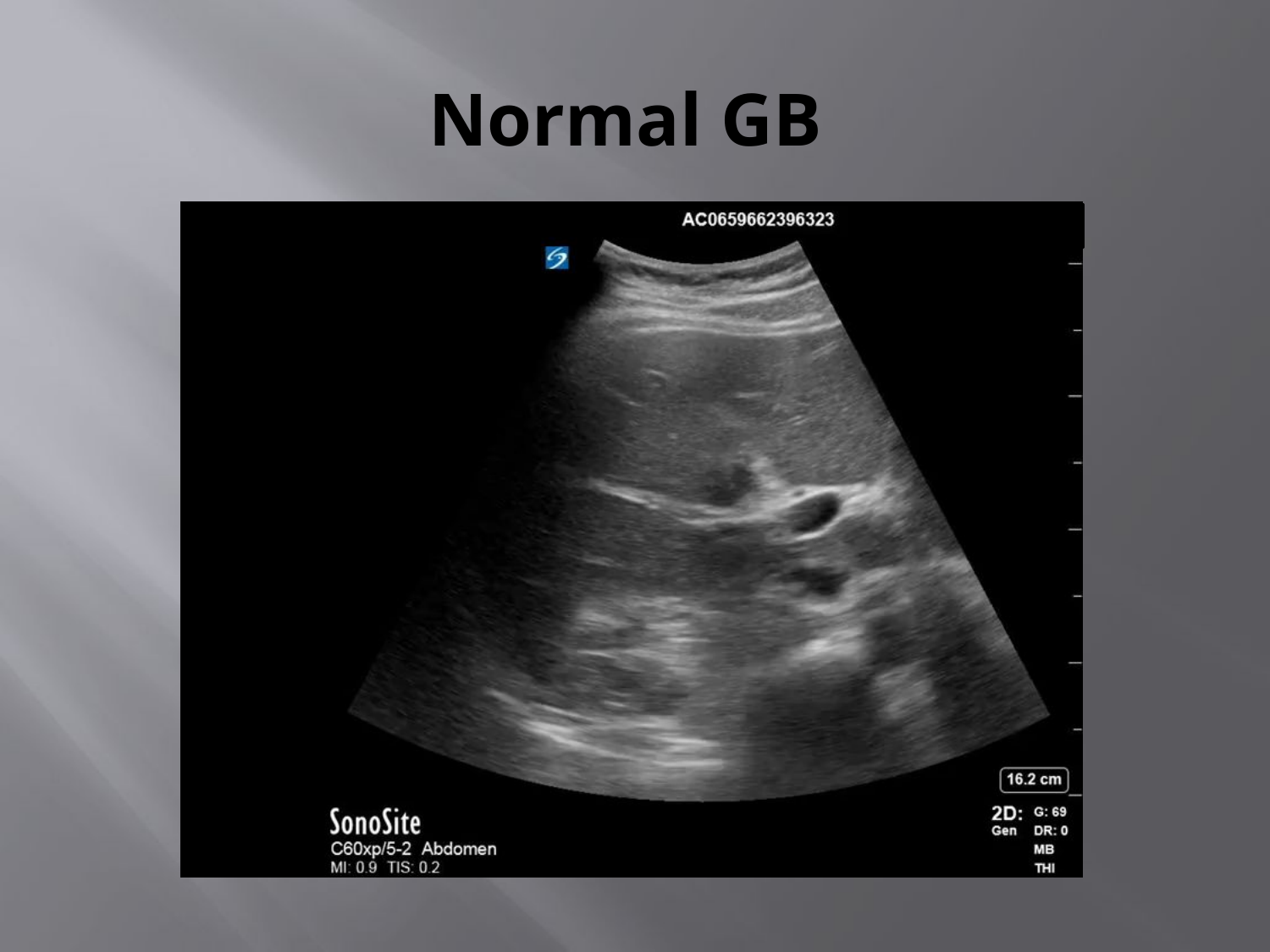

# Normal GB

## Slide 8
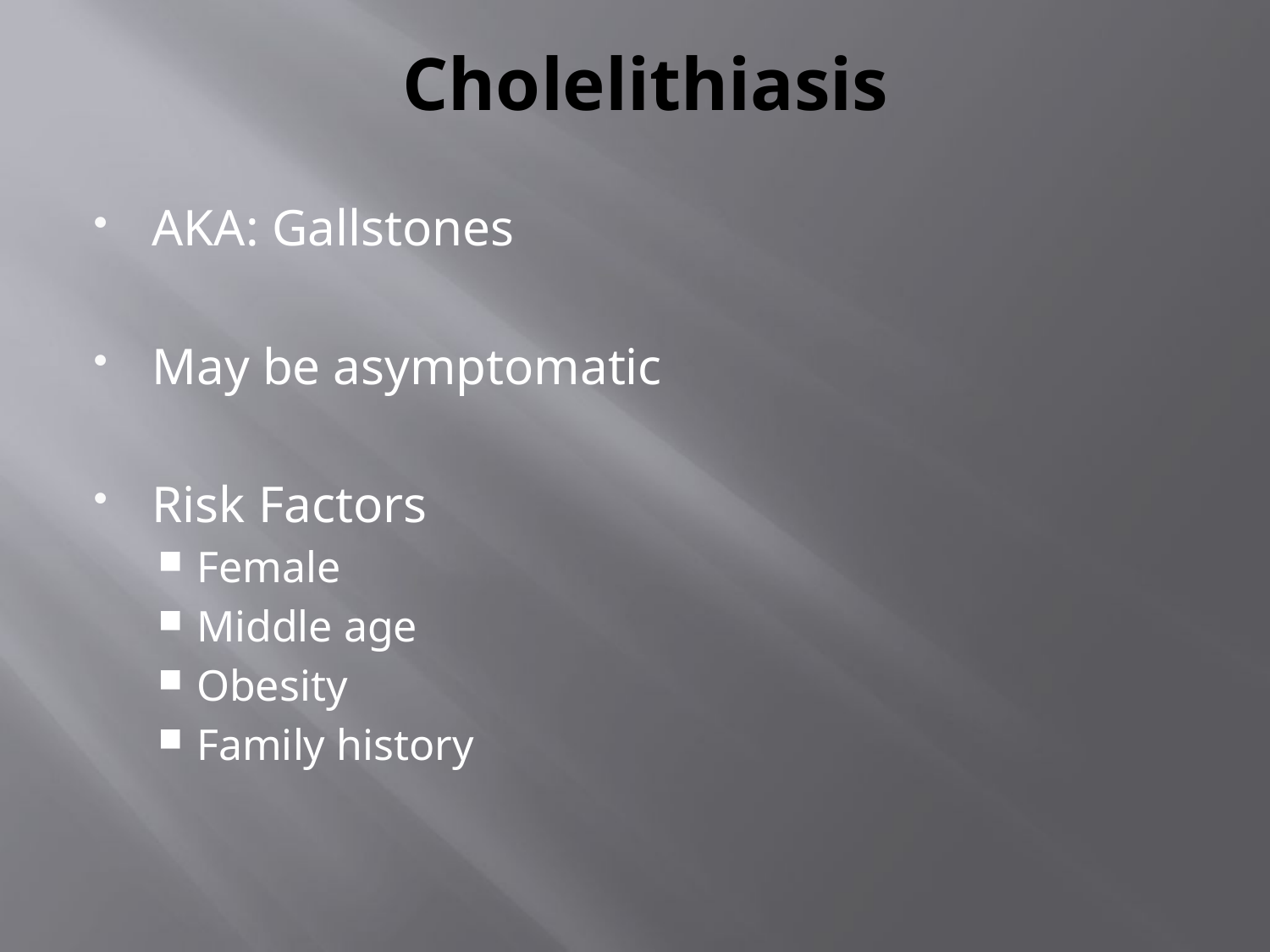

# Cholelithiasis
AKA: Gallstones
May be asymptomatic
Risk Factors
Female
Middle age
Obesity
Family history

## Slide 9
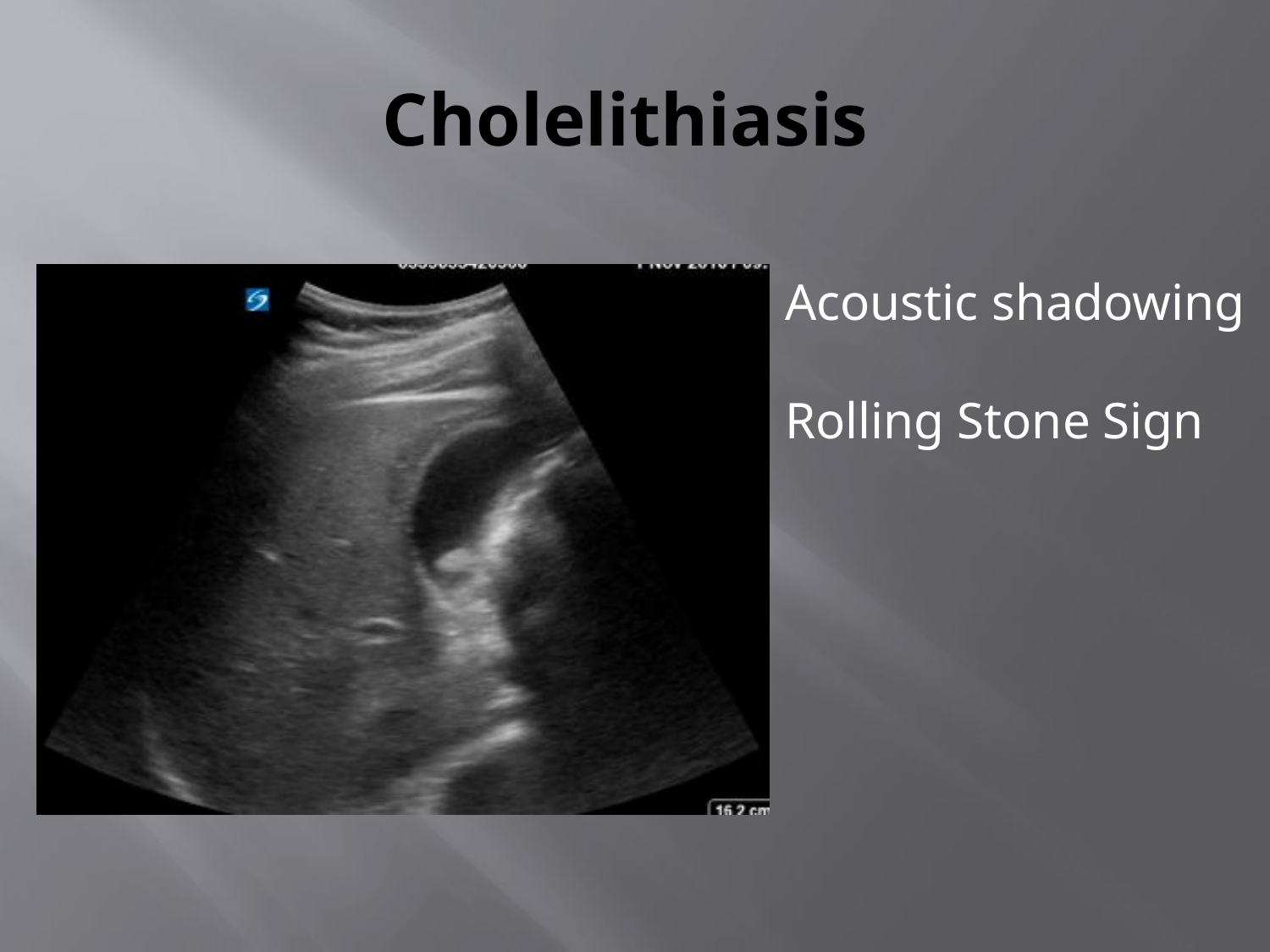

# Cholelithiasis
Acoustic shadowing
Rolling Stone Sign

## Slide 10
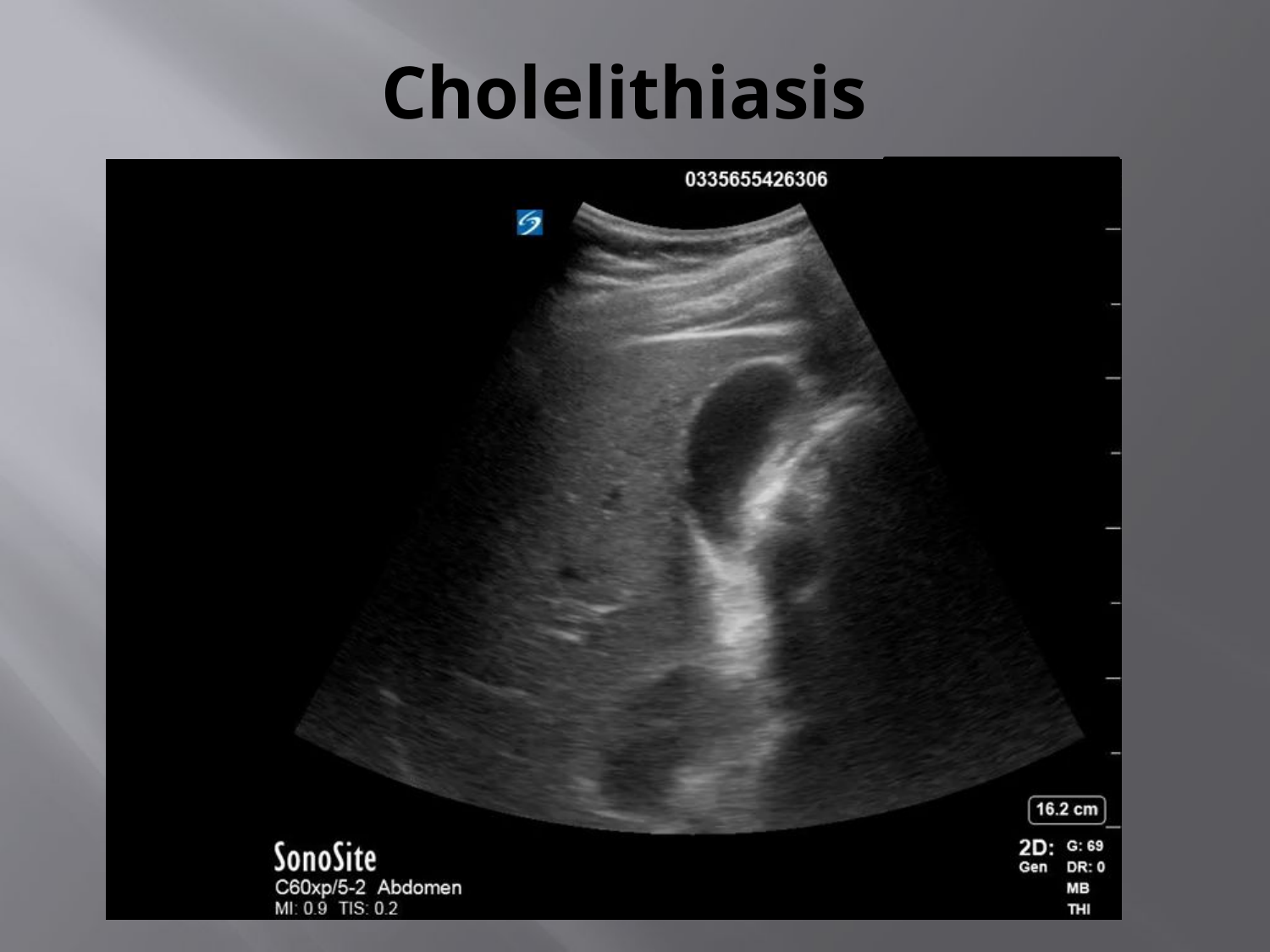

# Cholelithiasis

## Slide 11
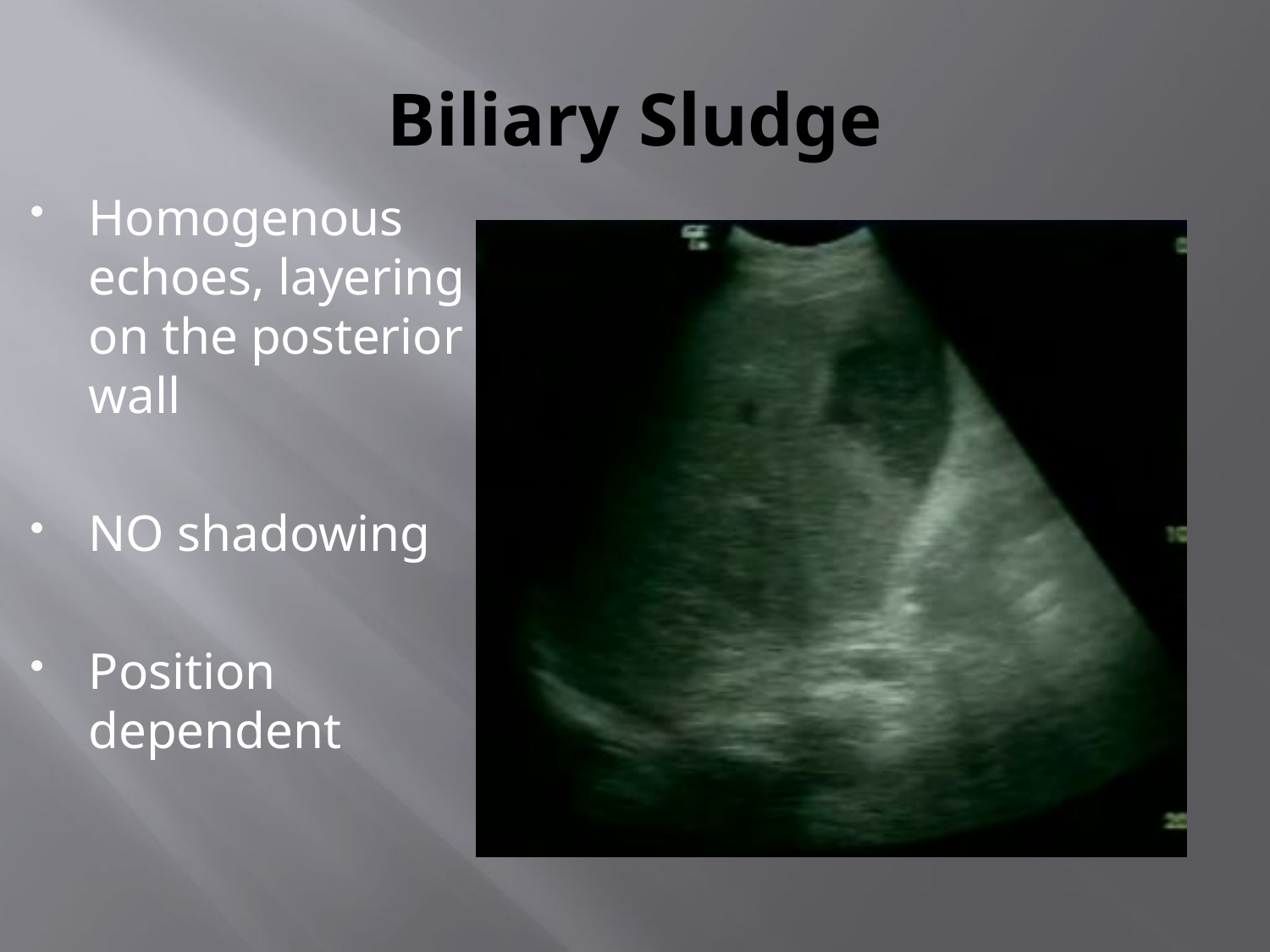

# Biliary Sludge
Homogenous echoes, layering on the posterior wall
NO shadowing
Position dependent

## Slide 12
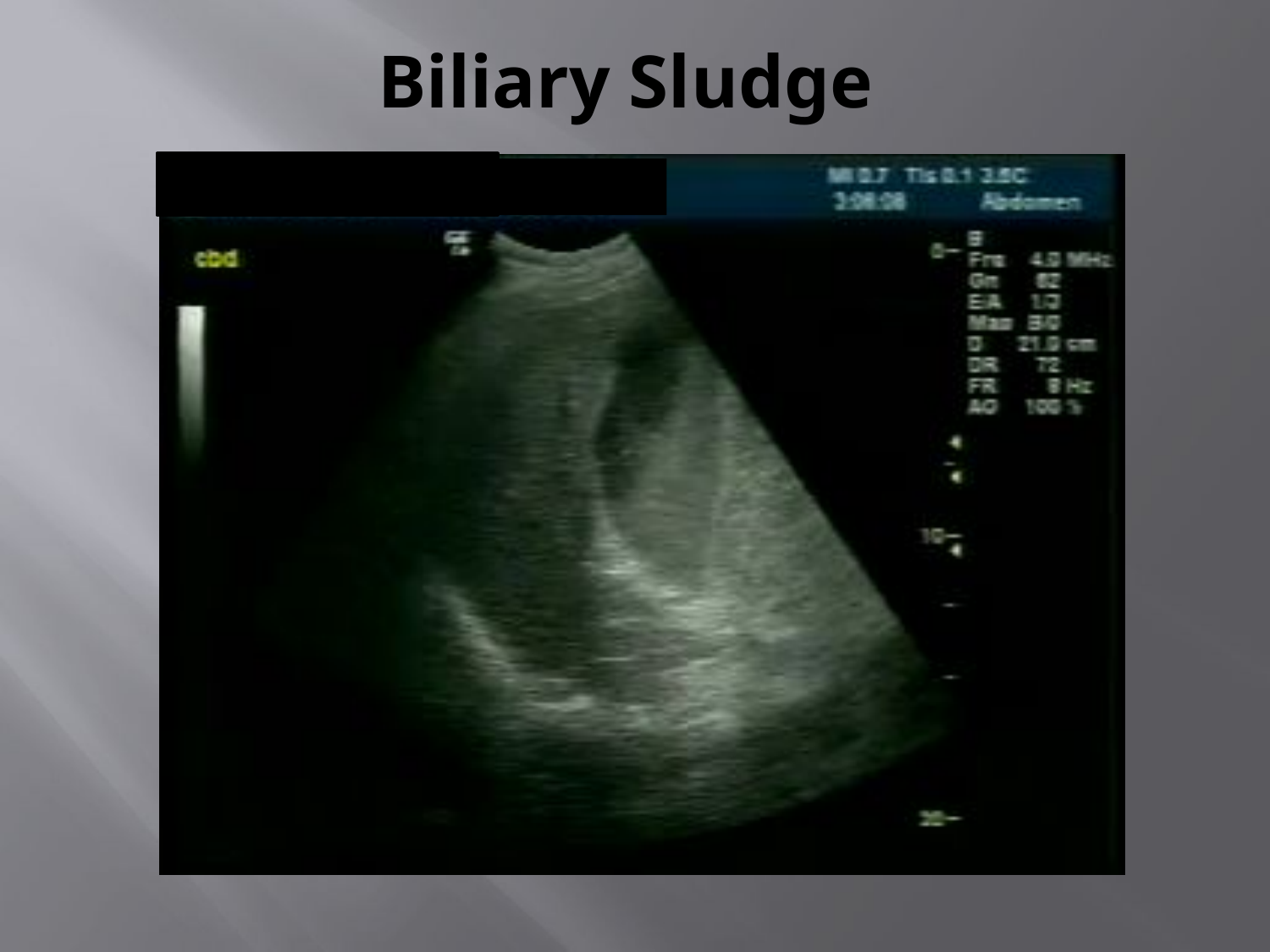

# Biliary Sludge

## Slide 13
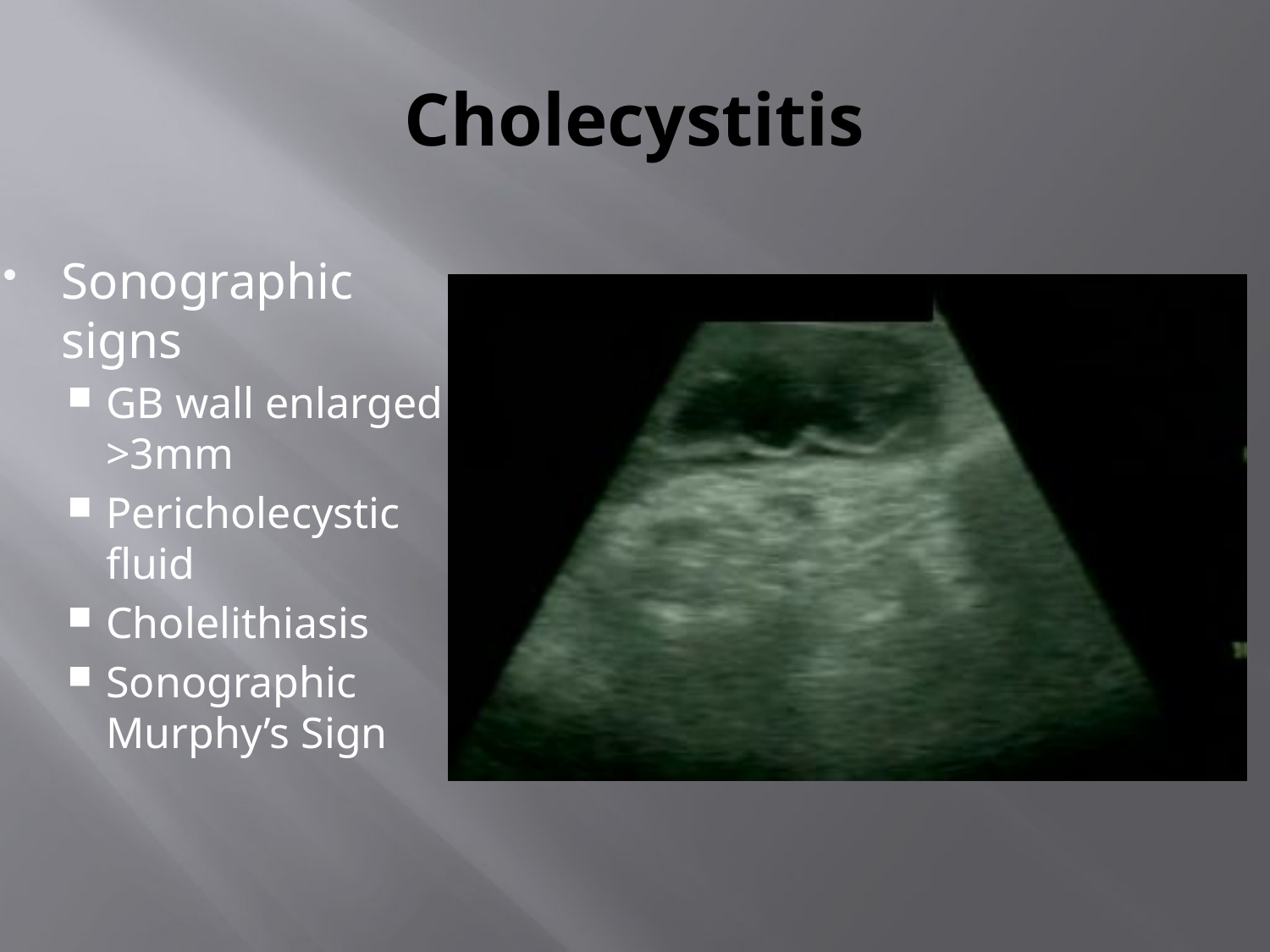

# Cholecystitis
Sonographic signs
GB wall enlarged >3mm
Pericholecystic fluid
Cholelithiasis
Sonographic Murphy’s Sign

## Slide 14
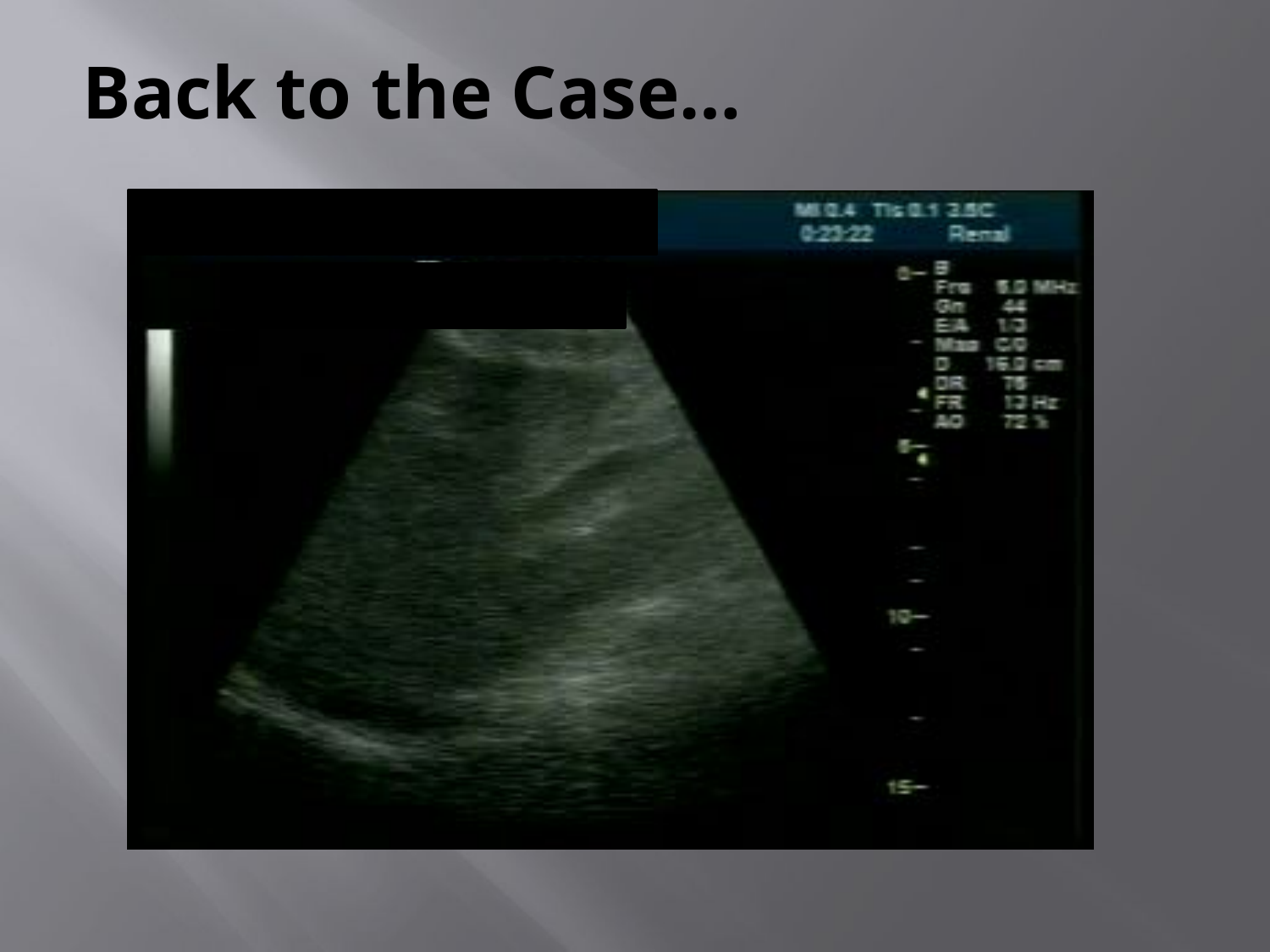

# Back to the Case…

## Slide 15
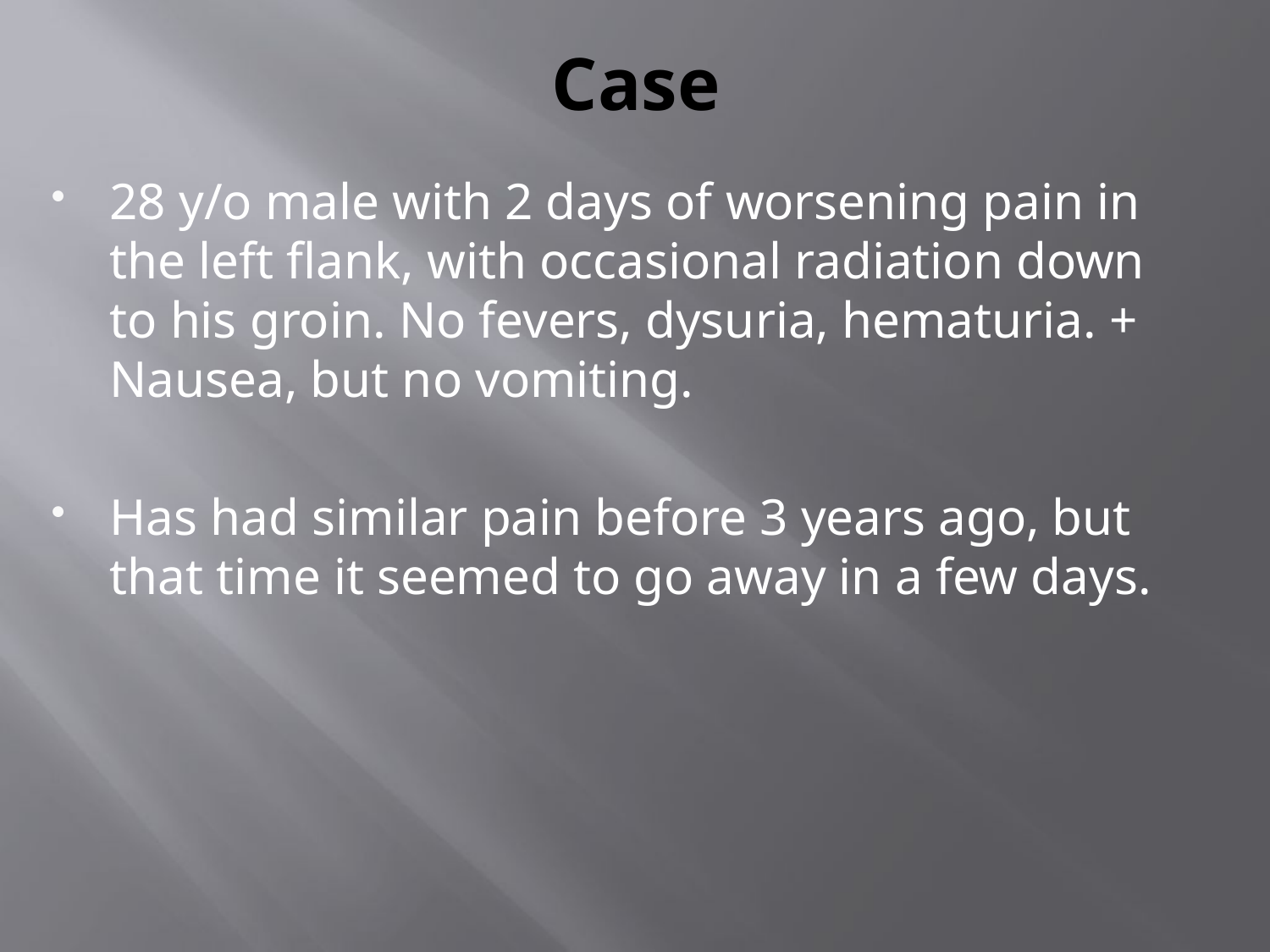

# Case
28 y/o male with 2 days of worsening pain in the left flank, with occasional radiation down to his groin. No fevers, dysuria, hematuria. + Nausea, but no vomiting.
Has had similar pain before 3 years ago, but that time it seemed to go away in a few days.

## Slide 16
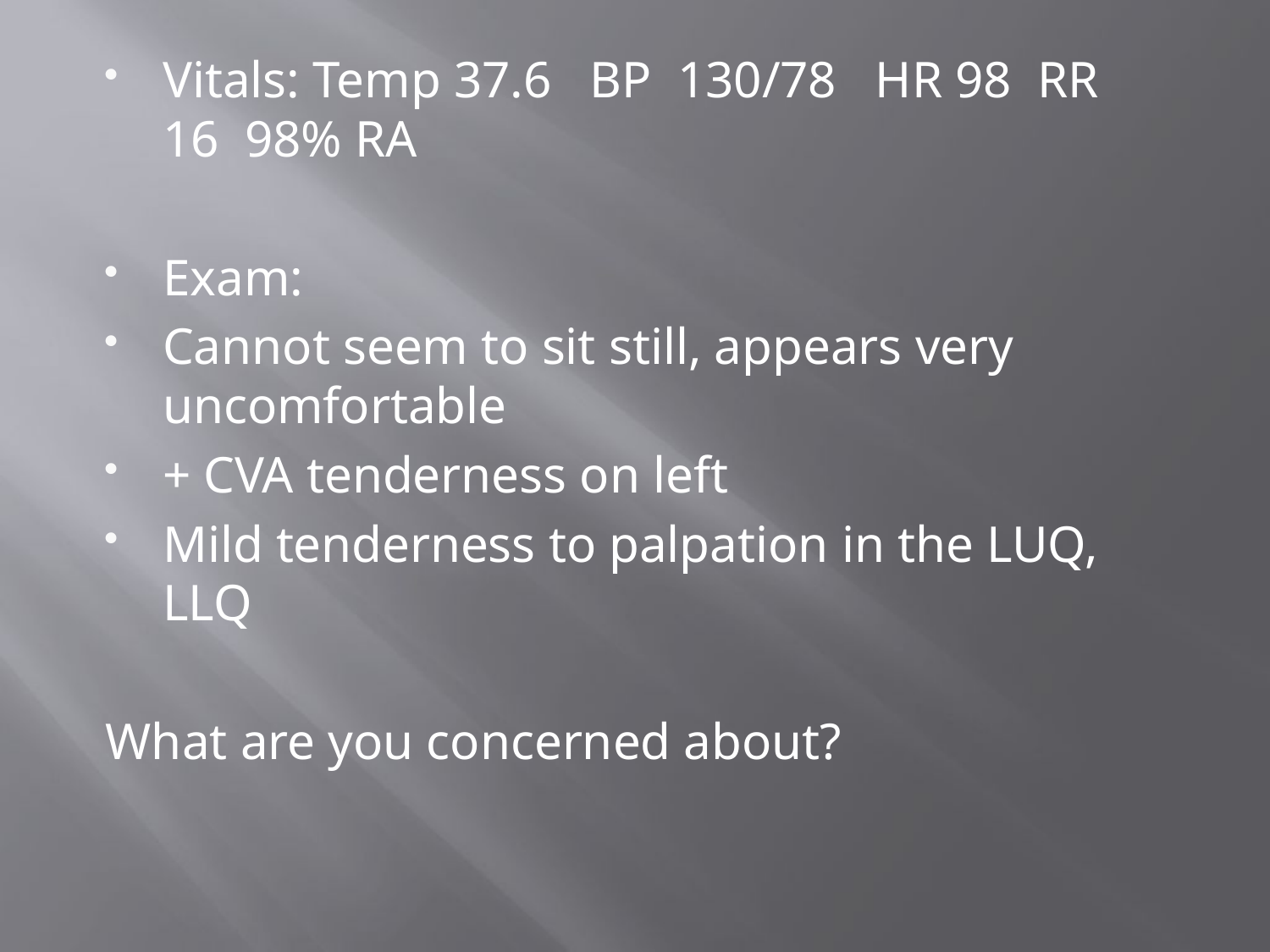

Vitals: Temp 37.6 BP 130/78 HR 98 RR 16 98% RA
Exam:
Cannot seem to sit still, appears very uncomfortable
+ CVA tenderness on left
Mild tenderness to palpation in the LUQ, LLQ
What are you concerned about?

## Slide 17
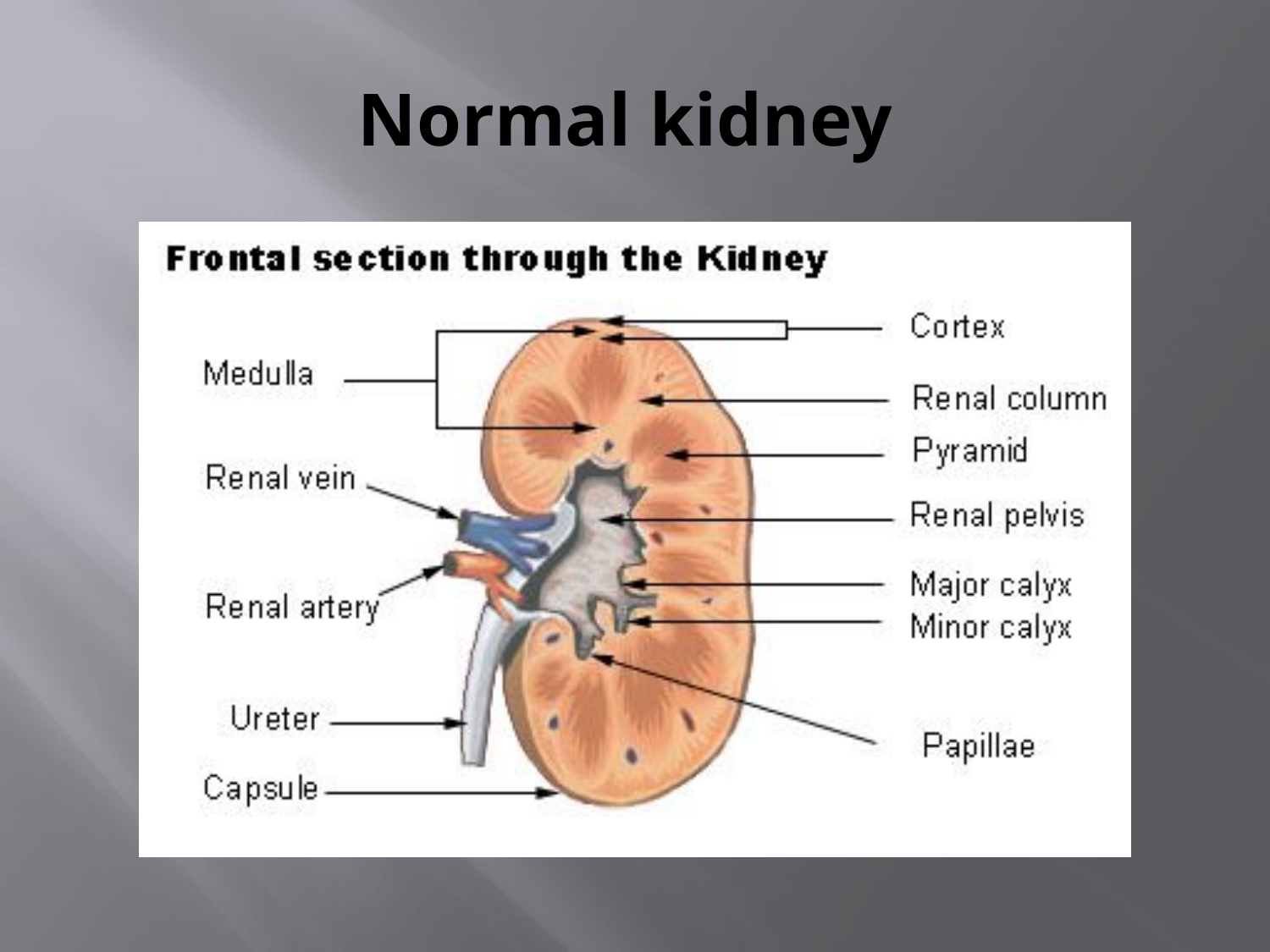

# Normal kidney

## Slide 18
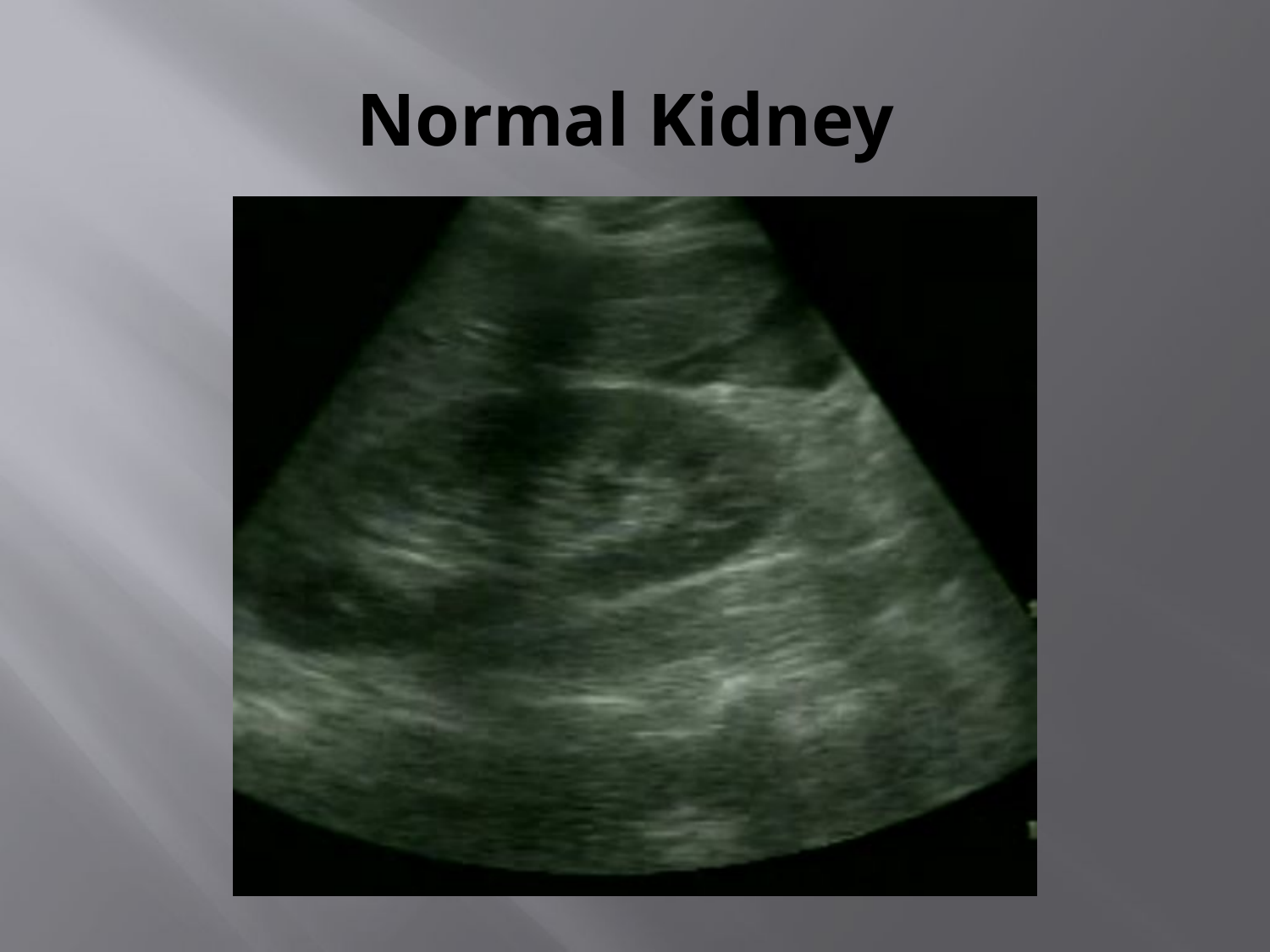

# Normal Kidney

## Slide 19
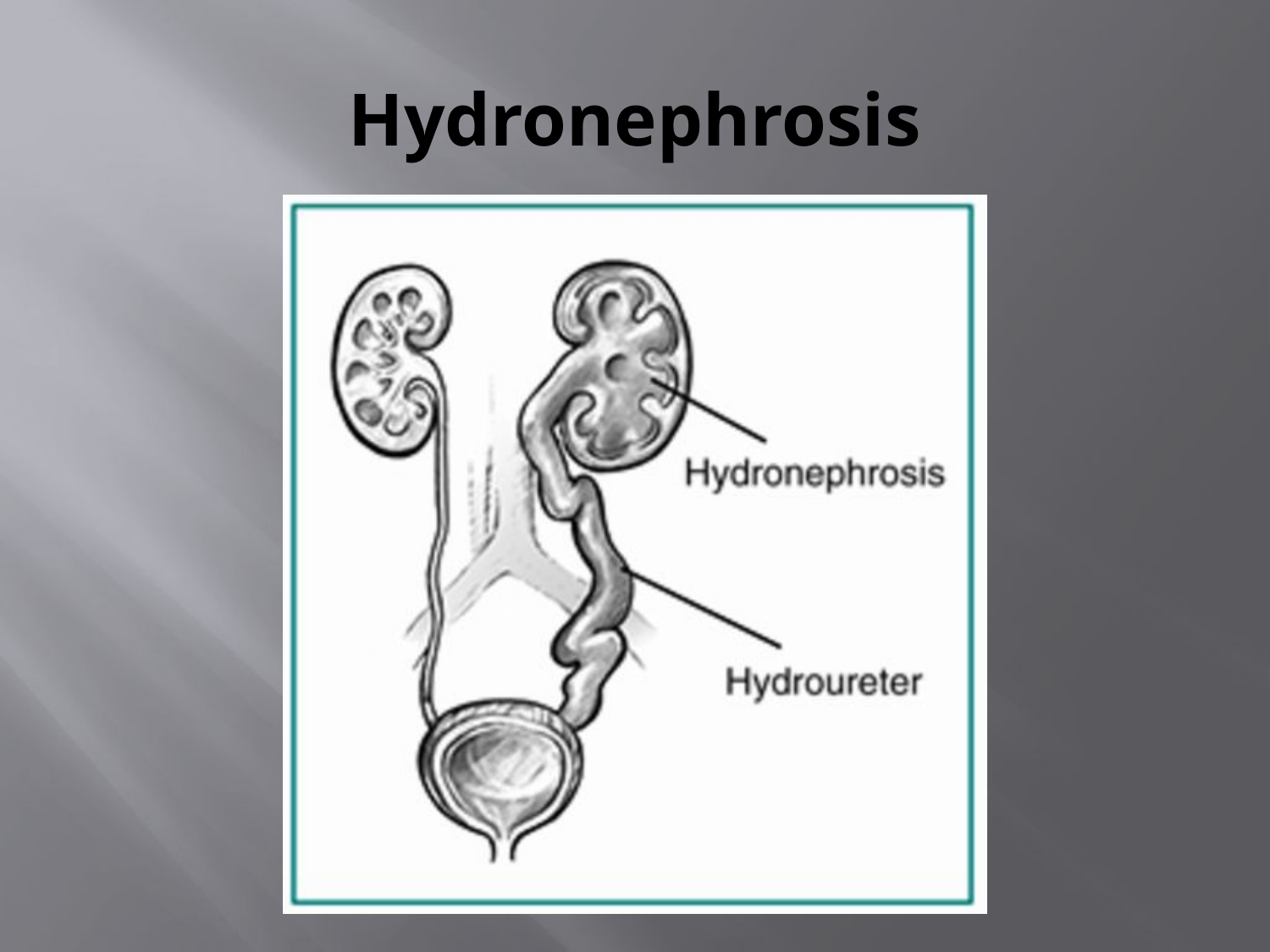

# Hydronephrosis

## Slide 20
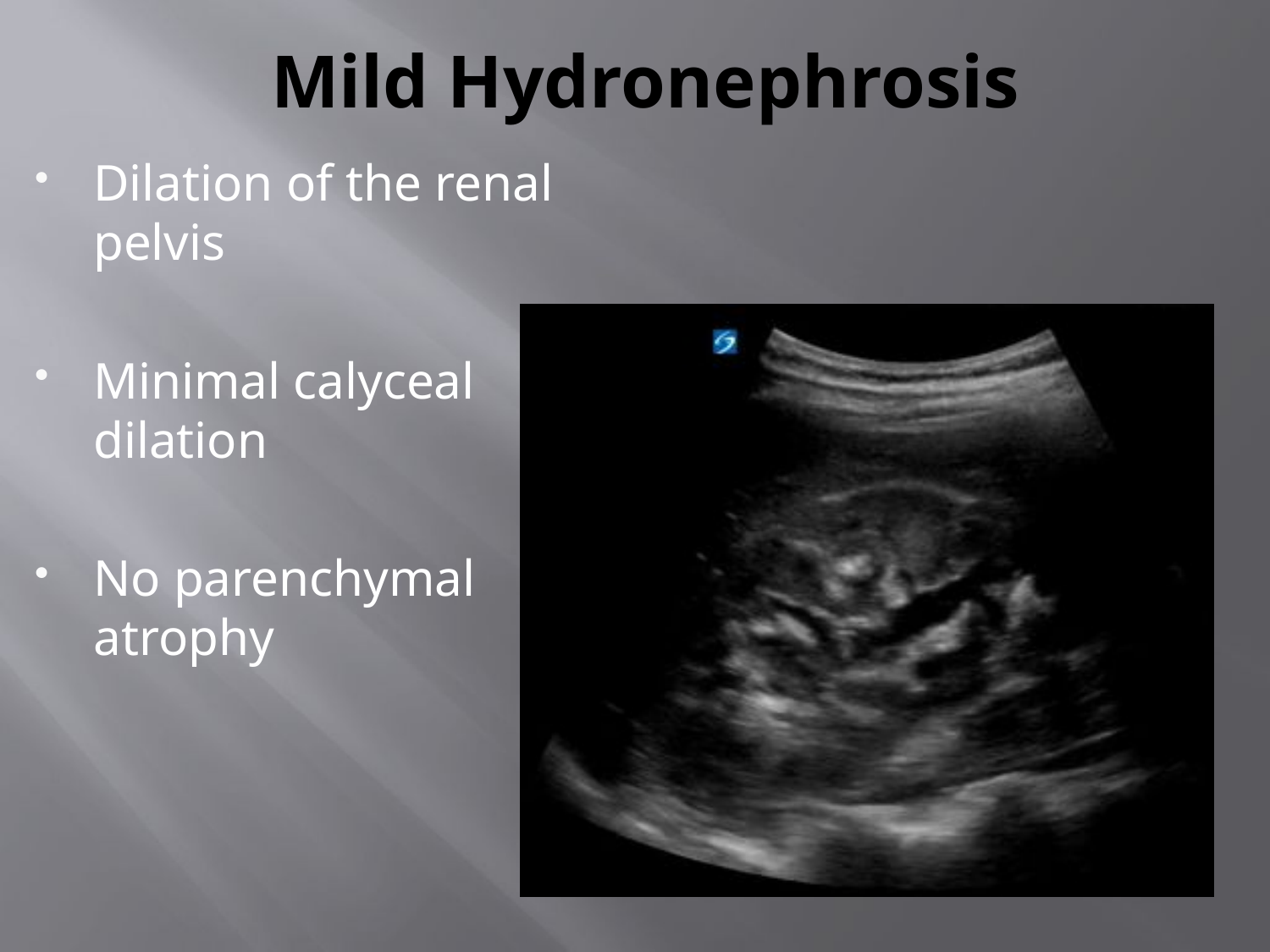

# Mild Hydronephrosis
Dilation of the renal pelvis
Minimal calyceal dilation
No parenchymal atrophy

## Slide 21
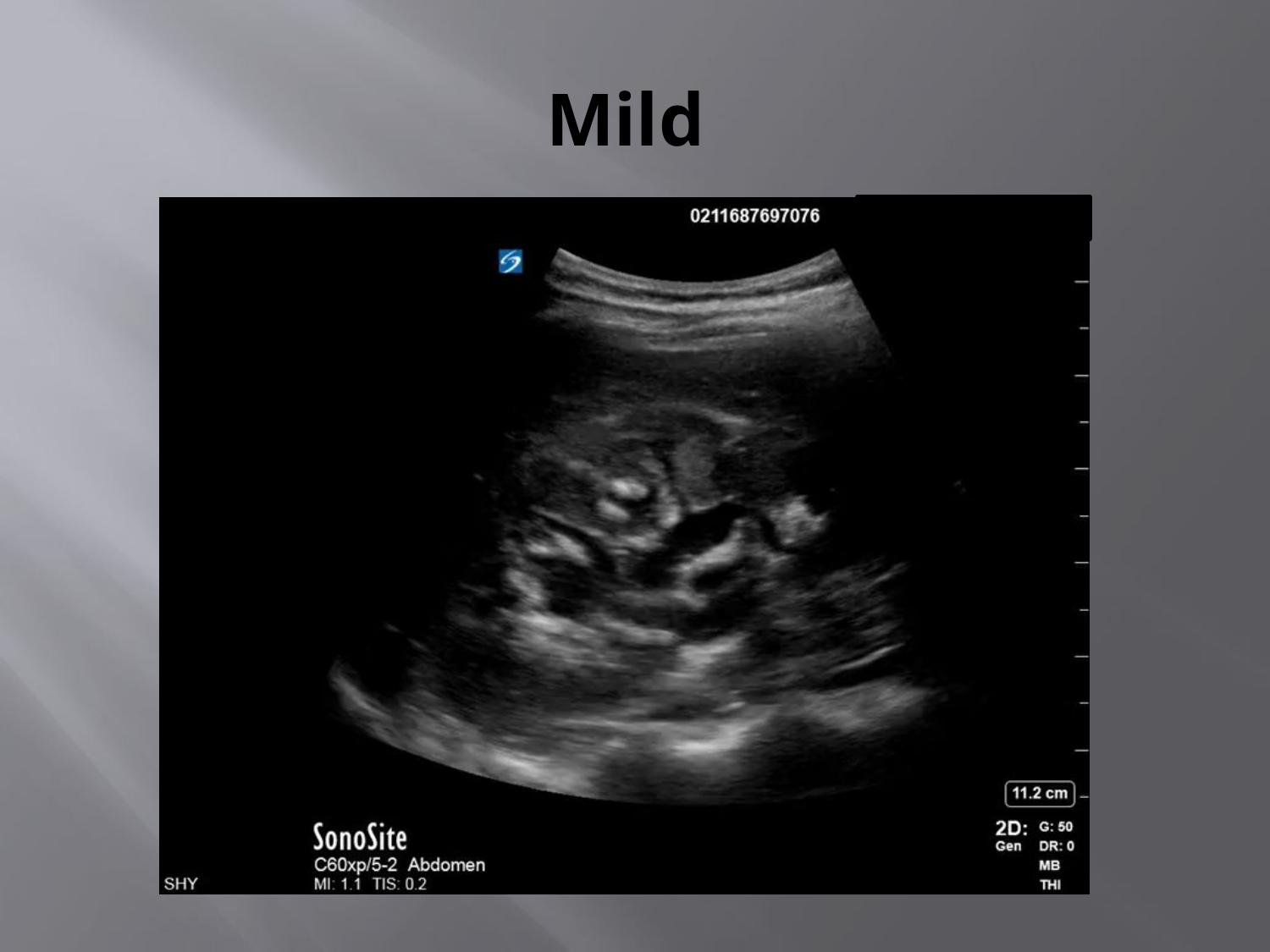

# Mild

## Slide 22
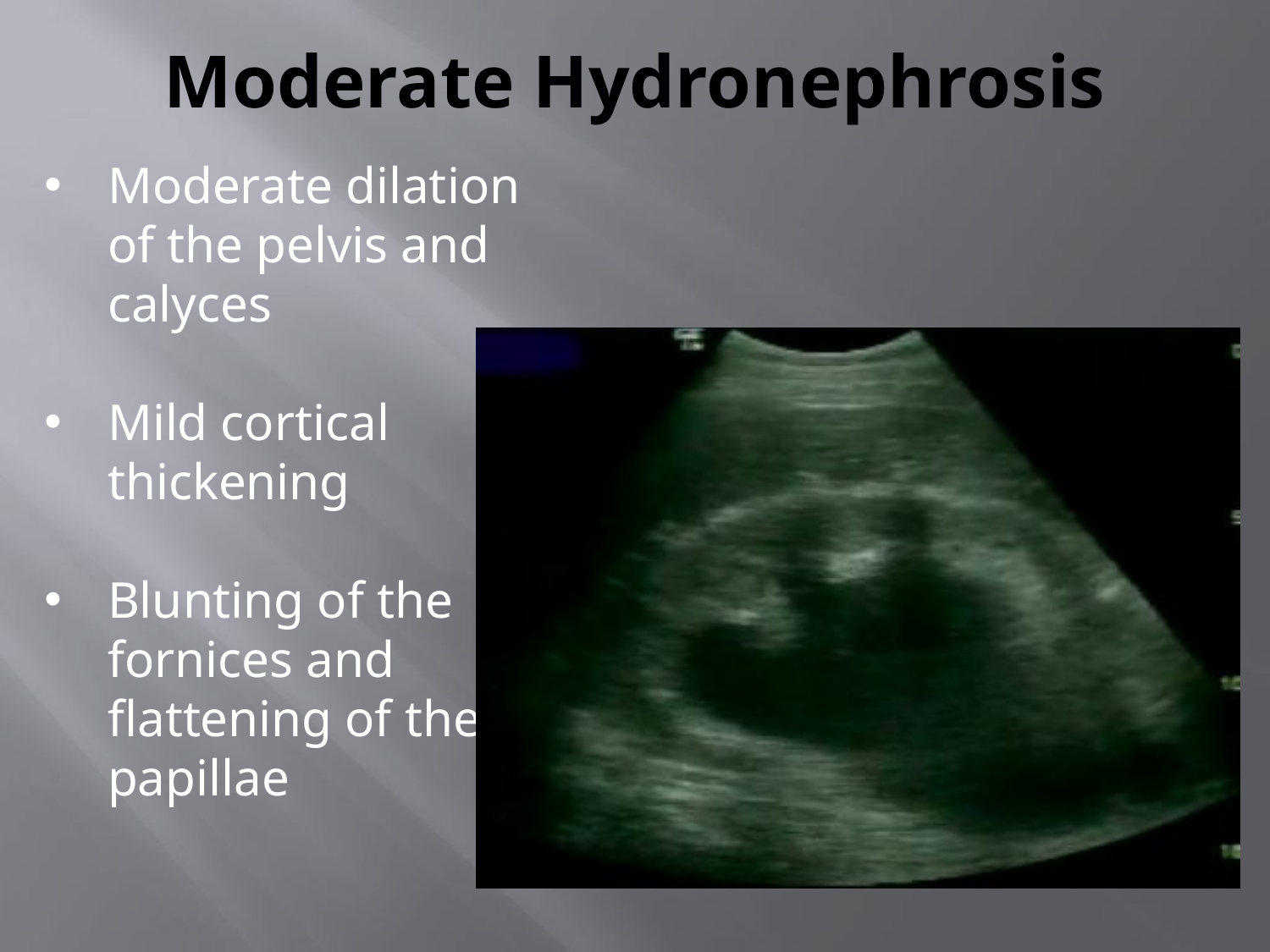

# Moderate Hydronephrosis
Moderate dilation of the pelvis and calyces
Mild cortical thickening
Blunting of the fornices and flattening of the papillae

## Slide 23
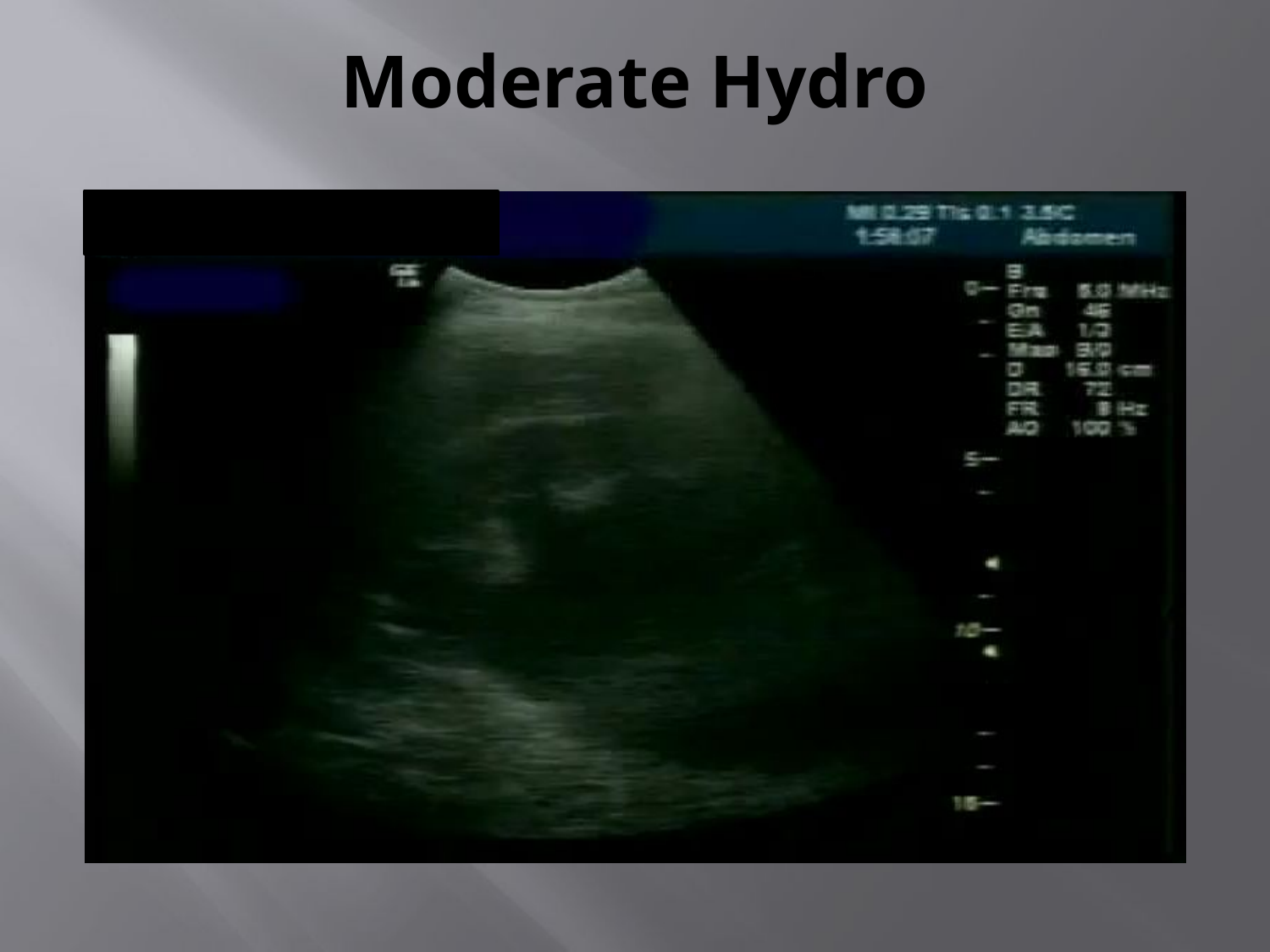

# Moderate Hydro

## Slide 24
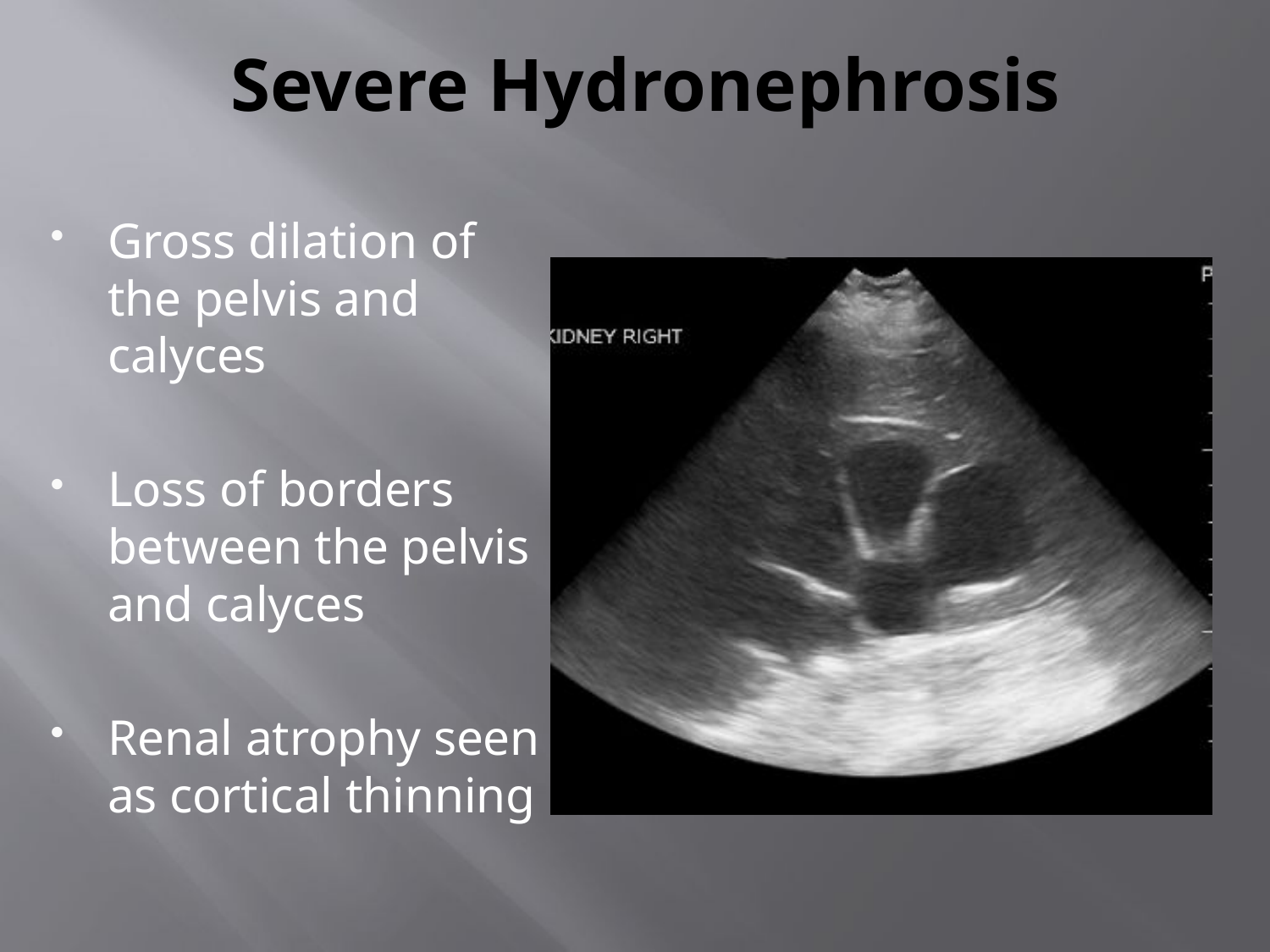

# Severe Hydronephrosis
Gross dilation of the pelvis and calyces
Loss of borders between the pelvis and calyces
Renal atrophy seen as cortical thinning

## Slide 25
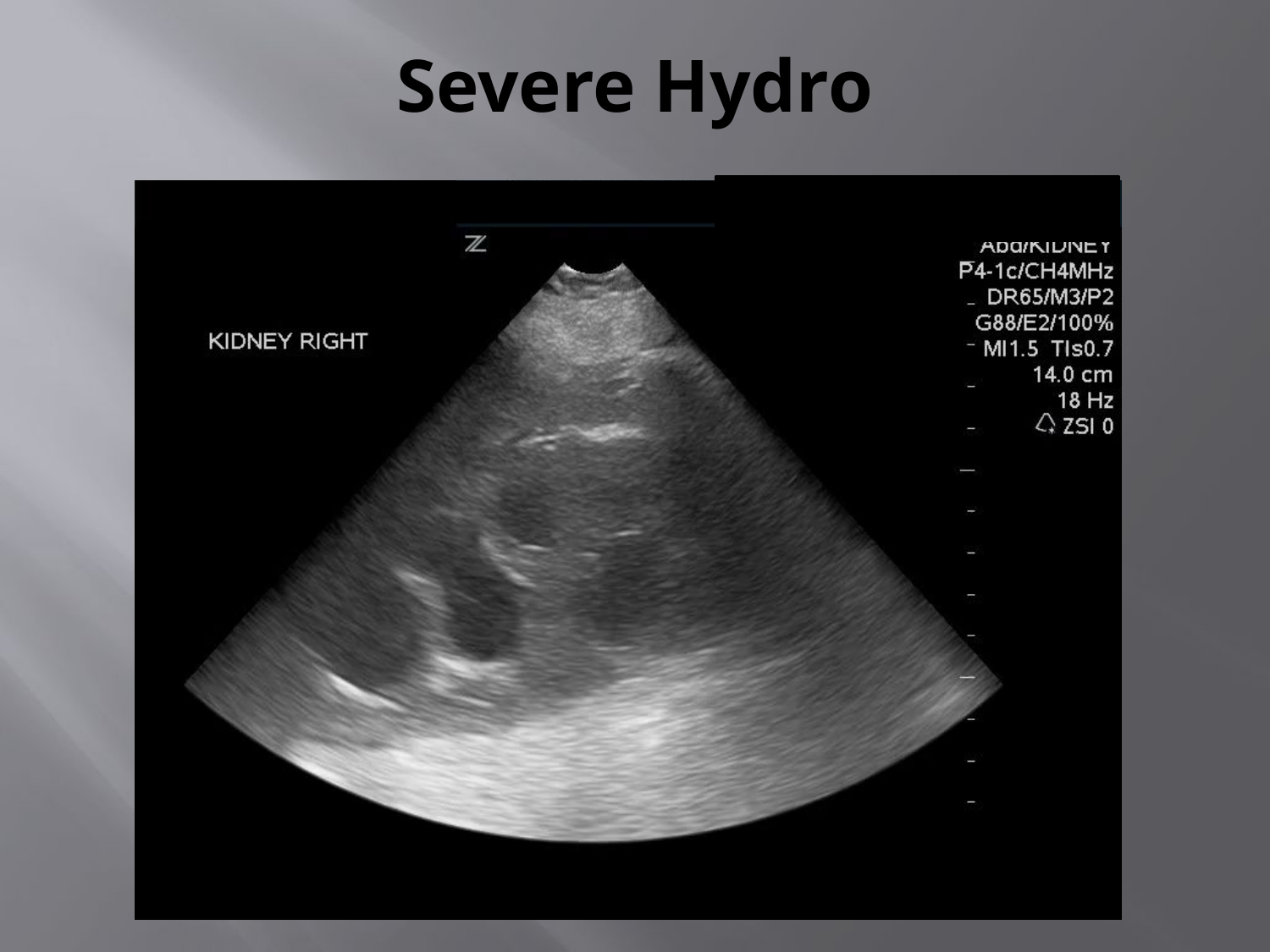

# Severe Hydro

## Slide 26
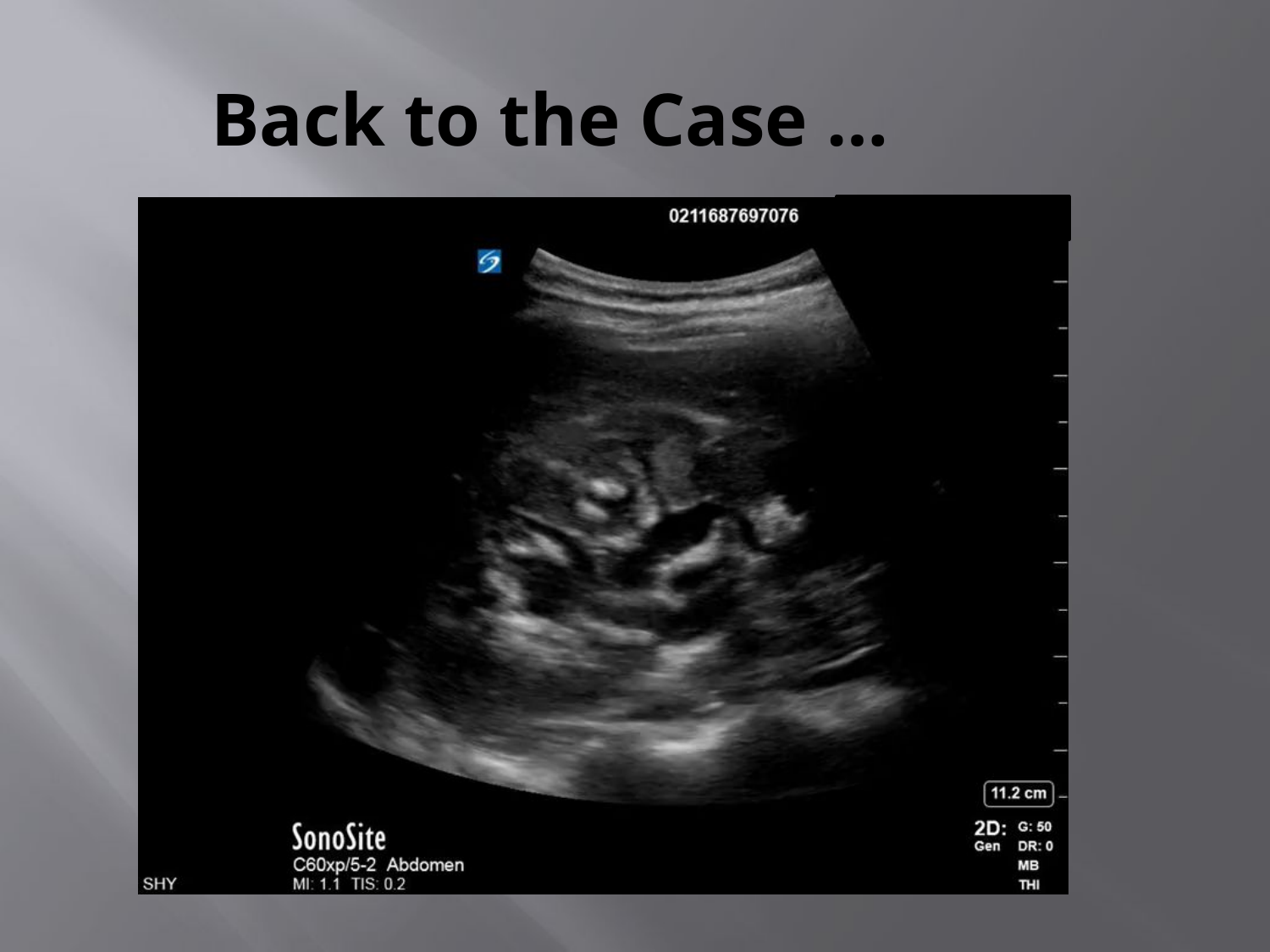

# Back to the Case …

## Slide 27
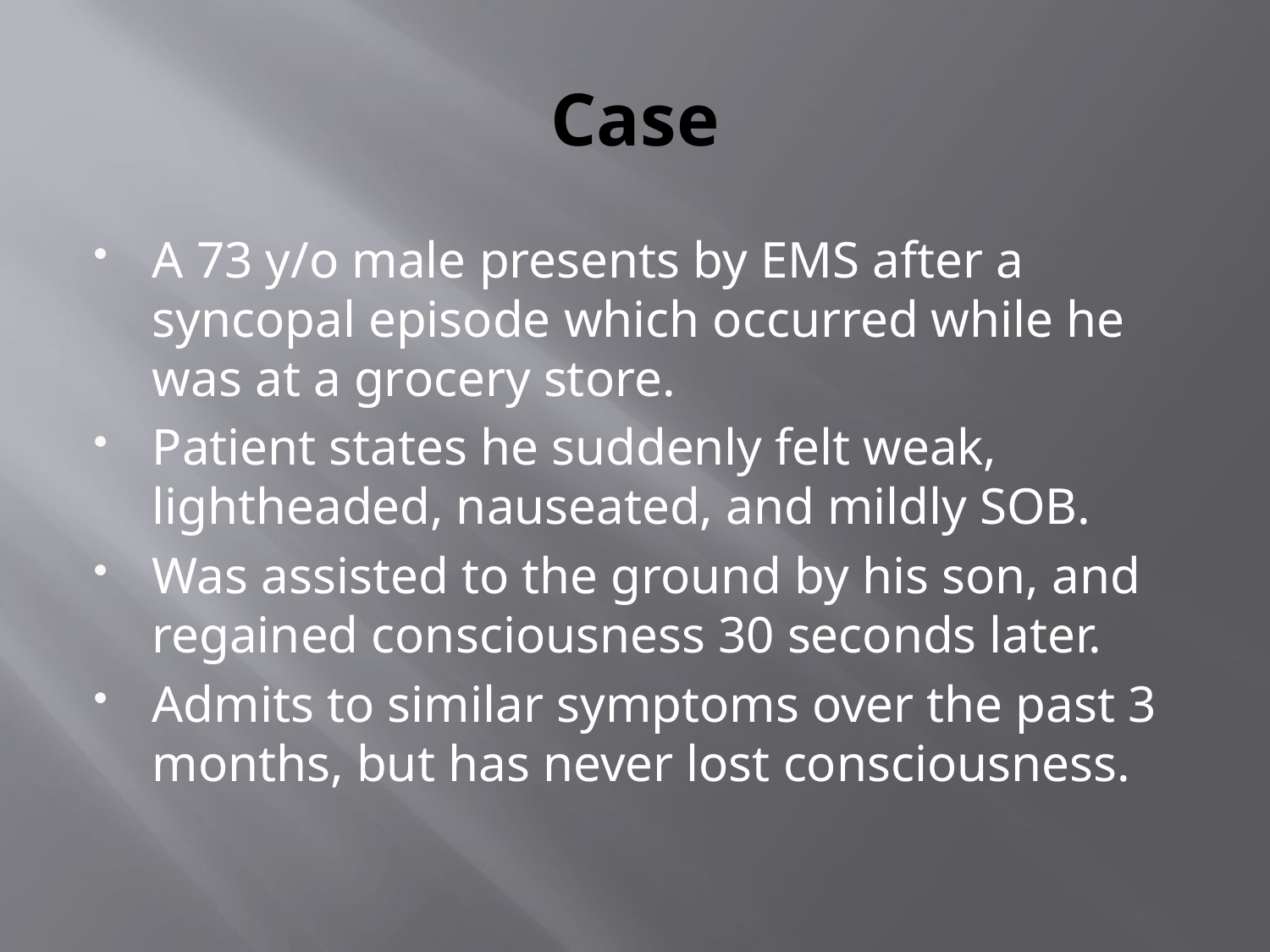

# Case
A 73 y/o male presents by EMS after a syncopal episode which occurred while he was at a grocery store.
Patient states he suddenly felt weak, lightheaded, nauseated, and mildly SOB.
Was assisted to the ground by his son, and regained consciousness 30 seconds later.
Admits to similar symptoms over the past 3 months, but has never lost consciousness.

## Slide 28
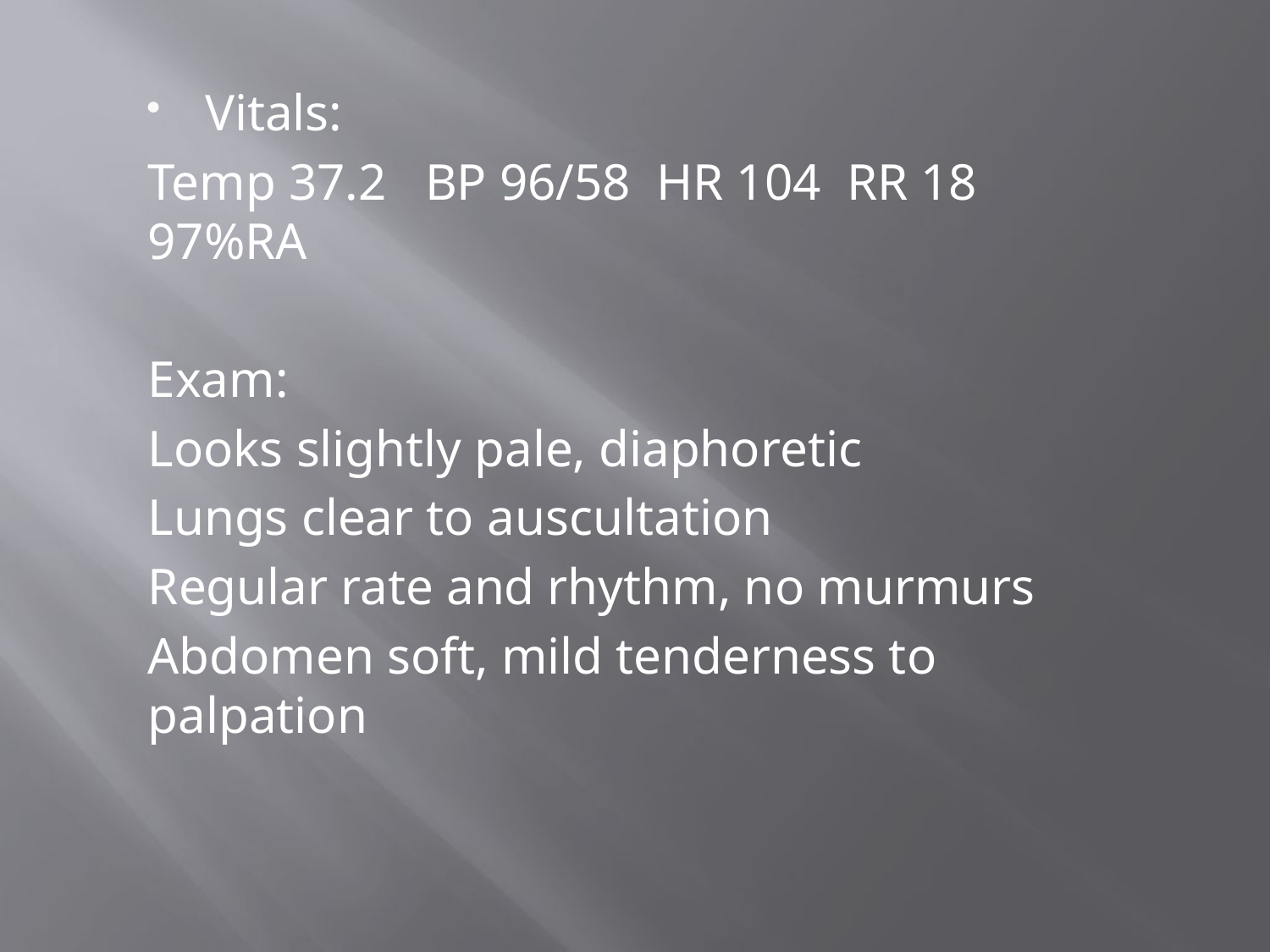

Vitals:
Temp 37.2 BP 96/58 HR 104 RR 18 97%RA
Exam:
Looks slightly pale, diaphoretic
Lungs clear to auscultation
Regular rate and rhythm, no murmurs
Abdomen soft, mild tenderness to palpation

## Slide 29
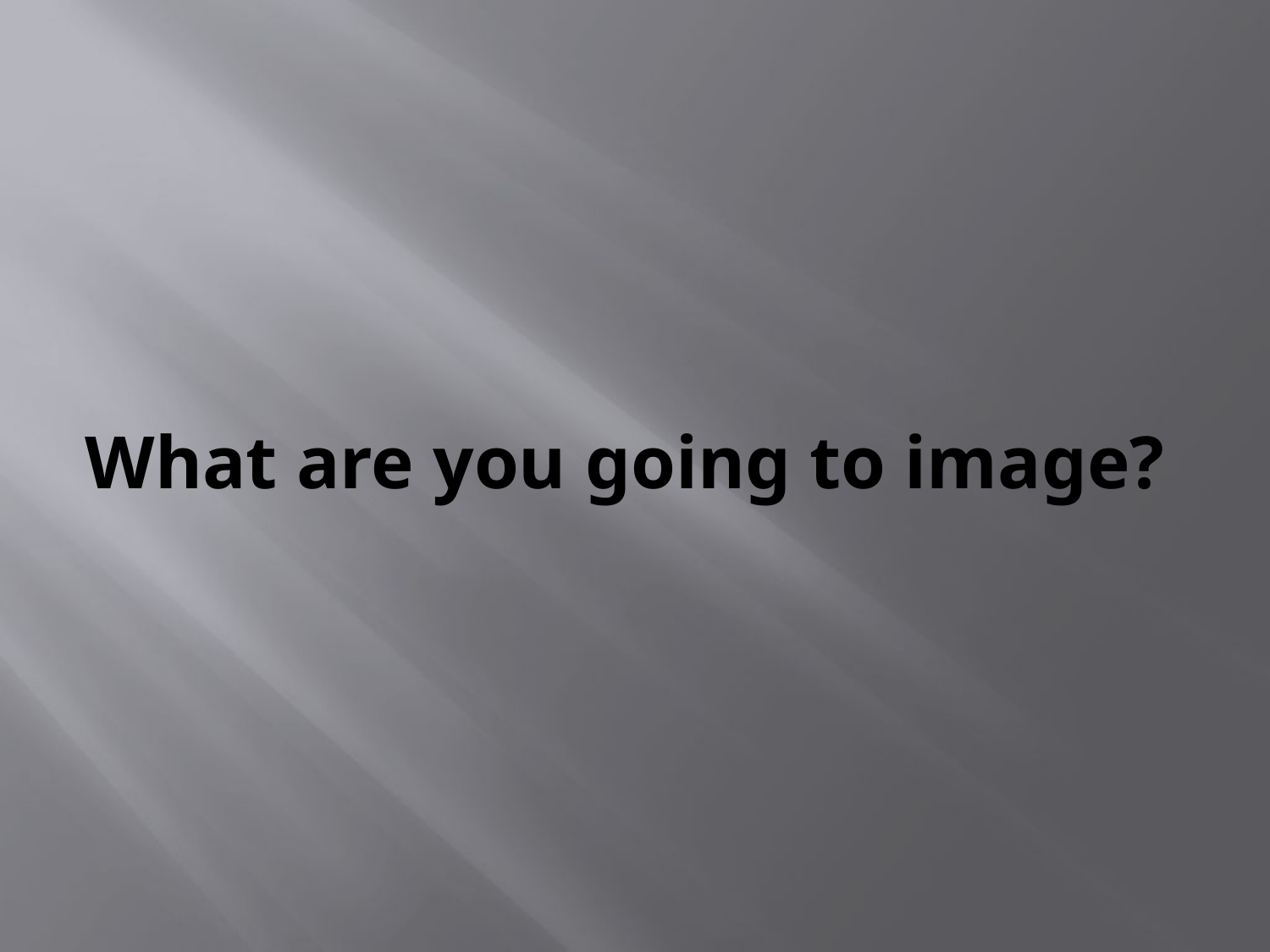

# What are you going to image?

## Slide 30
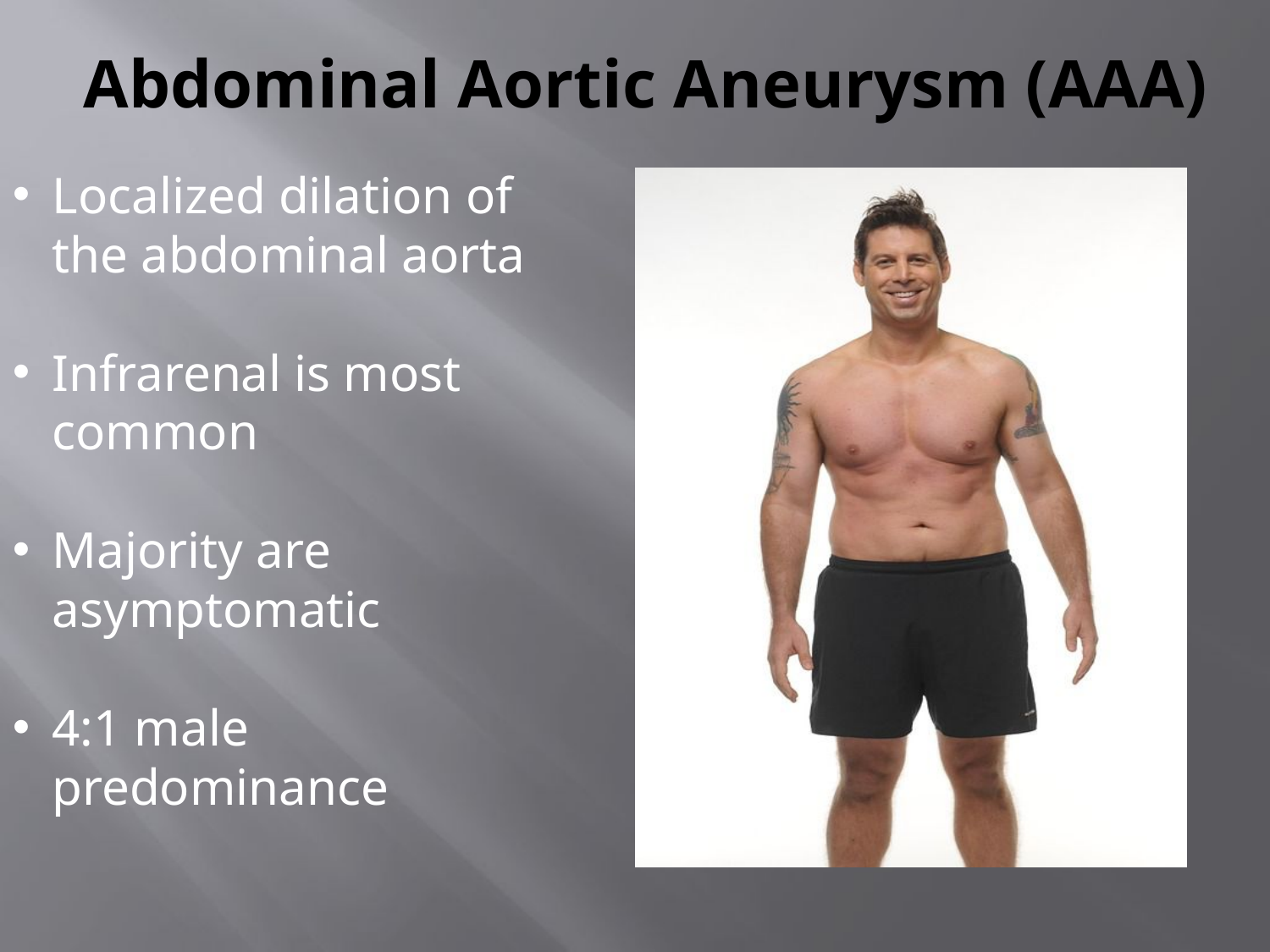

# Abdominal Aortic Aneurysm (AAA)
Localized dilation of the abdominal aorta
Infrarenal is most common
Majority are asymptomatic
4:1 male predominance

## Slide 31
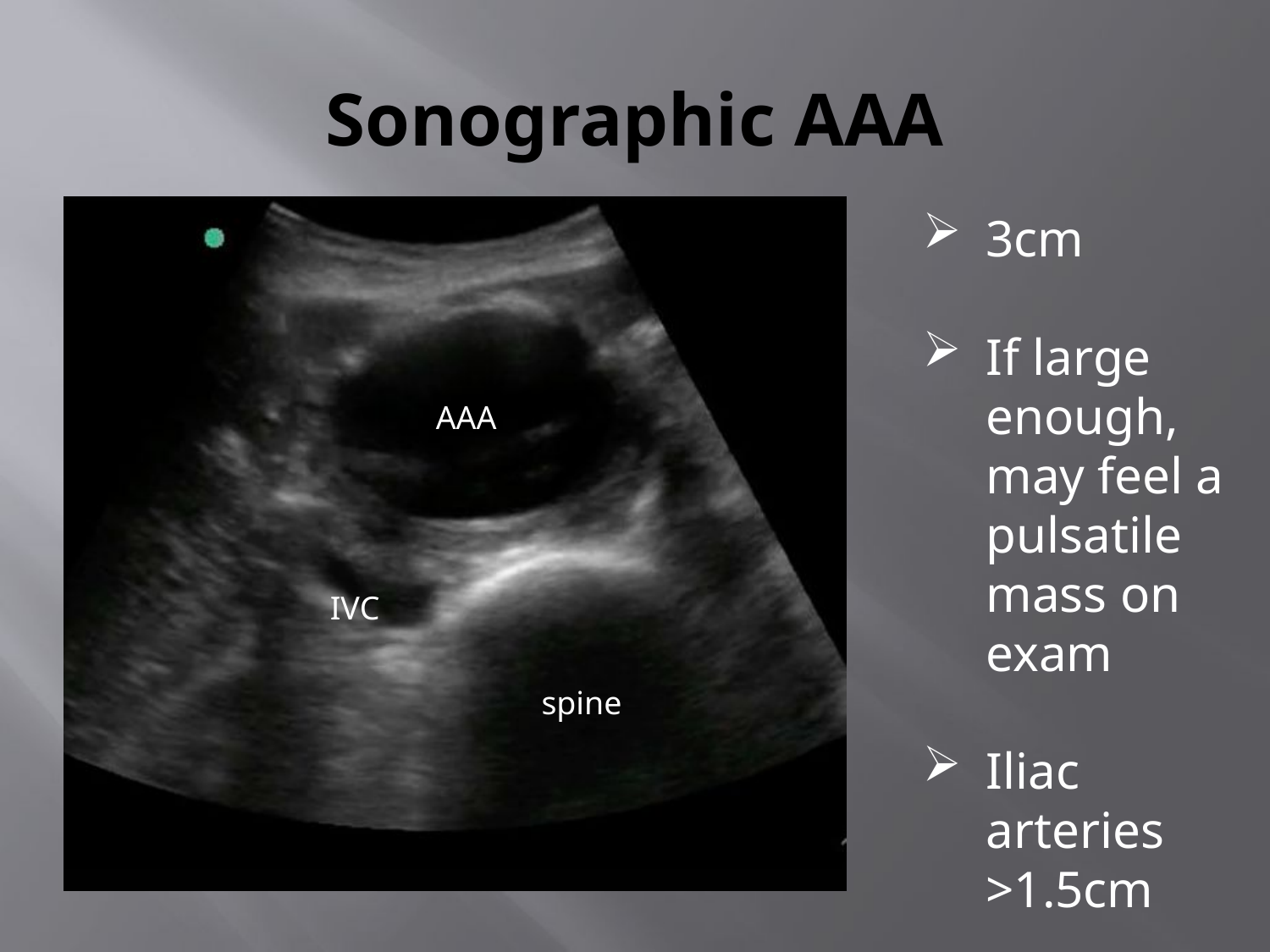

# Sonographic AAA
3cm
If large enough, may feel a pulsatile mass on exam
Iliac arteries >1.5cm
AAA
IVC
spine

## Slide 32
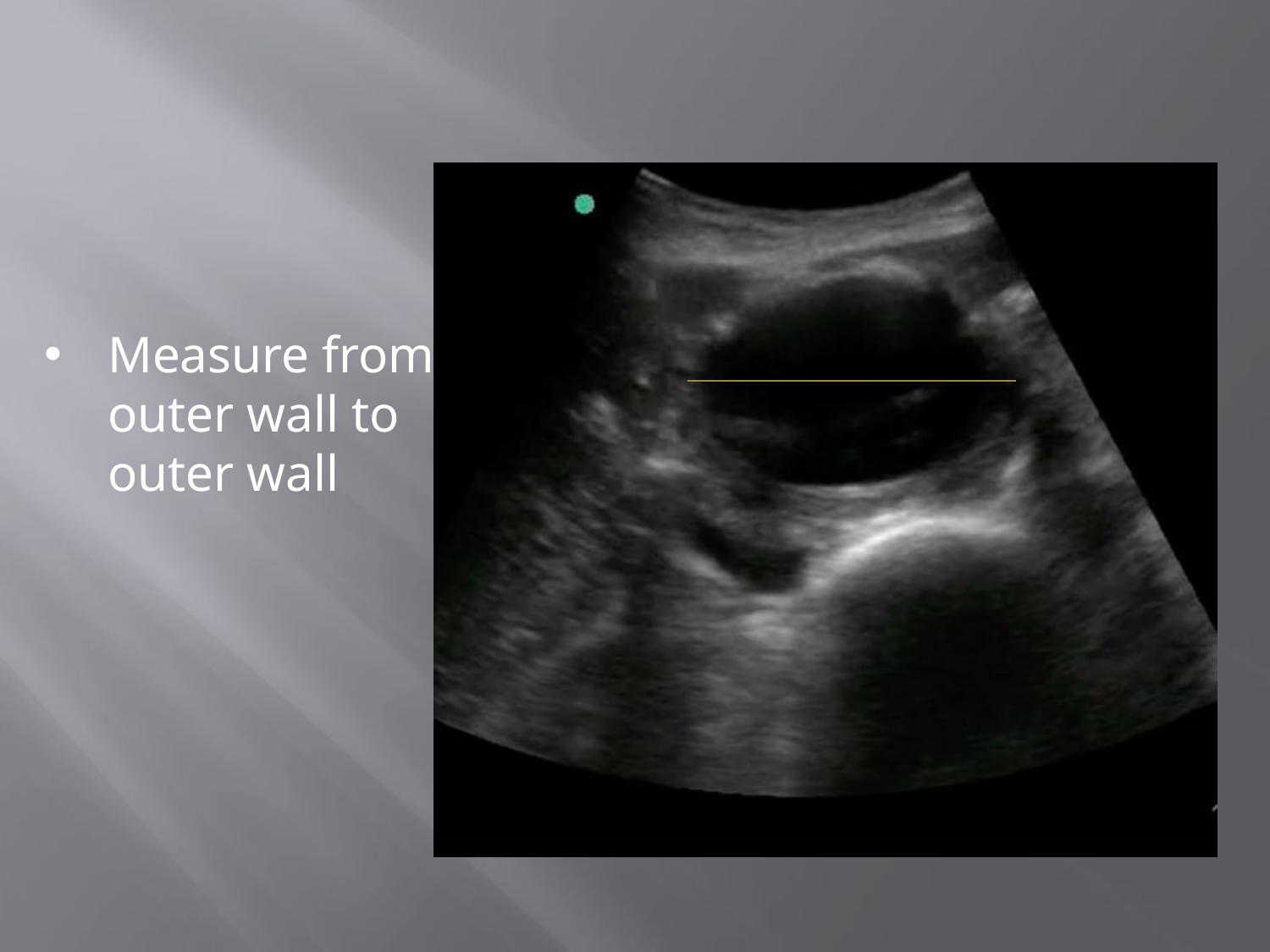

Measure from outer wall to outer wall

## Slide 33
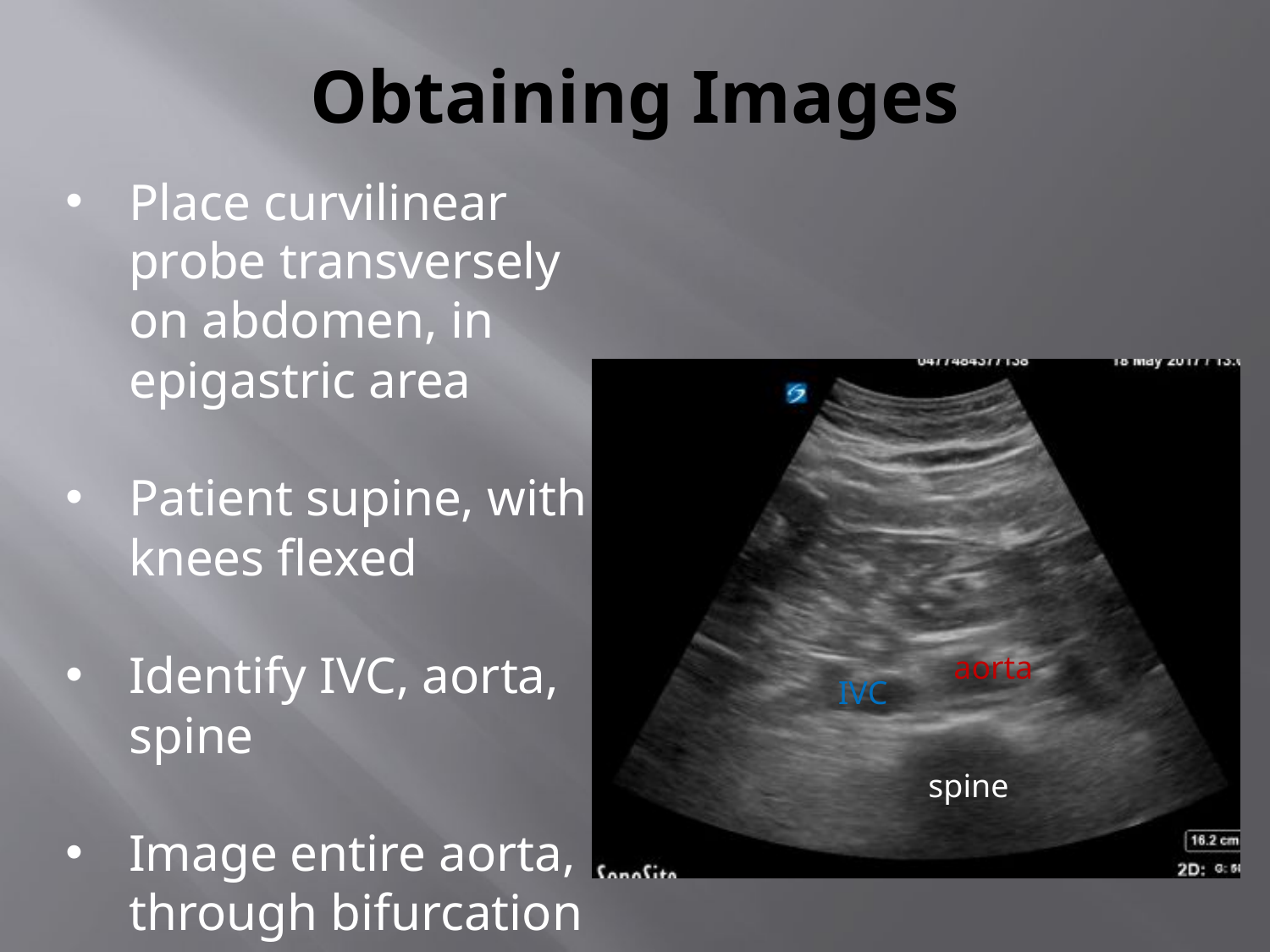

# Obtaining Images
Place curvilinear probe transversely on abdomen, in epigastric area
Patient supine, with knees flexed
Identify IVC, aorta, spine
Image entire aorta, through bifurcation
aorta
IVC
spine

## Slide 34
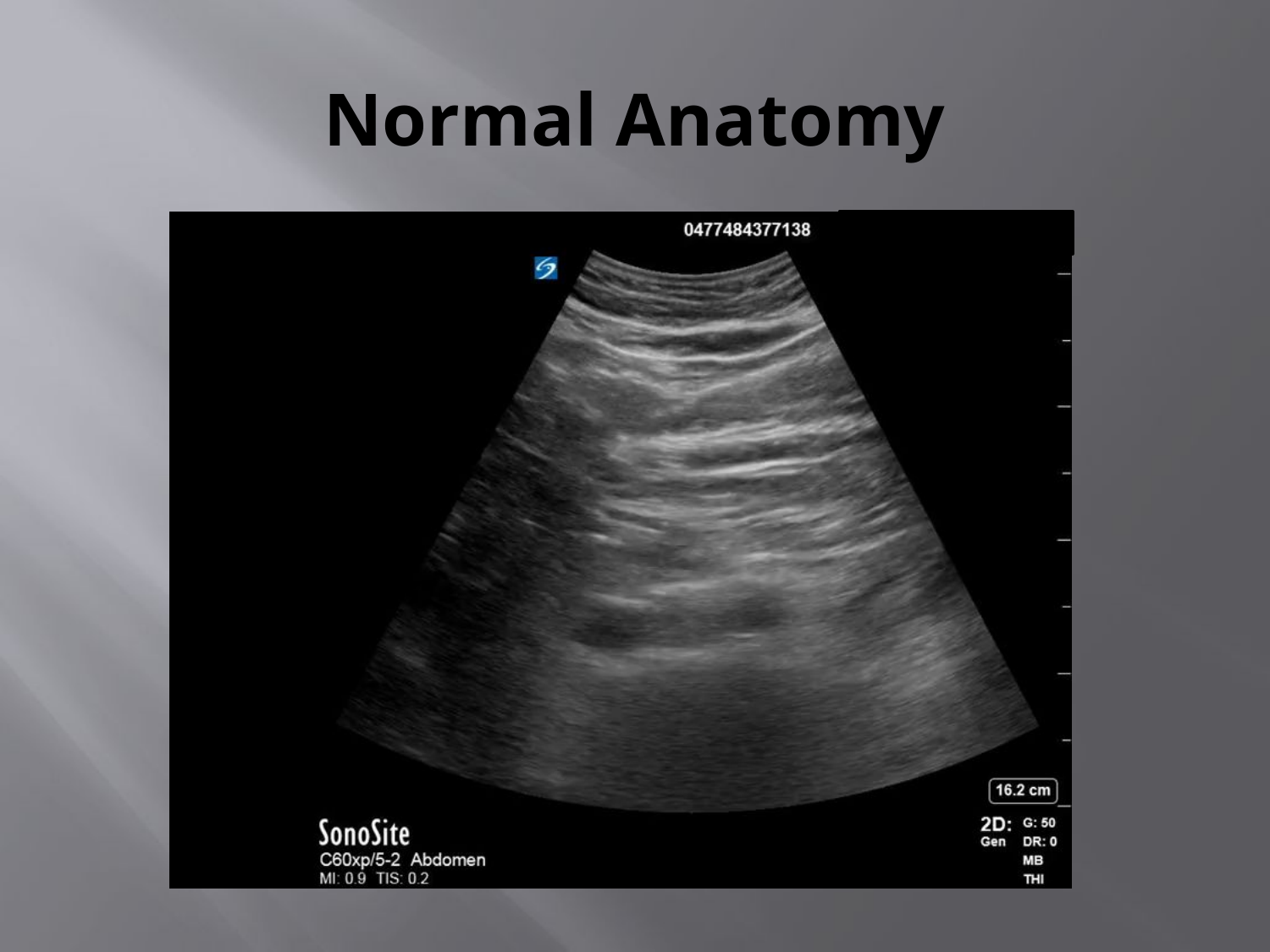

# Normal Anatomy

## Slide 35
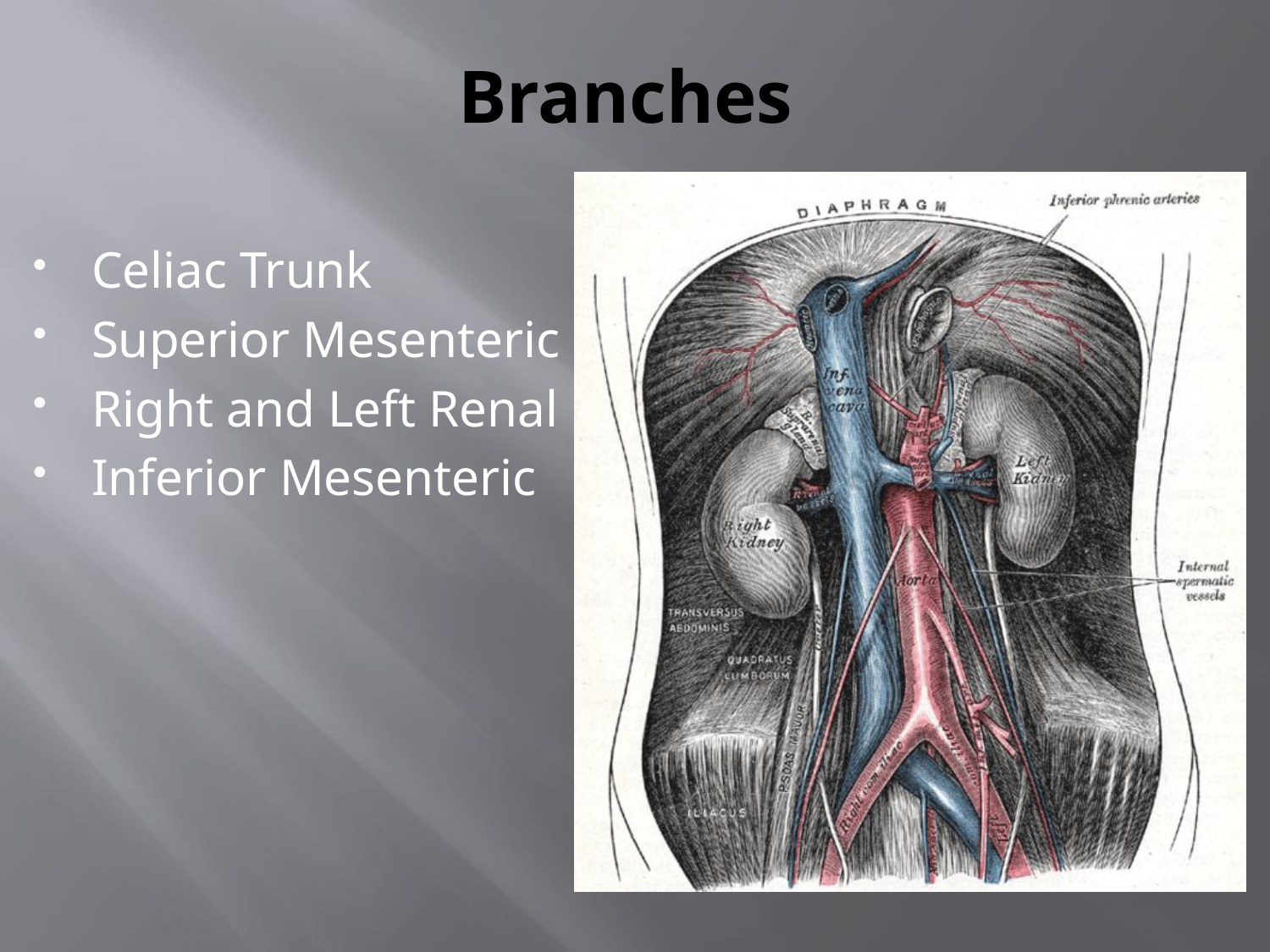

# Branches
Celiac Trunk
Superior Mesenteric
Right and Left Renal
Inferior Mesenteric

## Slide 36
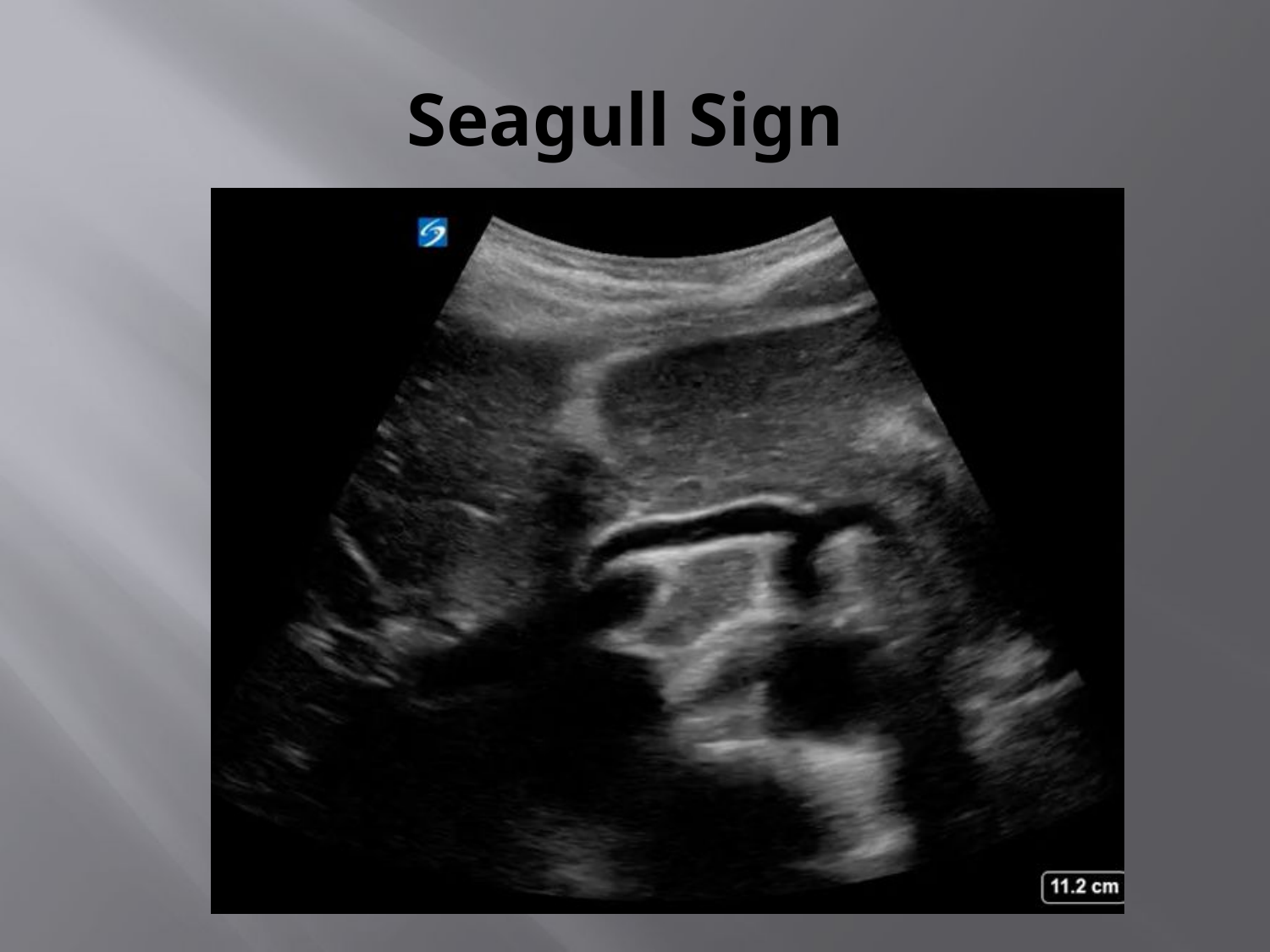

# Seagull Sign

## Slide 37
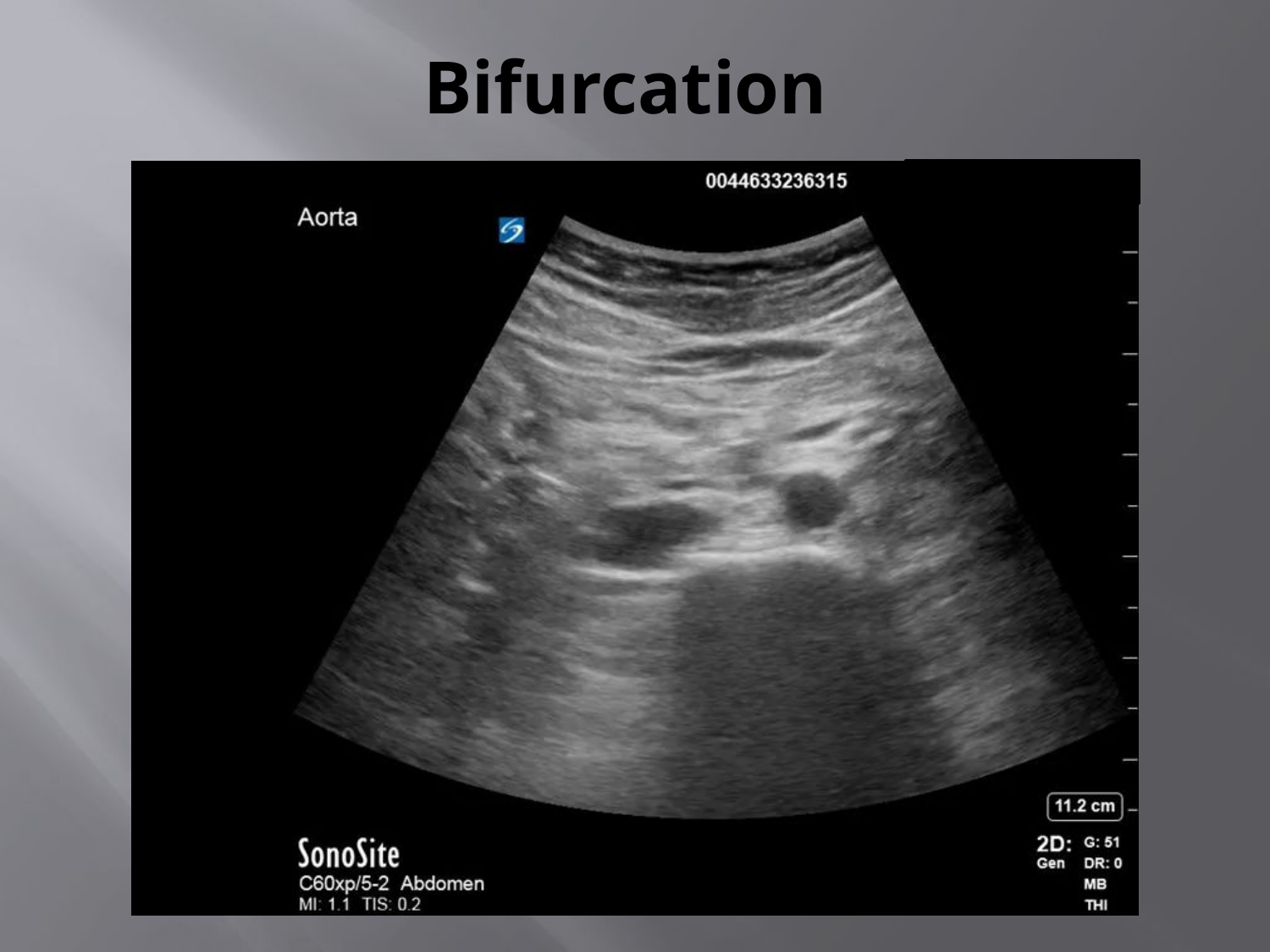

# Bifurcation

## Slide 38
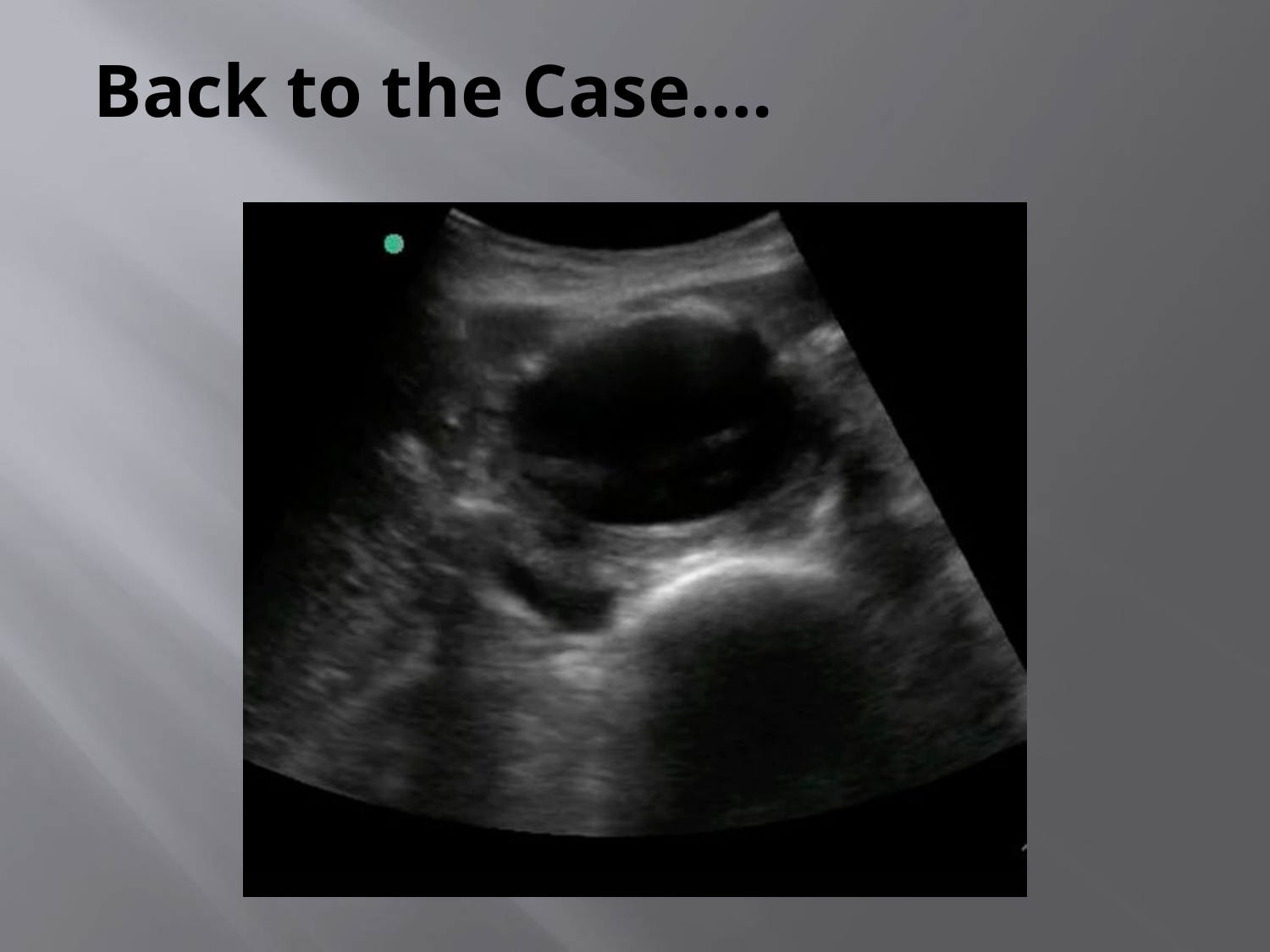

# Back to the Case….

## Slide 39
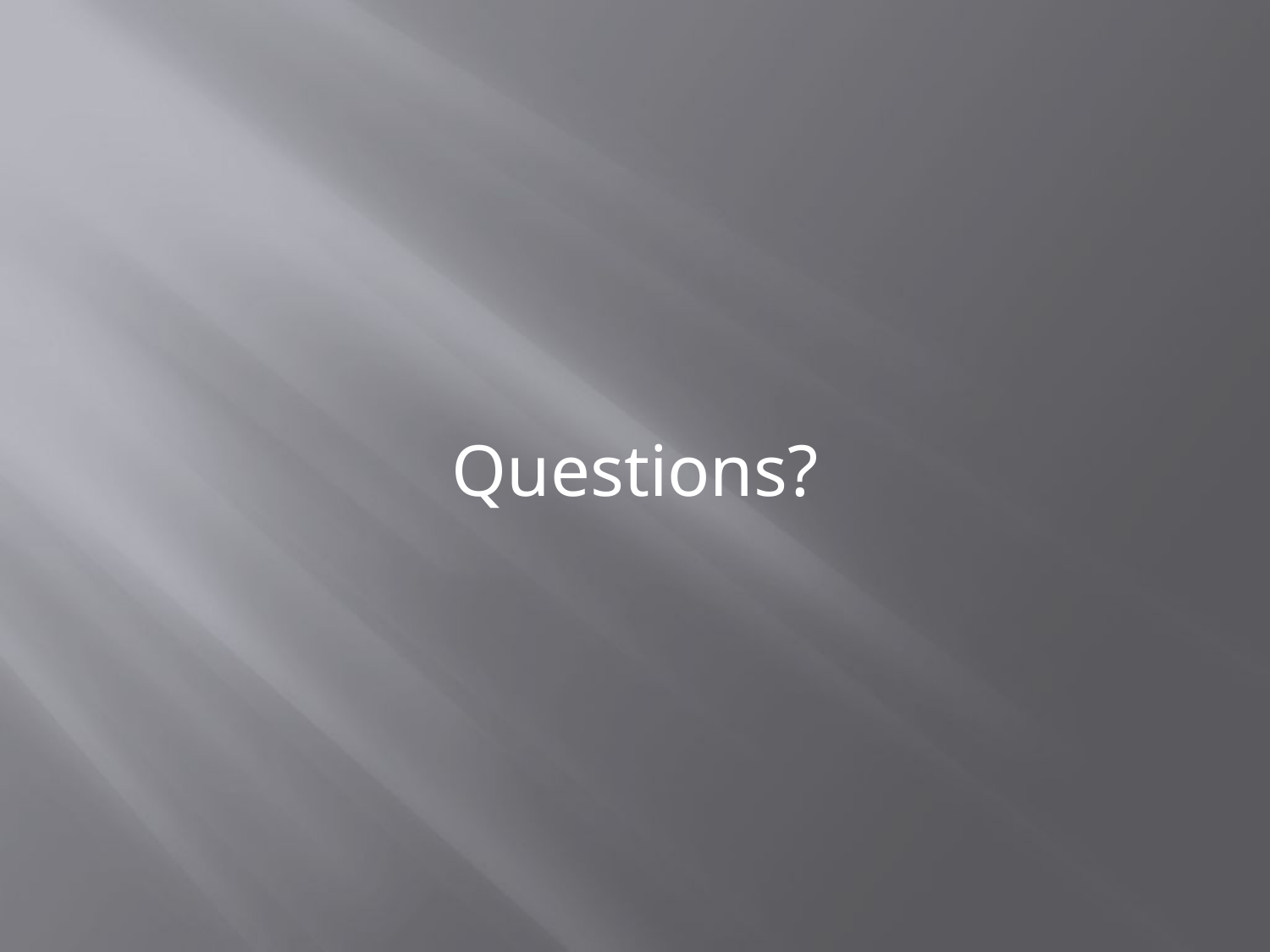

Questions?

## Slide 40
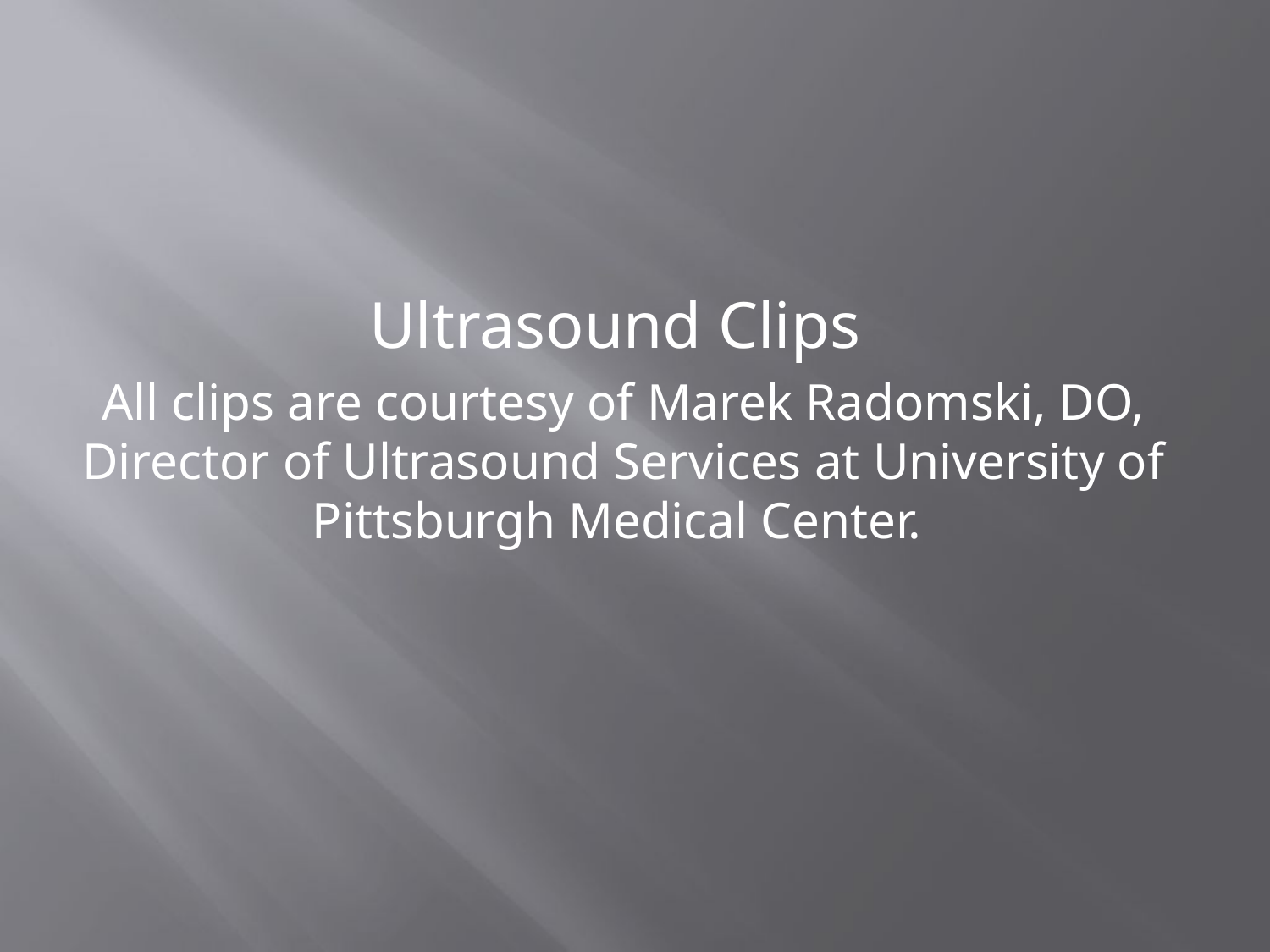

Ultrasound Clips
All clips are courtesy of Marek Radomski, DO, Director of Ultrasound Services at University of Pittsburgh Medical Center.
