# Supplementary material for: Use of Ultrasound in Introducing Anatomical Pathology to Preclinical Medical Students, in Correlation with Physical Exam Curricula
Source: MedEdPORTAL. 2020 Sep 25;16:10950. doi: 10.15766/mep_2374-8265.10950 (PMC7521063; doi:10.15766/mep_2374-8265.10950)
Supplement: Supplementary file 1 — Session 1 FAST Exam & the Trauma Patient.pptxSession 2 Cardiac and Lung.pptxSession 3 Gallbladder, Kidneys, & AAA.pptxSession 4 Ocular US & Central Access.pptxSession 1 Instructor Script.docxSession 2 Instructor Script.docxSession 3 Instructor Script.docxSession 4 Instructor Script.docxSurvey Questions.docx [file mep_2374-8265.10950-s001.zip › D. Session 4 Ocular US & Central Access.pptx]

## Slide 1
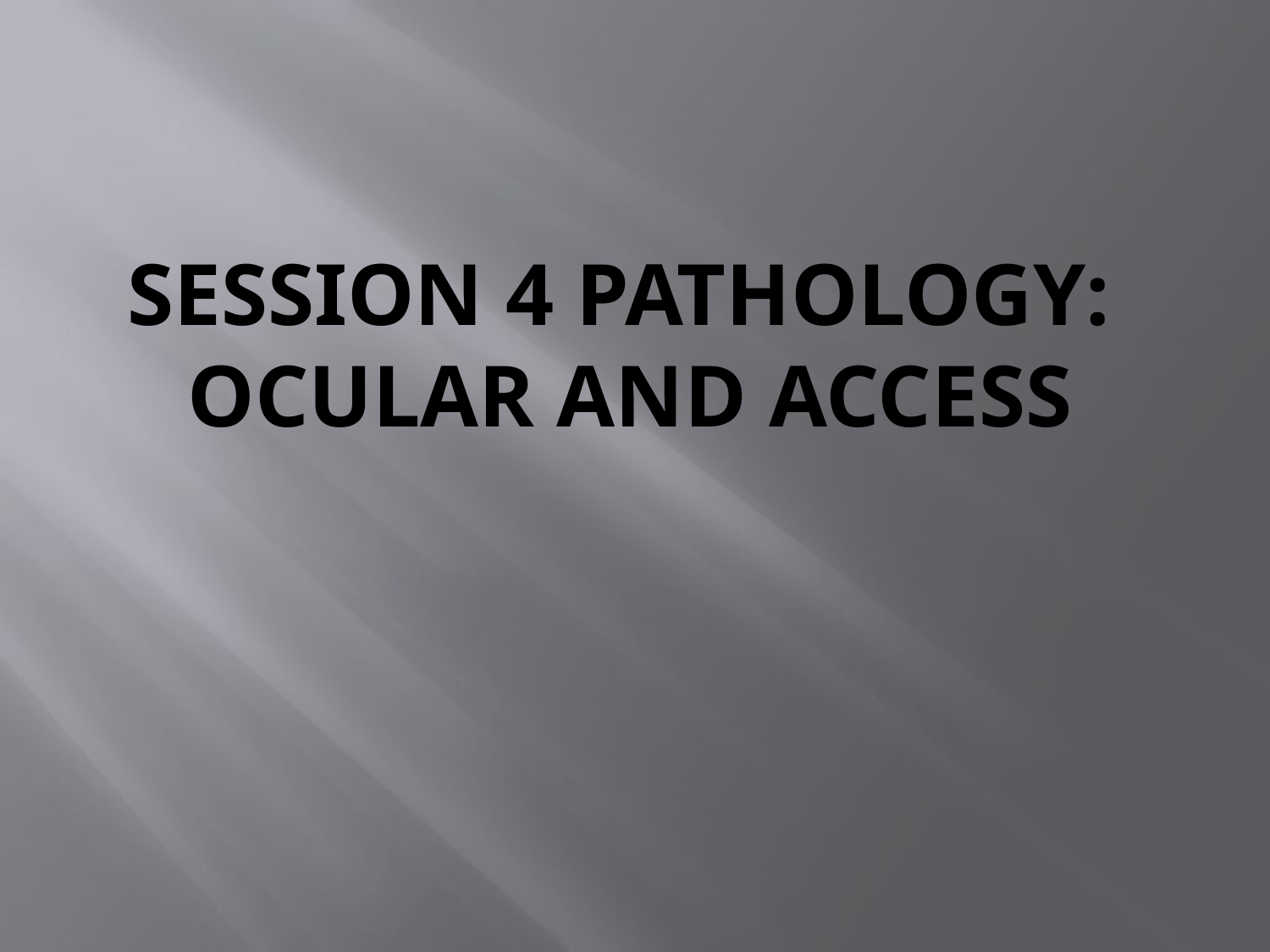

# Session 4 Pathology: Ocular and Access

## Slide 2
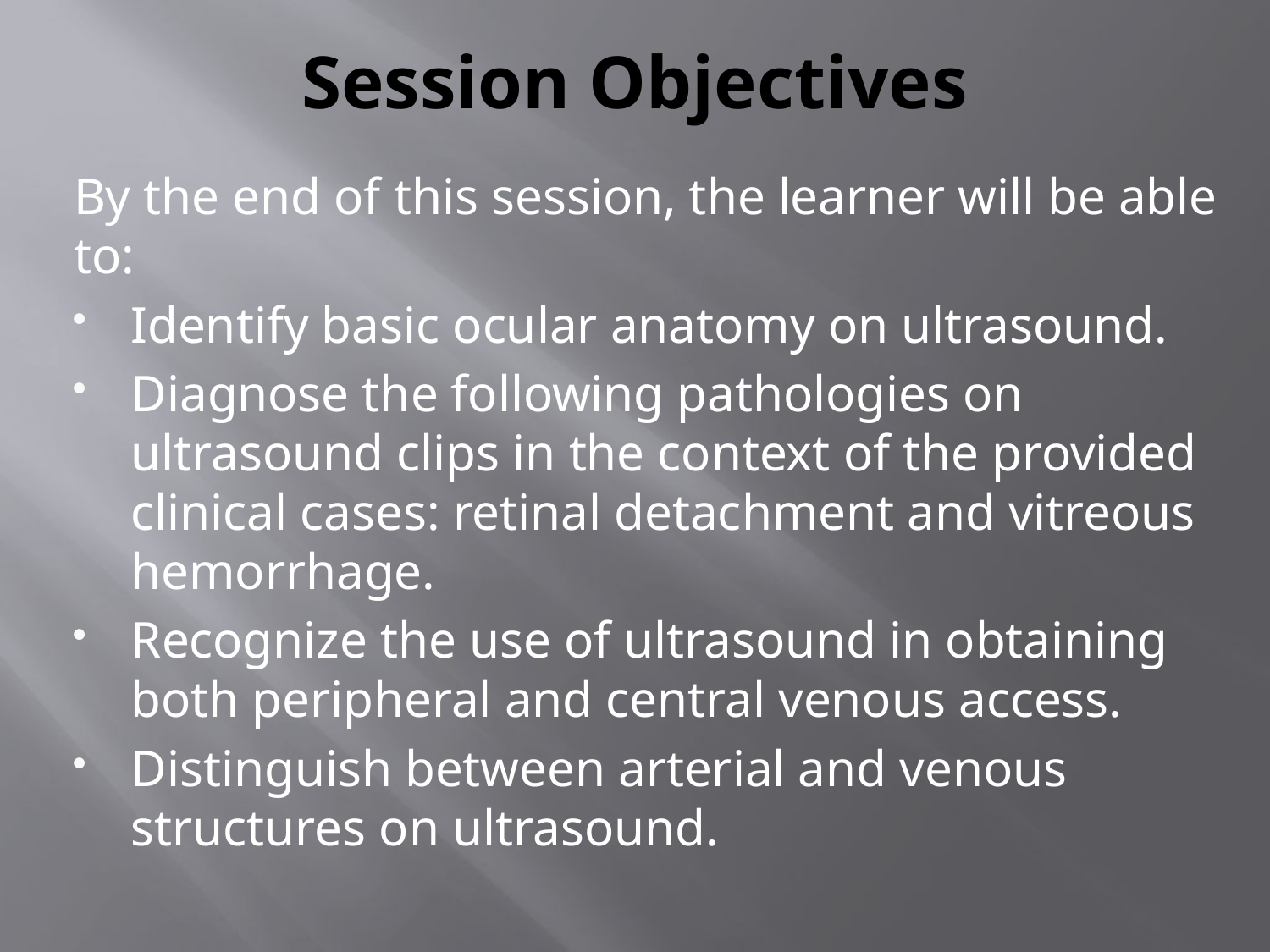

# Session Objectives
By the end of this session, the learner will be able to:
Identify basic ocular anatomy on ultrasound.
Diagnose the following pathologies on ultrasound clips in the context of the provided clinical cases: retinal detachment and vitreous hemorrhage.
Recognize the use of ultrasound in obtaining both peripheral and central venous access.
Distinguish between arterial and venous structures on ultrasound.

## Slide 3
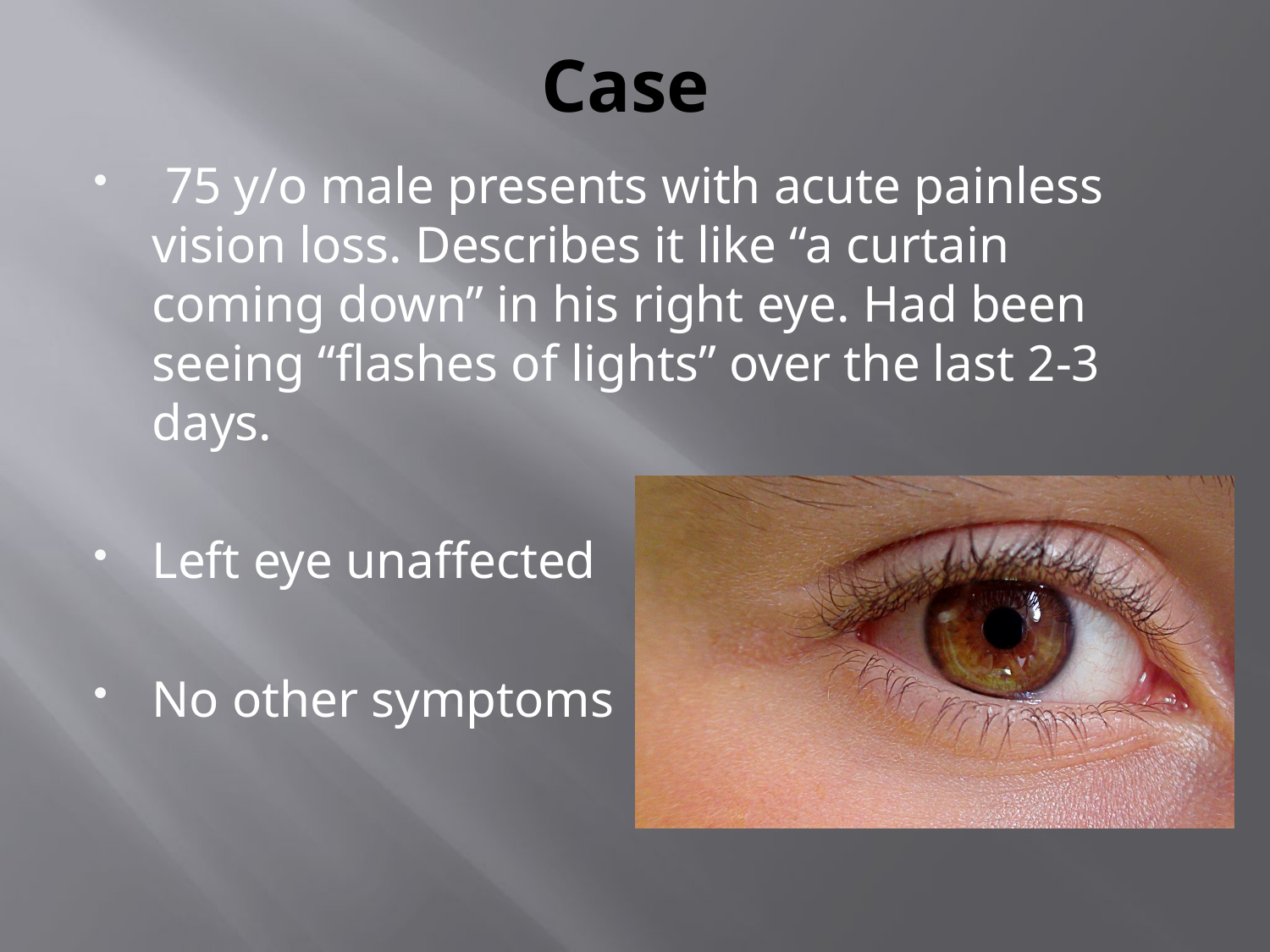

# Case
 75 y/o male presents with acute painless vision loss. Describes it like “a curtain coming down” in his right eye. Had been seeing “flashes of lights” over the last 2-3 days.
Left eye unaffected
No other symptoms

## Slide 4
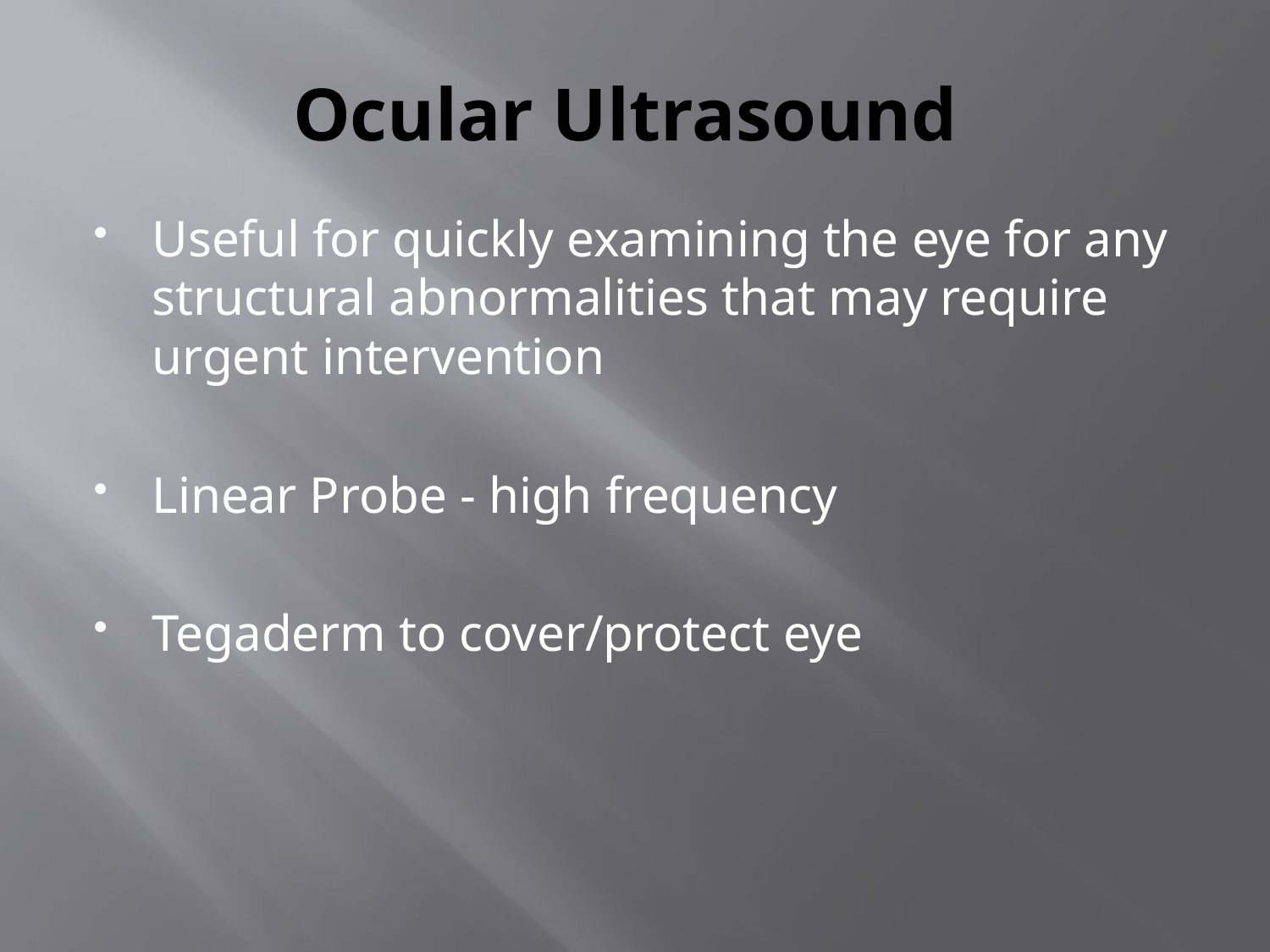

# Ocular Ultrasound
Useful for quickly examining the eye for any structural abnormalities that may require urgent intervention
Linear Probe - high frequency
Tegaderm to cover/protect eye

## Slide 5
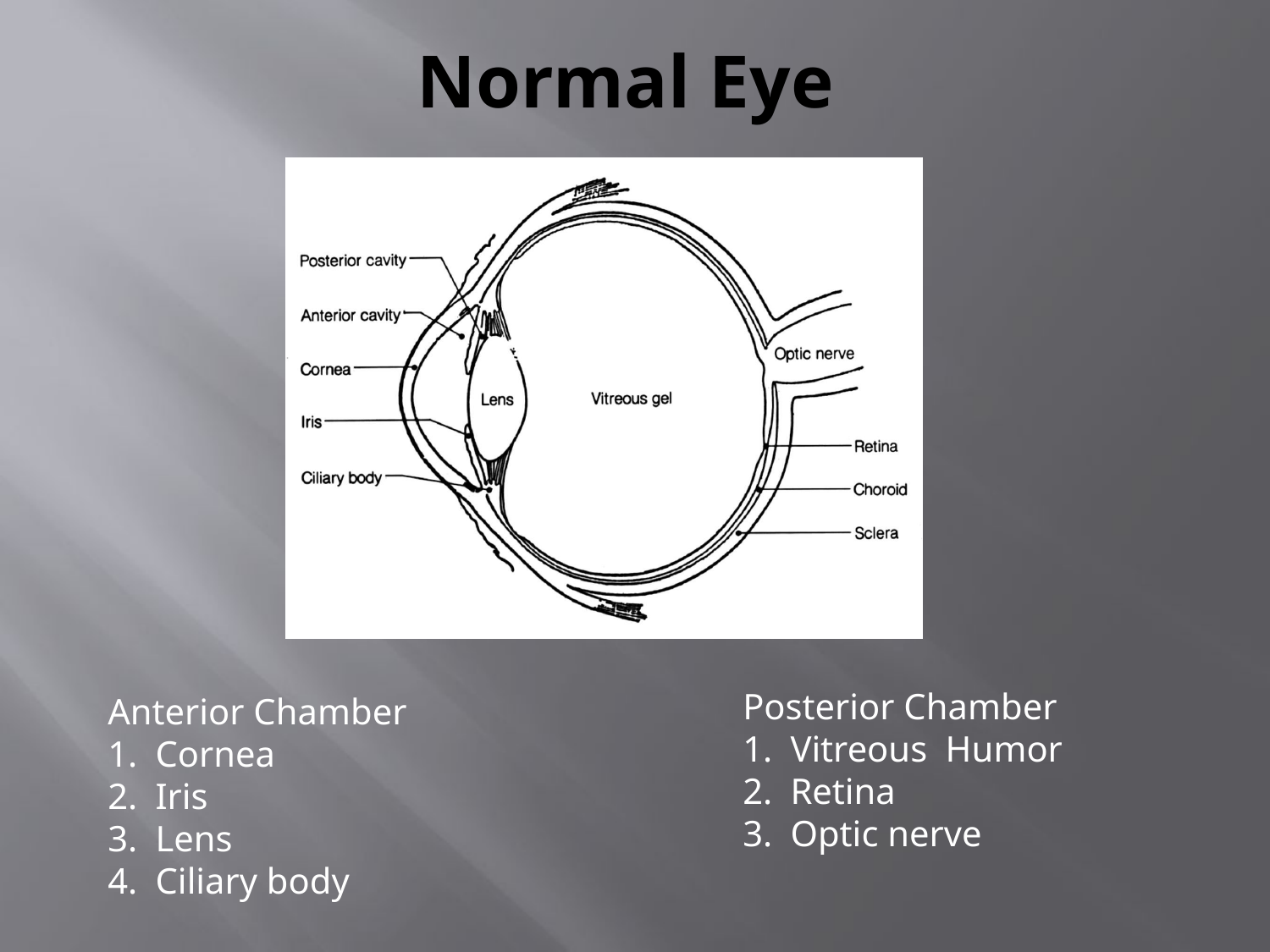

# Normal Eye
Posterior Chamber
Vitreous Humor
Retina
Optic nerve
Anterior Chamber
Cornea
Iris
Lens
Ciliary body

## Slide 6
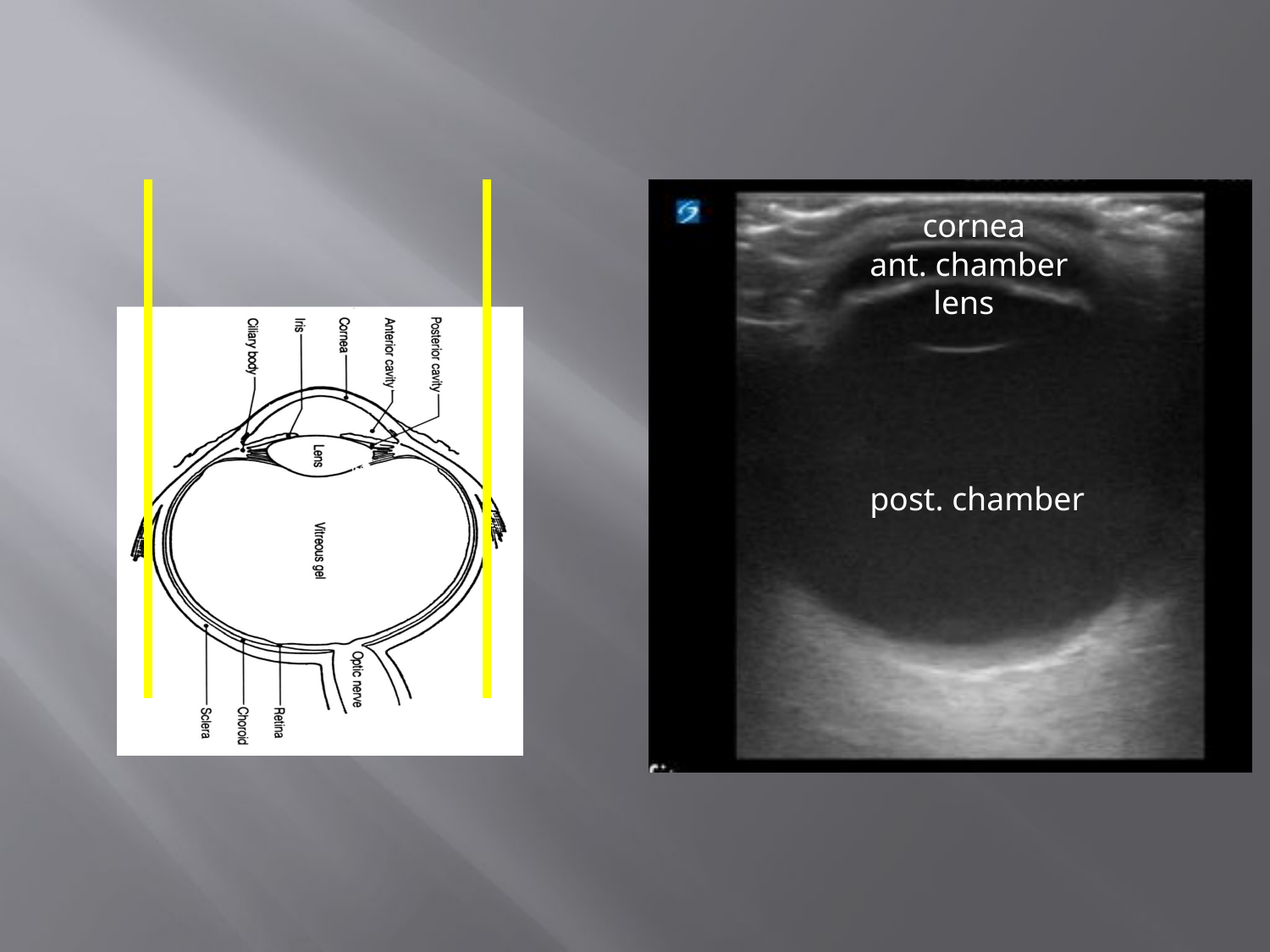

cornea
ant. chamber
lens
post. chamber

## Slide 7
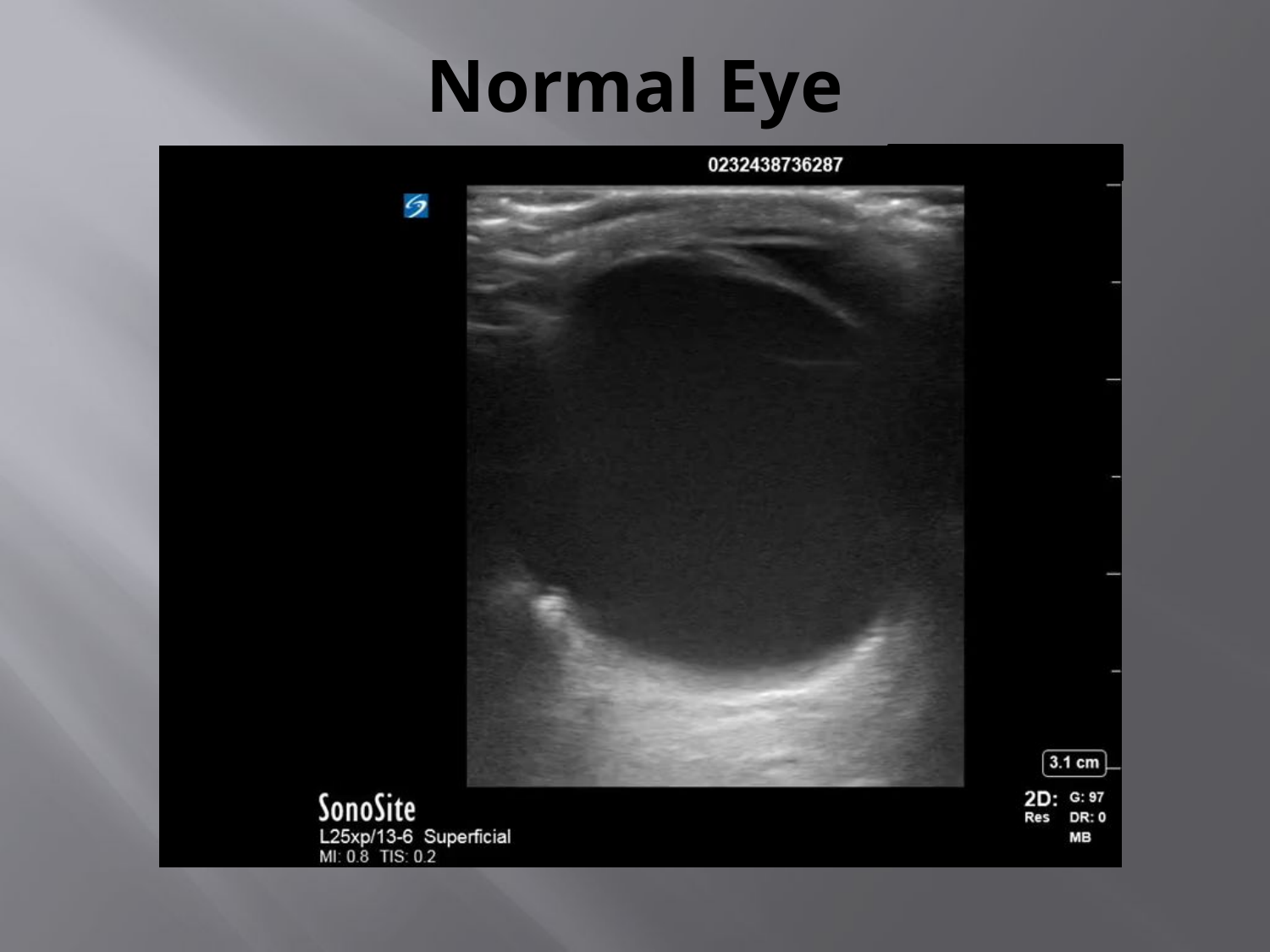

# Normal Eye

## Slide 8
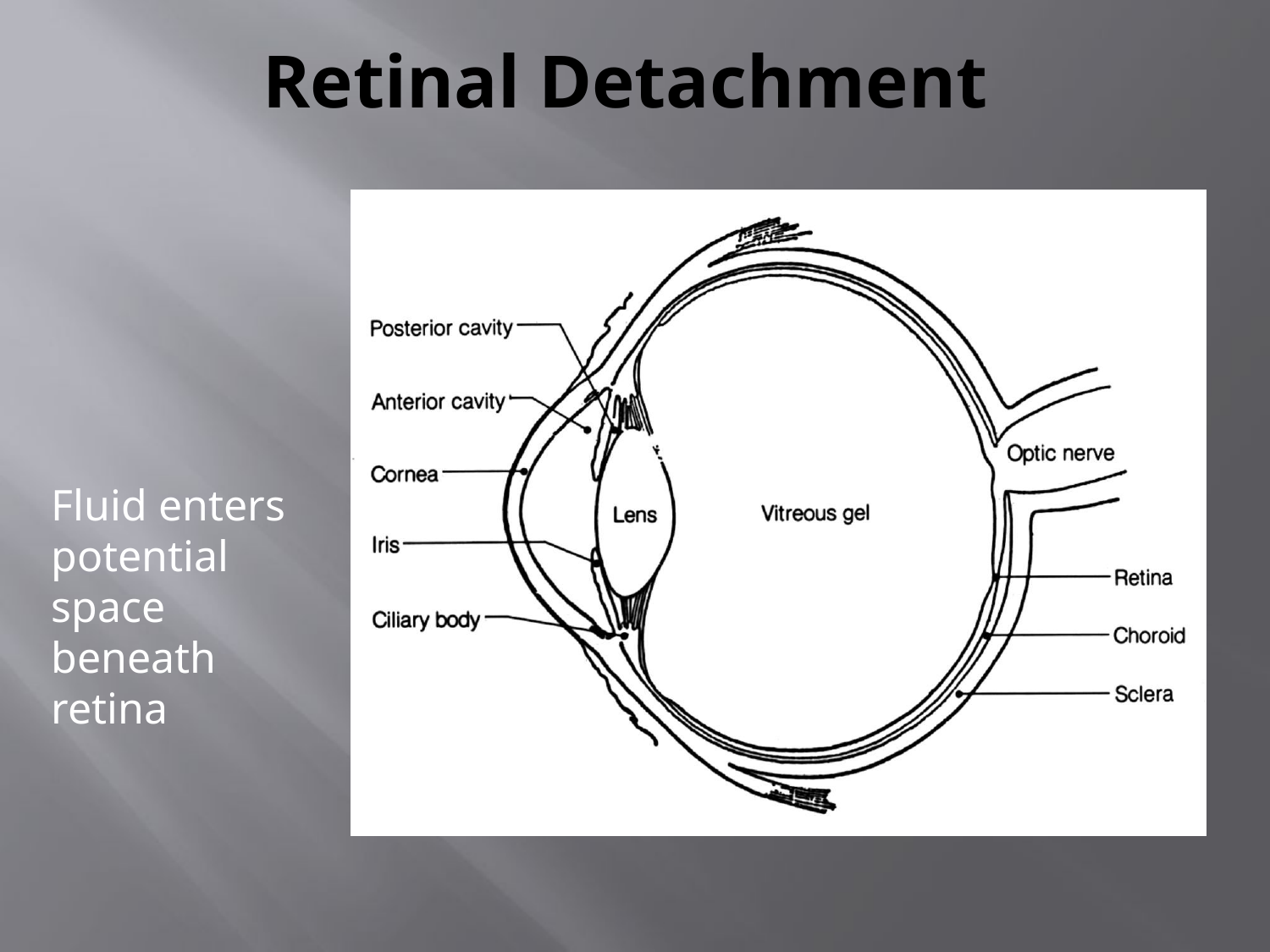

# Retinal Detachment
Fluid enters potential space beneath retina

## Slide 9
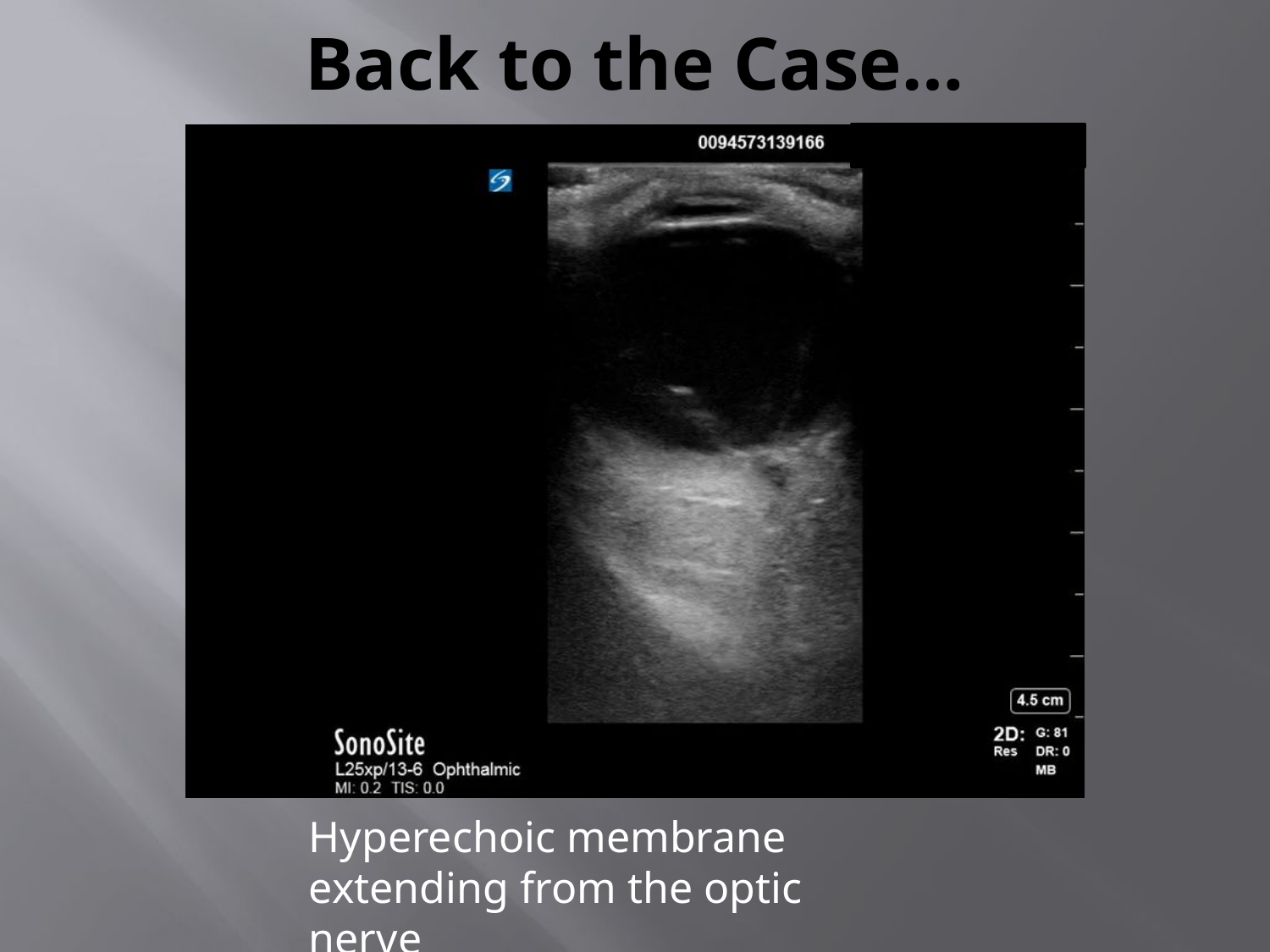

# Back to the Case…
Hyperechoic membrane extending from the optic nerve

## Slide 10
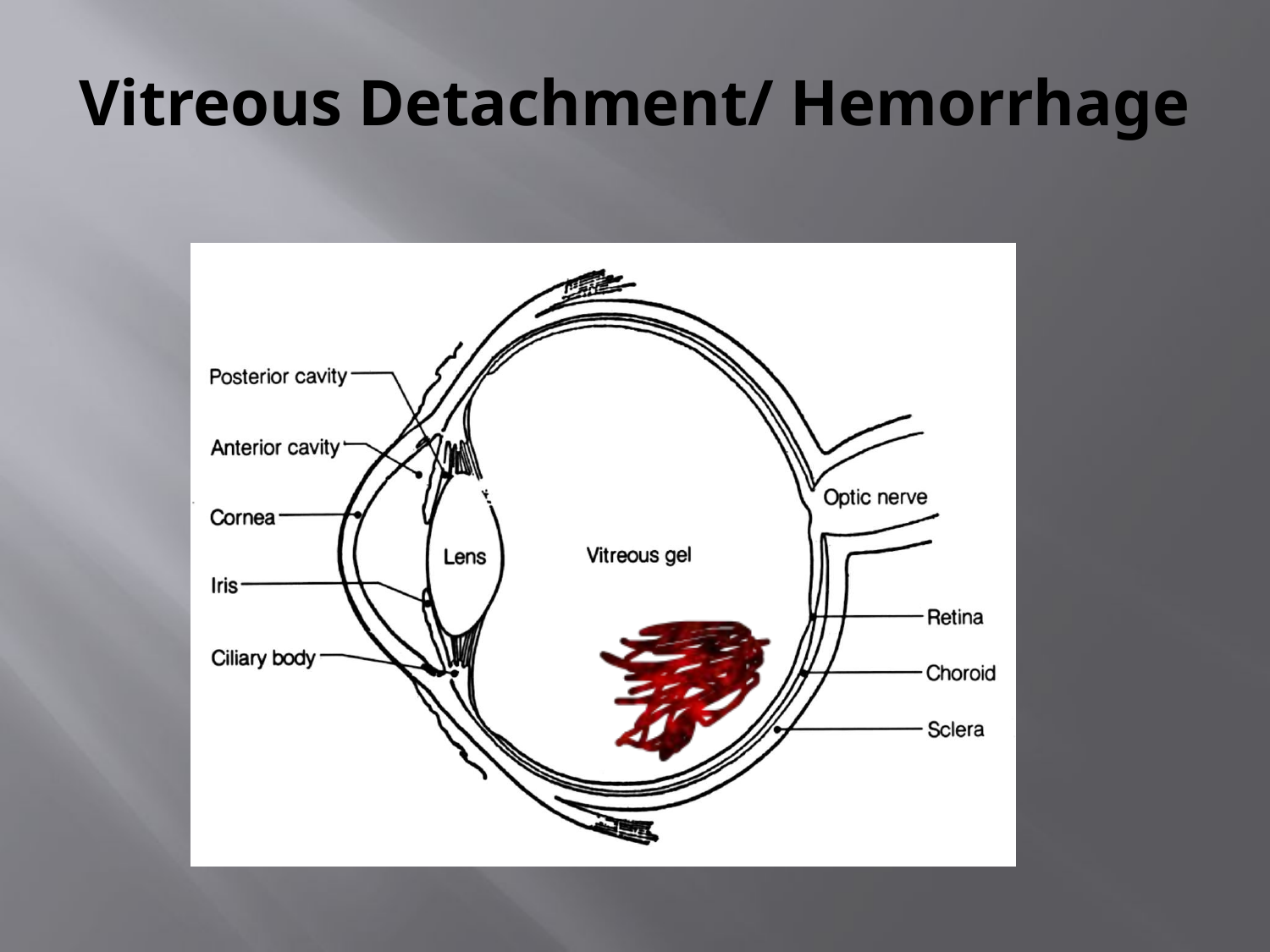

# Vitreous Detachment/ Hemorrhage

## Slide 11
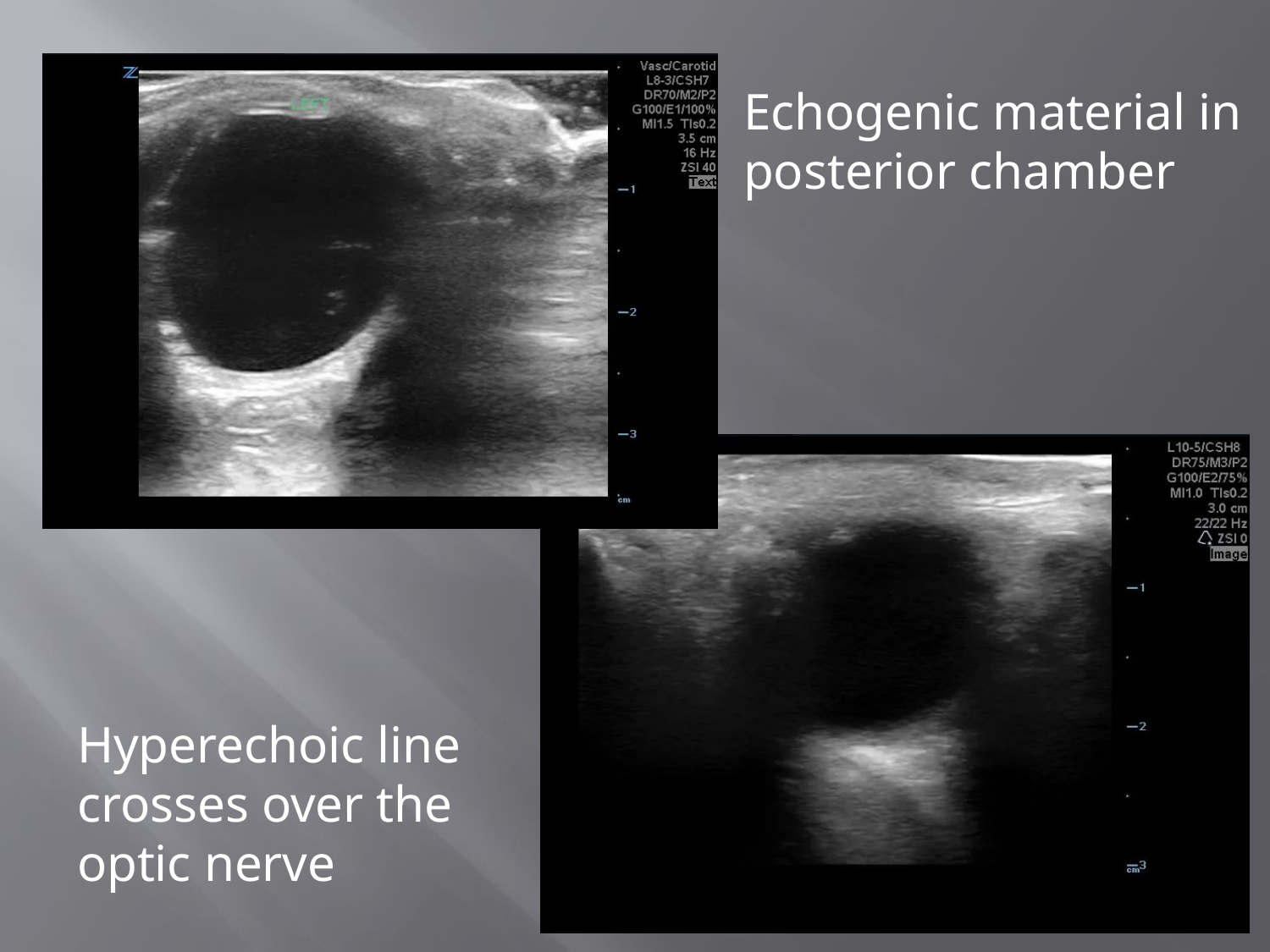

Echogenic material in posterior chamber
Hyperechoic line crosses over the optic nerve

## Slide 12
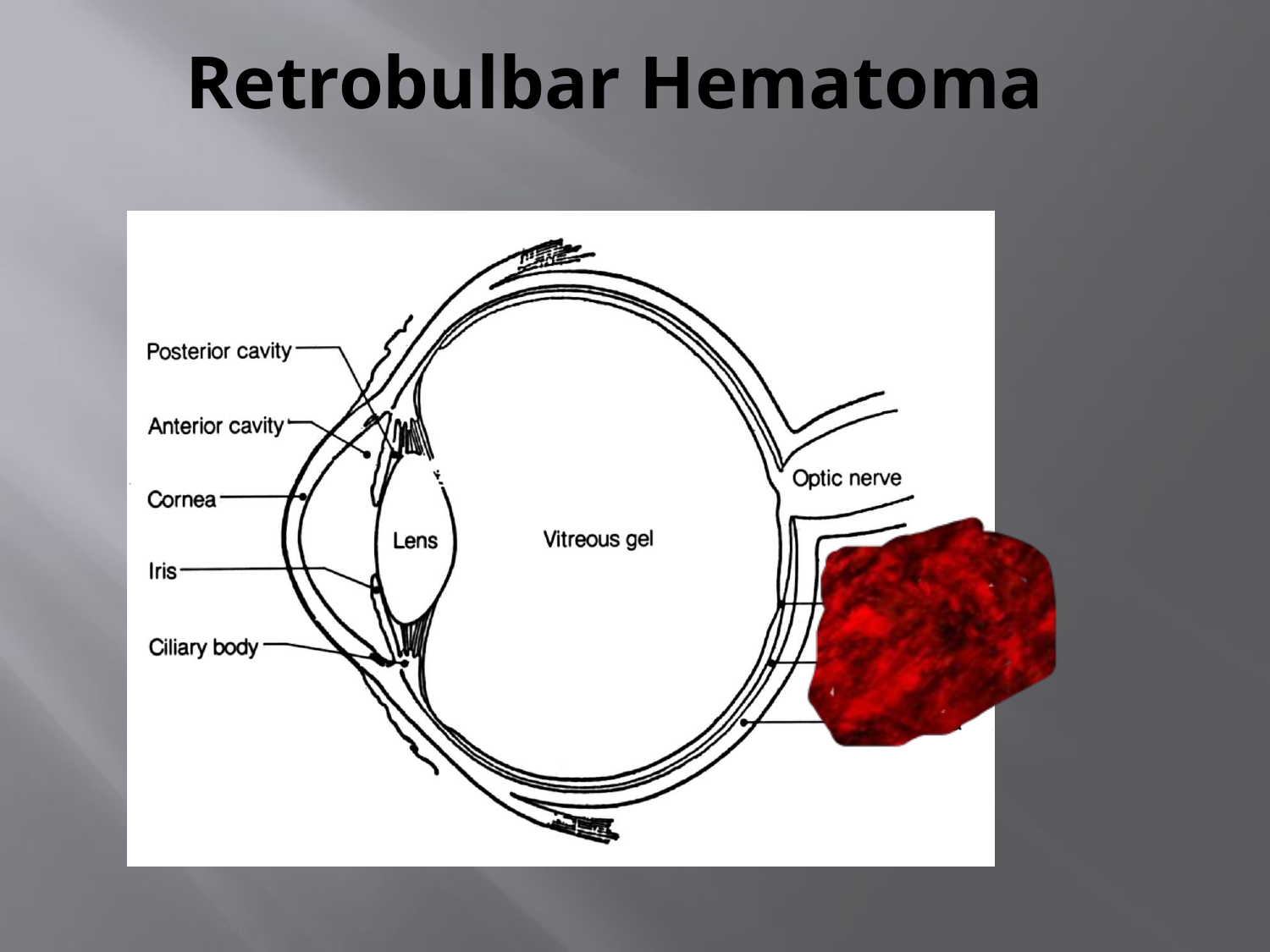

# Retrobulbar Hematoma

## Slide 13
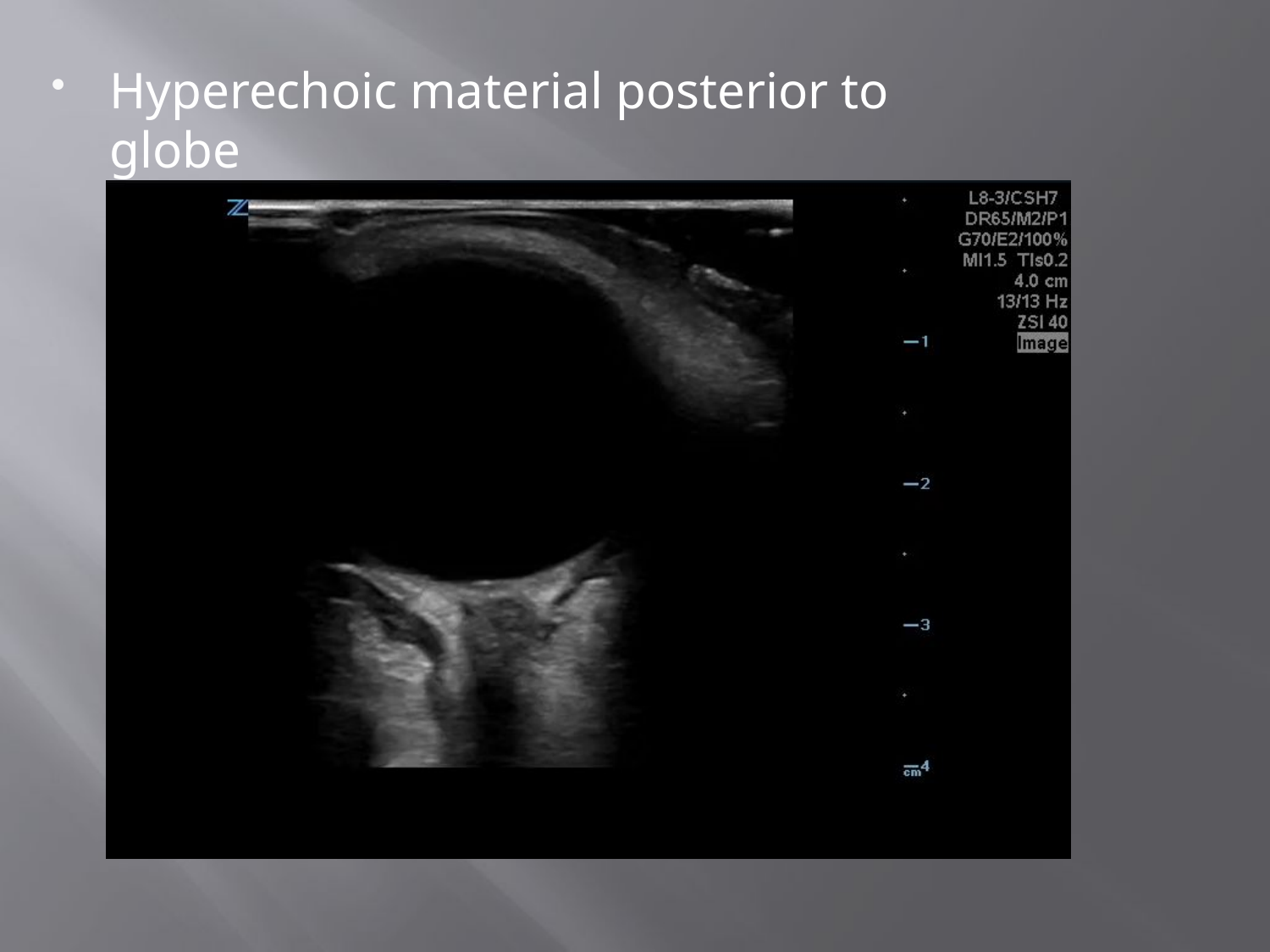

Hyperechoic material posterior to globe

## Slide 14
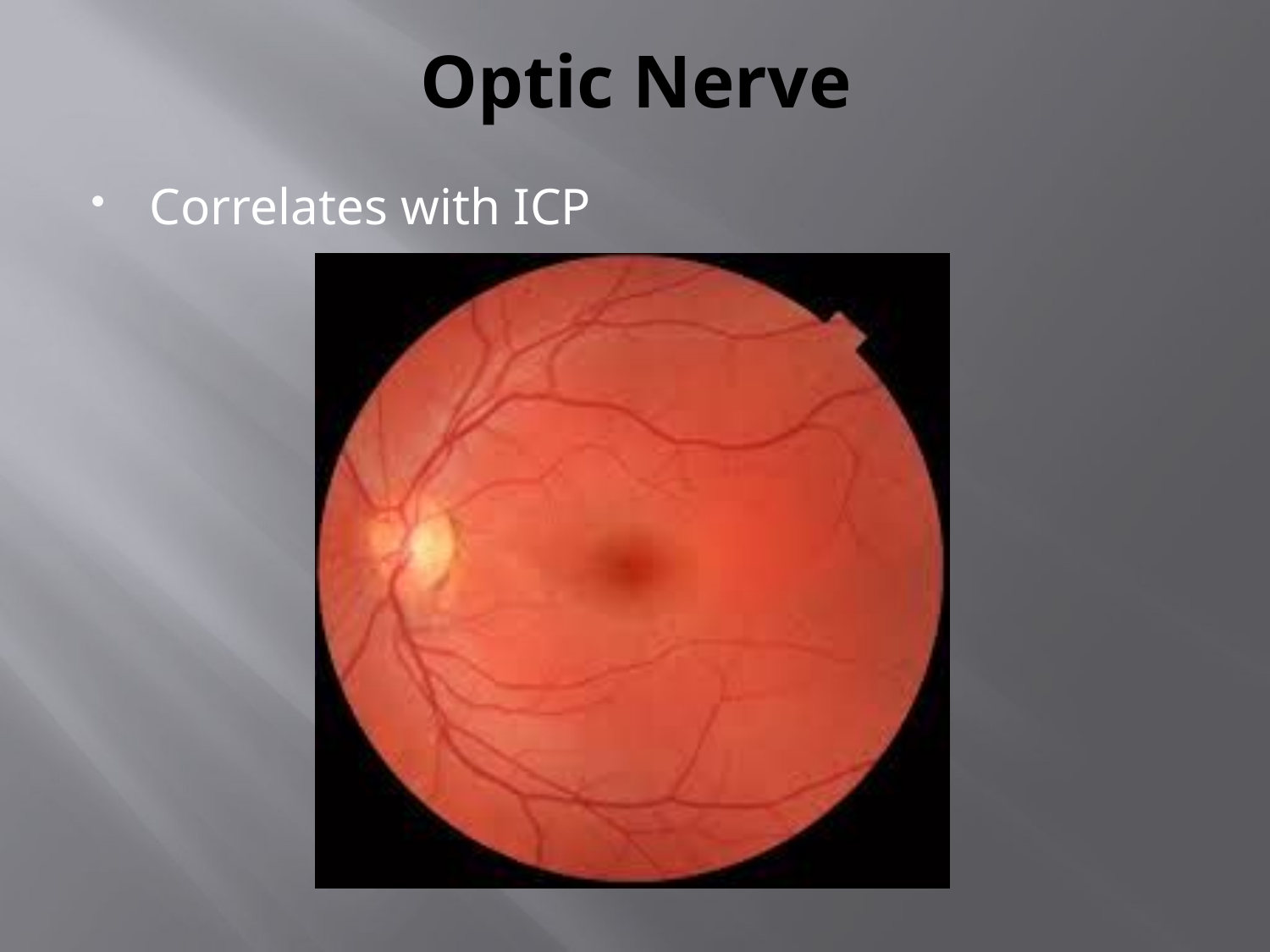

# Optic Nerve
Correlates with ICP

## Slide 15
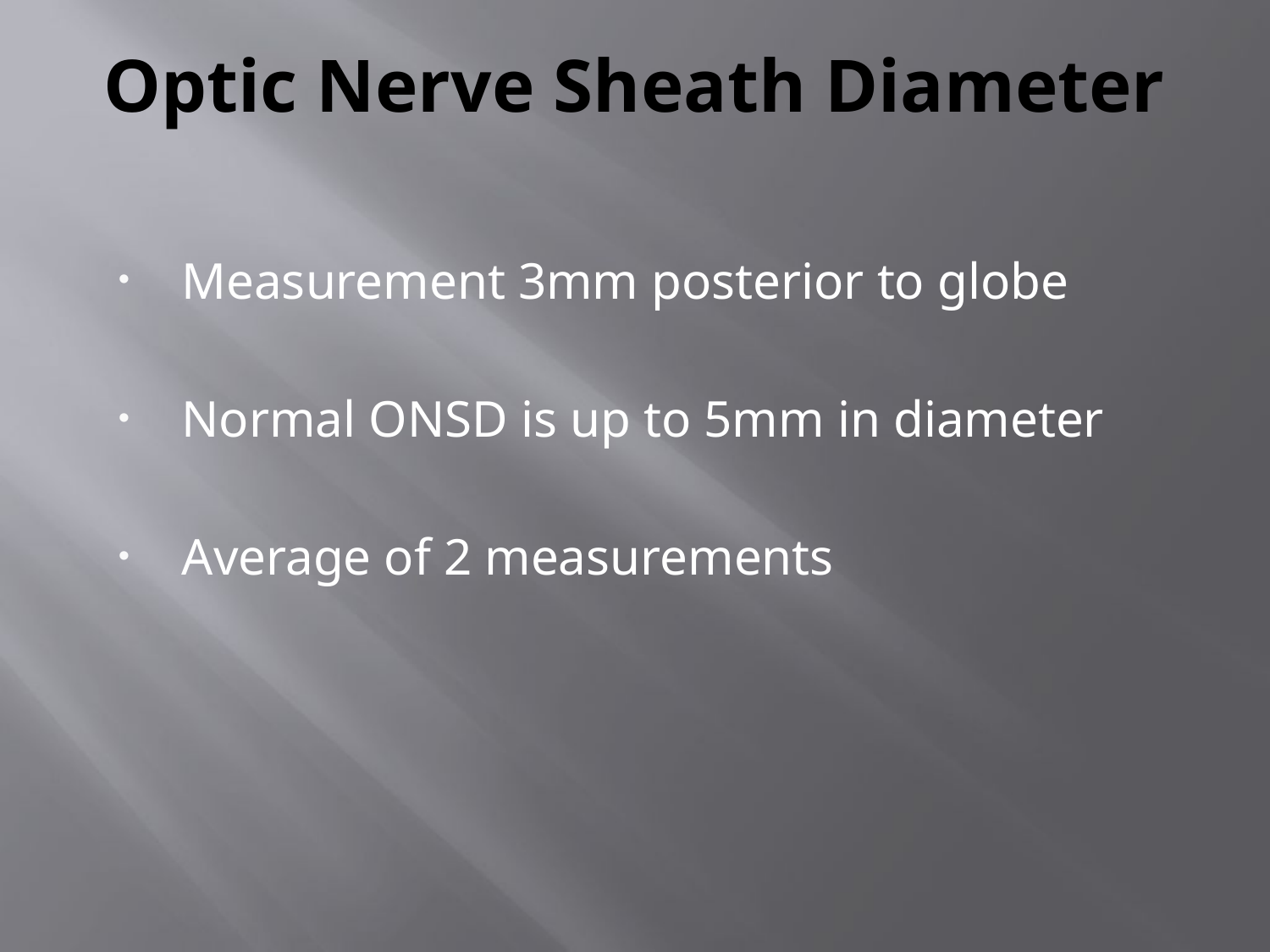

# Optic Nerve Sheath Diameter
Measurement 3mm posterior to globe
Normal ONSD is up to 5mm in diameter
Average of 2 measurements

## Slide 16
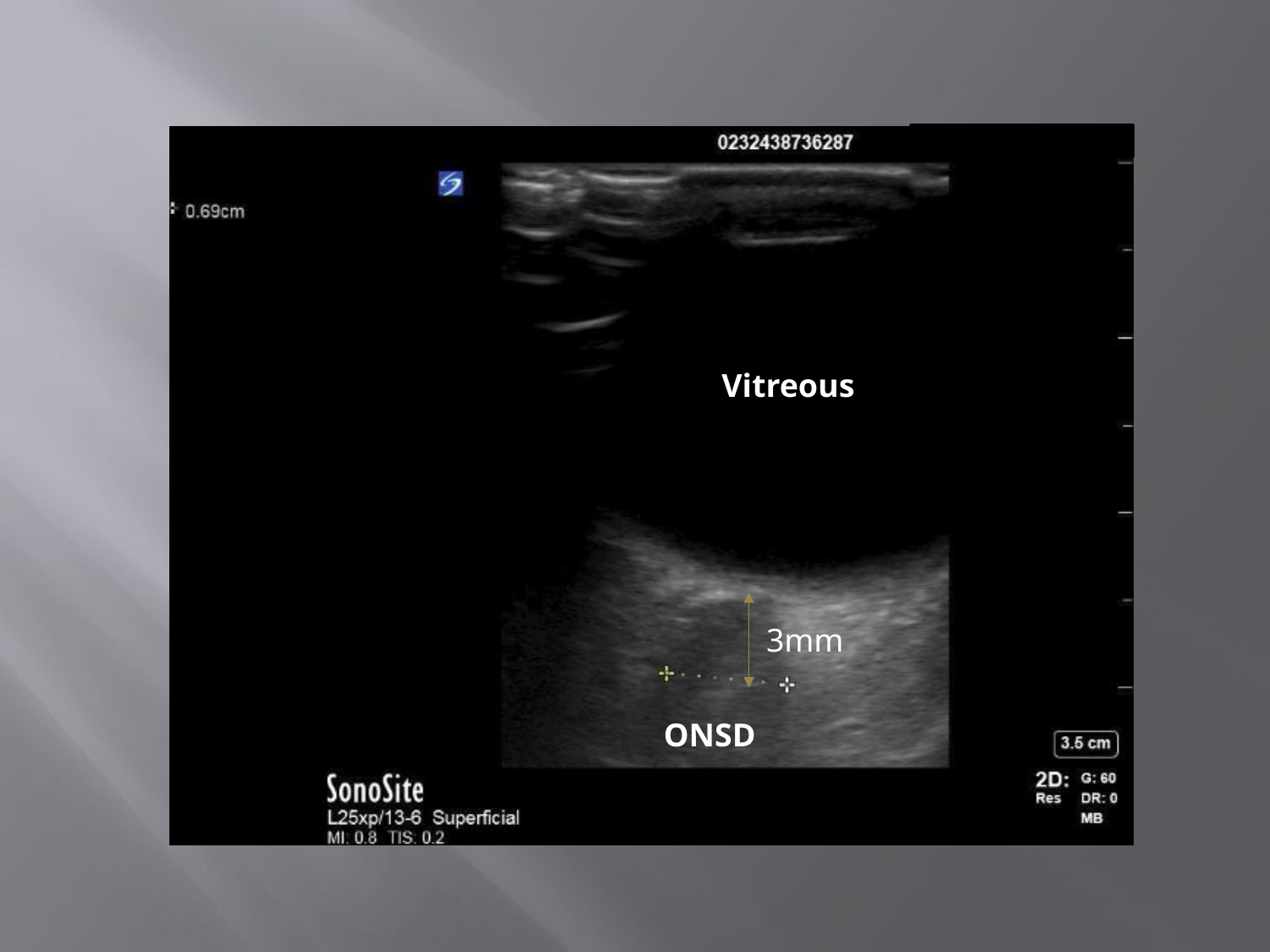

Vitreous
3mm
ONSD

## Slide 17
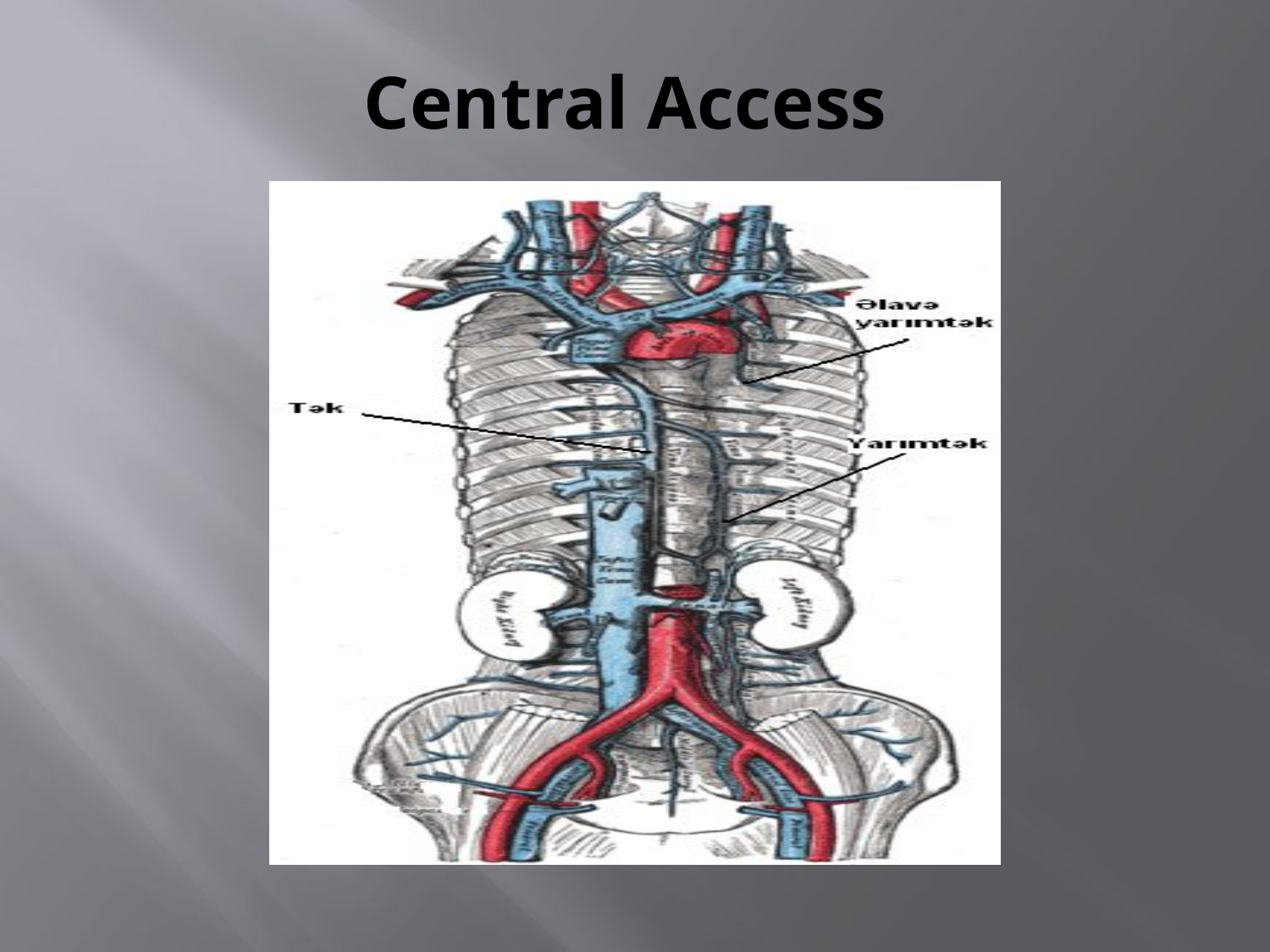

# Central Access

## Slide 18
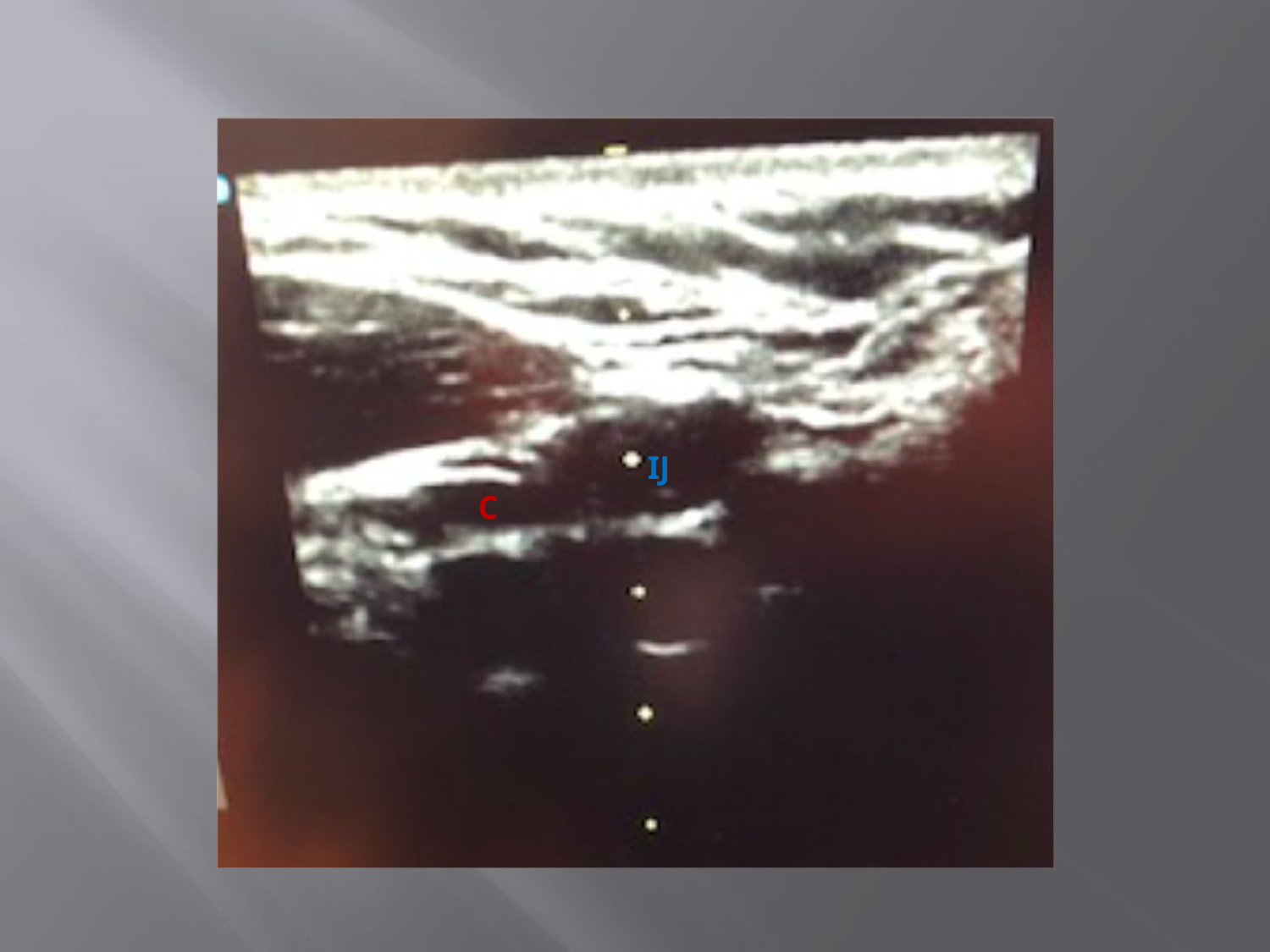

IJ
C

## Slide 19
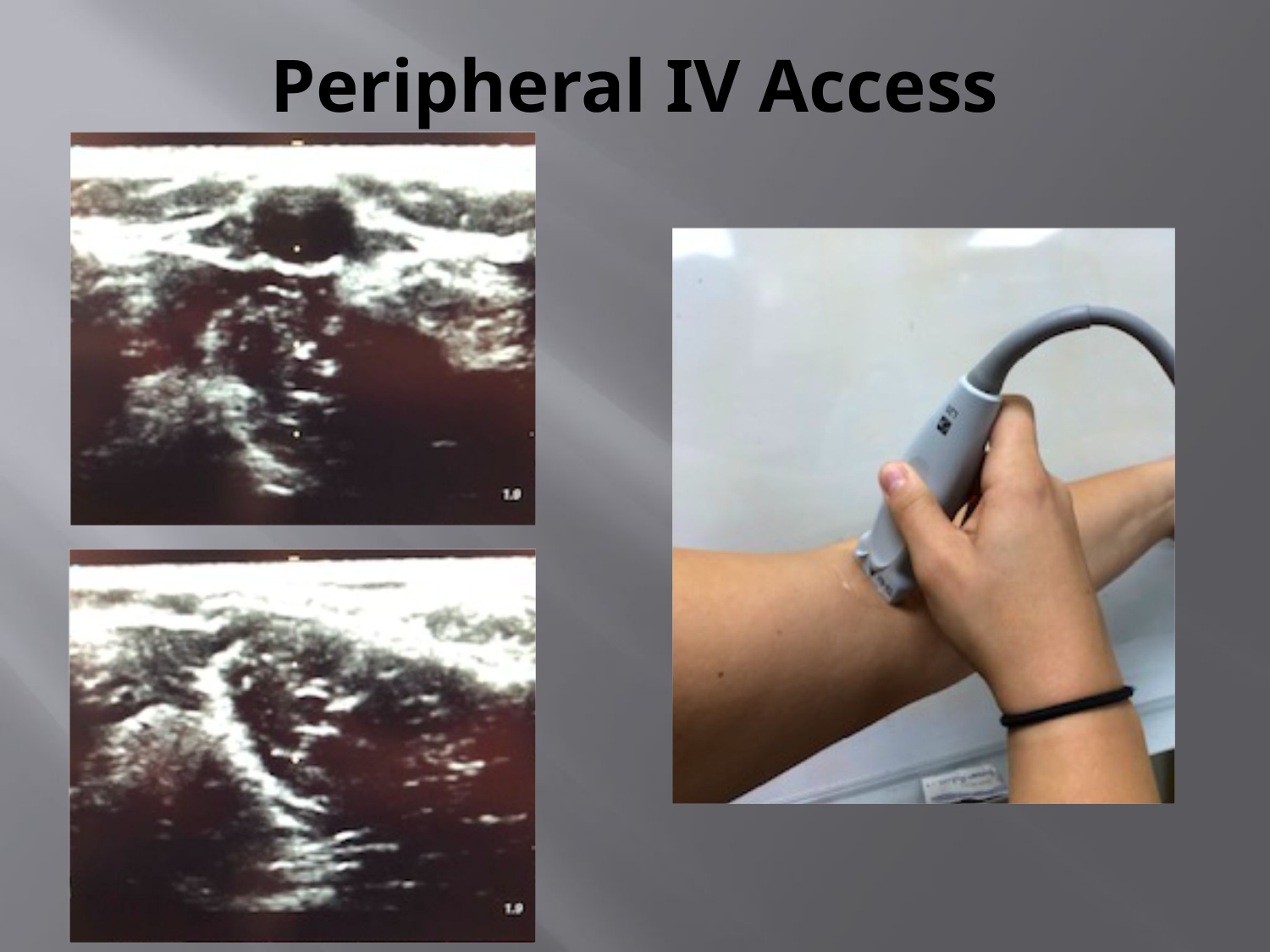

# Peripheral IV Access

## Slide 20
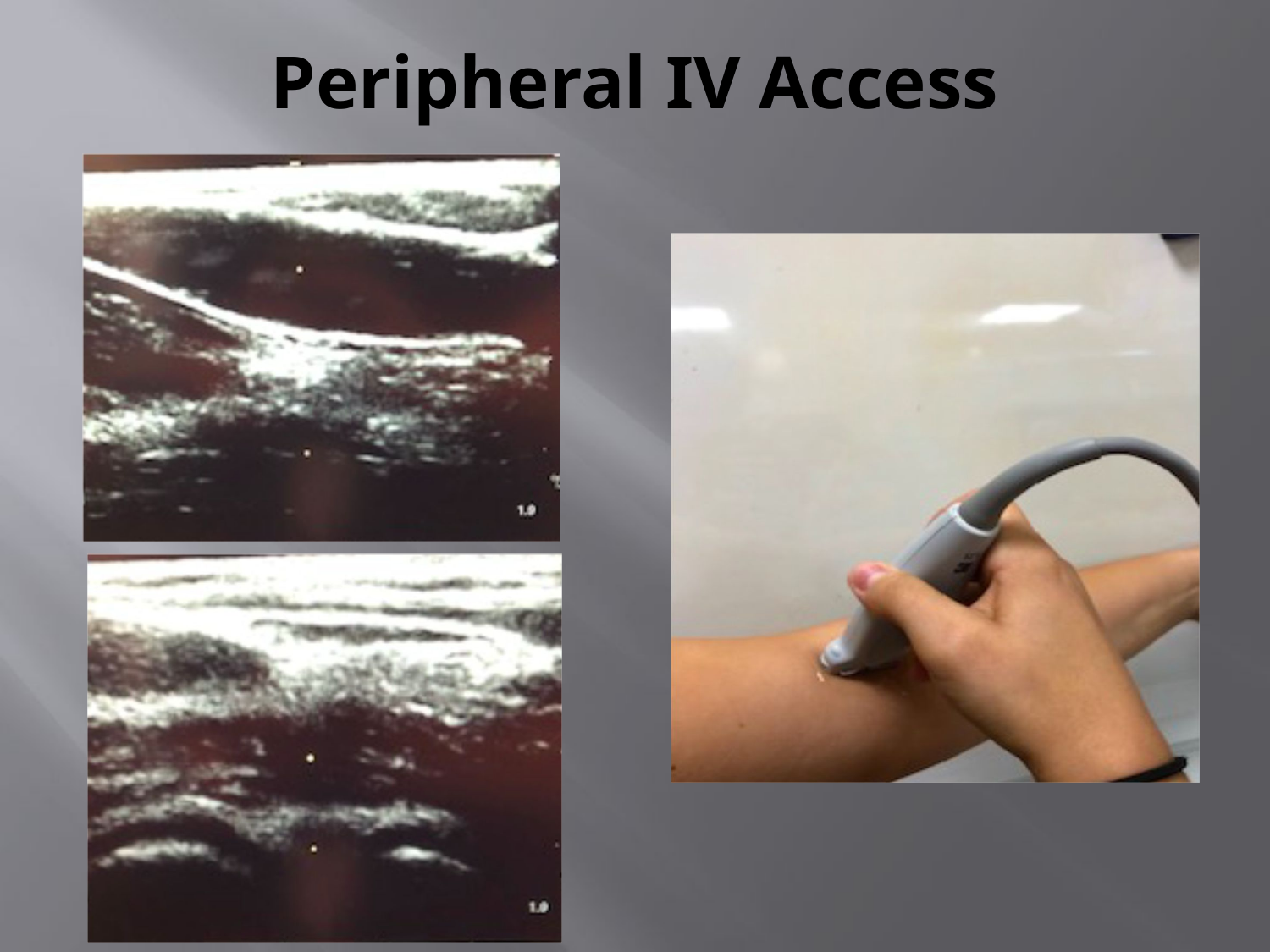

# Peripheral IV Access

## Slide 21
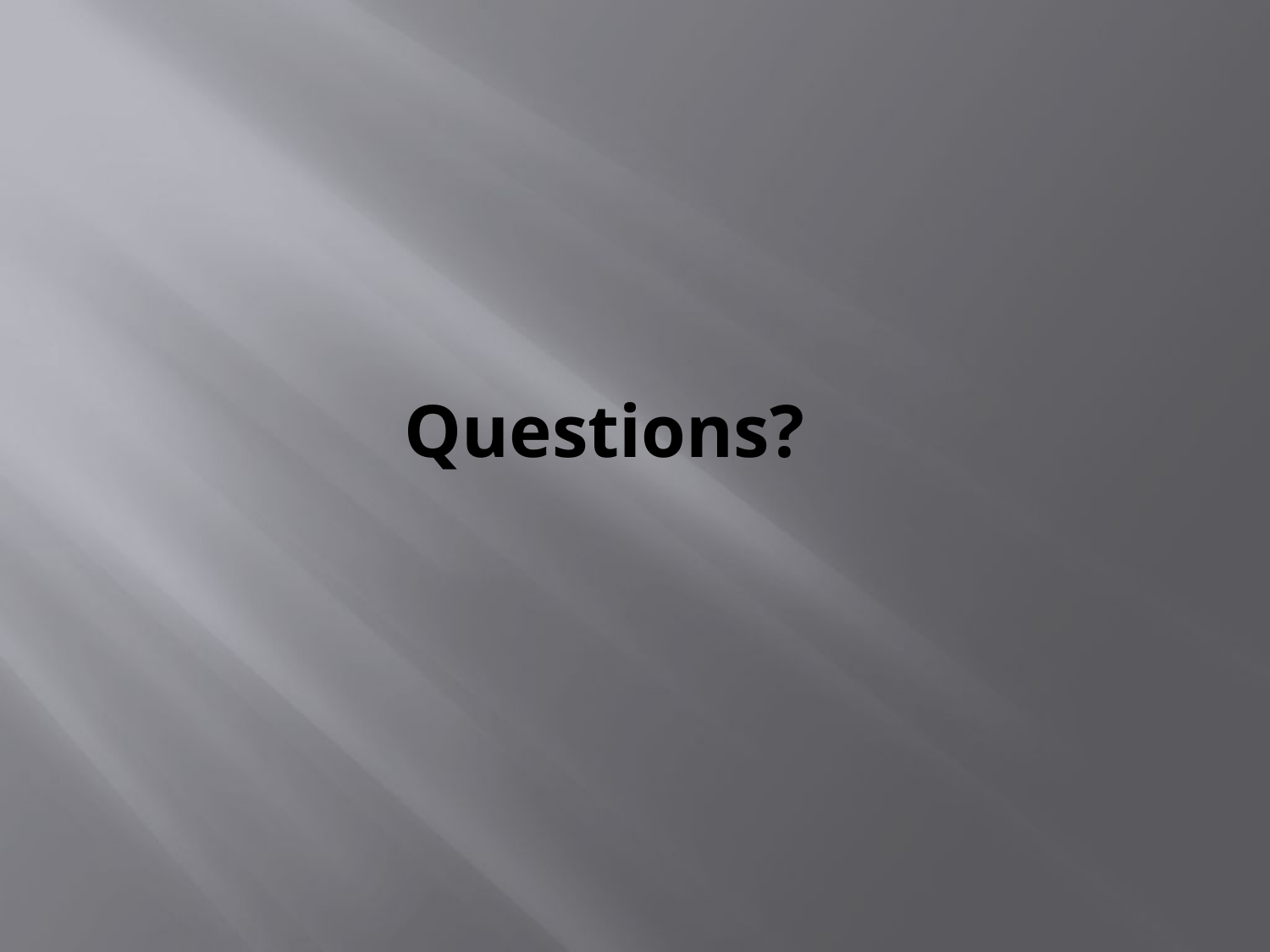

# Questions?

## Slide 22
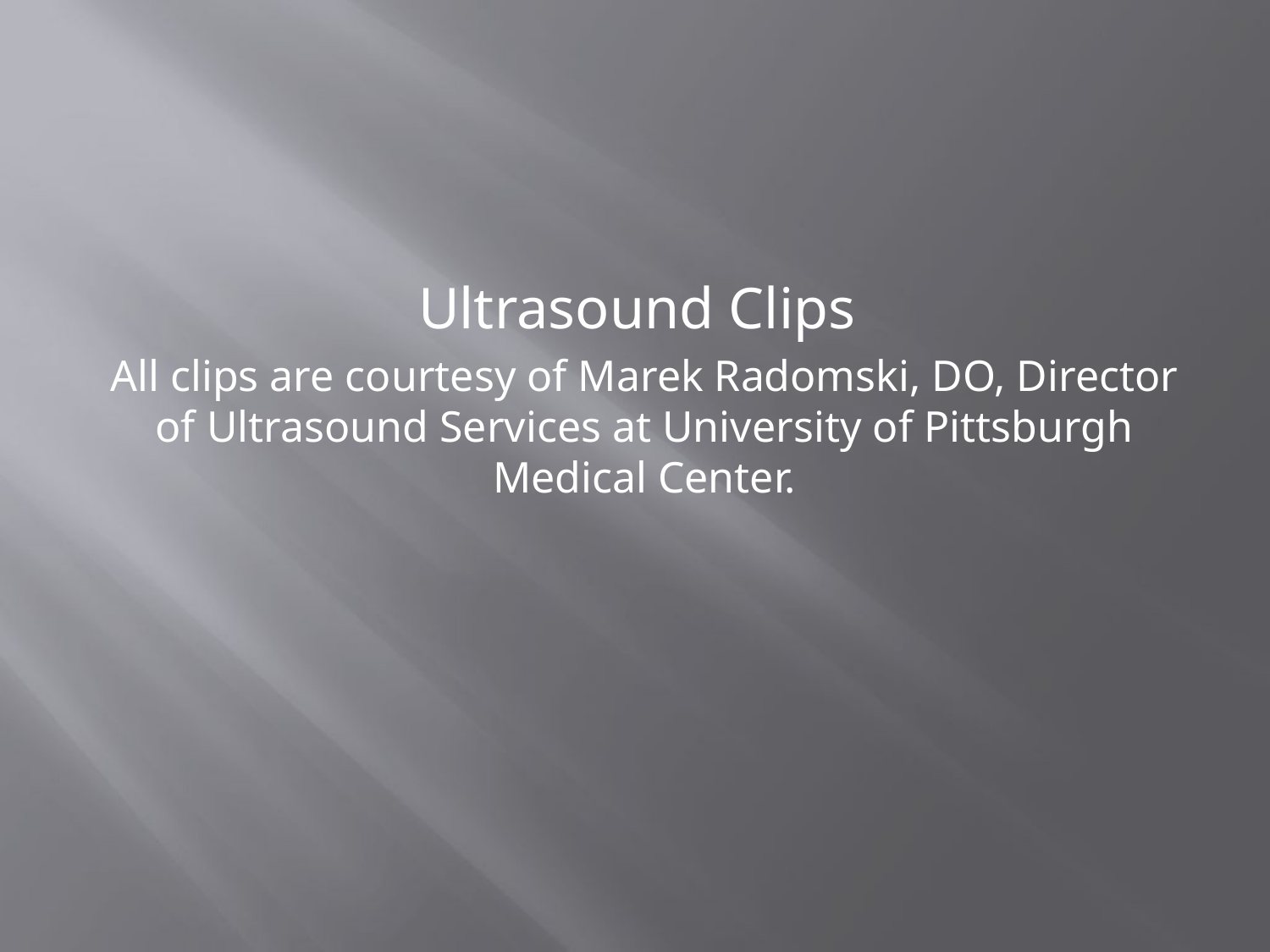

#
Ultrasound Clips
All clips are courtesy of Marek Radomski, DO, Director of Ultrasound Services at University of Pittsburgh Medical Center.
