# Supplementary material for: Use of Ultrasound in Introducing Anatomical Pathology to Preclinical Medical Students, in Correlation with Physical Exam Curricula
Source: MedEdPORTAL. 2020 Sep 25;16:10950. doi: 10.15766/mep_2374-8265.10950 (PMC7521063; doi:10.15766/mep_2374-8265.10950)
Supplement: Supplementary file 1 — Session 1 FAST Exam & the Trauma Patient.pptxSession 2 Cardiac and Lung.pptxSession 3 Gallbladder, Kidneys, & AAA.pptxSession 4 Ocular US & Central Access.pptxSession 1 Instructor Script.docxSession 2 Instructor Script.docxSession 3 Instructor Script.docxSession 4 Instructor Script.docxSurvey Questions.docx [file mep_2374-8265.10950-s001.zip › E. Session 1 Instructor Script.docx]

**Session 1 Pathology: Ultrasound in the Examination of the Trauma Patient**

Instructor PowerPoint Script

Slide 1

This course serves multiple purposes. First, it is designed to further enhance your learning of the physical exam by using ultrasound. It will also expose you to some anatomical pathology, which will help you more easily distinguish between normal and abnormal anatomical findings. Finally, this course will introduce and expose you to the many uses of ultrasound in clinical practice through clinical case-based scenarios.

Slide 2

Session objectives

This session will introduce you to the FAST exam and its pivotal role in the evaluation in the trauma patient. By the end of this session, you should be able to list the four different components of the FAST exam, identify the major abdominal organs within each view of the FAST exam, and recognize pathologic findings on each view. Two (hypothetical) patients will be presented here, and it will be up to you to diagnose them based on their ultrasound findings.

Slide 3

We will begin with the case of our first patient. The patient is a 45 year-old male coming in as a Level 1 trauma (most acute) to your Emergency Department (ED). He has fallen 30 feet off of some scaffolding. He landed on his right side and did lose consciousness. This is considered to be a significant mechanism of injury, and we should be highly concerned for internal injuries.

Primary survey - This is your ABCs of trauma: airway (patient is talking to you), breathing (you hear equal breath sounds), and circulation (has intact radial pulses and no sources of active external bleeding).

Secondary survey - this is your head to toe exam of the patient, done to quickly identify any injuries. On this patient’s secondary survey, he has an obvious deformity of his right wrist and his abdomen is distended and diffusely tender to palpation.

Vitals: What do you think? Are they normal or abnormal? Is this patient sick or not sick?

BP 90/54 (abnormal- hypotensive), HR 126 (abnormal- tachycardic), RR 18 (normal), oxygen saturation 98% (normal).

This patient is sick. He has a significant mechanism for trauma (30 ft. fall) and has an abnormal abdominal exam. His vitals are also abnormal, and his decreased blood pressure and increased heart rate are likely due to bleeding within his abdomen.

Slide 4

For trauma patients, often the best way to identify internal injuries is by obtaining imaging, most often CT. However, the CT scanner is often out of the department or in another location that the patient will have to be moved to. Ideally, you want to make sure that the patient will not acutely decompensate in the CT scanner.

So, for this patient, what do you think? Is he ready to go to the CT scanner? We have already identified this patient as having abnormal vital signs. His hypotension and tachycardia, along with our suspicion that he may have intra-abdominal bleeding, makes this patient very unstable. CT will likely take this patient out of the department. Further, although CT typically does not take very long, the time it would take to obtain a CT in this unstable patient is still too long. Therefore, this patient should NOT go to CT.

Slide 5

So, we have identified that this patient is unstable and should NOT go to CT. How will we then determine what injuries he has? This is where ultrasound comes in. US is an excellent bedside tool that can quickly evaluate the trauma patient for evidence of intraperitoneal bleeding. The information obtained by using the US in the evaluation of this patient is absolutely vital to making sure he gets appropriate care. If intraperitoneal bleeding is detected, this patient will likely go right to the operating room for surgery.

Slide 6

The US exam conducted on the Trauma patient is the FAST exam, which stands for “Focused Assessment with Sonography in Trauma.” It is a rapid, bedside screening imaging exam to identify intra-abdominal free fluid or cardiac tamponade. As we have just learned, it is also an excellent diagnostic tool for unstable patients who should not go to CT and are likely to go to the operating room (OR) emergently.

Slide 7

The FAST exam consists of 4 different views/images: Right Upper Quadrant (RUQ), Left Upper Quadrant (LUQ), suprapubic view, and subxiphoid view.

Classically, you start with the RUQ view. However, depending on the patient and situation, you could start with the view where you are most concerned for injury. For example, in a patient who comes in with a stab wound to the chest, you would be most concerned about an injury to the heart, and therefore, you would start with the subxiphoid view, as this is the view that visualizes the heart.

Slide 8

Why 4 views? The FAST exam is used to examine the major abdominal organs likely to be injured in the trauma patient, such as the liver, spleen, and heart. The 4 views allow you to examine the most common places where blood from these injuries would be likely to collect in a patient lying supine (on their back).

Slide 9

Let’s quickly review the basics about the US probe. For the FAST exam, you will be using the curvilinear probe. The indicator of the probe should always be facing either towards the patient’s head when held in the sagittal position, or towards the patient’s right when in the transverse position (refer to the pictures on the slide). This will help to keep the orientation of the images standardized, to easily allow anyone to correctly interpret them.

Slide 10

The first view or window we look at is classically the RUQ. The probe will be positioned below the right costal margin, with the indicator towards the patient’s head (refer to picture).

Slide 11

First, we try to identify the major abdominal organs in the RUQ, which will help us orient ourselves to obtain a good window to assess for any intra-abdominal bleeding.

Identify: Liver, Kidney (refer to slide for labels).

Identify: Morrison’s pouch (refer to slide for label): between the liver and kidney. It is the place where blood is most likely to collect in this RUQ view. Blood will appear black (anechoic) on US.

Slide 12

Normal RUQ view. There is no fluid between the liver and kidney, so this would be considered “normal” or “negative” in the context of the trauma patient.

Slide 13

Abnormal RUQ view

There is anechoic material in Morrison’s pouch. In the trauma patient, we assume this is blood. Therefore, we consider this to be an “abnormal” or “positive” image in the context of a trauma patient.

Slide 14

RUQ pathology: positive for blood in Morrison’s pouch

Slide 15

What do you think about this one?

RUQ pathology: positive for blood in Morrison’s pouch

Slide 16

Now let’s take a look at the LUQ view. Again, we will hold the probe with the indicator pointed towards the patient’s head. We will start at the costal margin, but for this window, the probe is typically closer to the patient’s head than in the RUQ view (refer to picture).

Slide 17

The LUQ image is very similar to the RUQ image, except now we see the spleen instead of the liver.

Identify: spleen, kidney, diaphragm (refer to labels on slide).

Slide 18

Normal anatomy in the LUQ view – point out spleen, kidney, diaphragm (refer to previous slide for labels).

Slide 19

LUQ abnormal view – there is blood (anechoic material) between the diaphragm and spleen. This is the most likely place for blood to collect in this view. However, we can also see blood sometimes between the spleen and kidney, similar to Morrison’s pouch.

Slide 20

LUQ pathology is present – there is fluid, presumably blood, between the spleen and kidney, which is a pathologic finding. This would be considered an “abnormal” or “positive” image.

Slide 21

How about this one? LUQ pathology is present – Here, we see fluid, presumably blood, between the spleen and the diaphragm. This is another example of a pathologic finding and a “positive” image.

Slide 22

The third window in the FAST exam is the suprapubic view. This window can be obtained in either the transverse view (pictured) or the sagittal view. The probe indicator should be pointed towards the patient’s right in the transverse view (as pictured on slide) and towards the patient’s head when held in the sagittal view.

Slide 23

As in the other views, we first identify the structures we expect to see in this view.

Identify: bladder (filled with urine- anechoic).

Slide 24

This is an example of the transverse view image – normal.

Slide 25

Example of an abnormal suprapubic image- this is how blood would appear. Anechoic material is present, but it is outside of the bladder. Therefore, we can assume that it is blood in the context of the trauma patient. This is an abnormal, “positive” image.

Slide 26

How blood would appear in the sagittal suprapubic view- anechoic material outside of bladder. This is another example of an abnormal, “positive” image.

Slide 27

Finally, the last view we obtain is the subxiphoid view. Place the probe just inferior to the xiphoid process. Indicator to the patient’s right. This view requires some pressure to adequately view the heart. The probe should be almost parallel to the patient.

Slide 28

As in the other views, we must first orient ourselves and identify the major organs. The subxiphoid view allows us to visualize the heart.

Identify: liver, heart (refer to slide labels).

Slide 29

Normal subxiphoid view.

Slide 30

Abnormal subxiphoid view.

Blood appears in the pericardium, between the heart and liver on this view. This is an abnormal finding and would be considered a “positive” image.

Slide 31

What about this patient? Normal or abnormal?

This is an abnormal finding. There is fluid, presumably blood, within the pericardial sac.

Slide 32

Now that we’ve learned the components of the FAST exam, let’s apply it to our trauma patient we met earlier. To refresh your memory, the patient had fallen 30 feet, is hypotensive and tachycardic, with a diffusely tender abdomen. We decided he was too unstable to go to CT and have decided to perform a FAST exam on him. Let’s see what we find.

Slide 33

Patient’s RUQ- (abnormal- blood in Morrison’s pouch).

Slide 34

Patient’s LUQ- normal. No evidence of fluid.

Slide 35

Patient’s suprapubic (transverse view)– normal. No evidence of fluid.

Slide 36

Patient’s subxiphoid view- normal. No evidence of pericardial fluid.

Summarize patient’s FAST exam: “positive”. There is fluid in Morrison’s pouch, which is an abnormal finding. In the context of our unstable trauma patient, a positive FAST examination indicates intra-abdominal bleeding and necessitates a trip to the operating room.

Slide 37

Let’s consider a new patient. This is a 26 year-old male who was dropped off at the door of your ED looking pale and with an obvious stab wound to the center of his chest. On primary survey, the patient’s airway is patent and he has equal breath sounds bilaterally. However, his pulse is weak and thready.

Review vitals- normal or abnormal?

BP 80/40 (abnormal- hypotensive), HR 120 (abnormal- tachycardic), RR 22 (abnormal- slightly tachypneic), oxygen saturation 96% (normal).

Slide 38

In this patient, what are we most concerned about? Where do we think the injury is? – Because this patient was stabbed in the chest, we are most concerned about a potential injury to the heart.

What view of the FAST exam are we going to want to get first? – Subxiphoid view, as we are concerned about an injury to the heart. This will allow us to see if there is a pericardial effusion present.

Slide 39

This is the patient’s subxiphoid view – normal or abnormal? This is abnormal, as there is fluid, presumably blood, within the pericardial sac.

Slide 40

Questions?
