# Supplementary material for: Use of Ultrasound in Introducing Anatomical Pathology to Preclinical Medical Students, in Correlation with Physical Exam Curricula
Source: MedEdPORTAL. 2020 Sep 25;16:10950. doi: 10.15766/mep_2374-8265.10950 (PMC7521063; doi:10.15766/mep_2374-8265.10950)
Supplement: Supplementary file 1 — Session 1 FAST Exam & the Trauma Patient.pptxSession 2 Cardiac and Lung.pptxSession 3 Gallbladder, Kidneys, & AAA.pptxSession 4 Ocular US & Central Access.pptxSession 1 Instructor Script.docxSession 2 Instructor Script.docxSession 3 Instructor Script.docxSession 4 Instructor Script.docxSurvey Questions.docx [file mep_2374-8265.10950-s001.zip › F. Session 2 Instructor Script.docx]

**Session 2 Pathology: Cardiac and Lung**

Instructor PowerPoint script

Slide 1

In this session, we will learn about pathology associated with the cardiac and lung ultrasound (US) exams.

Slide 2

Session objectives

In this session you will learn how to obtain the different sonographic cardiac views and identify basic cardiac anatomy on each one. You will also learn the basic sonographic features of both cardiac tamponade and pneumothorax. Finally, you should be able to correctly diagnose cardiac and lung pathology on ultrasound exams of two hypothetical patient cases presented.

Slide 3

Let’s begin with a case. This is your patient. He is a 74 year-old male who just had a coronary artery bypass graft (CABG) surgery last week. He is brought in by EMS for several hours of worsening shortness of breath (SOB), substernal chest pain, and generalized weakness. So far, his post-operative course has been uncomplicated.

Slide 4

Patient’s history

Past medical history (PMH): coronary artery disease (CAD), hypertension (HTN), hyperlipidemia (HLD), type 2 diabetes (DM2), and myocardial infarction (MI).

Past surgical history (PSH): CABG

Meds: as listed. Aspirin (ASA)

Allergies (ALL): No known drug allergies (NKDA)

Family history (FH): as listed. Cerebral vascular accident- “stroke” (CVA).

Social History (SH): as listed. Alcohol (etoh).

Slide 5

Patient’s examination

Vital signs: review- normal or abnormal? – temperature 37.2 (normal), BP 100/58 (abnormal, borderline hypotensive), HR 114 (abnormal, tachycardic), RR 28 (abnormal, tachypneic), and oxygen saturation 96% (normal).

Exam findings- what is concerning? – distant heart sounds, jugular venous distension (JVD), diminished pulses, tachycardic, and borderline blood pressure.

Slide 6

What do you think might be going on in this patient? Just like we saw in the trauma patient, US can be used as part of our exam to give us quick information about the patient that will help us know what to do to take care of him. In this patient, who just had a bypass, we’d be suspicious for something abnormal going on in his heart. This patient has many abnormalities on his exam that are concerning, and US can be used at the bedside to rapidly assess his heart.

Slide 7

In this patient, it would be very useful to perform a quick cardiac US. For cardiac US exams, we use the phased array probe. Orientation is flipped for cardiac mode, so it will take some practice to get used to the orientation of the images on the screen.

Slide 8

Why cardiac US? On our cardiac US exam, we can quickly assess for cardiac effusion/tamponade, which is the accumulation of blood or fluid within the pericardial sac that inhibits the heart from contracting appropriately. We can also assess for an estimate of cardiac contractility, valvular dysfunction, chamber dilation, hypertrophy, or vegetations on valves. Here, however, we will focus only on identifying effusion/tamponade.

Slide 9

For the cardiac exam, we typically examine the heart in 4 different views: parasternal long, parasternal short, apical, and subxiphoid.

Slide 10

We will start with the parasternal long view.

Slide 11

For the parasternal long view, the phased array probe should be oriented so that the indicator (red line) of the probe (yellow circle) is pointed toward the patient’s right shoulder. We typically start at the 2^nd^-4^th^ intercostal spaces on the patient’s left, but may have to move up or down a rib space to obtain an adequate view. This will vary patient to patient depending on body habitus.

Slide 12

To help orient you for the parasternal long view, we will be viewing the heart as if we are obtaining this cross-section (refer to slide). Again, cardiac mode is a flipped image, so it will appear in opposite orientation on the screen. This is essentially a “long” view of the heart.

Slide 13

Identify structures in the parasternal long view: right ventricle (RV), right atrium (RA)- not seen in this image, left ventricle (LV), and left atrium (LA). The mitral valve (MV) is between the left atrium and left ventricle. The aortic valve (AV) can also be seen here.

Useful tip: the RV is the most anterior portion of the heart, and therefore the closest chamber to the probe. Therefore, it will always be the structure closest to the top of the screen. From there, you can identify the other chambers quickly.

Slide 14

Normal parasternal long view.

Identify structures (refer to previous slide).

Slide 15

Now let’s take a look at the next view in the cardiac exam: the parasternal short view. In this view, we get a cross-section of the LV and RV.

Slide 16

To obtain this view, the indicator (red line) of the phased array probe (yellow circle) should be pointing towards the patient’s left shoulder. From the parasternal long (PSL) view, simply rotate the probe 90 degrees clockwise. Again, we are in the patient’s left 2^nd^-4^th^ intercostal spaces.

Slide 17

For orientation purposes, in this view we are obtaining transverse sections through the ventricles.

Slide 18

Let’s go over the parasternal short anatomy.

Identify: RV, LV, papillary muscle.

This view, because of the shape of the LV, is considered the “fish mouth view.”

Slide 19

Clip of normal parasternal short view on US.

Identify chambers (refer to previous slide).

Slide 20

The next view of the cardiac exam is the apical view. In this view, we get a good look at the 4 different heart chambers and can easily identify dilation and assess contractility as well. We also get a good look at the valves.

Slide 21

To obtain the apical 4 chamber view, we place the indicator again to the left shoulder. We are now at the 4-5^th^ intercostal space, more lateral than the other views.

Slide 22

Visual to help orient. Essentially, we are obtaining a cross-section of the heart that goes through each of the four chambers. We are at the apex of the heart, looking up towards the patient’s head. The yellow lines represent the beams of the US, and the probe would be where the lines almost intersect.

Slide 23

Let’s identify structures in this view: LV, RV, MV, tricuspid valve (TV), RA, LA.

Refer to the slide labels.

Slide 24

Clip of normal apical view- identify chambers (refer to previous slide labels).

Slide 25

Finally, we have the subxiphoid view. Again, we have a view of the 4 chambers, but this time, we use the liver as an acoustic window to enhance our view. In basic terms, having the US beams go through the liver first enhances our visualization of the heart.

Slide 26

As in the subxiphoid view of the FAST exam, we have to apply pressure in order to obtain an adequate view. Probe will almost be parallel to the patient’s torso. Start just inferior to the xiphoid process and aim up towards the patient’s head. Indicator should be towards the patient’s left shoulder.

Slide 27

This picture is to help you visualize this view. It is similar to the apical 4 chamber view, but now we are more parallel to the body. The yellow lines represent the ultrasound beams, and the probe would be where the lines almost intersect.

Slide 28

Here in the subxiphoid view, we can apply our trick of identifying the RV first, as it will be the closest to the top of the probe, and thus to the top of the screen. Identify: liver, RV, RA, LV, LA.

Refer to the labels on the slide.

Slide 29

Clip of normal subxiphoid view.

Identify structures (refer to previous slide labels).

Slide 30

Now that we have gone through the basics of the sonographic cardiac exam, let’s go back to the case and apply it to our patient. What are we seeing in the clip? We see anechoic material in the pericardial sac, which is abnormal. This clip also demonstrates tamponade physiology, which we will discuss in subsequent slides.

Slide 31

What is cardiac tamponade? Blood (or fluid) surrounds the heart in the pericardial sac, preventing the heart from filling during diastole, limiting cardiac output. This limited cardiac output leads to hypotension, which our patient had.

Slide 32

There are certain signs of tamponade that we will see on US. First, as we saw, there will be blood (anechoic material) surrounding the heart. Next, we will see the RV collapse during diastole, which prevents filling. As it progresses, we will then see RA collapse, inferior vena cava (IVC) dilation with loss of respiratory variations, and ventricular inter-dependence.

Slide 33

Flowchart of how the change in pressure gradients leads to tamponade physiology and what we will see on US.

Slide 34

Clip of tamponade physiology. Point out the pericardial effusion and the RV collapse.

Slide 35

Now let’s move on to another case. Your patient is a young male who was in a motor vehicle accident (MVA). He was the restrained driver and was hit by another vehicle in the driver’s side of the car while going approximately 40mph. He did not lose consciousness. The airbags did go off. The patient was able to get out of the car himself after the accident, but he is complaining of left side pain.

Slide 36

Past medical history (PMH): none

Past surgical history (PSH): broken leg

He does not take medications.

Allergies (ALL): penicillin (PCN)

Family History (FH): hypertension (HTN) in father

Social history (SH): denies smoking, drinks alcohol socially, denies illicit drug use

Slide 37

This patient has a patent airway, slightly diminished breath sounds on the left, and good radial pulses. (primary survey)

Vitals: temp 36.8 (normal), BP 128/90 (normal), HR 84 (normal), RR 20 (upper limit of normal), oxygen saturation 98% (normal).

Exam: point out the diminished breath sounds on the left along with the tenderness to palpation along the left lateral inferior ribs.

Slide 38

So you get a chest xray (CXR), which confirms some rib fractures. Then about 45 minutes later, the nurse comes to find you because the patient is now having difficulty breathing and looks more uncomfortable. Respiratory rate- normal or abnormal? (30 is abnormally high). Oxygen saturation- normal or abnormal? (92% is abnormal, low). Lung sounds on the left are now more diminished, and you’re not sure if you can really hear anything at all. He is also hyperresonant to percussion. You want to order another CXR, but they are backed up and you are told it will be at least 30 minutes before it can be done….

Slide 39

What do you think is wrong with this patient? This patient likely has a pneumothorax.

Slide 40

This patient has a pneumothorax, which is when air collects in the pleural space and collapses the lung. This leads to decreased breath sounds and hyperresonance to percussion on exam. If the pneumothorax gets large enough, it can lead to decreased cardiac output, with similar physiology to cardiac tamponade. This is called tension pneumothorax.

Slide 41

Pneumothorax (PTX) will appear on US as the absence of lung sliding on B mode. On M mode, it will appear as the “stratosphere sign.” We will not discuss the different ultrasound modes here further.

US is actually 92-100% sensitive for identifying pneumothorax, which is better than XR is in the supine patient!

Slide 42

For the lung exam, we will be using the linear probe (represented by the yellow line). It will be positioned in the sagittal plane, perpendicular to the 2^nd^ rib spaces in the midclavicular line. We will scan 2-3 interspaces on each side. The indicator (red dot) will be oriented towards the patient’s head.

Slide 43

First, we must orient ourselves by identifying structures: ribs, pleural line, intercostal muscles (refer to the labels on the slide). Ribs appear hypoechoic, while the pleural line appears hyperechoic (brighter).

Slide 44

What is lung sliding? Sliding indicates lack of air between the visceral and parietal pleura. This is the NORMAL finding. Lack of sliding indicates that there is air in between the visceral and parietal pleura, which indicates a pneumothorax. A lack of sliding on US is ABNORMAL. The change seen on US is because US waves are unable to penetrate through air, and the visceral layer thus cannot be visualized.

Slide 45

Clip of normal lung sliding. Identify structures (refer to previous slides).

Slide 46

Clip of no lung sliding= abnormal finding, suggests pneumothorax.

Slide 47

Pneumothorax can also be identified in another mode of US: “M mode.” This stands for motion. The movement associated with lung sliding in a normal patient creates an unorganized, “static” appearance on M mode. This is referred to as the “Seashore sign,” and is a normal finding. When there is air present, this prevents movement, and therefore the appearance on US is more organized, resulting in horizontal lines. This is referred to as the “stratosphere sign,” and is a pathologic finding, concerning for pneumothorax. It is also referred to as the “barcode sign.” The ultrasound still image depicted on the slide is an example of the seashore sign, which is the normal finding.

Slide 48

Clip of normal findings on M mode. Seashore sign is present.

Slide 49

Now, back to the case. So you examine this patient with US, and this is what you see. Normal or abnormal? There is absence of lung sliding, so this patient likely has a pneumothorax.

Slide 50

Questions?
