# Supplementary material for: Use of Ultrasound in Introducing Anatomical Pathology to Preclinical Medical Students, in Correlation with Physical Exam Curricula
Source: MedEdPORTAL. 2020 Sep 25;16:10950. doi: 10.15766/mep_2374-8265.10950 (PMC7521063; doi:10.15766/mep_2374-8265.10950)
Supplement: Supplementary file 1 — Session 1 FAST Exam & the Trauma Patient.pptxSession 2 Cardiac and Lung.pptxSession 3 Gallbladder, Kidneys, & AAA.pptxSession 4 Ocular US & Central Access.pptxSession 1 Instructor Script.docxSession 2 Instructor Script.docxSession 3 Instructor Script.docxSession 4 Instructor Script.docxSurvey Questions.docx [file mep_2374-8265.10950-s001.zip › G. Session 3 Instructor Script.docx]

**Session 3 Pathology: Abdomen**

Instructor PowerPoint Script

Slide 1

In this session, we will go over some intra-abdominal pathology.

Slide 2

Session objectives

In this session, you will learn how to obtain images of the gallbladder, abdominal aorta, and kidneys and be able to identify sonographic signs of cholecystitis, AAA, and hydronephrosis, respectively. Finally, we will see three patient cases and you should be able to diagnose their pathology based on their ultrasound findings.

Slide 3

We will start with our first case. Your patient is a 41 year-old obese female with 3 days of worsening abdominal pain, mostly in the right upper quadrant (RUQ). Her pain is worsened by food intake. She also reports fever, nausea, and vomiting since yesterday.

Vitals: temp 38.4 (abnormal- high), BP 145/86 (abnormal- high), HR 102 (abnormal- high), RR 16 (normal), oxygen saturation 98% room air (normal).

Slide 4

Patient’s exam findings: moderately tender to palpation in the RUQ, but without any rebound or guarding. What do you think could be wrong with this patient? What would you consider doing next?

This is another scenario in which ultrasound (US) can be incredibly useful in diagnosing a patient at the bedside (or in the radiology suite). Based on this patient’s presentation, we should be concerned about several different differential diagnoses, but one of these is gallbladder pathology.

Slide 5

For imaging the gallbladder, we will use the curvilinear probe. There are different ways to scan for the gallbladder, but typically we will start by placing the patient in the left lateral decubitus position. We can often start by holding the probe in the sagittal orientation, with the indicator towards the patient’s head, and then scan along the border of the ribs (along the path represented by the black line) and try to identify a small sac-like structure adjacent to the liver, which is the gallbladder. Another tip is to have the patient take a deep breath and hold it. This will cause the diaphragm to push downward, thus sliding the liver and gallbladder into a better view for you. Just remember to have the patient breathe again!

Slide 6

This is an example of what a normal gallbladder will look like on US. It appears as an anechoic sac-like structure near the liver. We can view it in both the longitudinal and transverse planes. Be careful, because in the transverse view, it can almost appear as a vascular structure!

Slide 7

Clip of a normal gallbladder on US.

Slide 8

Cholelithiasis can also be a normal gallbladder finding. This is just the medical term for gallstones. Not every patient with gallstones will be symptomatic. Many people in this room probably have gallstones. The known risk factors associated with gallstones are female gender, middle age (40s), obesity, and family history.

Slide 9

Gallstones will often appear as hyperechoic (bright) round structures within the gallbladder. They will often produce shadowing, which is referred to as acoustic shadowing. Often, as the patient breathes or makes subtle movements, you can also see the stones actually rolling within the gallbladder, aptly named the “rolling stone sign.” In the US image depicted, we can see a gallstone at the inferior portion of the gallbladder.

Slide 10

Clip of cholelithiasis, stones within the gallbladder.

Slide 11

Often, we can also see something within the gallbladder named biliary sludge. This appears as homogenous echoes, with often layering on the posterior or inferior wall of the gallbladder. There will NOT be shadowing with this. As with the rolling stone sign, sludge is also position dependent. Essentially, this is material that has not yet formed distinct stones. The US image depicted shows biliary sludge in the gallbladder, layered to just about halfway.

Slide 12

Clip of biliary sludge.

Slide 13

Cholecystitis is a good example of abnormal gallbladder pathology. This term refers to inflammation or infection of the gallbladder. This often occurs when a stone becomes lodged within the cystic duct, impeding drainage. There are several different signs of cholecystitis on US. First, the anterior wall (closest to the probe and therefore on the top of the screen) will be >3mm. There will be pericholecystic fluid, or fluid surrounding the gallbladder. Often, you will also see the stones themselves (but stones themselves do not always mean infection!). Finally, the last sign is something called a sonographic Murphy’s sign, which is the most sensitive sign. This actually refers to a finding on exam. As you apply pressure with the probe in the RUQ and ask the patient to take a deep breath, the pain associated with the pressure of the probe will cause the patient to pause during inspiration. This finding is suggestive of cholecystitis.

Slide 14

So, let’s go back to the case! Again, our patient is a middle-aged female complaining of RUQ abdominal pain. What are we concerned about in this patient? (cholecystitis). This is what we see on ultrasound. (The clip shows the gallbladder with a thickened anterior wall and pericholecystic fluid. We cannot see any clear gallstones, however).

Slide 15

Case 2. We have a new patient. We are seeing a 28 year-old male with 2 days of worsening left flank pain, occasionally radiating down to his left groin. He is mildly nauseated. He has had similar pain before, but says that the pain went away after a few days. He denies fevers, burning with urination, and hematuria.

Slide 16

Vital signs: normal or abnormal? Temp 37.6 (normal), BP 130/76 (normal), HR 98 (upper limit of normal), RR 16 (normal), oxygen saturation 98% (normal).

Exam: appears very uncomfortable, cannot find a comfortable position. Tender along the left costovertebral angle (CVA), and also has mild tenderness in the LUQ and LLQ.

What do you think is going on with this patient? – This is the classic presentation of nephrolithiasis (kidney stones).

Slide 17

Let’s review basic anatomy of the kidneys. Identify: renal vasculature, ureter, renal pelvis, medulla, cortex, calyces (refer to labels on slide image).

Slide 18

US still image of normal kidney anatomy. Identify: cortex, medulla.

Slide 19

Here we have some abnormal anatomy. Hydronephrosis refers to the dilated ureter and urinary system outflow, often from an obstruction. A kidney stone can sometimes cause an obstruction like this. There are varying degrees of dilation and hydronephrosis, which depend on how bad the obstruction is and how long it has been there.

Slide 20

First, let’s review mild hydronephrosis. This is the first phase and least severe. It refers to the dilation of the renal pelvis with minimal calyceal dilation. There is no parenchymal atrophy. As depicted in the image.

Slide 21

Clip of mild renal hydronephrosis. Note how the renal pelvis is dilated with minimal calyceal involvement.

Slide 22

As it progresses, the next step is moderate hydronephrosis. In moderate hydro, we have moderate dilation of the pelvis and calyces, mild cortical thickening, blunting of the fornices, and flattening of the papillae. As depicted in the US image.

Slide 23

Clip of Moderate Hydronephrosis. Note the differences between this and mild hydro.

Slide 24

Finally, we have severe hydronephrosis. Here, there are obvious changes on US. We have gross dilation of the pelvis and calyces, loss of the borders between the pelvis and calyces, and renal atrophy with cortical thinning. As depicted in the US image.

Slide 25

US clip of severe hydro. Note the cortical thinning and the loss of borders between the pelvis and calyces.

Slide 26

Back to the case. So, we have a young male with normal vitals and left flank pain. What do we think is going on? (kidney stones). Let’s looks at his US (mild hydro). So, this patient can likely go home with pain meds and follow up on an outpatient basis. Of note, CT scan is also often used to diagnose kidney stones and is considered superior to US. However, US can play a role in young patients, pregnant patients, and in patients who have had previous episodes of stones in the past, in order to limit their radiation exposure.

Slide 27

Our next case is a 73 year-old male presenting after a syncopal episode. Admits to similar episodes over the past several months, but has never lost consciousness before. He states that he suddenly felt weak, lightheaded, nauseated, and mildly short of breath (SOB) before he lost consciousness.

Slide 28

Vitals: normal or abnormal? Temp 37.2 (normal), BP 96/58 (abnormal- hypotensive), HR 104 (abnormal- tachycardic), RR 18 (normal), oxygen saturation 97% (normal).

This patient is hypotensive and tachycardic. Exam: he looks pale, diaphoretic, and his abdomen is mildly tender to palpation.

Slide 29

What do we think is going on in this patient? Obviously, this is a lecture on US, so we know we need to image something with US. But, what are we going to image? (abdomen for AAA). While CT can often provide better imaging of an abdominal aortic aneurysm (AAA) if present, US can be performed much faster in this patient with abnormal vitals and can rapidly provide us with a diagnosis that can guide our treatment.

Slide 30

Abdominal Aortic Aneurysm (AAA) is a localized dilation of the abdominal aorta. The majority are asymptomatic, but as it grows larger, the risk for rupture increases. The most common location is infrarenal (inferior to the takeoff of the renal arteries), and it is more common in males.

Slide 31

On US, this is what a AAA will look like. As we identify the abdominal aorta, a diameter up to 3cm is considered normal. Anything >3cm would be considered dilated. At the bifurcation of the iliac arteries, anything >1.5cm is considered dilated and abnormal. If it grows large enough, you may feel a pulsatile abdominal mass on exam.

Slide 32

To accurately measure the diameter of the abdominal aorta, you must measure from the outer wall to the outer wall (yellow line). A diameter > 5-5.5cm has a higher risk of rupture.

Slide 33

To obtain images of the abdominal aorta, we will use the curvilinear probe, in the transverse plane, with the indicator pointed towards the patient’s right. This is an example of a normal abdominal aorta (US image). Identify: aorta, IVC, spine (refer to slide labels). Once we locate the aorta, we must scan through the entire aorta, from just below the xiphoid process to the bifurcation of the iliac arteries.

Slide 34

Example of normal anatomy: identify aorta, IVC, spine (refer to labels on previous slide).

Slide 35

Basic anatomy of the abdominal aorta. Go over the branches: celiac trunk, superior mesenteric artery, right and left renal arteries, inferior mesenteric artery (refer to image labels). Again, most AAAs are infrarenal.

Slide 36

The first branch, the celiac trunk, has this classic appearance on US. It is referred to as the seagull sign. This is where you should begin scanning the abdominal aorta for dilation.

Slide 37

As we follow the aorta down the abdomen, we will eventually come to the bifurcation of the iliac arteries. Again, here, the diameter should be no more than 1.5cm. The US clip depicts the aorta bifurcating into the two iliac arteries.

Slide 38

Now, let’s go back to our patient. We have an elderly gentleman with syncope who looks pale and diaphoretic on exam, with hypotension and tachycardia as well as some abdominal pain. As we scan his belly with US, you see this. What does he have and why is this bad? (AAA and it has likely ruptured or in the process of rupturing🡪 hemorrhagic shock). This patient needs a vascular surgeon immediately, as well as several other interventions.

Slide 39

Questions?
