# Supplementary material for: Use of Ultrasound in Introducing Anatomical Pathology to Preclinical Medical Students, in Correlation with Physical Exam Curricula
Source: MedEdPORTAL. 2020 Sep 25;16:10950. doi: 10.15766/mep_2374-8265.10950 (PMC7521063; doi:10.15766/mep_2374-8265.10950)
Supplement: Supplementary file 1 — Session 1 FAST Exam & the Trauma Patient.pptxSession 2 Cardiac and Lung.pptxSession 3 Gallbladder, Kidneys, & AAA.pptxSession 4 Ocular US & Central Access.pptxSession 1 Instructor Script.docxSession 2 Instructor Script.docxSession 3 Instructor Script.docxSession 4 Instructor Script.docxSurvey Questions.docx [file mep_2374-8265.10950-s001.zip › H. Session 4 Instructor Script.docx]

**Session 4 US Pathology: Ocular and Access**

Instructor PowerPoint Script

Slide 1

In this last session, we will review ocular pathology on ultrasound (US) and the use of US in obtaining intravenous (IV) access.

Slide 2

Review session objectives. In this session, you will be able to identify basic ocular anatomy on ultrasound and be able to diagnose retinal detachment and vitreous hemorrhage. You will also learn how to differentiate between arterial and venous structures on ultrasound and recognize the use of ultrasound in obtaining both peripheral and central venous access.

Slide 3

First, our patient is a 75 year-old male presenting with acute painless vision loss. Describes it like a “curtain coming down” in his right eye. He had been seeing flashes of lights intermittently in that eye over the last 2-3 days. No other symptoms, and his left eye is unaffected.

Slide 4

Here, we will see that US can even be used to examine the eyes! More specifically, it is very useful at viewing potential posterior eye pathology. The patient in our case will likely still require a dilated eye examination by an ophthalmologist, but we can find out more about what is going on with the patient first by using US. For ocular US, we use the linear probe. You can place it directly over the eye, but it is nicer for your patient to place a tegaderm over the right eye first to prevent the US gel from getting in the eye.

Slide 5

A review of the normal eye anatomy. Identify: anterior chamber structures and posterior chamber structures (refer to image labels).

Slide 6

To visualize the cross-section view on US, here are some examples. The most anterior portion of the eye is closest to the probe and thus on the top of the screen. The optic nerve, which is the most posterior portion of the eye, is on the bottom of the screen. It is one of the more intuitive US examinations. Point out structures on the US image (refer to labels). The yellow lines on the cartoon image represent the beams of the ultrasound, and the probe would be positioned at the top of the lines.

Slide 7

US clip of normal eye anatomy. Identify: anterior chamber, posterior chamber, optic nerve, cornea, lens (refer to previous slide).

Slide 8

Retinal detachment is when fluid enters the potential space beneath the retina of the eye, causing displacement/detachment of the retina. Depicted as the red in the image.

Slide 9

This is how a retinal detachment would appear on US. There will be a hyperechoic membrane extending from the optic nerve. This is our patient’s ultrasound, and therefore, he appears to have a retinal detachment. His story of painless vision loss described as a “curtain coming down” is the classic story for retinal detachment.

Slide 10

The other example of posterior chamber eye pathology is vitreous hemorrhage. This actually refers to bleeding within the vitreous, or posterior chamber of the eye. Depicted red in the image.

Slide 11

Vitreous hemorrhage appears as echogenic material in the posterior chamber of the eye, as seen here (left image). Sometimes, if there is an associated vitreous detachment (right image), it can appear similarly to a retinal detachment. However, the hyperechoic membrane will often cross the optic nerve, which would never happen with a retinal detachment.

Slide 12

Finally, we will cover retrobulbar hematoma. This is the term for a hematoma or bleeding behind, or posterior to, the globe itself. On physical exam of the patient, the eye will appear proptotic, or protruding. The hematoma is depicted as the red in this image.

Slide 13

On US, a retrobulbar hematoma will appear as hyperechoic material posterior to the globe, as seen in this US image.

Slide 14

Finally, let’s go over some optic nerve pathology. The optic nerve is important for many reasons, but just one of them is that it correlates to ICP (intracranial pressure). On fundoscopy, this is what a normal optic nerve looks like. With an optic nerve in a patient with increased ICP (papilledema), the cup:disk borders would be distorted. Here in this normal image, we see that the optic nerve (bright spot on the left of the image where the vessels are branching from) has clearly defined borders.

Slide 15

One way of detecting increased intracranial pressure is by measuring the optic nerve sheath diameter (ONSD) on US. To obtain this measurement, we first measure 3mm posterior to the posterior wall of the globe. From this point, we measure the diameter of the optic nerve (the most hypoechoic appearing material). We take an average of 2 measurements. Normal ONSD is up to 5mm.

Slide 16

Example of an increased ONSD on US. The measurement here is almost 7mm, which is abnormally high. This finding would suggest elevated ICP.

Slide 17

Another major use of US is its use in obtaining central line access. Review basic anatomy. Identify: right atrium, subclavian vein, internal jugular vein (refer to labels on image). In placing a central line, we aim to put a catheter in either the internal jugular vein or the subclavian vein. We can also place one in the femoral vein, but we will not discuss that here.

Slide 18

We would typically be standing at the head of the bed to place an internal jugular (IJ) central line. Using ultrasound, we would typically see an image similar to this one. Identify on US image: IJ, Carotid (refer to labels). In order to distinguish between arteries and veins on US, there are several things we look at. First, arteries typically have thicker walls and are pulsatile. However, when a patient is in extremis and hypotensive, we may not always be able to see pulsations. Venous structures are collapsible under pressure, which is the main finding which allows us to distinguish them from arterial structures. A venous structure (without a clot in it) will be able to be fully compressed when pressure is applied by the US probe, whereas an arterial structure will not be.

Slides 19 / 20

We can also use US to insert peripheral IVs. This is a very useful skill to have, especially in patients who need bloodwork and access, but don’t necessarily need a central line placed. Again, vascular structures will appear hypoechoic on US, and the needle tip will appear hyperechoic. We can hold the US in either the transverse orientation (most common), as seen on this slide, or in the longitudinal plane (next slide). Again, we identify venous structures by their ability to be completely compressed when pressure is applied. The top picture is without pressure and clearly shows a vascular structure. The bottom picture is with pressure applied, and we see that the vascular structure has been fully compressed, indicating that it is venous. In the longitudinal view, it is harder to acquire the exact plane of both the needle and the vessel, but we can directly watch as the needle penetrates the vessel. On the other hand, in the transverse view, it is much easier to view both the needle track and vessel, but it is harder to find the cross section where the needle exactly enters the vessel. Instead, we must always try to keep track of where the needle tip is and slowly inch the probe along the track of the vessel, and then advance the catheter. We continue to slowly alternate between advancing the probe, and then slowly advancing the catheter until the needle tip again comes into view within the vessel. Once we do this 2-3 times, we then can advance the catheter all the way and remove the needle. As you can imagine, it takes a lot of practice. However, inserting peripheral IVs with ultrasound is an invaluable skill to acquire!

Slide 21

Questions?
