# Supplementary material for: Use of Ultrasound in Introducing Anatomical Pathology to Preclinical Medical Students, in Correlation with Physical Exam Curricula
Source: MedEdPORTAL. 2020 Sep 25;16:10950. doi: 10.15766/mep_2374-8265.10950 (PMC7521063; doi:10.15766/mep_2374-8265.10950)
Supplement: Supplementary file 1 — Session 1 FAST Exam & the Trauma Patient.pptxSession 2 Cardiac and Lung.pptxSession 3 Gallbladder, Kidneys, & AAA.pptxSession 4 Ocular US & Central Access.pptxSession 1 Instructor Script.docxSession 2 Instructor Script.docxSession 3 Instructor Script.docxSession 4 Instructor Script.docxSurvey Questions.docx [file mep_2374-8265.10950-s001.zip › I. Survey Questions.docx]

Survey Questions

1.“I found the 4 Pathology Sessions useful for my learning.”

Strongly Agree

Agree

Neutral

Disagree

Strongly Disagree

2.“The information presented in these sessions was appropriate for my level of training.”

Strongly Agree

Agree

Neutral

Disagree

Strongly Disagree

3.“Learning pathological correlates to the physical exam helped me understand normal anatomy and physiology.”

Strongly Agree

Agree

Neutral

Disagree

Strongly Disagree

4.“The Pathology sessions helped me better understand the basic physics of ultrasound (gain, depth of image, echogenicity of tissues, shadowing effects, etc)”

Strongly Agree

Agree

Neutral

Disagree

Strongly Disagree

5.“The pathology sessions helped enhance my ultrasound training.”

Strongly Agree

Agree

Neutral

Disagree

Strongly Disagree

6.“The Pathology sessions helped increase my confidence on identifying “normal” versus “abnormal” anatomy on ultrasound.”

Strongly Agree

Agree

Neutral

Disagree

Strongly Disagree

7.“Please provide any additional feedback on any of the sessions (1. FAST, 2. Cardiac and lung, 3. Gallbladder/kidney/AAA, 4. Eye and central access).”
