# Supplementary material for: Use of Ultrasound in Introducing Anatomical Pathology to Preclinical Medical Students, in Correlation with Physical Exam Curricula
Source: MedEdPORTAL. 2020 Sep 25;16:10950. doi: 10.15766/mep_2374-8265.10950 (PMC7521063; doi:10.15766/mep_2374-8265.10950)
Supplement: Supplementary file 1 — Session 1 FAST Exam & the Trauma Patient.pptxSession 2 Cardiac and Lung.pptxSession 3 Gallbladder, Kidneys, & AAA.pptxSession 4 Ocular US & Central Access.pptxSession 1 Instructor Script.docxSession 2 Instructor Script.docxSession 3 Instructor Script.docxSession 4 Instructor Script.docxSurvey Questions.docx [file mep_2374-8265.10950-s001.zip › _Educational_Summary_Report_10950.pdf]

MedEdPORTAL® The AAMC Journal of Teaching and Learning Resources

Original Publication Open Access

Use of Ultrasound in Introducing Anatomical Pathology to Preclinical Medical Students, in Correlation with Physical Exam Curricula

Lauren Trembley, MD*, Marek Radomski, DO

*Corresponding Author: westovel@gmail.com

Abstract

Introduction: Point-of-care ultrasound has become an important diagnostic tool in many clinical settings. Many medical schools have responded by incorporating instruction on ultrasound into the curriculum for medical students in their clinical years. The curriculum presented here will assist preclinical medical students in distinguishing between normal and pathologic sonographic anatomical findings. **Methods:** The course consisted of four, approximately 30-minute case-based PowerPoint slide shows introducing pathologic anatomical findings on ultrasound through clinical case-based scenarios. Twelve preclinical (first- and second-year) medical students attended each weekly session. An emergency medicine resident created and presented the course content as an adjunct to an established course instructing students on how ultrasound correlates to the normal physical exam. Upon completion of the course, the instructors emailed the students an online, seven-question survey. **Results:** Survey results showed positive feedback, with 71% of respondents answering *strongly agree* to the survey question that addressed the primary educational objective, which was to enable students to distinguish between normal and pathologic anatomical findings on ultrasound. Open response feedback highlighted that the course complemented the existing course well and suggested that the course be continued. **Discussion:** A curriculum which presents pathologic anatomical findings on ultrasound can be a useful tool in the education of preclinical medical students. Such a course enabled learners to more easily distinguish between normal and pathologic exam findings and introduced them to the many clinical uses of ultrasound at an earlier stage in their training, allowing them to develop this important skillset.

Keywords
Ultrasound, Ultrasonography, Pathology, Preclinical Students, Anatomy

Educational Objectives

By the end of this course, preclinical medical students will be able to:

1. Distinguish between normal and pathologic anatomical ultrasound findings through clinically based case examples.
2. Identify several predetermined anatomical pathologies of the heart, lung, gallbladder, kidneys, abdominal aorta, eye, and the trauma patient, through clinically based case examples.
3. Demonstrate how to obtain adequate sonographic images of the above anatomical regions through practice with partners.

Citation:
Trembley L, Radomski M. Use of ultrasound in introducing anatomical pathology to preclinical medical students, in correlation with physical exam curricula. *MedEdPORTAL*. 2020;16:10950.
https://doi.org/10.15766/mep_2374-8265.10950

Introduction

Point-of-care ultrasound (POCUS) has become a useful tool in identifying various anatomical pathologies. Its use has become especially valuable within emergency medicine, as its ability to be used at the bedside can provide quick diagnostic information and guide further treatment and management decisions, which is of particular value in unstable patients.[1] POCUS can be a useful diagnostic tool in identifying a variety of clinical applications, including goal-directed echocardiography,[2] use in patients with cardiopulmonary arrest,[3] and rapid detection of pneumothorax.[4] In addition, POCUS can be more specific in diagnosing certain conditions as compared to traditional imaging modalities (for instance, chest X-ray for pneumothorax), especially when looking to make diagnoses at the bedside in a rapid and accurate manner. Through the focused assessment with sonography in trauma (FAST) exam, it has also become a key diagnostic study in unstable trauma patients.[5] Further, ultrasound has numerous procedural applications, including central and peripheral venous catheterization.[5-6]

Copyright © 2020 Trembley and Radomski. This is an open-access publication distributed under the terms of the Creative Commons Attribution license.

Many medical schools have recognized the growing importance of ultrasound as a diagnostic tool and have incorporated introductory sessions on its use into the curriculum, often for students in their clinical clerkships or as electives.[7-8] Several courses incorporated ultrasound into learning the physical exam, mostly emphasizing normal anatomical findings.[9-10] Because students typically gain practice with ultrasound either by scanning their classmates or standardized patients, there exists a knowledge gap in recognizing true anatomical pathology and abnormal exam findings at this stage in training.

This curriculum used ultrasound as a modality to introduce anatomical pathology to preclinical medical students as a means to fill this gap and aid students in distinguishing between normal and pathologic anatomical findings. The introduction to ultrasound at this early stage in training allowed students to become familiar with its use and develop a basic skillset. By focusing on pathology, it took the existing course on ultrasound correlates to the physical exam in the literature one step further,[9-10] providing students with a framework and the context to actually distinguish between normal and abnormal anatomy. This curriculum presented pathology from high-yield clinical scenarios in a case-based format so that students also learned the importance of ultrasound in many diagnostic evaluations. The cases incorporated numerous ultrasound clips demonstrating the pathologies in the appropriate clinical contexts, allowing for direct comparison with normal anatomical findings. To our knowledge, no previous curriculum in the current literature targeting preclinical medical students has focused on pathologic correlates to the physical exam.

Methods

We designed this curriculum for preclinical (first- and second-year) medical students, who had no prior exposure to, or just basic familiarity with, ultrasound. We implemented the course as an adjunct to an already established elective course for preclinical medical students at the University of Pittsburgh Medical School entitled, "Ultrasound Basics: Correlation with the Physical Exam." Faculty that were trained in POCUS or have completed an emergency ultrasound fellowship led the didactic portion of the course. Facilitators that assisted students with the scanning portions were clinical instructors from various specialties, including emergency medicine, OB/GYN, and critical care medicine. The course consisted of four, approximately 90-minute weekly sessions. Each session consisted of a brief introductory didactic portion, with the time remaining used for scanning practice. The didactic portion introduced students to the basics of ultrasound use, presenting introductory information on the different probes and their use for different exams, as well as the basic settings (gain and depth). The first session was an introductory class designed to focus on the basics of ultrasound image acquisition, the second session covered cardiac and aortic ultrasound, the third session reviewed the genitourinary system, and the final session focused on head and neck ultrasound. During the hands-on scanning portion, students practiced obtaining images to enhance their learning of the anatomical region discussed that week with supervision from a facilitator who ensured adequate image acquisition. In short, the course instructed students on the basic use of ultrasound and used ultrasound as a tool to aid in learning normal anatomy.

The new curriculum presented here focused on introducing clinical pathology and was implemented into the existing course as an adjunct, given after each scanning portion. This new adjunct course consisted of four, approximately 30-minute lectures designed in a case-based format (Appendices A-D). The pathology introduced correlated to the anatomical region covered in the first portion of the course. The four topics of the sessions were: FAST exam and the evaluation of the trauma patient; cardiac and lung; abdomen; and ocular and access. The specific pathologies introduced within these topics included: intraperitoneal blood, cardiac tamponade, pneumothorax, cholecystitis, hydronephrosis, abdominal aortic aneurysm, and retinal and vitreous detachment. Therefore, after we implemented the pathology course, for each weekly session the students first received a brief didactic portion on the basics of ultrasound and on the anatomical region that was the topic for that session (approximately 15-20 minutes). The students then practiced scanning that region on their classmates in small groups led by a facilitator (70-75 minutes). Students self-assembled into small groups of three to four students, based on gender in order to assure student comfort. Facilitators were also of the same gender. Finally, students received the new course material exposing them to the clinical pathology of that region, again in the large-group setting (30 minutes). Because students scanned each other during scanning practice, they presumably observed a myriad of only normal findings. Therefore, each session concluded with the pathology component, enabling timely exposure to pathologic findings which students would not have seen during the practice sessions scanning their colleagues. An emergency medicine resident created and presented the pathology sessions, and an emergency ultrasound fellowship-trained physician reviewed the sessions for content accuracy prior to their incorporation into the course.

Copyright © 2020 Trembley and Radomski. This is an open-access publication distributed under the terms of the Creative Commons Attribution license.

Materials needed for the pathology sessions included a computer with a hook-up to projection device, the four presentation slides, and the four scripts to accompany each of the presentations (Appendices E-H). If chosen to be implemented after an established curriculum or ultrasound scanning session as done at our institution, additional materials would include enough ultrasound machines to facilitate groups of three to four students.

After the implementation of the four pathology sessions, we emailed students an online survey (using Survey Monkey). The survey questions assessed how well the students felt the pathology course had enabled them to distinguish between normal and abnormal findings (educational objective), and how it impacted their overall ultrasound skills. There were six questions, with responses measured on a Likert scale. In addition, students could leave any further feedback in a seventh, open-ended response question. Because the course was an elective for students, survey questions were basic, primarily designed to elicit student feedback to determine the merit in implementing this adjunct course and whether to continue it in future sessions. Therefore, survey questions did not undergo prior validity testing. Survey questions can be found in Appendix I. To augment the number of responses, the students received a second email a week later as a reminder to complete the survey.

Results

Twelve preclinical medical students registered for and completed the elective course with the four pathology sessions included in January-February of 2018. Seven of the 12 students completed the online survey at the conclusion of the course. Survey feedback was generally positive. Seventy-one percent answered *strongly agree* to the prompt, “Learning pathological correlates to the physical exam helped me understand normal anatomy and physiology,” which targeted the first educational objective. No one responded *disagree* or *strongly disagree*. Question 5 (“The pathology sessions helped enhance my ultrasound training”), which targeted the third educational objective, also had generally positive responses, with again no one responding *disagree* or *strongly disagree*. The open-ended responses highlighted that the pathology course complemented the existing course well and suggested that the new course be continued. All of the open-ended responses received included:

- “I thought that all the sessions and especially the case presentations were a great complement to the scanning sessions themselves!”
- “All were great to have, just my lack of experience in general with ultrasound prevents me from saying these sessions actually improved my skills. I definitely appreciated them, would suggest you continue them if given the opportunity.“
- “[The instructor] did a great job with the pathological correlates in her lectures and was also a very helpful and approachable facilitator. The only comment I would make is that for the next mini-elective I would consider having the pathology sessions right after [the main ultrasound lecture]. It was more cohesive that way and allowed us to just stay in the rooms and practice for longer, without having to reshuffle back to the lecture room.”

Discussion

To enable preclinical medical students to more easily distinguish between normal and pathologic anatomical exam findings, we created a curriculum introducing students to pathologic findings on ultrasound, which was implemented as an adjunct to an established course focused on ultrasound correlates to the physical exam. The four sessions were designed in a case-based format, highlighting high-yield clinical scenarios in which ultrasound can be a useful diagnostic tool.

The strengths of this novel course are many. First, the introduction of clinical pathology on ultrasound to preclinical medical students aided their understanding and identification of normal and pathologic anatomical findings. When ultrasound is introduced into the curriculum in current practice as described in the literature, students typically gain scanning experience by scanning either their fellow classmates or standardized patients.[7,8] As a result, they became adept at identifying normal anatomical findings. Thus, the pathology sessions allowed the students to immediately compare normal findings to abnormal ones, thus allowing for more immediate, comprehensive learning. The pathologies chosen were based on high-yield, can’t-miss diagnoses that are able to be identified with POCUS. Next, the case-based format of the sessions allowed students to see the various uses for ultrasound in the clinical setting, as well as its growing importance as a diagnostic tool. The use of a case-based format also allowed the students to see where POCUS fits into the clinical decision-making paradigm, as compared to other imaging or diagnostic studies. Finally, the course introduced students to ultrasound at an earlier stage in their training, thus allowing their skills and familiarity with ultrasound to develop during their preclinical years, so that they will already be reasonably adept during their clinical clerkships.

One limitation of the course was that some of the material presented in the pathology sessions, because of the clinical

Copyright © 2020 Trembley and Radomski. This is an open-access publication distributed under the terms of the Creative Commons Attribution license.

case-based format, would be considered advanced for the level of training of a preclinical medical student. For example, one of the cases in the session focused on cardiac and lung pathology presented an elderly male patient with chest pain after a recent coronary artery bypass graft surgery. He was ill-appearing, hypotensive, and tachycardic. The included ultrasound clips showed a large pericardial effusion and demonstrated tamponade physiology. Students were able to correctly identify the abnormal vital signs, and then instructors gave a brief summary on the basics of tamponade physiology. For students who were just learning the basic anatomy of the heart, this was an advanced topic. Although there was some perceived confusion during the presentation, students did not explicitly provide negative feedback within the end-of-course survey responses. For improvement, however, we would suggest that the focus remain on the abnormal sonographic features of tamponade which is, in its simplest form, fluid around the heart, and that fluid appears anechoic on ultrasound.

Going forward, more robust evaluation methods could also be implemented as well. The course evaluation survey completed by the learners in this case was mostly subjective, assessing mainly how each learner felt the course enhanced his or her own learning. Perhaps a future assessment tool could target the actual practical skills of learners before and after the course, asking them to look at a series of ultrasound clips of normal and pathologic findings and distinguish between the two. In this way, a direct, more objective assessment could be performed to better assess the achievement of the first educational objective (distinguishing between normal and pathologic anatomical findings). Future assessment methods could also focus on the learner's ability to obtain adequate images, which is the third educational objective. In the current course, this ability is assessed really only through observation by the facilitator within the small-group scanning portions. For more objective data, image acquisition could be assessed after the course by means of a practical exam. However, the main goal of implementing this adjunct course was to expose preclinical medical students to pathologic anatomical findings on ultrasound so that they may better understand normal anatomical findings, and therefore increase their confidence in, and develop a better understanding of, distinguishing between normal and pathologic findings. Because this course targeted students at such an early point in their training, before their clinical years and before any true need for objective assessments of achievement, we felt that our subjective assessment was adequate and provided enough information to determine the course's overall value to the students.

The four pathology sessions were designed to stand alone and could be presented to any preclinical medical student to enhance their understanding of basic anatomy. However, at our institution, we did implement the pathology sessions as an adjunct to a basic ultrasound course on normal anatomy. If chosen to be repeated as an adjunct to such a course, we would recommend that the pathology session be presented just after the main introductory didactic portion instead of after the scanning portion. One student provided this suggestion in the survey feedback at the end of the course. Indeed, this order switch would improve the overall flow and organization of the sessions. This would also minimize shuffling between rooms during each session. Further, it would allow learners to have more time to process and discuss this new material with the large amount of time dedicated to scanning practice with a small-group facilitator. We intend to make this change for future sections of this course.

Another potential benefit of implementing the pathology course as an adjunct to a basic ultrasound course is that students will already have a familiarity with basics of the ultrasound machine itself, including the different probes and their correct uses as well as basic settings, such as brightness, gain, and depth. In addition, they will also have a familiarity with basics of ultrasound image interpretation, such as the concept of echogenicity. While this background knowledge was useful for the preclinical medical student, an in-depth understanding of it is not necessary for the pathology courses, which granted them the ability to stand alone. The pathology course content already included instruction on the use of the correct probe for each exam presented, as well as instruction on how to obtain images for the corresponding exam. However, because the focus of the pathology course was on identifying sonographic pathology, preclinical students would really only need to understand the very basic concepts of ultrasound image interpretation. This would mainly only include the concept of echogenicity, which is already imbedded with the course content, as relevant to each case. It would be left at the discretion of the instructor whether to supplement this material based on the skill level of the learner. In fact, we do plan to emphasize the concept of echogenicity at the beginning of the first week session for future sections of this course.

Though most of our instructors and facilitators for the course were either emergency ultrasound fellowship-trained or had received advanced training in POCUS, because of the basic, general content contained in these sessions, only a basic understanding of POCUS would be needed for a facilitator to assist with the course. Resident physicians or fellows would make excellent course instructors. Most medical schools are

Copyright © 2020 Trembley and Radomski. This is an open-access publication distributed under the terms of the Creative Commons Attribution license.

beginning to incorporate ultrasound into their curriculum, and this pathology course can be utilized as an adjunct to any basic ultrasound course, but also has stand-alone potential as well. It could also very easily be adapted for medical students in their clinical years (third- or fourth-year students). For clinical students, more focus could be on the pathophysiology within each case and recognizing the use of POCUS in the appropriate clinical setting.

Appendices

A. Session 1 FAST Exam & the Trauma Patient.pptx
B. Session 2 Cardiac and Lung.pptx
C. Session 3 Gallbladder, Kidneys, & AAA.pptx
D. Session 4 Ocular US & Central Access.pptx
E. Session 1 Instructor Script.docx
F. Session 2 Instructor Script.docx
G. Session 3 Instructor Script.docx
H. Session 4 Instructor Script.docx
I. Survey Questions.docx

All appendices are peer reviewed as integral parts of the Original Publication.

Lauren Trembley, MD: Clinical Instructor, Department of Emergency Medicine, University of Pittsburgh

Marek Radomski, DO: Assistant Professor, Department of Emergency Medicine, University of Pittsburgh

Disclosures
None to report.

Funding/Support
None to report.

Ethical Approval
Reported as not applicable.

References

1. Walley PE, Walley KR, Goodgame B, Punjabi V, Sirounis D. A practical approach to goal-directed echocardiography in the critical care setting. *Crit Care*. 2014;18:681. https://doi.org/10.1186/s13054-014-0681-z
2. Blaivas M, Fox JC. Outcome in cardiac arrest patients found to have cardiac standstill on the bedside emergency department echocardiogram. *Acad Emerg Med*. 2001;8(6):616-621. https://doi.org/10.1111/j.1553-2712.2001.tb00174.x
3. Wilkerson RG, Stone MB. Sensitivity of bedside ultrasound and supine anteroposterior chest radiographs for the identification of pneumothorax after blunt trauma. *Acad Emerg Med*. 2010;17(1): 11-17. https://doi.org/10.1111/j.1553-2712.2009.00628.x
4. Engles S, Saini NS, Rathore S. Emergency focused assessment with sonography in blunt trauma abdomen. *Int J Appl Basic Med Res*. 2019;9(4):193-196. https://doi.org/10.4103/ijabmr.IJABMR_273_19
5. Barr L, Hatch N, Roque PJ, Wu TS. Basic ultrasound-guided procedures. *Crit Care Clin*. 2014;30(2):275–304. https://doi.org/10.1016/j.ccc.2013.10.004
6. Hatch N, Wu TS. Advanced ultrasound procedures. *Crit Care Clin*. 2014;30(2):305-329. https://doi.org/10.1016/j.ccc.2013.10.005
7. Bahner D, Goldman E, Way D, Royall NA, Liu YT. The state of ultrasound education in US medical schools: results of a national survey. *Acad Med*. 2014;89(12):1681-1686. https://doi.org/10.1097/ACM.0000000000000414
8. Narula N, Straus C, Feinstein K. Ultrasound for third- and fourth-year medical students. *MedEdPORTAL*. 2014;10:9786. https://doi.org/10.15766/mep_2374-8265.9786
9. Blackstock U, Carmody K. Transforming learning anatomy: basics of ultrasound lecture and abdominal ultrasound anatomy hands-on session. *MedEdPORTAL*. 2016;12:10446. https://doi.org/10.15766/mep_2374-8265.10446
10. Brown B, Adhikari S, Marx J, Lander L, Todd GL. Introduction of ultrasound into gross anatomy curriculum: perceptions of medical students. *J Emerg Med*. 2012;43(6):1098-1102. https://doi.org/10.1016/j.jemermed.2012.01.041

Received: September 10, 2019
Accepted: February 6, 2020
Published: September 25, 2020

Copyright © 2020 Trembley and Radomski. This is an open-access publication distributed under the terms of the Creative Commons Attribution license.
